# Discovery of novel targets for important human and plant fungal pathogens via an automated computational pipeline *HitList*

**DOI:** 10.1371/journal.pone.0323991

**Published:** 2025-06-03

**Authors:** David E. Condon, Brenda K. Schroeder, Paul A. Rowley, F. Marty Ytreberg

**Affiliations:** 1 Institute for Modeling Collaboration and Innovation, University of Idaho, Moscow, Idaho, United States of America; 2 Department of Software Engineering, College of Computer and Information Sciences, King Saud University, Riyadh 11543, Saudi Arabia; 3 Department of Biological Sciences, University of Idaho, Moscow, Idaho, United States of America; 4 Institute of Ocean and Earth Sciences, Universiti Malaya, C308, Institute of Advanced Studies Building, Kuala Lumpur 50603, Malaysia; 5 Department of Physics, University of Idaho, Moscow, Idaho, United States of America; KTH Royal Institute of Technology, SWEDEN

## Abstract

Fungi are a major threat to human health and agricultural productivity, causing 1.7 million human deaths and billions of dollars in crop losses and spoilage annually. While various antifungal compounds have been developed to combat these fungi in medical and agricultural settings, there are concerns that effectiveness is waning due to the emergence of acquired drug resistance and novel pathogens. Effectiveness is further hampered due to the limited number of modes of action for available antifungal compounds. To develop new strategies for the control and mitigation of fungal disease and spoilage, new antifungals are needed with novel fungal-specific protein targets that can overcome resistance, prevent host toxicity, and can target fungi that have no effective control measures. The increasing availability of complete genomes of pathogenic and spoilage fungi has enabled identification of novel protein targets essential for viability and not found in host plants or humans. In this study, an automated bioinformatics pipeline utilizing BLAST, Clustal Ω, and subtractive genomics was created and used to identify potential new targets for any combination of hosts and pathogens with available genomic or proteomic data. This pipeline called HitList allows *in silico* screening of thousands of possible targets. HitList was then used to generate a list of potential antifungal targets for the World Health Organization fungal priority pathogens list and the top 10 agricultural fungal pathogens. Known antifungal targets were found, validating the approach, and an additional eight novel protein targets were discovered that could be used for the rational design of antifungal compounds.

## Introduction

Fungal pathogens cause infectious disease in many organisms [1,2] among both animals [3] and plants [4]. Fungal pathogens of agriculture such as *Puccina* [5], *Botrytis* [6], and others [7] can be devastating to yields of many different crops [8] and endanger food security among a growing population [9]. In addition, the production of mycotoxins by fungi such as *Fusarium graminearum* [10] can contaminate agricultural products, [11] and threaten human and animal health if consumed [12,13]. Fungal pathogens in humans cause comparable mortality to tuberculosis and malaria [14]. The World Health Organization (WHO) recently published a list of the most concerning fungal pathogens, [15] highlighting the global concern over fungal infection. The repeated use of antifungals with limited number of modes of action results in an increase in resistant strains within fungal populations that are pathogens of humans or agriculture [16,17].

Fungal infections in humans can range from mild subcutaneous and mucosal infections [18] to life-threatening systemic disease. In particular, the rise of sophisticated medical devices, interventions, and therapeutics has increased vulnerable and immunocompromised patient populations that tend to be more susceptible to mycoses [19,20]. Populations that are especially vulnerable to fungal infections include prematurely born children [21] and AIDS patients, [22] leukemia, [23] and organ transplant patients [24]. Fungal infections can be a heavy cost for the health care system. For example, in 2018 there were 666,235 fungal infections were diagnosed in the United States alone [25–27]. Specifically, *Candida* yeasts are a leading cause of nosocomial fungal infections, particularly *Candida albicans*, which is a common fungal species associated with human mucosal surfaces [28]. Non-life-threatening infections include mucosal infections by *Candida* yeasts. Specifically, vulvovaginal candidiasis affects 75% of women at least once in their lifetime and is a major cause of morbidity, especially due to recurrent infections caused by drug-resistant species of yeasts [29]. Invasive fungal diseases can have high mortality rates, depending on the organisms. Recent outbreaks of mucomycoses associated with the COVID-19 pandemic have also highlighted the lack of effective fungal treatments, with mortality rates > 40% [30].

Agriculture incurs substantial losses from many different fungal pathogens in all regions of the world [31]. Infection by pathogens such such as *Pyricularia oryzae* and *Fusarium* species, that can cause devastating economic losses [10] costing farmers billions of dollars annually [32] in antifungal, i.e., fungicide, treatments. For example infection by *Pyricularia oryzae*, the causal organism of rice blast, can reduce rice productivity by 10-35%. *Ustilago maydis*, causing corn smut [33], can reduce corn yield by 2-20% [34]. Fungal plant pathogens can have a wide host range, such as *Botrytis cinerea* and *Colletotrichum truncatum* [35], or a narrow host range, such as *Blumeria graminis* [36]. Globalization can also introduce new fungal pathogens to agriculture from different regions of the world, potentially introducing new fungi for which plants and farmers have no experience [37], such as the wheat pathogen *Mycosphaerella graminicola*, which spread to the Americas and Australia over the last 500 years [38]. Similarly, wheat rust *Puccinia graminis tritici* strain Ug99 presents a major threat to food security, spreading from east Africa into Asia [39]. In addition to acting as direct pathogens, some fungi, such as saprophytes, can spoil food stores [40]. Repeated use of fungicides in agriculture also selects for resistance in *Aspergillus* to azoles [41]; this is problematic because azoles are used for both agricultural and human medicinal applications [42].

It is widely understood that there is a critical need for new classes of antifungal compounds [43,44]. Developing antifungal compounds with novel modes of action will provide more treatment options, reducing the need for repeated applications and selection for resistance. One key challenge in the development of new antifungal drugs is the lack of novel protein targets. An ideal compound should maximize harm against the pathogen and minimize off-target effects for the host organism, which can be accomplished by designing compounds to bind targets that are absent in the host. This can be challenging due to the shared eukaryotic ancestry and similar but not identical biochemical pathways [45] between fungi, plants, and humans. For example, within fungal systems, ergosterol serves many of the same cellular functions that cholesterol serves in humans, but is not present in animals, hence proteins in the ergosterol synthesis pathway have been targeted by many different antifungals (S1 Table). Similarly, azoles targeting 14α-demethylase [46,47] and morpholines targeting Erg2 and Erg24 in the ergosterol synthesis pathway are commonly used antifungals [48] (S1 Table). Likewise, the echinocandins target the pathway of cell wall β-(1,3) glucan synthesis [49], but are exclusively used against human and animal infections [50,51]. Because the cell wall is a unique structure to fungi, this pathway is absent in humans and leads to fungal osmotic instability and death [52]. Unfortunately, many of these antifungal compounds also have undesirable properties (S1 Table). Azoles are used against both human and agricultural pathogens [53]. Despite their potent activity against fungal pathways of ergosterol synthesis, they have been associated with hepatotoxicity and numerous hormone-related effects [54]. In addition, polyenes such as amphotericin B have severe renal toxicity [55,56]. Proliferation of resistance to current antifungal compounds further highlights the need for new modes of action [53]. Examples include *Candida*’s resistance to echinocandins [57] and flucytosine [58], azole resistance in *Aspergillus* [59] and *Candida* [60–62] and fungal species that are resistant to multiple types of antifungal drugs [63]. Some other minor classes of antifungals exist in addition to those mentioned above [64], and others used exclusively in agriculture [65].

Identification of target proteins can be done with subtractive genomics [66], which identifies potentially targetable molecules based on differences in alignment. “Subtractive genomics” can refer to protein sequences or to genomic pathways, or to genomic pathways where orthologs are removed [67], and will be referred to in the protein sense. This approach has been used to identify many potential protein targets in *Mycobacterium tuberculosis* [68], *Mycoplasma pneumoniae* [69], and *Mycoplasma genitalium* [70]. The only study that used subtractive genomics to identify anti-fungal targets has been on *Histoplasma capsulatum*, using the similar process called “reverse vaccinology” to identify drug and vaccine candidates [71]. However, most fungal pathogens have never been analyzed with subtractive genomics.

Computer-aided drug design, or CADD [72] has been used in the design and development of antifungals to treat human disease and it is significantly cheaper and faster for screening large libraries of compounds. Virtual screening is performed by using docking strategies to simulate the binding of a ligand with a given protein target [73], then applying various scoring functions to estimate the protein-ligand binding strength. A common method for developing antifungal compounds involves high-throughput empirical screening of large chemical libraries to identify possible antifungal candidates [74]. This process is expensive and time-consuming, with the added challenge that the cellular target of the compound is unknown [75,76]. CADD has been used to identify further inhibitors of known drug targets, but has yet to produce a viable antifungal drug [77]. At least 70 human drugs have been approved that used virtual screening as part of the drug discovery process, including Captopril, Norfloxacin, and Imatinib, but antifungals were not among them [78]. Recent advances in computational determination and approximation [79] of protein structure are also accelerating the pace of drug discovery due to the increased availability of protein structures.

In this study, a bioinformatics pipeline, HitList, was developed and used to identify possible antimicrobial targets for fungi. The purpose was to discover novel antifungal targets for the World Health Organization critical fungal pathogen list [15] and the top 10 agricultural fungal pathogens [10]. The current study started with a list of essential genes from the model fungus *Saccharomyces cerevisiae*. Essential genes with known human and/or plant orthologs were removed. Subtractive genomics [66] was then used to identify protein regions that could serve as targets. HitList surveyed more than a thousand proteins. HitList was validated by identifying proteins that already have known antifungal inhibitors, but we also found eight proteins that had not been previously considered as antifungal targets.

## Methods

### Source data and resources

Protein sequences encoded by essential genes from the Database for Essential Genes (DEG) [80] for *S. cerevisiae* (yeast) were downloaded from http://essentialgene.org. All hosts and pathogens are listed in Table 1. The pathogen targets were identified by utilizing the World Health Organization critical pathogen list [81] (S4 Table) and the top 10 agricultural fungal pathogen list (S5 Table) [10]). Proteome data was downloaded for common agricultural organisms that can be hosts for fungi *Homo sapiens* (human), *Glycine max* (soy), *Oryza sativa* (rice), *Solanum tuberosum* (potato), and *Zea mays* (corn) from https://ftp.ncbi.nlm.nih.gov/genomes/refseq/ (S3 Table). Pathogen proteomes were downloaded from from https://ftp.ncbi.nlm.nih.gov (S4 and S5 Tables).

**Table 1:** List of host and pathogen species used in this study. Source data for each species is indicated in S1 Table, ^A^host for agricultural pathogens, ^W^host for WHO pathogens.

Hosts	Human WHO pathogens	Ag. pathogens
*Glycine max* (soy)^A^	*Aspergillus fumigatus*	*Blumeria graminis*
*Homo sapiens* (human)^AW^	*Candida albicans*	*Botrytis cinerea*
*Oryza sativa* (rice)^A^	*Candida auris*	*Colletotrichum truncatum*
*Solanum tuberosum* (potato)^A^	*Candida parapsilosis*	*Fusarium graminearum*
*Zea mays* (maize, or corn)^A^	*Candida tropicalis*	*Mycosphaerella graminicola*
	*Cryptococcus neoformans*	*Puccinia graminis*
	*Histoplasma capsulatum*	*Puccinia striiformis*
	*Nakaseomyces glabratus*	*Puccinia triticina*
		*Pyricularia oryzae*
		*Ustilago maydis*

### Bioinformatics pipeline

**Fig 1:**
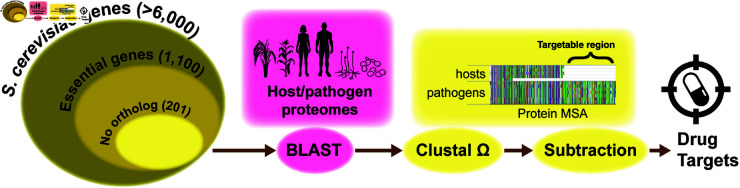
Graphical representation of HitList, the bioinformatics pipeline that identifies potential drug targets. Considering only genes that have no human ortholog according to the Saccharomyces Genome Database (SGD) [88], the field of essential genes was narrowed to 201. These genes are then used as BLAST [89] queries against all pathogen and host proteomes (S1 Section). Clustal Ω was then used to create MSAs to identify targetable regions via subtractive genomics [66].

Our HitList pipeline starts with all genes from *S. cerevisiae* as obtained from the DEG [82]. Of the 1,110 that were considered essential, 909 genes that have human orthologs according to the Alliance of Genome Resources [83] were eliminated from consideration (Fig 1). The remaining 201 genes were used as queries in a BLASTP analysis using default parameters [84] (version 2.14) against all hosts and pathogens proteomes. Proteins with homology to query proteins having an expectation value above 0.1 were excluded. For each query protein, the top resulting protein from each pathogen and each host BLAST analysis was placed together and aligned with Clustal 1.2.4 [85] to obtain multiple sequence alignments (MSAs). MSA regions that show alignment with pathogens and not with hosts are considered potentially targetable, using an approach that has previously been called “subtractive genomics” [66]. This approach identifies amino acid sequences that are likely only present in the pathogens, and absent in the hosts, avoiding effects on the host. Analgous to a study showing that amino acid sequence could determine whether or not an immunoglobulin E protein would bind to casein for milk-allergic patients [86], we sought to identify amino acid regions of proteins that would be susceptible to any sort of potential inhibitor or antibody.

**Fig 2:**
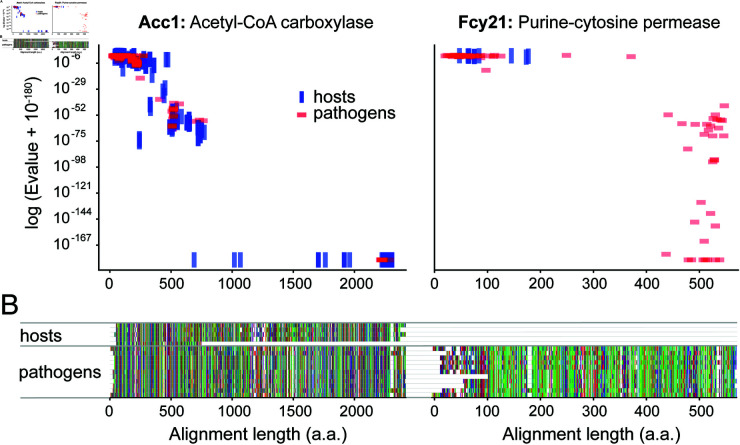
Representative output from the HitList pipeline. An example of two essential *S. cerevisiae* proteins analyzed for target suitability among the top 10 agricultural fungal pathogens. The figure shows results from BLAST searches performed for each essential gene with alignment statistics shown. **(A)** Acc1 is an example of a poor target that shows high similarity between homologous proteins of hosts and pathogens, while on the right Fcy21 is an example of a potentially good target that shows neither desirable expectation values nor long alignment lengths with host proteins. **(B)** The desirable alignment between host and pathogen proteins for Acc1, and the lack of host proteins for Fcy21 is visible in multiple sequence alignments, which are visualized using a modified version of CIAlign [90].

Fig 2 shows an example comparing two proteins, one with a poor targetability, and another with desirable targetability. The targetability of a region is quantified by the number of pathogens present at each residue in the MSA, the more pathogen proteins are present within a given region, the better that region is for a target. While it is beyond the scope of this study, proteins with known experimental, e.g., NMR or X-ray, structures are preferred, as eventual structure-based drug design [87] would have a more reliable starting point for design of antifungal compounds. Additionally, protein location within the cell is also important, as drugs must be able to bind the target protein wherever it is found in the cell.

The similarity of the amino acids within each MSA was quantified by Sneath’s similarity index [91] (S2 Table). Sneath’s ϕ was chosen because of its intuitive nature, in that it ranks within (0,1], and an amino acid compared with itself can be exactly 1. The Sneath index is calculated at each position within an MSA by comparing every amino acid in a group, against every other amino acid in that group:

$$\varphi =\sum_{i}^{N_{seq}}\sum_{j}^{N_{seq}}\frac{M_{ij}}{N_{seq}(N_{seq}-1)}$$ (1)

where *M* values for two amino acids *i* and *j* are given by the Sneath similarity in S2 Table, and *N*_*seq*_ is the number of sequences at that position. For example, consider two peptides, each containing nine alanine residues. In this case, all Sneath similarity values would be 1.0. Comparing a 9-mer of alanine against a 9-mer of cysteine would give a Sneath similarity of 0.87 across the 9 residues. Uncertain amino acids, e.g. “X” are set to the minimum Sneath value of 0.003 to avoid type II errors. If a known amino acid is compared with an ambiguous amino acid, e.g. A with B, where B could be either D or N, then the Sneath index of A and B is set at the mean of *M*_*A*/*D*_ and *M*_*A*/*N*_.

## Results & Discussion

**Fig 3:**
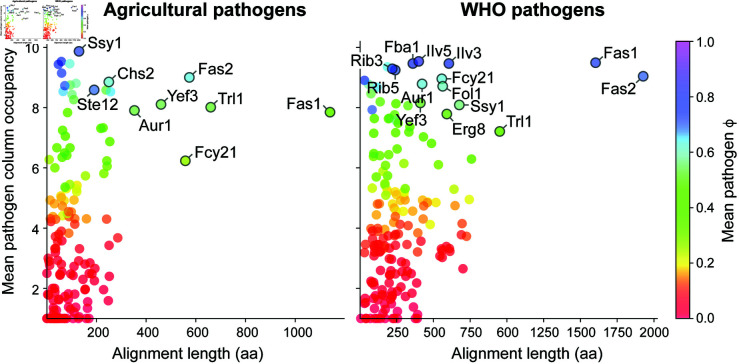
Visual representation of HitList output for agricultural and WHO pathogens. The output of the pipeline is visualized by a scatterplot of alignment length, mean pathogen column occupancy, and ϕ (Eq 1). Labeled proteins are those with long targetable regions, and large ϕ and mean column occupancy values.

In this study, our newly developed bioinformatics pipeline HitList was used to identify antifungal protein targets against two groups of pathogens: WHO fungal pathogens (S4 Table), and agricultural pathogens (S5 Table). Desirable qualities in protein targets include (e.g., “Fcy21” in Fig 2): (1) High bit scores and longer alignment lengths to an essential protein for *S. cerevisiae* and not to any host protein. (2) Homogeneity as measured by ϕ approximately within the range 0.7≤ϕ≤1. That is, proteins in the top right corners of Fig 3 represent the best preliminary targets. Proteins in the top left will have shorter targetable regions, but could be potential targets. Conversely, proteins that have good alignment between both host and pathogen proteins and to the *S. cerevisiae* essential protein, especially over the entire length, are considered less desirable targets (e.g., “Acc1” in Fig 2).

Table 2 summarizes our list of most promising potential protein targets. Of the 16 proteins in the table, seven are in common between WHO and agricultural fungal pathogens and thus could be targets that lead to the development of broad-spectrum antifungal compounds. Detailed figures and tables are shown for each protein in Table 2, sorted alphabetically in S2 Section, containing S1-S197 Figs and S6-S39 Tables. While the potential target list is greater than these 16 proteins, these are the targets that met our criteria listed above in Fig 3 and hence further analysis will focus on the proteins listed in Table 2. A larger number of possible protein targets were found for the WHO pathogens than for agricultural pathogens, which is expected because agricultural hosts include not only human but also a wide phylogenetic range of plants, as compared to WHO pathogens that only include human as a host.

**Table 2:** Summary of potential antifungal protein targets for both agricultural and human pathogens. The UniProt IDs are provided in the second column. Protein length is in the third column in number of amino acids. The fourth column contains information summarized from the SGD and UniProt [88,107]. Literature references citing these targets are in the last column; if no literature reference citing a protein as an antifungal target could be found, then “none” was entered. More detailed information is available in S2 Section . ^A^agricultural pathogens, ^W^WHO pathogens.

Protein	UniProt	Length	Summary	Lit.
Alr1^AW^	Q08269	859	Plasma membrane ${\text{Mg}}^{2+}$ transporter	[92]
Aur1^AW^	P36107	401	Inositol phosphoceramide synthase	[93]
Chs2^A^	P14180	963	Chitin synthase II; catalyzes transfer of N-acetylglucosamine to chitin	[94–96]
${\text{Erg8}}^{\text{W}}$	P24521	451	Phosphomevalonate kinase; contributes to ergosterol biosynthesis	none
Fas1^AW^	P07149	2051	β Subunit of cytoplasmic fatty acid synthase	[97–99]
Fas2^AW^	P19097	1887	α Subunit of cytoplasmic fatty acid synthase	[97–99]
Fba1^W^	P14540	359	Fructose 1,6-bisphosphate aldolase	[100]
Fcy21	P40039	528	Nucleobase membrane transporter	none
Fol1^W^	P53848	824	Multifunctional enzyme of the folic acid biosynthesis pathway	[101]
Ilv3^W^	P39522	585	Dihydroxy-acid dehydratase for branched-chain amino acid biosynthesis	none
Ilv5^W^	P06168	395	Ketol-acid reductoisomerase for branched-chain amino acid biosynthesis	none
Rib3^W^	Q99258	208	Cytosolic synthase involved in riboflavin biosynthesis	none
Rib5^W^	P38145	238	Riboflavin synthase	none
Ssy1^AW^	Q03770	852	Amino acid sensor	none
Ste12^A^	P13574	688	DNA binding transcription factor	none
Trl1^AW^	P09880	827	tRNA ligase	[102–104]
Yef3^AW^	P16521	1044	Translation elongation factor 3	[105,106]

### Identification of previously identified targets validates the pipeline

Fig 3 shows that HitList identifies previously known targets that already have known inhibitors, detailed information of which is included in S3 Section, containing S198-S205 Figs and S40-S45 Tables. HitList selected Fas1 and Fas2 (S2.5 and S2.6 Sections); these subunits of fatty acid synthase [108] have been previously identified as viable targets against fungi, and inhibitors have already been found [97–99], serving as a validation of HitList. Fas1 is the specific target of the antifungal compound NPD6433 [98,109]. Our analysis suggests Fas2 is a better target for WHO pathogens compared to agricultural pathogens, because host plant proteins show weakly aligned sequences, and fungal sequences also align more weakly (S58 and S61 Figs). Indeed, the natural products CT2108A and CT2108B are effective antifungals against the FAS complex in *Candida*, *S. cerevisiae*, and *Cryptococcus*, yet both are made by the fungus *Penicillium solitum*, so broad antifungal activity is less likely. Fas1 BLAST hits for the plant pathogens *P. graminis*, *P. triticina*, and *P. striiformis* are much weaker than for other agricultural pathogens, as demonstrated by the lower ϕ value in Figs 3, as well as weaker alignments in S50 and S51 Figs. BLAST searches against the non-redundant protein database show that Fas1 is broadly conserved across many different genera of fungi from Asomycota, Basidomycota, and Zoopagomycota (S57 Fig) and Fas2 (S69 Fig). Table 2 also shows Chitin synthase 2 (Chs2) as a possible agricultural target, and this protein already has known inhibitors including nikkomycins and polyoxins [94–96]. However, Chs2 may not be a good choice for drug targeting due to strong homology to the human protein hyaluronan synthase. Nonetheless, chitin synthase (see Section S2.3) has no equivalent in humans or plants according to the SGD, agreeing with a search against the NCBI’s non-redundant database [110] (NR) (see S29 & S34 Figs). Even if Chs2 were successfully targeted, a Chs2-knockout strain of the human pathogen *C. albicans* did not have attenuated virulence [111], so targeting Chs2 with potential therapeutics may not be effective in treatment of infections. Finally, the plasma membrane ${\text{Mg}}^{2+}$ transporter Alr1 [112] was identified in the pipeline, which has been shown to be a potential antifungal target and is known to be inhibited by Bovine pancreatic trypsin inhibitor [92]. Folate synthesis (Fol1) is an effective target of antibacterials, such as trimethoprim and sulfamethoxazole [113], and has been effective in fungi too [114].

HitList also identified some proteins that have been previously suggested as potential antifungal targets in the literature but have no known inhibitors. Proteins that are part of chemical pathways that are absent in hosts are especially attractive, as the chance of side effects is significantly lower. For example, ceramide phosphoinositol transferase, part of sphingolipid synthesis (Aur1), [115] and fructose 1,6-bisphosphate aldolase (Fba1) [100] were identified as potential antifungal targets by this analysis and others, and do not have mammalian equivalents [93,115].

The pipeline identified Trl1, which is responsible for the splicing of introns from nascent tRNA as a potential target. Since this is done very differently in fungi compared to metazoa, Trl1 is a possible antifungal target [102–104,116]. Yeast Elongation factor 3 (Yef3 or eEF3) is very well conserved among fungi [105,117] and identified as a potential target using this pipeline. Because non-fungi use only two elongation factors when translating mRNA, as opposed to three for fungi, Yef3 has potential as a target [106,118] Indeed, S192 & S197 Figs both show strong conservation of Yef3 across the fungal kingdom, however, this study also reveals 960 metazoan species with strong hits (S196 Fig). This makes intuitive sense because Yef3 contains ATP-binding cassette domains [119] that are very common throughout many domains of life (S39 Table), and inhibitors must be carefully designed to avoid unintentional effects on host organisms. Further, results suggest that Trl1 and Yef3 are less likely to be broad spectrum targets as can be seen with the relatively low ϕ and weaker MSA (Figs 3, S176, S186), compared to other proteins identified in the pipeline.

### Identification of eight novel antifungal protein targets

Most importantly, our study has also uncovered a rich variety of novel possible protein targets that have not been previously identified to our knowledge (Table 2): Erg8, Fcy21, Ilv3, Ilv5, Rib3, Rib5, Ssy1, and Ste12. As will be detailed below, some of these targets are particularly well-suited for WHO pathogens and some for agricultural pathogens, while others appear to be candidates for antifungal development against both pathogen lists.

HitList identified potential broad-spectrum antifungal target proteins that are particularly well-suited for treating pathogens in the WHO pathogen list. The protein pair Ilv3 and Ilv5, [120] involved in branched chain amino acid synthesis, [121,122] is an especially attractive target because 1) no human proteins have strong alignments via BLAST, 2) there is a high ϕ value between pathogen hit sequences, and 3) branched chain amino acid synthesis does not occur in metazoa [123]. The last point is critical since inhibition of a protein pathway that is present in a pathogen, but not in a host, is unlikely to cause harm to the host. The dihydroxyacid dehydratase enzyme performs the same chemistry as Ilv3 in cyanobacteria and is inhibited by aspterric acid [124]. Further experimentation is necessary to determine whether aspterric acid or related derivatives have potential to function as a broad-spectrum antifungals. The pipeline also identified Rib3 and Rib5 as attractive protein targets for the WHO pathogen list, with high ϕ values. These proteins function in riboflavin synthesis, which does not occur in metazoa. Our study suggests that Ilv3, Ilv5, Rib3 and Rib5 are not as suitable for developing agricultural antifungals as for humans, because riboflavin [125] and branched chain amino acid synthesis [122] also occurs in plants and could potentially lead to phytotoxicity (see S136 and S151 Figs).

Ssy1 is an amino acid sensor protein that has potential for broad-spectrum use against both agriculture and WHO pathogens, but appears to be better suited for agricultural application. This apparently novel antifungal target shows much stronger alignment with *Candida* pathogens than *Cryptococcus* (S154 Fig), which reduces the ϕ value within WHO pathogens. Furthermore, *Nakaseomyces* shows great differences with *Candida* and *Cryptococcus* sequence hits with Ssy1 (S153 and S154 Figs). BLAST hits against the Non-Redundant protein database animal and plant proteins are few and weak (S159 and S164 Figs), so any well-designed drug against Ssy1 would be unlikely to affect animals and plants. Furthermore, as a membrane protein, drug design need not be concerned with membrane penetration.

Fcy21, for “flucytosine resistance,” is another potential novel protein target for antifungal compounds, which has potential to be an antifungal target for WHO human pathogens. Fcy21 is a putative purine-cytosine permease [126,127]. This protein is related to the target of flucytosine (Table 2) Fcy2p, but cannot substitute for its function [126] No human protein showed significant alignment with Fcy21 (S82 Fig), but strong conservation is seen among pathogenic fungi, apart from the first 100 residues in the N-terminal domain. No non-fungal orthologs exist according to the SGD, and searches against the non-redundant database indicate strong conservation among WHO pathogenic fungi, and no hits among metazoa and insignificant hits among plants (S92 Fig). Interestingly, Fcy21 does not have homology with proteins from three of the agricultural fungal pathogens of agricultural products, namely *Puccina* and *P. oryzae* (S85 Fig and S21 Table), hence Fcy21 is not likely to be as useful for developing agricultural antifungals. Ste12 was identified by HitList for agricultural pathogens, but not for human pathogens, and was not labeled due to weak alignment with the model *S. cerevisiae* (Fig 3). Ste12, a transcription factor that is important for the virulence of mycoparasites, including *Trichoderma atroviride* which is a parasite of plant pathogens, [128,129] shows good antifungal potential for the top 10 agricultural fungal pathogens (S2.15 Section). Ste12 is one of the two proteins that is a good target against agricultural pathogens, but not against human pathogens (S165 and S168 Figs).

Our study shows that agricultural host proteomes have much stronger hits to Erg8 (S38 Fig) than human (S35 Fig), so any antifungal drug targeting of Erg8 would likely be more effective against human pathogens. Erg8 is part of the ergosterol synthesis pathway, enabling phosphomevalonate kinase activity. While the ergosterol synthesis pathway is well-studied and targeted by azole [47,130] and morpholine antifungals [131], Erg8 appears to be a novel potential target. The difference in primary sequence of the Erg8 homolog from *Cryptococcus neoformans* compared to the homologs from other pathogen species is significant, with large gaps in the multiple sequence alignment because of *C. neoformans* (S35 Fig).

### Limitations of the study

The primary limitation of the pipeline is that it relies on the list of essential genes. Here, essential genes for *S. cerevisiae* from DEG that do not have human orthologs according to the SGD are used. If a gene is not on this list, this gene will not be able to identified as a target. For example, consider Fks1, the target of echinocandin antifungals. Fks1 does not appear on our list because the β-1,3-glucan synthase proteins (Fks1, Fks2, Fks3) are not considered essential genes according to the DEG [80]. Nonetheless, BLAST analysis of Fks1 against the non-redundant database identifies protein homologs in 1,149 fungal species (S203 Fig), and a multiple sequence alignment of these demonstrates that Fks1 is an ideal target (S202 Fig), as there are very large regions of the protein in the MSA that only have alignment to pathogen protein domains. This shows that Fks1 would have appeared on our final list of target proteins if it was in the initial pool of genes considered. Such results should be expected, as fungal cell wall synthesis is unique to fungi [132]. Furthermore, if a gene is essential in the model, but isn’t essential in the target pathogen, for example Chs2, HitList will identify a false positive.

Another potential weakness of HitList is our elimination of human orthologs. While this step reduces the chances of side effects in a resulting therapeutic, it could also obscure potential targets. For example, the antimalarial drug methylene blue targets the glutathione reductase protein in *Plasmodium falciparum* [133], in spite of a very similar protein being present in the human host. However, HitList is designed with caution in mind, and avoiding potential side effects to increase the chance of developing a successful therapeutic.

HitList also requires a well-characterized organism closely related to target pathogens such as *S. cerevisiae*, with an annotated genome and/or proteome, and a list of essential genes. For this study, NCBI had all desired pathogens available. However, niche organisms may not necessarily be available with the necessary quality for analysis. This study was possible due to the very high quality genome annotation of *S. cerevisiae*, and the availability of the pathogen data from NCBI.

HitList is designed to avoid possible effects on the host, and does so at the possible risk of missing otherwise good targets. For example, Erg11, the target of azole antifungals (S1 Table), has a high homology to a human protein, which can be further seen (S198 Fig). Indeed, human lanosterol and non-targeted cytochrome P450 are also affected by azole antifungals [134]. Similarly, Erg24 is a target of the morpholine antifungals, and shows a desirable sequence similarity with delta(14)-sterol reductase, and has human orthologs [83].

Evaluation of HitList cannot be evaluated by usual measures such as false positives, true negatives, etc., as no quantifiable metric exists for what constitutes a targetable protein, and personal opinion may differ from one individual to another. Databases are not necessarily consistent with proteins that are considered human homologs and not, so which proteins should be fed into the pipeline is not always clear. Also, few organisms are as well annotated as humans and *S. cerevisiae* are. Potatoes for example, and do not necessarily have an exact equivalent in the database for each protein. Furthermore, the lengths that are found targetable for each protein may be usable for some proteins, but not others.

Collateral damage of targeting potential proteins should also be taken into account. For example, symbiotic bacteria and mycorrhizal fungi [135,136] were not included in this study’s host-pathogen relationship. Indeed, the location of the symbiotes and which part of the plant is affected by the pathogen should be considered, i.e. whether applied to leaves, roots, etc. Additionally, neither the impact on insects that prey on herbivores [137], nor downstream environmental impacts were considered. Off-target damage can be minimized by considering proteins that are fungal-specific and have no equivalent in any animal or plant, for example Yef3. Chs2, for example, although not recommended for targeting here, has equivalents in insects and fish.

We note that our strategy for validating HitList is its ability to find previously identified targets such as Fas1 and others. Ideally, it would be possible to quantify HitList’s performance in terms of accuracy and precision, however, this is not possible for the following reasons: 1) Essential gene lists can differ depending on how many are available for the model in question, depending on methods used for determination of essentiality [138], so an exact determination of HitList’s accuracy, precision, etc. is not possible. 2) The particular model organism used may not be appropriate for the pathogens in question. For example, fungi have at least two well-characterized species, *S. cerevisiae* and *S. pombe*, and for some pathogenic fungi, one model or the other may be appropriate, adding uncertainty. 3) The exact amino acid length necessary to target a protein can vary depending on antibody or inhibitor design capability, further eroding any clarity between what constitutes a true and false positive. 4) What constitutes an “ortholog” can vary between databases, and if A is an ortholog of B in one database, B is not guaranteed to be an ortholog of A in another database. 5) Stronger genomic data in the future could lead to changes in what is considered targetable.

In order to use CADD techniques, the identified target proteins should also be well-characterized structurally, e.g. by NMR or X-ray crystallography. Proteins should have identifiable binding pockets to be used for computational chemistry docking programs, so that potential inhibitors can be screened [139]. Many proteins in Table 2, such as Ssy1 and Fcy21, do not have any experimental structures available as of publication, while proteins such as Yef3 does have an X-ray structure available [106]. The paucity of experimental structural data in the Protein Data Bank [140] for the proteins that are found to be attractive targets here also precludes 3-dimensional structural searches, such as Dali [141] and FoldSeek [142], and harms AlphaFold [79] predictions by having an inadequate training set. The inability to perform structural searches against potential targets means that we do not have a means to check whether similar binding pockets may exist in host proteins, leading to possible off target effects for the resulting treatment. While AlphaFold structures can provide good estimates, experimental structures are preferred. Future research of possible protein targets should consider the presence and quality of existing structures.

### Diversity of pathogens and hosts hinders target selection

The phylogenetic diversity of the ascomycetes is very wide [143], complicating identification of targets. Consider the large evolutionary distance between the two ascomycete genera *Saccharomyces* and *Schizosaccharomyces*, which is on the order of 350 million years of divergence [144]. Indeed, *Cryptococcus* and *Puccina* are both genera within basidomycota, which is in an entirely different phylum than *S. cerevisiae’*s ascomycota, and at an even greater evolutionary distance than between *Schizosaccharomyces* and *Saccharomyces*. No basidomycete has the quality of annotation that *S. cerevisiae* has and a known essential gene list, so an ascomycete pathogen is the best choice at present. Nonetheless, potential protein targets were found that included the basidomycetes, indicating conservation of genes over very long periods in distantly related phyla [145].

The number of hosts/pathogens and their identities affects the possible protein targets and the length of the targetable regions identified by the pipeline. Consider Ssy1, which has a much longer targetable section for the WHO pathogens than for the top 10 agricultural pathogens (Fig 3 and S2.14 Section). An MSA of the Ssy1 homologs from the top 10 agricultural pathogen and respective host proteomes shows large gaps, reducing the potential target length (S156 Fig). Gaps also exist for Ssy1 homologs from the WHO pathogen proteomes (S156 Fig) in approximately the same regions as for the Ssy1 homologs from the top 10 agricultural pathogens, but the homologs from the *Candida* species and *N. glabratus* proteomes exhibit homology across these gaps, lengthening the potential region that could be targeted with antifungal compounds. For the agricultural pathogens, the plant proteins within the MSA shorten the targetable region. The larger the number of hosts and pathogens that are included in the analysis, and the more diversity within each group, the fewer and shorter the targets will be for antifungal compounds.

The length of targetable proteins is reduced by increasing diversity of pathogens and hosts, which is a consequence of diversity in primary sequence. Consider, for example, using only *Candida* pathogens in the WHO list for HitList. When this analysis is performed, Ccc1 shows as targetable, but does not show as targetable when the entire WHO list is considered. Ccc1 [146] (Section S4.1, S206-S212 Figs, S46-S47 Tables) is a ${\text{Mn}}^{2+}$/${\text{Fe}}^{2+}$ transporter that has previously been identified as a potential antifungal target [147]. Indeed, Ccc1 is particularly attractive because no metazoan equivalent exists, and *Candida* is a human pathogen. *Cryptococcus* proteins clearly cluster differently in the MSA from the other fungal genera (S206 and S207 Figs), which is intuitive as *Cryptococcus* is a basidomycete, while all other pathogens are ascomycetes. *Candida* pathogen proteins clearly show both desirable alignment to one another and to *S. cerevisiae*, while *Cryptococcus* shows its own cluster that doesn’t align strongly to *S. cerevisiae* (S207 Fig). Strong homology exists between the *Candida* proteins and *S. cerevisiae*, making more probable that this protein is essential in *Candida* as well, agreeing with S207 Fig. Inspection of S206 Fig implies that the broad-spectrum targeting region is approximately from residue 110-170 in MSA coordinates, a narrow region of the protein to target with a compound. Given that antibodies can target decapeptides [86], Ccc1 is a possible target for *Cryptococcus*, but development of an antifungal drug could be challenging. By contrast, using only *Candida* species as pathogens suggest that residues 100-340 are targetable (S206 Fig), which could simplify compound design. Thus, when using HitList, the number of pathogens should be kept to a minimum, and the pathogens that are used should be as closely related to one another as possible.

### Conclusion

A new automated bioinformatics pipeline (HitList) that uses subtractive genomics to identify protein targets in parallel for any pathogen-host combination was developed, able to test thousands of proteins within a few hours on personal computers. HitList was applied to the discovery of novel antifungal targets for the WHO critical list of fungal pathogens and the top 10 agricultural fungal pathogens, using essential genes from *S. cerevisiae*. Current antifungal targets, such as Fas1 and Fas2, and previously hypothesized antifungal targets such as Trl1 and Yef3 were identified as potential targets by HitList. Both lists of pathogens and hosts showed established targets. Many previously identified targets that were identified do not yet have inhibitors, such as Alr1, Aur1, and Trl1. Most importantly, HitList identified novel protein targets that could be used to develop broad-spectrum antifungals that have not been previously mentioned in the literature, to our knowledge: Erg8, Fcy21, Ilv3, Ilv5, Rib3, Rib5, Ssy1, and Ste12. Based on our analysis, we anticipate that some of these targets will be more well-suited for developing antifungals against the WHO list (Erg8, Ilv3, Ilv5, Rib3, and Rib5), Ste12 against the agriculture list only, and others against both lists (Fcy21 and Ssy1). In addition, stronger potential targets are identified for the WHO/human pathogen list, since it has a single host, as compared to the agricultural top 10 list, that has five hosts across two different domains of life. While HitList identified proteins that could potentially be targets of compounds for antifungal purposes (Table 2), more research is clearly needed in order to validate these targets and develop possible inhibitors that could lead to new classes of antifungal drugs. Finally, we note that HitList could be used to investigate any list of pathogens and hosts for any kingdom of life, provided that a list of essential genes exists, and genomes and/or proteomes are available. The HitList software to run the bioinformatics pipeline and identify potential protein targets is freely available at https://github.com/hhg7/HitList.

## Supporting information

Supporting Information Supporting Information files (PDF).
