# Correction: Discovery of novel targets for important human and plant fungal pathogens via an automated computational pipeline *HitList*

**DOI:** 10.1371/journal.pone.0335482

**Published:** 2025-10-29

**Authors:** The PLOS One Staff

## Notice of Republication

An incorrect version of the funding statement was published in error. This article was republished on October 6, 2025 to correct this error. Please download this article again to view the correct version. The publisher apologizes for the error.
