# Supplementary material for: Discovery of novel targets for important human and plant fungal pathogens via an automated computational pipeline HitList
Source: PLoS One. 2025 Jun 3;20(6):e0323991. doi: 10.1371/journal.pone.0323991 (PMC12132981; doi:10.1371/journal.pone.0323991)
Supplement: Supporting Information — (PDF). [file pone.0323991.s001.pdf]

Supporting Information

David E. Condon

February 19, 2025

Contents

S1 Data Sources **S9**

S2 Results: Best Targetable Proteins **S10**

S2.1 Alr1 . . . S10
S2.1.1 WHO Critical Pathogens . . . S10
S2.1.2 Top 10 Agricultural Fungal Pathogens . . . S12
S2.1.3 NR . . . S15
S2.2 Aur1 . . . S21
S2.2.1 WHO Critical Pathogens . . . S21
S2.2.2 Top 10 Agricultural Fungal Pathogens . . . S23
S2.2.3 NR . . . S26
S2.3 Chs2 . . . S32
S2.3.1 WHO Critical Pathogens . . . S32
S2.3.2 Top 10 Agricultural Fungal Pathogens . . . S34
S2.3.3 NR . . . S37
S2.4 Erg8 . . . S43
S2.4.1 WHO Critical Pathogens . . . S43
S2.4.2 Top 10 Agricultural Fungal Pathogens . . . S45
S2.4.3 NR . . . S48
S2.5 Fas1 . . . S54
S2.5.1 WHO Critical Pathogens . . . S54
S2.5.2 Top 10 Agricultural Fungal Pathogens . . . S56
S2.5.3 NR . . . S59
S2.6 Fas2 . . . S65
S2.6.1 WHO Critical Pathogens . . . S65
S2.6.2 Top 10 Agricultural Fungal Pathogens . . . S67
S2.6.3 NR . . . S70
S2.7 Fba1 . . . S76
S2.7.1 WHO Critical Pathogens . . . S76
S2.7.2 Top 10 Agricultural Fungal Pathogens . . . S78
S2.7.3 NR . . . S81
S2.8 Fcy21 . . . S87
S2.8.1 WHO Critical Pathogens . . . S87
S2.8.2 Top 10 Agricultural Fungal Pathogens . . . S89
S2.8.3 NR . . . S92
S2.9 Fol1 . . . S98
S2.9.1 WHO Critical Pathogens . . . S98
S2.9.2 Top 10 Agricultural Fungal Pathogens . . . S100
S2.9.3 NR . . . S103
S2.10 Ilv3 . . . S109
S2.10.1 WHO Critical Pathogens . . . S109
S2.10.2 Top 10 Agricultural Fungal Pathogens . . . S111
S2.10.3 NR . . . S114
S2.11 Ilv5 . . . S120
S2.11.1 WHO Critical Pathogens . . . S120

S2.11.2 Top 10 Agricultural Fungal Pathogens S122
S2.11.3 NR S125
S2.12 Rib3 . . . . . S131
S2.12.1 WHO Critical Pathogens S131
S2.12.2 Top 10 Agricultural Fungal Pathogens S133
S2.12.3 NR S136
S2.13 Rib5 . . . . . S142
S2.13.1 WHO Critical Pathogens S142
S2.13.2 Top 10 Agricultural Fungal Pathogens S144
S2.13.3 NR S147
S2.14 Ssy1 . . . . . S153
S2.14.1 WHO Critical Pathogens S153
S2.14.2 Top 10 Agricultural Fungal Pathogens S155
S2.14.3 NR S158
S2.15 Ste12 . . . . . S164
S2.15.1 WHO Critical Pathogens S164
S2.15.2 Top 10 Agricultural Fungal Pathogens S166
S2.15.3 NR S169
S2.16 Trl1 . . . . . S175
S2.16.1 WHO Critical Pathogens S175
S2.16.2 Top 10 Agricultural Fungal Pathogens S177
S2.16.3 NR S180
S2.17 Yef3 . . . . . S184
S2.17.1 WHO Critical Pathogens S184
S2.17.2 Top 10 Agricultural Fungal Pathogens S186
S2.17.3 NR S189

S3 Previously identified protein targets **S195**
S3.1 Erg11 . . . . . S195
S3.2 Erg24 . . . . . S197
S3.3 Erg2 . . . . . S198
S3.4 Fks1 . . . . . S199
S3.5 Fks3 . . . . . S201
S3.6 Gsc2 . . . . . S202

S4 Results: Genus-specific Good Targets **S203**
S4.1 Ccc1 . . . . . S203
S4.1.1 WHO Critical Pathogens S203
S4.1.2 Aspergillus S205
S4.1.3 Candida S205
S4.1.4 Cryptococcus S205
S4.1.5 Top 10 Agricultural Fungal Pathogens S206

List of Tables

S1 Major current human anti-fungal compound classes and their targets, all genes refer to *Saccharomyces cerevisiae*. S9
S2 Similarity between amino acids can be quantified by Sneath's index ϕ, where identical amino acids have a value of 1, and the greater the index between two amino acids is, the more similar the amino acids are to one another.. S9
S3 Species and the data sources used for hosts. The Top 10 agricultural pathogens group used every species here, while the WHO pathogens only used *H. sapiens*. S9
S4 World Health Organization fungal pathogens and the data sources used. S10
S5 Top ten agricultural pathogens and the data sources used. S10
S6 Pairwise alignment info from yeast Alr1 (DEG20010935), cf. Figure S1. . . . . S12
S7 Pairwise alignment info from yeast Alr1 (DEG20010935), cf. Figure S4. . . . . S14
S8 Pairwise alignment info from yeast Aur1 (DEG20010598), cf. Figure S12. . . . . S23
S9 Pairwise alignment info from yeast Aur1 (DEG20010598), cf. Figure S15. . . . . S25
S10 Pairwise alignment info from yeast Chs2 (DEG20010039), cf. Figure S23. . . . . S34

S11 Pairwise alignment info from yeast Chs2 (DEG20010039), cf. Figure S26. . . . S36
S12 Pairwise alignment info from yeast Erg8 (DEG20010822), cf. Figure S35. . . . S45
S13 Pairwise alignment info from yeast Erg8 (DEG20010822), cf. Figure S38. . . . S47
S14 Pairwise alignment info from yeast Fas1 (DEG20010641), cf. Figure S47. . . . S56
S15 Pairwise alignment info from yeast Fas1 (DEG20010641), cf. Figure S50. . . . S58
S16 Pairwise alignment info from yeast Fas2 (DEG20011054), cf. Figure S58. . . . S67
S17 Pairwise alignment info from yeast Fas2 (DEG20011054), cf. Figure S61. . . . S69
S18 Pairwise alignment info from yeast Fba1 (DEG20010617), cf. Figure S70. . . . S78
S19 Pairwise alignment info from yeast Fba1 (DEG20010617), cf. Figure S73. . . . S80
S20 Pairwise alignment info from yeast Fcy21 (DEG20010294), cf. Figure S82. . . . S89
S21 Pairwise alignment info from yeast Fcy21 (DEG20010294), cf. Figure S85. . . . S91
S22 Pairwise alignment info from yeast Fol1 (DEG20010889), cf. Figure S93. . . . S100
S23 Pairwise alignment info from yeast Fol1 (DEG20010889), cf. Figure S96. . . . S102
S24 Pairwise alignment info from yeast Ilv3 (DEG20010579), cf. Figure S105. . . . S111
S25 Pairwise alignment info from yeast Ilv3 (DEG20010579), cf. Figure S108. . . . S113
S26 Pairwise alignment info from yeast Ilv5 (DEG20010747), cf. Figure S117. . . . S122
S27 Pairwise alignment info from yeast Ilv5 (DEG20010747), cf. Figure S120. . . . S124
S28 Pairwise alignment info from yeast Rib3 (DEG20010261), cf. Figure S129. . . . S133
S29 Pairwise alignment info from yeast Rib3 (DEG20010261), cf. Figure S132. . . . S135
S30 Pairwise alignment info from yeast Rib5 (DEG20010082), cf. Figure S141. . . . S144
S31 Pairwise alignment info from yeast Rib5 (DEG20010082), cf. Figure S144. . . . S146
S32 Pairwise alignment info from yeast Ssy1 (DEG20010185), cf. Figure S153. . . . S155
S33 Pairwise alignment info from yeast Ssy1 (DEG20010185), cf. Figure S156. . . . S157
S34 Pairwise alignment info from yeast Ste12 (DEG20010472), cf. Figure S165. . . . S166
S35 Pairwise alignment info from yeast Ste12 (DEG20010472), cf. Figure S168. . . . S168
S36 Pairwise alignment info from yeast Trl1 (DEG20010555), cf. Figure S176. . . . S177
S37 Pairwise alignment info from yeast Trl1 (DEG20010555), cf. Figure S179. . . . S179
S38 Pairwise alignment info from yeast Yef3 (DEG20010729), cf. Figure S186. . . . S186
S39 Pairwise alignment info from yeast Yef3 (DEG20010729), cf. Figure S189. . . . S188
S40 Pairwise alignment info from yeast Erg11 . . . S197
S41 Pairwise alignment info from yeast Erg24 . . . S198
S42 Pairwise alignment info from yeast Erg2 . . . S199
S43 Pairwise alignment info from yeast Fks1 . . . S201
S44 Pairwise alignment info from yeast Fks3 . . . S202
S45 Pairwise alignment info from yeast Gsc2 . . . S203
S46 Pairwise alignment info from *S. cerevisiae* Ccc1 with all pathogens. . . . S203
S47 Pairwise alignment info from *S. cerevisiae* Ccc1 with all pathogens. . . . S207

List of Figures

S1 Multiple sequence alignment of yeast Alr1 (WHO Critical Pathogens). Cf. Figure S2 for alignment quality, and Figure S3 for Sneath similarity. Cf. Table S6 for protein names, and pairwise alignment metrics with yeast Alr1. S10
S2 Multiple sequence alignment quality of Alr1 (WHO Critical Pathogens). Cf. Figure S1 . . . S11
S3 Sneath Similarity of Alr1 for WHO Critical Pathogens, cf. Figure S1 . . . S12
S4 Multiple sequence alignment of yeast Alr1 (Top 10 Agricultural Fungal Pathogens). Cf. Figure S5 for alignment quality, and Figure S6 for Sneath similarity. Cf. Table S7 for protein names, and pairwise alignment metrics with yeast Alr1. . . . S13
S5 Multiple sequence alignment quality of Alr1 (Top 10 Agricultural Fungal Pathogens). Cf. Figure S4 . . . S13
S6 Sneath Similarity of Alr1 for Top 10 Agricultural Fungal Pathogens, cf. Figure S4 . . . S15
S7 Non-redundant (NR) protein hits for DEG20010935/Alr1, with expectation value of no more than 0.1. Green points are medians, and red points are arithmetic means. . . . S16
S8 Non-redundant (NR) protein hits for Alr1 in the kingdom Metazoa. . . . S17
S9 Non-redundant (NR) protein hits for Alr1 in the kingdom Viridiplantae. . . . S18
S10 Non-redundant (NR) protein hits for Alr1 in the kingdom Fungi. . . . S19
S11 Non-redundant (NR) protein hits for DEG20010935/Alr1 at 20 amino acid length queries. . . . S20
S12 Multiple sequence alignment of yeast Aur1 (WHO Critical Pathogens). Cf. Figure S13 for alignment quality, and Figure S14 for Sneath similarity. Cf. Table S8 for protein names, and pairwise alignment metrics with yeast Aur1. S21

S13 Multiple sequence alignment quality of Aur1 (WHO Critical Pathogens). Cf. Figure S12 . . . S22
S14 Sneath Similarity of Aur1 for WHO Critical Pathogens, cf. Figure S12 . . . S23
S15 Multiple sequence alignment of yeast Aur1 (Top 10 Agricultural Fungal Pathogens). Cf. Figure S16 for alignment quality, and Figure S17 for Sneath similarity. Cf. Table S9 for protein names, and pairwise alignment metrics with yeast Aur1. . . . S24
S16 Multiple sequence alignment quality of Aur1 (Top 10 Agricultural Fungal Pathogens). Cf. Figure S15 . . . S24
S17 Sneath Similarity of Aur1 for Top 10 Agricultural Fungal Pathogens, cf. Figure S15 . . . S26
S18 Non-redundant (NR) protein hits for DEG20010598/Aur1, with expectation value of no more than 0.1. Green points are medians, and red points are arithmetic means. . . . S27
S19 Non-redundant (NR) protein hits for Aur1 in the kingdom Viridiplantae. . . . S28
S20 Non-redundant (NR) protein hits for Aur1 in the kingdom Fungi. . . . S29
S21 Non-redundant (NR) protein hits for Aur1 in the kingdom Metazoa. . . . S30
S22 Non-redundant (NR) protein hits for DEG20010598/Aur1 at 20 amino acid length queries. . . . S31
S23 Multiple sequence alignment of yeast Chs2 (WHO Critical Pathogens). Cf. Figure S24 for alignment quality, and Figure S25 for Sneath similarity. Cf. Table S10 for protein names, and pairwise alignment metrics with yeast Chs2. S32
S24 Multiple sequence alignment quality of Chs2 (WHO Critical Pathogens). Cf. Figure S23 . . . S33
S25 Sneath Similarity of Chs2 for WHO Critical Pathogens, cf. Figure S23 . . . S34
S26 Multiple sequence alignment of yeast Chs2 (Top 10 Agricultural Fungal Pathogens). Cf. Figure S27 for alignment quality, and Figure S28 for Sneath similarity. Cf. Table S11 for protein names, and pairwise alignment metrics with yeast Chs2. . . . S35
S27 Multiple sequence alignment quality of Chs2 (Top 10 Agricultural Fungal Pathogens). Cf. Figure S26 . . . S35
S28 Sneath Similarity of Chs2 for Top 10 Agricultural Fungal Pathogens, cf. Figure S26 . . . S37
S29 Non-redundant (NR) protein hits for DEG20010039/Chs2, with expectation value of no more than 0.1. Green points are medians, and red points are arithmetic means. . . . S38
S30 Non-redundant (NR) protein hits for Chs2 in the kingdom Viridiplantae. . . . S39
S31 Non-redundant (NR) protein hits for Chs2 in the kingdom Fungi. . . . S40
S32 Non-redundant (NR) protein hits for Chs2 in the kingdom SAR. . . . S41
S33 Non-redundant (NR) protein hits for Chs2 in the kingdom Metazoa. . . . S42
S34 Non-redundant (NR) protein hits for DEG20010039/Chs2 at 20 amino acid length queries. . . . S43
S35 Multiple sequence alignment of yeast Erg8 (WHO Critical Pathogens). Cf. Figure S36 for alignment quality, and Figure S37 for Sneath similarity. Cf. Table S12 for protein names, and pairwise alignment metrics with yeast Erg8. S44
S36 Multiple sequence alignment quality of Erg8 (WHO Critical Pathogens). Cf. Figure S35 . . . S44
S37 Sneath Similarity of Erg8 for WHO Critical Pathogens, cf. Figure S35 . . . S45
S38 Multiple sequence alignment of yeast Erg8 (Top 10 Agricultural Fungal Pathogens). Cf. Figure S39 for alignment quality, and Figure S40 for Sneath similarity. Cf. Table S13 for protein names, and pairwise alignment metrics with yeast Erg8. . . . S46
S39 Multiple sequence alignment quality of Erg8 (Top 10 Agricultural Fungal Pathogens). Cf. Figure S38 . . . S46
S40 Sneath Similarity of Erg8 for Top 10 Agricultural Fungal Pathogens, cf. Figure S38 . . . S48
S41 Non-redundant (NR) protein hits for DEG20010822/Erg8, with expectation value of no more than 0.1. Green points are medians, and red points are arithmetic means. . . . S49
S42 Non-redundant (NR) protein hits for Erg8 in the kingdom Metazoa. . . . S50
S43 Non-redundant (NR) protein hits for Erg8 in the kingdom Viridiplantae. . . . S51
S44 Non-redundant (NR) protein hits for Erg8 in the kingdom Fungi. . . . S52
S45 Non-redundant (NR) protein hits for Erg8 in the kingdom SAR. . . . S53
S46 Non-redundant (NR) protein hits for DEG20010822/Erg8 at 20 amino acid length queries. . . . S54
S47 Multiple sequence alignment of yeast Fas1 (WHO Critical Pathogens). Cf. Figure S48 for alignment quality, and Figure S49 for Sneath similarity. Cf. Table S14 for protein names, and pairwise alignment metrics with yeast Fas1. S55
S48 Multiple sequence alignment quality of Fas1 (WHO Critical Pathogens). Cf. Figure S47 . . . S55
S49 Sneath Similarity of Fas1 for WHO Critical Pathogens, cf. Figure S47 . . . S56
S50 Multiple sequence alignment of yeast Fas1 (Top 10 Agricultural Fungal Pathogens). Cf. Figure S51 for alignment quality, and Figure S52 for Sneath similarity. Cf. Table S15 for protein names, and pairwise alignment metrics with yeast Fas1. . . . S57
S51 Multiple sequence alignment quality of Fas1 (Top 10 Agricultural Fungal Pathogens). Cf. Figure S50 . . . S57
S52 Sneath Similarity of Fas1 for Top 10 Agricultural Fungal Pathogens, cf. Figure S50 . . . S59
S53 Non-redundant (NR) protein hits for DEG20010641/Fas1, with expectation value of no more than 0.1. Green points are medians, and red points are arithmetic means. . . . S60
S54 Non-redundant (NR) protein hits for Fas1 in the kingdom Fungi. . . . S61
S55 Non-redundant (NR) protein hits for Fas1 in the kingdom Viridiplantae. . . . S62

S56 Non-redundant (NR) protein hits for Fas1 in the kingdom SAR. . . . S63
S57 Non-redundant (NR) protein hits for DEG20010641/Fas1 at 20 amino acid length queries. . . . S64
S58 Multiple sequence alignment of yeast Fas2 (WHO Critical Pathogens). Cf. Figure S59 for alignment quality, and Figure S60 for Sneath similarity. Cf. Table S16 for protein names, and pairwise alignment metrics with yeast Fas2. S65
S59 Multiple sequence alignment quality of Fas2 (WHO Critical Pathogens). Cf. Figure S58 . . . . S66
S60 Sneath Similarity of Fas2 for WHO Critical Pathogens, cf. Figure S58 . . . . S67
S61 Multiple sequence alignment of yeast Fas2 (Top 10 Agricultural Fungal Pathogens). Cf. Figure S62 for alignment quality, and Figure S63 for Sneath similarity. Cf. Table S17 for protein names, and pairwise alignment metrics with yeast Fas2. . . . . S68
S62 Multiple sequence alignment quality of Fas2 (Top 10 Agricultural Fungal Pathogens). Cf. Figure S61 . . . . S68
S63 Sneath Similarity of Fas2 for Top 10 Agricultural Fungal Pathogens, cf. Figure S61 . . . . S70
S64 Non-redundant (NR) protein hits for DEG20011054/Fas2, with expectation value of no more than 0.1. Green points are medians, and red points are arithmetic means. S71
S65 Non-redundant (NR) protein hits for Fas2 in the kingdom Metazoa. S72
S66 Non-redundant (NR) protein hits for Fas2 in the kingdom SAR. S73
S67 Non-redundant (NR) protein hits for Fas2 in the kingdom Viridiplantae. S74
S68 Non-redundant (NR) protein hits for Fas2 in the kingdom Fungi. S75
S69 Non-redundant (NR) protein hits for DEG20011054/Fas2 at 20 amino acid length queries. S76
S70 Multiple sequence alignment of yeast Fba1 (WHO Critical Pathogens). Cf. Figure S71 for alignment quality, and Figure S72 for Sneath similarity. Cf. Table S18 for protein names, and pairwise alignment metrics with yeast Fba1. S77
S71 Multiple sequence alignment quality of Fba1 (WHO Critical Pathogens). Cf. Figure S70 . . . . S77
S72 Sneath Similarity of Fba1 for WHO Critical Pathogens, cf. Figure S70 . . . . S78
S73 Multiple sequence alignment of yeast Fba1 (Top 10 Agricultural Fungal Pathogens). Cf. Figure S74 for alignment quality, and Figure S75 for Sneath similarity. Cf. Table S19 for protein names, and pairwise alignment metrics with yeast Fba1. . . . . S79
S74 Multiple sequence alignment quality of Fba1 (Top 10 Agricultural Fungal Pathogens). Cf. Figure S73 . . . . S79
S75 Sneath Similarity of Fba1 for Top 10 Agricultural Fungal Pathogens, cf. Figure S73 . . . . S81
S76 Non-redundant (NR) protein hits for DEG20010617/Fba1, with expectation value of no more than 0.1. Green points are medians, and red points are arithmetic means. S82
S77 Non-redundant (NR) protein hits for Fba1 in the kingdom Viridiplantae. S83
S78 Non-redundant (NR) protein hits for Fba1 in the kingdom SAR. S84
S79 Non-redundant (NR) protein hits for Fba1 in the kingdom Metazoa. S85
S80 Non-redundant (NR) protein hits for Fba1 in the kingdom Fungi. S86
S81 Non-redundant (NR) protein hits for DEG20010617/Fba1 at 20 amino acid length queries. S87
S82 Multiple sequence alignment of yeast Fcy21 (WHO Critical Pathogens). Cf. Figure S83 for alignment quality, and Figure S84 for Sneath similarity. Cf. Table S20 for protein names, and pairwise alignment metrics with yeast Fcy21. . . . . S88
S83 Multiple sequence alignment quality of Fcy21 (WHO Critical Pathogens). Cf. Figure S82 . . . . S88
S84 Sneath Similarity of Fcy21 for WHO Critical Pathogens, cf. Figure S82 . . . . S89
S85 Multiple sequence alignment of yeast Fcy21 (Top 10 Agricultural Fungal Pathogens). Cf. Figure S86 for alignment quality, and Figure S87 for Sneath similarity. Cf. Table S21 for protein names, and pairwise alignment metrics with yeast Fcy21. . . . . S90
S86 Multiple sequence alignment quality of Fcy21 (Top 10 Agricultural Fungal Pathogens). Cf. Figure S85 . . . . S90
S87 Sneath Similarity of Fcy21 for Top 10 Agricultural Fungal Pathogens, cf. Figure S85 . . . . S92
S88 Non-redundant (NR) protein hits for DEG20010294/Fcy21, with expectation value of no more than 0.1. Green points are medians, and red points are arithmetic means. S93
S89 Non-redundant (NR) protein hits for Fcy21 in the kingdom Fungi. S94
S90 Non-redundant (NR) protein hits for Fcy21 in the kingdom SAR. S95
S91 Non-redundant (NR) protein hits for Fcy21 in the kingdom Viridiplantae. S96
S92 Non-redundant (NR) protein hits for DEG20010294/Fcy21 at 20 amino acid length queries. S97
S93 Multiple sequence alignment of yeast Fol1 (WHO Critical Pathogens). Cf. Figure S94 for alignment quality, and Figure S95 for Sneath similarity. Cf. Table S22 for protein names, and pairwise alignment metrics with yeast Fol1. S98
S94 Multiple sequence alignment quality of Fol1 (WHO Critical Pathogens). Cf. Figure S93 . . . . S99
S95 Sneath Similarity of Fol1 for WHO Critical Pathogens, cf. Figure S93 . . . . S100
S96 Multiple sequence alignment of yeast Fol1 (Top 10 Agricultural Fungal Pathogens). Cf. Figure S97 for alignment quality, and Figure S98 for Sneath similarity. Cf. Table S23 for protein names, and pairwise alignment metrics with yeast Fol1. . . . . S101
S97 Multiple sequence alignment quality of Fol1 (Top 10 Agricultural Fungal Pathogens). Cf. Figure S96 . . . . S101

S98 Sneath Similarity of Fol1 for Top 10 Agricultural Fungal Pathogens, cf. Figure S96 . . . . . S103
S99 Non-redundant (NR) protein hits for DEG20010889/Fol1, with expectation value of no more than 0.1. Green points are medians, and red points are arithmetic means. S103
S100 Non-redundant (NR) protein hits for Fol1 in the kingdom SAR. S104
S101 Non-redundant (NR) protein hits for Fol1 in the kingdom Viridiplantae. S105
S102 Non-redundant (NR) protein hits for Fol1 in the kingdom Fungi. S106
S103 Non-redundant (NR) protein hits for Fol1 in the kingdom Metazoa. S107
S104 Non-redundant (NR) protein hits for DEG20010889/Fol1 at 20 amino acid length queries. S108
S105 Multiple sequence alignment of yeast Ilv3 (WHO Critical Pathogens). Cf. Figure S106 for alignment quality, and Figure S107 for Sneath similarity. Cf. Table S24 for protein names, and pairwise alignment metrics with yeast Ilv3. S109
S106 Multiple sequence alignment quality of Ilv3 (WHO Critical Pathogens). Cf. Figure S105 . . . . . S110
S107 Sneath Similarity of Ilv3 for WHO Critical Pathogens, cf. Figure S105 . . . . . S111
S108 Multiple sequence alignment of yeast Ilv3 (Top 10 Agricultural Fungal Pathogens). Cf. Figure S109 for alignment quality, and Figure S110 for Sneath similarity. Cf. Table S25 for protein names, and pairwise alignment metrics with yeast Ilv3. . . . . . S112
S109 Multiple sequence alignment quality of Ilv3 (Top 10 Agricultural Fungal Pathogens). Cf. Figure S108 . . . . . S112
S110 Sneath Similarity of Ilv3 for Top 10 Agricultural Fungal Pathogens, cf. Figure S108 . . . . . S114
S111 Non-redundant (NR) protein hits for DEG20010579/Ilv3, with expectation value of no more than 0.1. Green points are medians, and red points are arithmetic means. S115
S112 Non-redundant (NR) protein hits for Ilv3 in the kingdom Metazoa. S116
S113 Non-redundant (NR) protein hits for Ilv3 in the kingdom SAR. S117
S114 Non-redundant (NR) protein hits for Ilv3 in the kingdom Fungi. S118
S115 Non-redundant (NR) protein hits for Ilv3 in the kingdom Viridiplantae. S119
S116 Non-redundant (NR) protein hits for DEG20010579/Ilv3 at 20 amino acid length queries. S120
S117 Multiple sequence alignment of yeast Ilv5 (WHO Critical Pathogens). Cf. Figure S118 for alignment quality, and Figure S119 for Sneath similarity. Cf. Table S26 for protein names, and pairwise alignment metrics with yeast Ilv5. S121
S118 Multiple sequence alignment quality of Ilv5 (WHO Critical Pathogens). Cf. Figure S117 . . . . . S121
S119 Sneath Similarity of Ilv5 for WHO Critical Pathogens, cf. Figure S117 . . . . . S122
S120 Multiple sequence alignment of yeast Ilv5 (Top 10 Agricultural Fungal Pathogens). Cf. Figure S121 for alignment quality, and Figure S122 for Sneath similarity. Cf. Table S27 for protein names, and pairwise alignment metrics with yeast Ilv5. . . . . . S123
S121 Multiple sequence alignment quality of Ilv5 (Top 10 Agricultural Fungal Pathogens). Cf. Figure S120 . . . . . S123
S122 Sneath Similarity of Ilv5 for Top 10 Agricultural Fungal Pathogens, cf. Figure S120 . . . . . S125
S123 Non-redundant (NR) protein hits for DEG20010747/Ilv5, with expectation value of no more than 0.1. Green points are medians, and red points are arithmetic means. S126
S124 Non-redundant (NR) protein hits for Ilv5 in the kingdom Metazoa. S127
S125 Non-redundant (NR) protein hits for Ilv5 in the kingdom SAR. S128
S126 Non-redundant (NR) protein hits for Ilv5 in the kingdom Viridiplantae. S129
S127 Non-redundant (NR) protein hits for Ilv5 in the kingdom Fungi. S130
S128 Non-redundant (NR) protein hits for DEG20010747/Ilv5 at 20 amino acid length queries. S131
S129 Multiple sequence alignment of yeast Rib3 (WHO Critical Pathogens). Cf. Figure S130 for alignment quality, and Figure S131 for Sneath similarity. Cf. Table S28 for protein names, and pairwise alignment metrics with yeast Rib3. . . . . . S132
S130 Multiple sequence alignment quality of Rib3 (WHO Critical Pathogens). Cf. Figure S129 . . . . . S132
S131 Sneath Similarity of Rib3 for WHO Critical Pathogens, cf. Figure S129 . . . . . S133
S132 Multiple sequence alignment of yeast Rib3 (Top 10 Agricultural Fungal Pathogens). Cf. Figure S133 for alignment quality, and Figure S134 for Sneath similarity. Cf. Table S29 for protein names, and pairwise alignment metrics with yeast Rib3. . . . . . S134
S133 Multiple sequence alignment quality of Rib3 (Top 10 Agricultural Fungal Pathogens). Cf. Figure S132 . . . . . S134
S134 Sneath Similarity of Rib3 for Top 10 Agricultural Fungal Pathogens, cf. Figure S132 . . . . . S136
S135 Non-redundant (NR) protein hits for DEG20010261/Rib3, with expectation value of no more than 0.1. Green points are medians, and red points are arithmetic means. S137
S136 Non-redundant (NR) protein hits for Rib3 in the kingdom Viridiplantae. S138
S137 Non-redundant (NR) protein hits for Rib3 in the kingdom SAR. S139
S138 Non-redundant (NR) protein hits for Rib3 in the kingdom Fungi. S140
S139 Non-redundant (NR) protein hits for Rib3 in the kingdom Metazoa. S141
S140 Non-redundant (NR) protein hits for DEG20010261/Rib3 at 20 amino acid length queries. S142

S141 Multiple sequence alignment of yeast Rib5 (WHO Critical Pathogens). Cf. Figure S142 for alignment quality, and Figure S143 for Sneath similarity. Cf. Table S30 for protein names, and pairwise alignment metrics with yeast Rib5. . . . S143
S142 Multiple sequence alignment quality of Rib5 (WHO Critical Pathogens). Cf. Figure S141 . . . S143
S143 Sneath Similarity of Rib5 for WHO Critical Pathogens, cf. Figure S141 . . . S144
S144 Multiple sequence alignment of yeast Rib5 (Top 10 Agricultural Fungal Pathogens). Cf. Figure S145 for alignment quality, and Figure S146 for Sneath similarity. Cf. Table S31 for protein names, and pairwise alignment metrics with yeast Rib5. . . . S145
S145 Multiple sequence alignment quality of Rib5 (Top 10 Agricultural Fungal Pathogens). Cf. Figure S144 . . . S145
S146 Sneath Similarity of Rib5 for Top 10 Agricultural Fungal Pathogens, cf. Figure S144 . . . S147
S147 Non-redundant (NR) protein hits for DEG20010082/Rib5, with expectation value of no more than 0.1. Green points are medians, and red points are arithmetic means. . . . S148
S148 Non-redundant (NR) protein hits for Rib5 in the kingdom Fungi. . . . S149
S149 Non-redundant (NR) protein hits for Rib5 in the kingdom SAR. . . . S150
S150 Non-redundant (NR) protein hits for Rib5 in the kingdom Metazoa. . . . S151
S151 Non-redundant (NR) protein hits for Rib5 in the kingdom Viridiplantae. . . . S152
S152 Non-redundant (NR) protein hits for DEG20010082/Rib5 at 20 amino acid length queries. . . . S153
S153 Multiple sequence alignment of yeast Ssy1 (WHO Critical Pathogens). Cf. Figure S154 for alignment quality, and Figure S155 for Sneath similarity. Cf. Table S32 for protein names, and pairwise alignment metrics with yeast Ssy1. . . . S154
S154 Multiple sequence alignment quality of Ssy1 (WHO Critical Pathogens). Cf. Figure S153 . . . S154
S155 Sneath Similarity of Ssy1 for WHO Critical Pathogens, cf. Figure S153 . . . S155
S156 Multiple sequence alignment of yeast Ssy1 (Top 10 Agricultural Fungal Pathogens). Cf. Figure S157 for alignment quality, and Figure S158 for Sneath similarity. Cf. Table S33 for protein names, and pairwise alignment metrics with yeast Ssy1. . . . S156
S157 Multiple sequence alignment quality of Ssy1 (Top 10 Agricultural Fungal Pathogens). Cf. Figure S156 . . . S156
S158 Sneath Similarity of Ssy1 for Top 10 Agricultural Fungal Pathogens, cf. Figure S156 . . . S158
S159 Non-redundant (NR) protein hits for DEG20010185/Ssy1, with expectation value of no more than 0.1. Green points are medians, and red points are arithmetic means. . . . S159
S160 Non-redundant (NR) protein hits for Ssy1 in the kingdom Fungi. . . . S160
S161 Non-redundant (NR) protein hits for Ssy1 in the kingdom Metazoa. . . . S161
S162 Non-redundant (NR) protein hits for Ssy1 in the kingdom SAR. . . . S162
S163 Non-redundant (NR) protein hits for Ssy1 in the kingdom Viridiplantae. . . . S163
S164 Non-redundant (NR) protein hits for DEG20010185/Ssy1 at 20 amino acid length queries. . . . S164
S165 Multiple sequence alignment of yeast Ste12 (WHO Critical Pathogens). Cf. Figure S166 for alignment quality, and Figure S167 for Sneath similarity. Cf. Table S34 for protein names, and pairwise alignment metrics with yeast Ste12. . . . S165
S166 Multiple sequence alignment quality of Ste12 (WHO Critical Pathogens). Cf. Figure S165 . . . S165
S167 Sneath Similarity of Ste12 for WHO Critical Pathogens, cf. Figure S165 . . . S166
S168 Multiple sequence alignment of yeast Ste12 (Top 10 Agricultural Fungal Pathogens). Cf. Figure S169 for alignment quality, and Figure S170 for Sneath similarity. Cf. Table S35 for protein names, and pairwise alignment metrics with yeast Ste12. . . . S167
S169 Multiple sequence alignment quality of Ste12 (Top 10 Agricultural Fungal Pathogens). Cf. Figure S168 . . . S167
S170 Sneath Similarity of Ste12 for Top 10 Agricultural Fungal Pathogens, cf. Figure S168 . . . S169
S171 Non-redundant (NR) protein hits for DEG20010472/Ste12, with expectation value of no more than 0.1. Green points are medians, and red points are arithmetic means. . . . S170
S172 Non-redundant (NR) protein hits for Ste12 in the kingdom Viridiplantae. . . . S171
S173 Non-redundant (NR) protein hits for Ste12 in the kingdom Metazoa. . . . S172
S174 Non-redundant (NR) protein hits for Ste12 in the kingdom Fungi. . . . S173
S175 Non-redundant (NR) protein hits for DEG20010472/Ste12 at 20 amino acid length queries. . . . S174
S176 Multiple sequence alignment of yeast Trl1 (WHO Critical Pathogens). Cf. Figure S177 for alignment quality, and Figure S178 for Sneath similarity. Cf. Table S36 for protein names, and pairwise alignment metrics with yeast Trl1. . . . S175
S177 Multiple sequence alignment quality of Trl1 (WHO Critical Pathogens). Cf. Figure S176 . . . S176
S178 Sneath Similarity of Trl1 for WHO Critical Pathogens, cf. Figure S176 . . . S177
S179 Multiple sequence alignment of yeast Trl1 (Top 10 Agricultural Fungal Pathogens). Cf. Figure S180 for alignment quality, and Figure S181 for Sneath similarity. Cf. Table S37 for protein names, and pairwise alignment metrics with yeast Trl1. . . . S178

S180 Multiple sequence alignment quality of Trl1 (Top 10 Agricultural Fungal Pathogens). Cf. Figure S179 . . . . . . S178
S181 Sneath Similarity of Trl1 for Top 10 Agricultural Fungal Pathogens, cf. Figure S179 . . . . S180
S182 Non-redundant (NR) protein hits for DEG20010555/Trl1, with expectation value of no more than 0.1. Green points are medians, and red points are arithmetic means. S181
S183 Non-redundant (NR) protein hits for Trl1 in the kingdom Viridiplantae. S182
S184 Non-redundant (NR) protein hits for Trl1 in the kingdom Fungi. S183
S185 Non-redundant (NR) protein hits for DEG20010555/Trl1 at 20 amino acid length queries. S184
S186 Multiple sequence alignment of yeast Yef3 (WHO Critical Pathogens). Cf. Figure S187 for alignment quality, and Figure S188 for Sneath similarity. Cf. Table S38 for protein names, and pairwise alignment metrics with yeast Yef3. . . . . S185
S187 Multiple sequence alignment quality of Yef3 (WHO Critical Pathogens). Cf. Figure S186 . . . . S185
S188 Sneath Similarity of Yef3 for WHO Critical Pathogens, cf. Figure S186 . . . . S186
S189 Multiple sequence alignment of yeast Yef3 (Top 10 Agricultural Fungal Pathogens). Cf. Figure S190 for alignment quality, and Figure S191 for Sneath similarity. Cf. Table S39 for protein names, and pairwise alignment metrics with yeast Yef3. . . . . S187
S190 Multiple sequence alignment quality of Yef3 (Top 10 Agricultural Fungal Pathogens). Cf. Figure S189 . . . . . S187
S191 Sneath Similarity of Yef3 for Top 10 Agricultural Fungal Pathogens, cf. Figure S189 . . . . S189
S192 Non-redundant (NR) protein hits for DEG20010729/Yef3, with expectation value of no more than 0.1. Green points are medians, and red points are arithmetic means. S190
S193 Non-redundant (NR) protein hits for Yef3 in the kingdom Fungi. S191
S194 Non-redundant (NR) protein hits for Yef3 in the kingdom SAR. S192
S195 Non-redundant (NR) protein hits for Yef3 in the kingdom Viridiplantae. S193
S196 Non-redundant (NR) protein hits for Yef3 in the kingdom Metazoa. S194
S197 Non-redundant (NR) protein hits for DEG20010729/Yef3 at 20 amino acid length queries. S195
S198 MSA for Erg11; cf. Table S40 for gene names and exact alignment values. S196
S199 Histogram of Erg11 hits against NR in 20-aa windows S196
S200 MSA for Erg24; cf. Table S41 for gene names and exact alignment values. S197
S201 MSA for Erg2; cf. Table S42 for gene names and exact alignment values. S198
S202 MSA for Fks1; cf. Table S43 for gene names and exact alignment values. S199
S203 Histogram of Fks1 hits against NR in 20-aa windows S200
S204 MSA for Fks3; cf. Table S44 for gene names and exact alignment values. S201
S205 MSA for Gsc2; cf. Table S45 for gene names and exact alignment values. S202
S206 MSA of Ccc1, with all pathogens in the WHO Critical Pathogens group. Cf. Table S46 and Fig. S207. . . . . . S204
S207 2-D MSA of Ccc1, with all pathogens in the group. Cf. Table S46. . . . . S204
S208 MSA of Ccc1 with only *Aspergillus*, with all genes in the group. Cf. Table S46 and Fig. S206. . . . . S205
S209 MSA of Ccc1 with only *Candida*, with all genes in the group. Cf. Table S46 and Fig. S206. . . . . S205
S210 MSA of Ccc1 with only *Cryptococcus*, with all genes in the group. Cf. Table S46 and Fig. S206. . . . . S206
S211 MSA of Ccc1, with all pathogens in the Top 10 Agricultural Fungal Pathogens group. Cf. Table S47 and Fig. S212. . . . . S208
S212 2-D MSA of Ccc1, with all pathogens in the group. Cf. Table S47. . . . . S209

Drug Class	Mechanism	Drawbacks
echinocandins	β-(1,3)-glucan synthesis[1] (Fks1, Fks2/Gsc2, Fks3)	poor bioavailability, IV dosing
azoles	ergosterol synthesis (Erg11)inhibition of CYP-dependent 14α-demethylase	hepatotoxicity, weeks-months treatment
pyrimidine analogs	disrupts RNA & DNA synthesis	ineffective against *Aspergillus*; hepatotoxic
allylamines/thiocarbamates	squalene epoxidase/Erg1 (e.g. Terbinafine)	CYP2D6 drug-drug interactions
morpholines	Erg2/Erg24 in ergosterol biosynthesis: δ-14 reductase and δ-7–δ-8 isomerase	
polyenes	ergosterol in fungal cell membrane	severe renal toxicity

Table S1: Major current human anti-fungal compound classes and their targets, all genes refer to *Saccharomyces cerevisiae*.

	A	C	D	E	F	G	H	I	K	L	M	N	P	Q	R	S	T	V	W	Y
A	1	0.578	0.156	0.159	0.356	0.659	0.32	0.588	0.426	0.643	0.421	0.28	0.553	0.356	0.315	0.477	0.417	0.675	0.224	0.214
C	0.578	1	0.243	0.221	0.289	0.29	0.288	0.348	0.269	0.398	0.612	0.485	0.318	0.482	0.324	0.613	0.485	0.43	0.188	0.223
D	0.156	0.243	1	0.84	0.172	0.015	0.2	0.338	0.248	0.387	0.292	0.637	0.015	0.492	0.236	0.33	0.254	0.279	0.028	0.287
E	0.159	0.221	0.84	1	0.219	0.049	0.201	0.366	0.435	0.333	0.402	0.578	0.003	0.685	0.382	0.312	0.218	0.239	0.086	0.261
F	0.356	0.289	0.172	0.219	1	0.259	0.605	0.487	0.381	0.57	0.465	0.34	0.282	0.459	0.339	0.38	0.254	0.38	0.741	0.729
G	0.659	0.29	0.015	0.049	0.259	1	0.183	0.371	0.309	0.38	0.149	0.19	0.499	0.163	0.149	0.323	0.396	0.437	0.138	0.163
H	0.32	0.288	0.2	0.201	0.605	0.183	1	0.368	0.421	0.45	0.345	0.459	0.172	0.406	0.396	0.342	0.208	0.3	0.484	0.504
I	0.588	0.348	0.338	0.366	0.487	0.371	0.368	1	0.477	0.889	0.494	0.456	0.419	0.453	0.342	0.36	0.493	0.843	0.287	0.266
K	0.426	0.269	0.248	0.435	0.381	0.309	0.421	0.477	1	0.492	0.482	0.401	0.295	0.545	0.733	0.285	0.224	0.419	0.297	0.285
L	0.643	0.398	0.387	0.333	0.57	0.38	0.45	0.889	0.492	1	0.515	0.506	0.432	0.501	0.36	0.411	0.432	0.785	0.368	0.347
M	0.421	0.612	0.292	0.402	0.465	0.149	0.345	0.494	0.482	0.515	1	0.518	0.265	0.699	0.522	0.48	0.409	0.465	0.355	0.307
N	0.28	0.485	0.637	0.578	0.34	0.19	0.459	0.456	0.401	0.506	0.518	1	0.172	0.589	0.427	0.581	0.488	0.395	0.316	0.391
P	0.553	0.318	0.015	0.003	0.282	0.499	0.172	0.419	0.295	0.432	0.265	0.172	1	0.168	0.155	0.321	0.33	0.473	0.211	0.179
Q	0.356	0.482	0.492	0.685	0.459	0.163	0.406	0.453	0.545	0.501	0.699	0.589	0.168	1	0.561	0.501	0.34	0.416	0.353	0.368
R	0.315	0.324	0.236	0.382	0.339	0.149	0.396	0.342	0.733	0.36	0.522	0.427	0.155	0.561	1	0.317	0.258	0.307	0.288	0.407
S	0.477	0.613	0.33	0.312	0.38	0.323	0.342	0.36	0.285	0.411	0.48	0.581	0.321	0.501	0.317	1	0.668	0.439	0.243	0.354
T	0.417	0.485	0.254	0.218	0.254	0.396	0.208	0.493	0.224	0.432	0.409	0.488	0.33	0.34	0.258	0.668	1	0.551	0.176	0.285
V	0.675	0.43	0.279	0.239	0.38	0.437	0.3	0.843	0.419	0.785	0.465	0.395	0.473	0.416	0.307	0.439	0.551	1	0.195	0.199
W	0.224	0.188	0.028	0.086	0.741	0.138	0.484	0.287	0.297	0.368	0.355	0.316	0.211	0.353	0.288	0.243	0.176	0.195	1	0.565
Y	0.214	0.223	0.287	0.261	0.729	0.163	0.504	0.266	0.285	0.347	0.307	0.391	0.179	0.368	0.407	0.354	0.285	0.199	0.565	1

Table S2: Similarity between amino acids can be quantified by Sneath's index ϕ, where identical amino acids have a value of 1, and the greater the index between two amino acids is, the more similar the amino acids are to one another..

S1 Data Sources

**Species**	**Source**
Glycine max	GCF_000004515.6
Homo sapiens	GCF_000001405.40
Oryza sativa	GCF_001433935.1
Solanum tuberosum	GCF_000226075.1
Zea mays	GCF_902167145.1

Table S3: Species and the data sources used for hosts. The Top 10 agricultural pathogens group used every species here, while the WHO pathogens only used *H. sapiens*.

Species	Source
Aspergillus fumigatus	GCF_000002655.1
Candida albicans	GCF_000182965.3
Candida auris	GCF_003013715.1
Candida parapsilosis	GCF_000182765.1
Candida tropicalis	GCF_000006335.3
Cryptococcus.neoformans JEC21	GCF_000091045.1
Cryptococcus.neoformans.B 3501A	GCF_000149385.1
Cryptococcus.neoformans.grubii H99	GCF_000149245.1
Histoplasma capsulatum	GCF_000150115.1
Nakaseomyces glabratus	GCF_000002545.3

Table S4: World Health Organization fungal pathogens and the data sources used.

Species	Source
Blumeria graminis	GCA_905067625.1
Botrytis cinerea	GCF_000143535.2
Colletotrichum truncatum	GCF_014235925.1
Fusarium graminearum	GCF_000240135.3
Mycosphaerella graminicola	GCF_000219625.1
Puccinia graminis	GCF_000149925.1
Puccinia striiformis	GCF_021901695.1
Puccinia triticina	GCF_026914185.1
Pyricularia oryzae	GCF_000002495.2
Ustilago maydis	GCF_000328475.2

Table S5: Top ten agricultural pathogens and the data sources used.

S2 Results: Best Targetable Proteins

S2.1 Alr1

S2.1.1 WHO Critical Pathogens

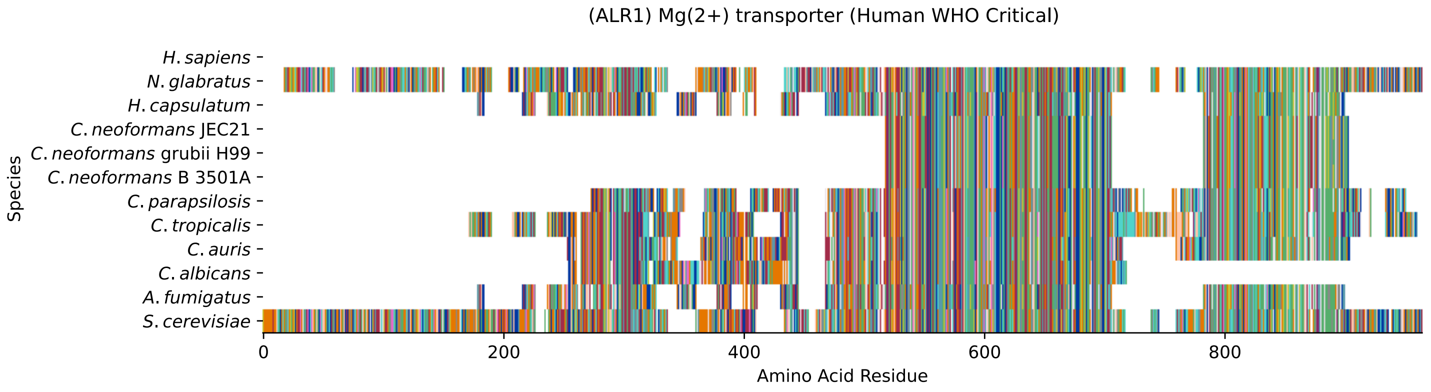

Figure S1: Multiple sequence alignment of yeast Alr1 (WHO Critical Pathogens). Cf. Figure S2 for alignment quality, and Figure S3 for Sneath similarity. Cf. Table S6 for protein names, and pairwise alignment metrics with yeast Alr1.

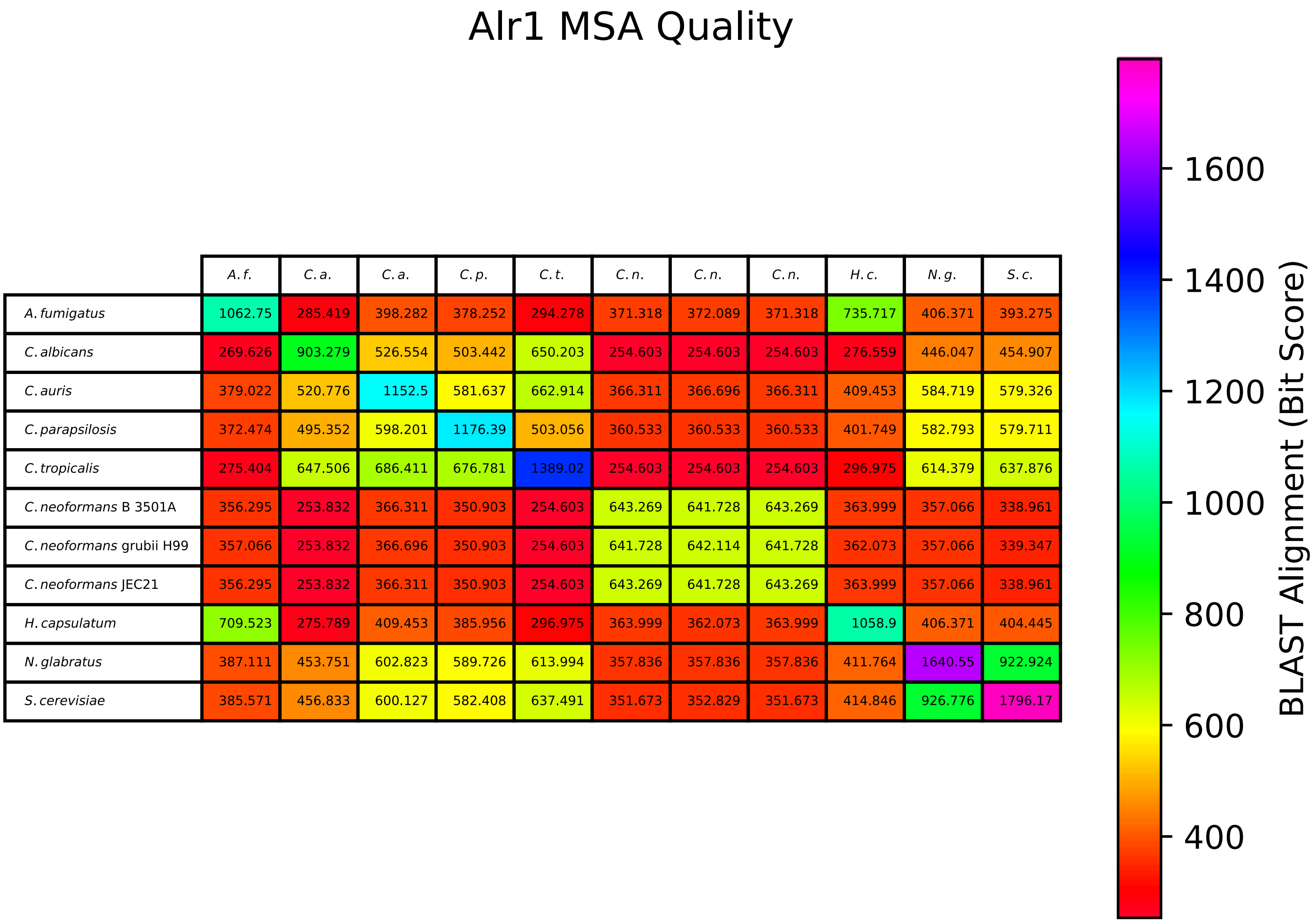

	A. f.	*C. a.*	*C. a.*	*C. p.*	*C. t.*	*C. n.*	*C. n.*	*C. n.*	*H. c.*	*N. g.*	*S. c.*
A. fumigatus	1062.75	285.419	398.282	378.252	294.278	371.318	372.089	371.318	735.717	406.371	393.275
C. albicans	269.626	903.279	526.554	503.442	650.203	254.603	254.603	254.603	276.559	446.047	454.907
C. auris	379.022	520.776	1152.5	581.637	662.914	366.311	366.696	366.311	409.453	584.719	579.326
C. parapsilosis	372.474	495.352	598.201	1176.39	503.056	360.533	360.533	360.533	401.749	582.793	579.711
C. tropicalis	275.404	647.506	686.411	676.781	1389.02	254.603	254.603	254.603	296.975	614.379	637.876
C. neoformans B 3501A	356.295	253.832	366.311	350.903	254.603	643.269	641.728	643.269	363.999	357.066	338.961
C. neoformans grubii H99	357.066	253.832	366.696	350.903	254.603	641.728	642.114	641.728	362.073	357.066	339.347
C. neoformans JEC21	356.295	253.832	366.311	350.903	254.603	643.269	641.728	643.269	363.999	357.066	338.961
H. capsulatum	709.523	275.789	409.453	385.956	296.975	363.999	362.073	363.999	1058.9	406.371	404.445
N. glabratus	387.111	453.751	602.823	589.726	613.994	357.836	357.836	357.836	411.764	1640.55	922.924
S. cerevisiae	385.571	456.833	600.127	582.408	637.491	351.673	352.829	351.673	414.846	926.776	1796.17

Figure S2: Multiple sequence alignment quality of Alr1 (WHO Critical Pathogens). Cf. Figure S1

Species	Hit Protein	Hit Length (a.a.)	evalue	align_len	bit_score	identity	positive	score	gaps	% identity	% positive
H.sapiens	-	-	-	-	-	-	-	-	-	-	-
N.glabratus	XP_445759.1 uncharacterized p-rotein CAGL0E01617g Nakaseomyc-es glabratus	856	0	856	927.161	513	605	2395	85	59.7	70.4
H.capsulatum	XP_045290975.1 magnesium tran-sporter ALR1 Histoplasma capsu-latum G186AR	601	1.6e-135	601	417.157	242	337	1071	98	28.2	39.2
C.neoformans.JEC21	XP_571831.1 hypothetical prot-ein CNG00910 Cryptococcus neof-ormans var. neoformans JEC21	350	1.6e-114	350	366.311	169	234	939	44	19.7	27.2
C.neoformans.grubii.H99	XP_012051300.1 magnesium tran-sporter Cryptococcus neoforman-s var. grubii H99	350	9e-115	350	367.081	169	234	941	44	19.7	27.2
C.neoformans.B.3501A	XP_774407.1 hypothetical prot-ein CNBG3880 Cryptococcus neof-ormans var. neoformans B-3501A	350	1.5e-114	350	366.311	169	234	939	44	19.7	27.2
C.parapsilosis	XP_036663332.1 uncharacterize-d protein CPAR2 101870 Candida parapsilosis	591	0	591	585.874	320	394	1509	48	37.3	45.9
C.tropicalis	XP_002548119.1 magnesium tran-sporter ALR1 Candida tropicali-s MYA-3404	727	0	727	639.032	368	467	1647	120	42.8	54.4
C.auris	XP_028892080.2 hypothetical p-rotein Candida auris	600	0	600	605.905	328	409	1561	91	38.2	47.6
C.albicans	XP_721686.1 Mg(2+) transporte-r Candida albicans SC5314	443	2.2e-149	443	461.455	244	293	1186	49	28.4	34.1
A.fumigatus	XP_754049.1 CorA family metal ion transporter, putative Asp-ergillus fumigatus Af293	601	6.9e-124	601	387.111	241	331	993	94	28.1	38.5

Table S6: Pairwise alignment info from yeast Alr1 (DEG20010935), cf. Figure S1.

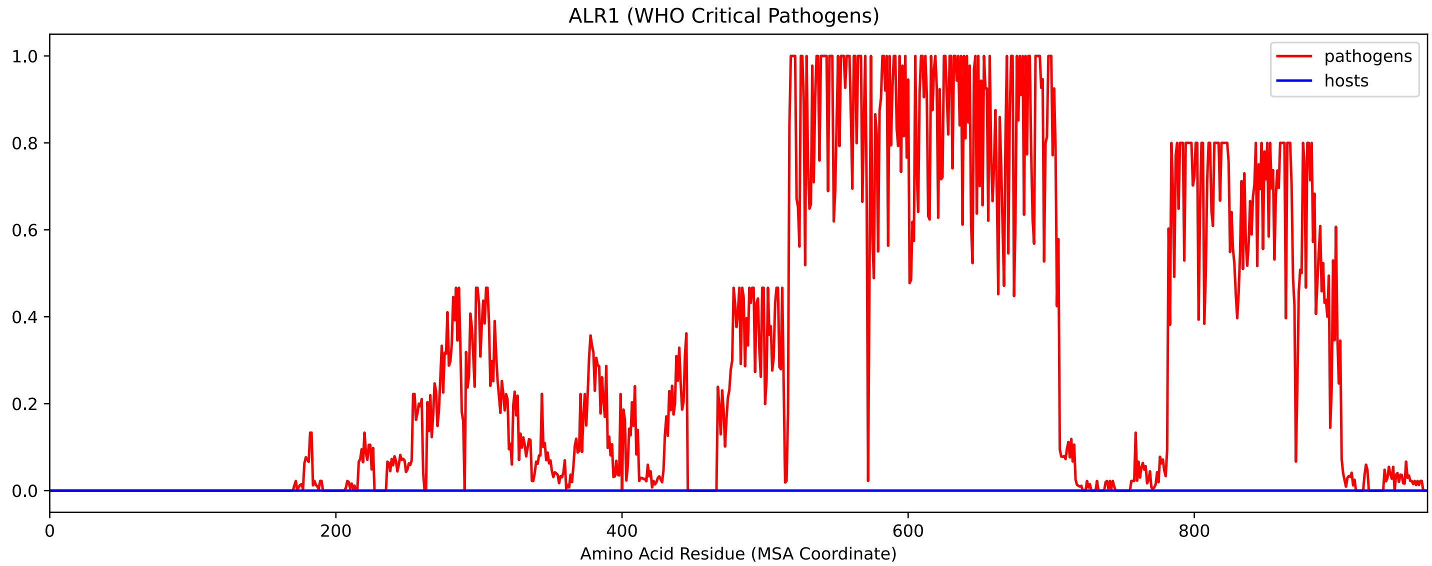

Figure S3: Sneath Similarity of Alr1 for WHO Critical Pathogens, cf. Figure S1

S2.1.2 Top 10 Agricultural Fungal Pathogens

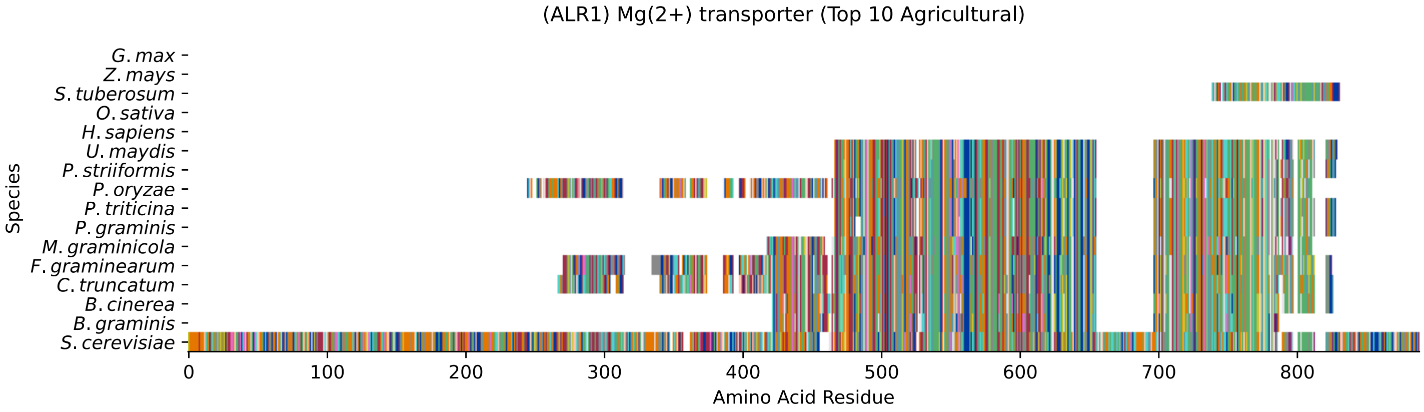

Figure S4: Multiple sequence alignment of yeast Alr1 (Top 10 Agricultural Fungal Pathogens). Cf. Figure S5 for alignment quality, and Figure S6 for Sneath similarity. Cf. Table S7 for protein names, and pairwise alignment metrics with yeast Alr1.

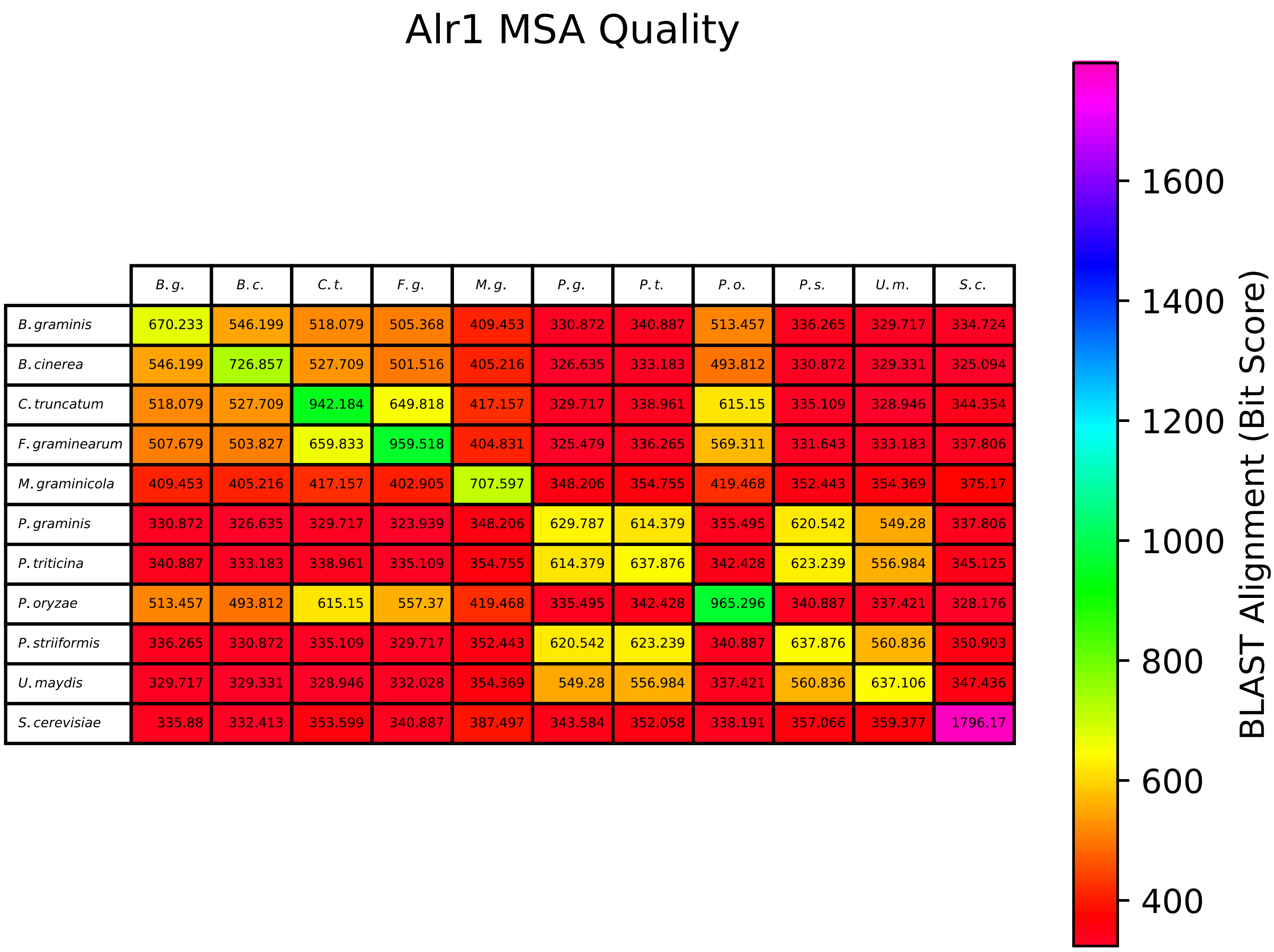

	B. g.	B. c.	C. t.	F. g.	M. g.	P. g.	P. t.	P. o.	P. s.	U. m.	S. c.
B. graminis	670.233	546.199	518.079	505.368	409.453	330.872	340.887	513.457	336.265	329.717	334.724
B. cinerea	546.199	726.857	527.709	501.516	405.216	326.635	333.183	493.812	330.872	329.331	325.094
C. truncatum	518.079	527.709	942.184	649.818	417.157	329.717	338.961	615.15	335.109	328.946	344.354
F. graminearum	507.679	503.827	659.833	959.518	404.831	325.479	336.265	569.311	331.643	333.183	337.806
M. graminicola	409.453	405.216	417.157	402.905	707.597	348.206	354.755	419.468	352.443	354.369	375.17
P. graminis	330.872	326.635	329.717	323.939	348.206	629.787	614.379	335.495	620.542	549.28	337.806
P. triticina	340.887	333.183	338.961	335.109	354.755	614.379	637.876	342.428	623.239	556.984	345.125
P. oryzae	513.457	493.812	615.15	557.37	419.468	335.495	342.428	965.296	340.887	337.421	328.176
P. striiformis	336.265	330.872	335.109	329.717	352.443	620.542	623.239	340.887	637.876	560.836	350.903
U. maydis	329.717	329.331	328.946	332.028	354.369	549.28	556.984	337.421	560.836	637.106	347.436
S. cerevisiae	335.88	332.413	353.599	340.887	387.497	343.584	352.058	338.191	357.066	359.377	1796.17

Figure S5: Multiple sequence alignment quality of Alr1 (Top 10 Agricultural Fungal Pathogens). Cf. Figure S4

Species	Hit Protein	Hit Length (a.a.)	evalue	align_len	bit_score	identity	positive	score	gaps	% identity	% positive
G.max	-	-	-	-	-	-	-	-	-	-	-
Z.mays	-	-	-	-	-	-	-	-	-	-	-
S.tuberosum	XP_006363963.1 PREDICTED: uncharacterized protein LOC102592-202 Solanum tuberosum	93	0.016	93	38.891	31	48	89	15	3.6	5.6
O.sativa	-	-	-	-	-	-	-	-	-	-	-
H.sapiens	-	-	-	-	-	-	-	-	-	-	-
U.maydis	XP_011386252.1 uncharacterized protein UMAG 00361 Ustilago maydis 521	348	9.2e-117	348	374.785	170	237	961	42	19.8	27.6
P.striiformis	XP_047797628.1 hypothetical protein Pst134EA 030450 Puccinia striiformis f. sp. tritici	348	3.5e-115	348	369.777	169	234	948	43	19.7	27.2
P.oryzae	mRNA M BR32 EuGene 00004751-p1 — transcript=mRNA M BR32 EuGene 00004751 — gene=M BR32 EuGene 00004751 — organism=Pyricularia oryzae BR32 — gene product=unspecified product — transcript product=unspecified product — location=BR32 scaffold00001:1398493-1400442(-) — protein length=621 — sequence SO=supercontig — SO=protein coding gene — is pseudo=false	560	1e-106	560	340.887	219	285	873	110	25.5	33.2
P.triticina	XP_053028148.1 uncharacterized protein PtA15 17A74 Puccinia triticina	348	9.9e-115	348	364.385	166	231	934	43	19.3	26.9
P.graminis	XP_003336742.2 hypothetical protein PGTG 17997 Puccinia graminis f. sp. tritici CRL 75-36--700-3	348	2.9e-109	348	359.762	167	229	922	47	19.4	26.7
M.graminicola	ZTRI 8.421.mRNA-p1 — transcript=ZTRI 8.421.mRNA — gene=ZTRI 8.421 — organism=Zymoseptoria tritici IPO323 — gene product=similar to cora family metal ion transporter — transcript product=similar to cora family metal ion transporter — location=Ztri chr 8:1277069-1279072(−) — protein length=667 — sequence SO=chromosome — SO=protein coding gene — is pseudo=false	384	2.1e-127	384	396.741	196	252	1018	49	22.8	29.3
F.graminearum	XP_011318426.1 hypothetical protein FGSG 02495 Fusarium graminearum PH-1	536	3.8e-109	536	347.821	202	286	891	84	23.5	33.3
C.truncatum	XP_036584879.1 CorA-like Mg2+ transporter Colletotrichum truncatum	538	8.3e-113	538	357.451	215	287	916	88	25.0	33.4
B.cinerea	XP_024547928.1 hypothetical protein BCIN 03g07480 Botrytis cinerea B05.10	392	4.5e-107	392	342.813	171	237	878	52	19.9	27.6
B.graminis	VDB86198.1 — transcript=BGT96224V316 LOCUS3808 t1 — gene=BGT96224V316 LOCUS3808 — organism=Blumeria graminis f. sp. tritici 96224 — gene product=unspecified product — transcript product=unspecified product — location=LR026988:17506578-17508517(+) — protein length=630 — sequence SO=chromosome — SO=protein coding gene — is pseudo=false	362	3.8e-107	362	341.658	171	223	875	47	19.9	26.0

Table S7: Pairwise alignment info from yeast Alr1 (DEG20010935), cf. Figure S4.

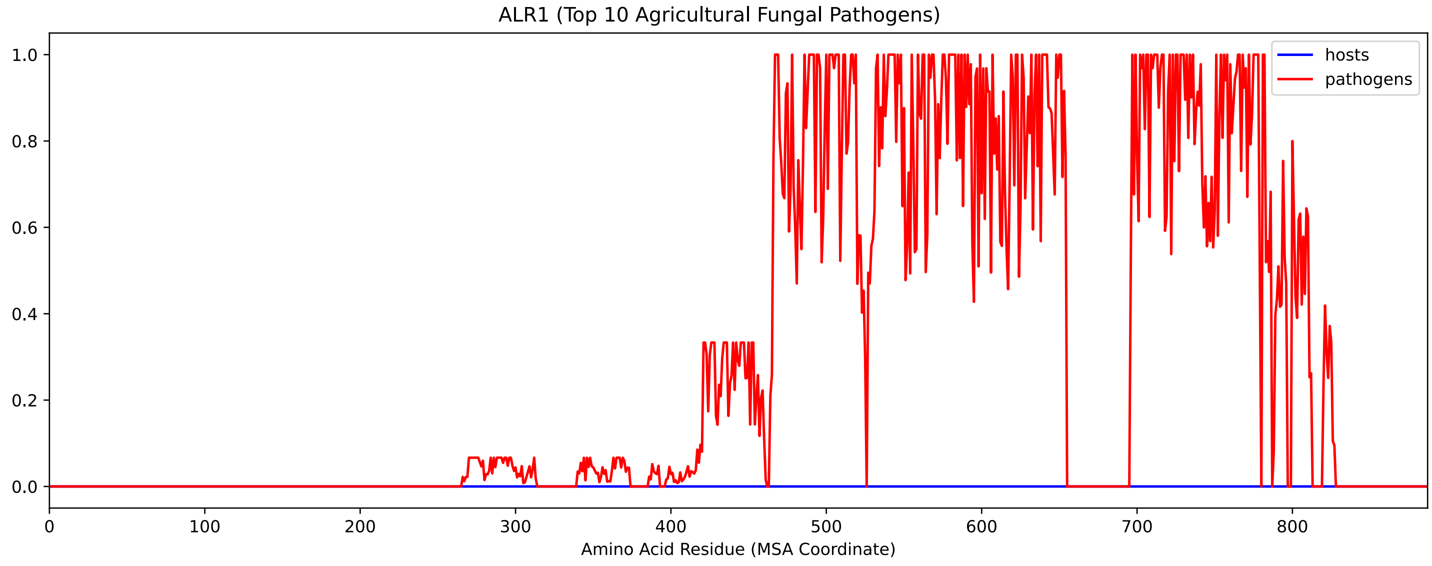

Figure S6: Sneath Similarity of Alr1 for Top 10 Agricultural Fungal Pathogens, cf. Figure S4

S2.1.3 NR

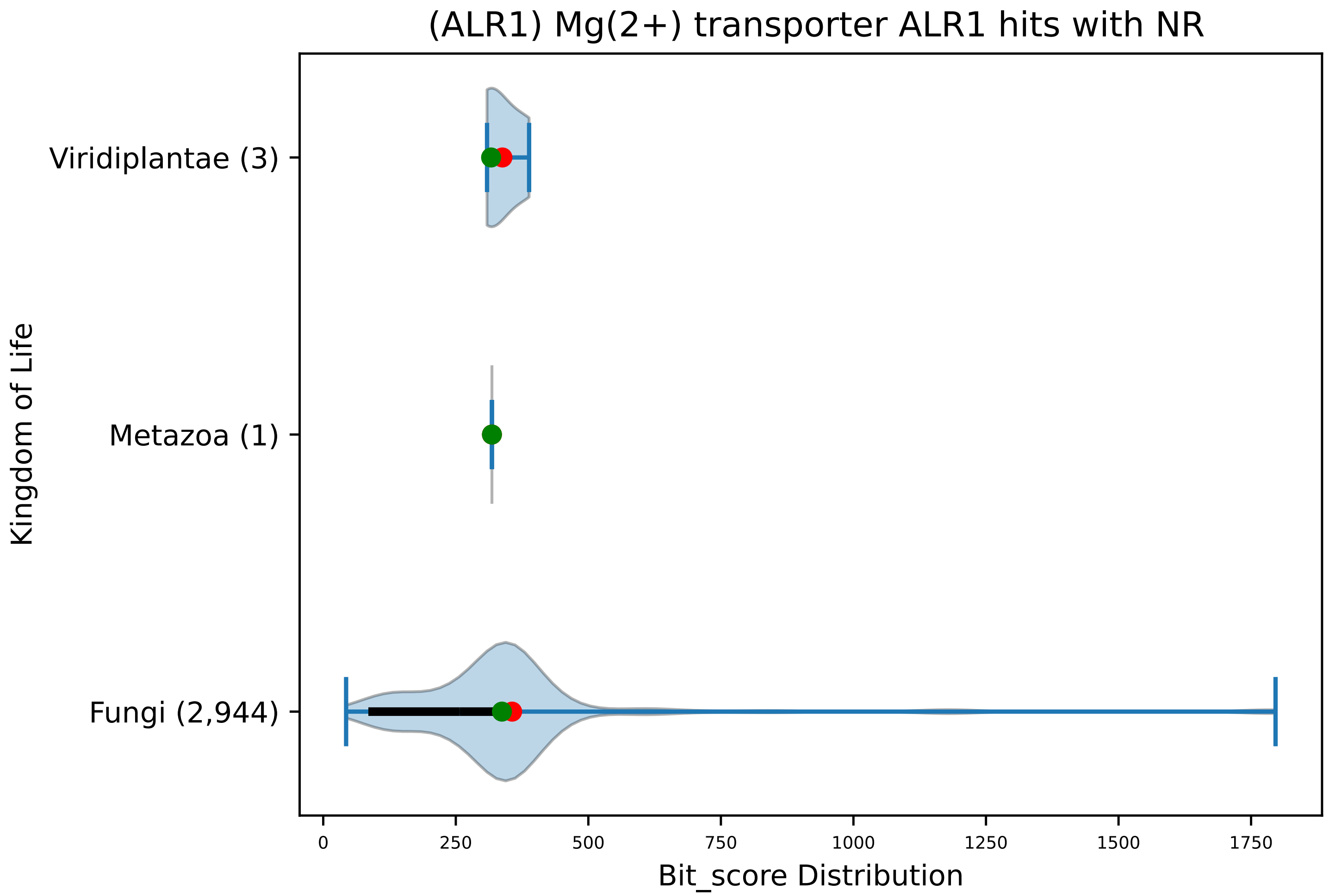

Figure S7: Non-redundant (NR) protein hits for DEG20010935/Alr1, with expectation value of no more than 0.1. Green points are medians, and red points are arithmetic means.

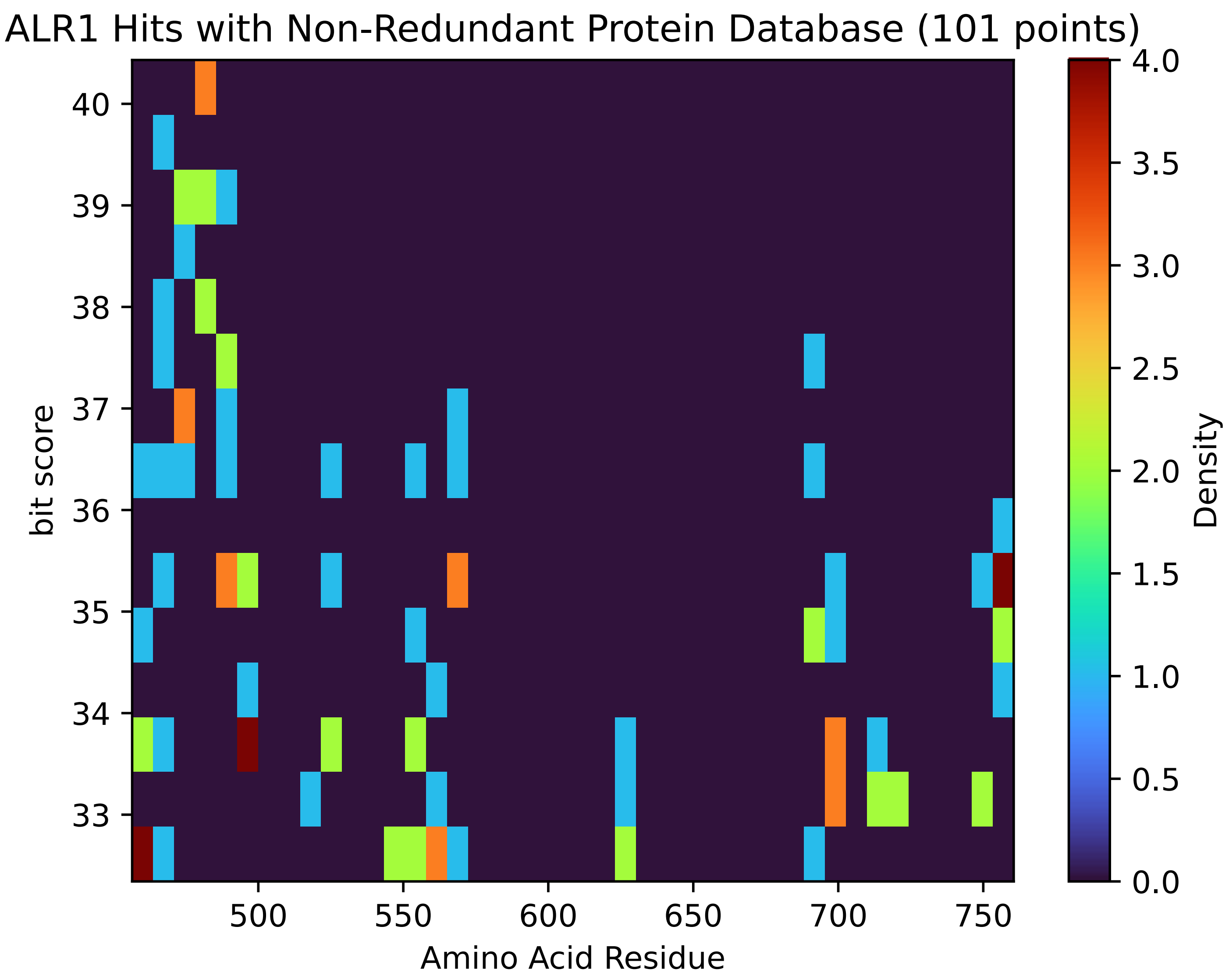

Figure S8: Non-redundant (NR) protein hits for Alr1 in the kingdom Metazoa.

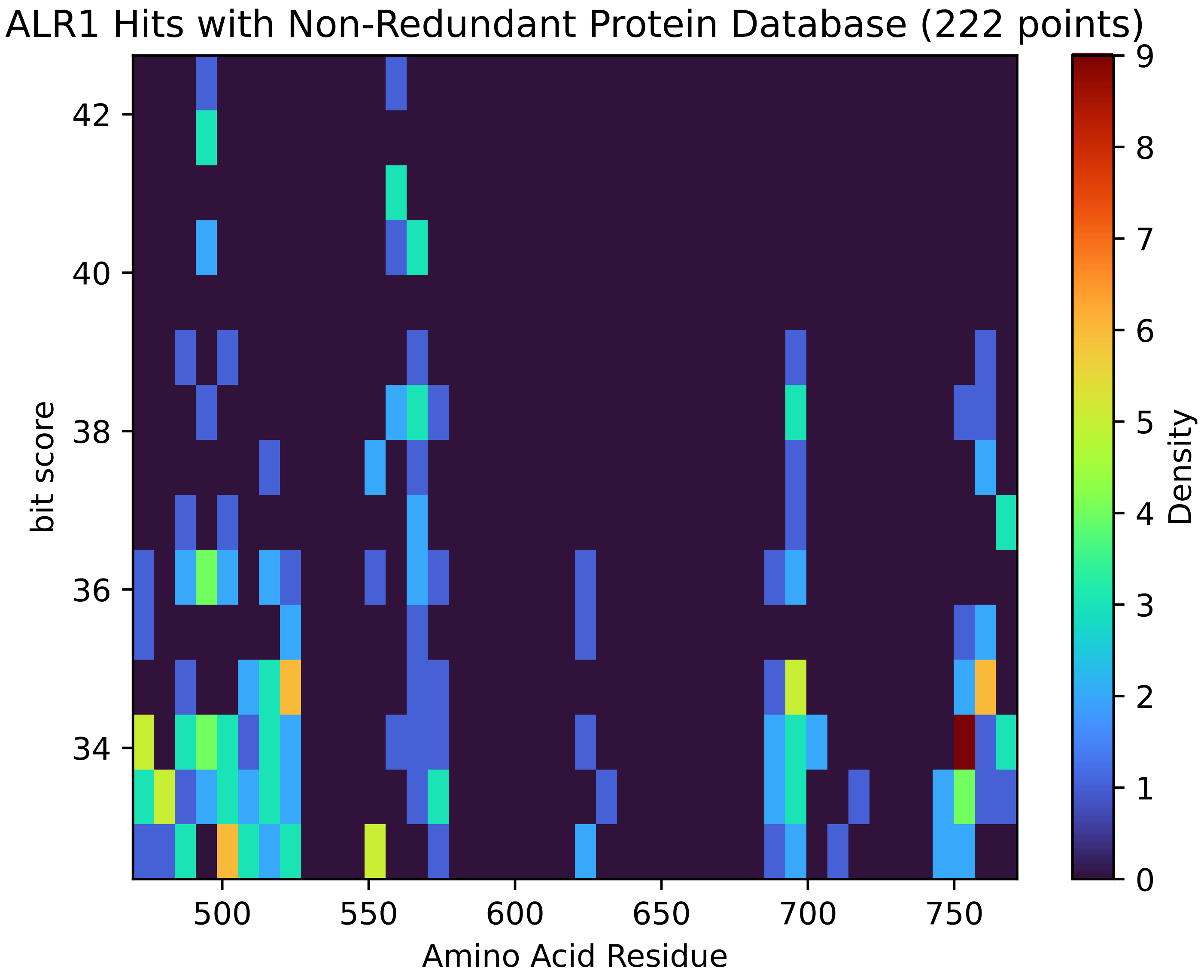

Figure S9: Non-redundant (NR) protein hits for Alr1 in the kingdom Viridiplantae.

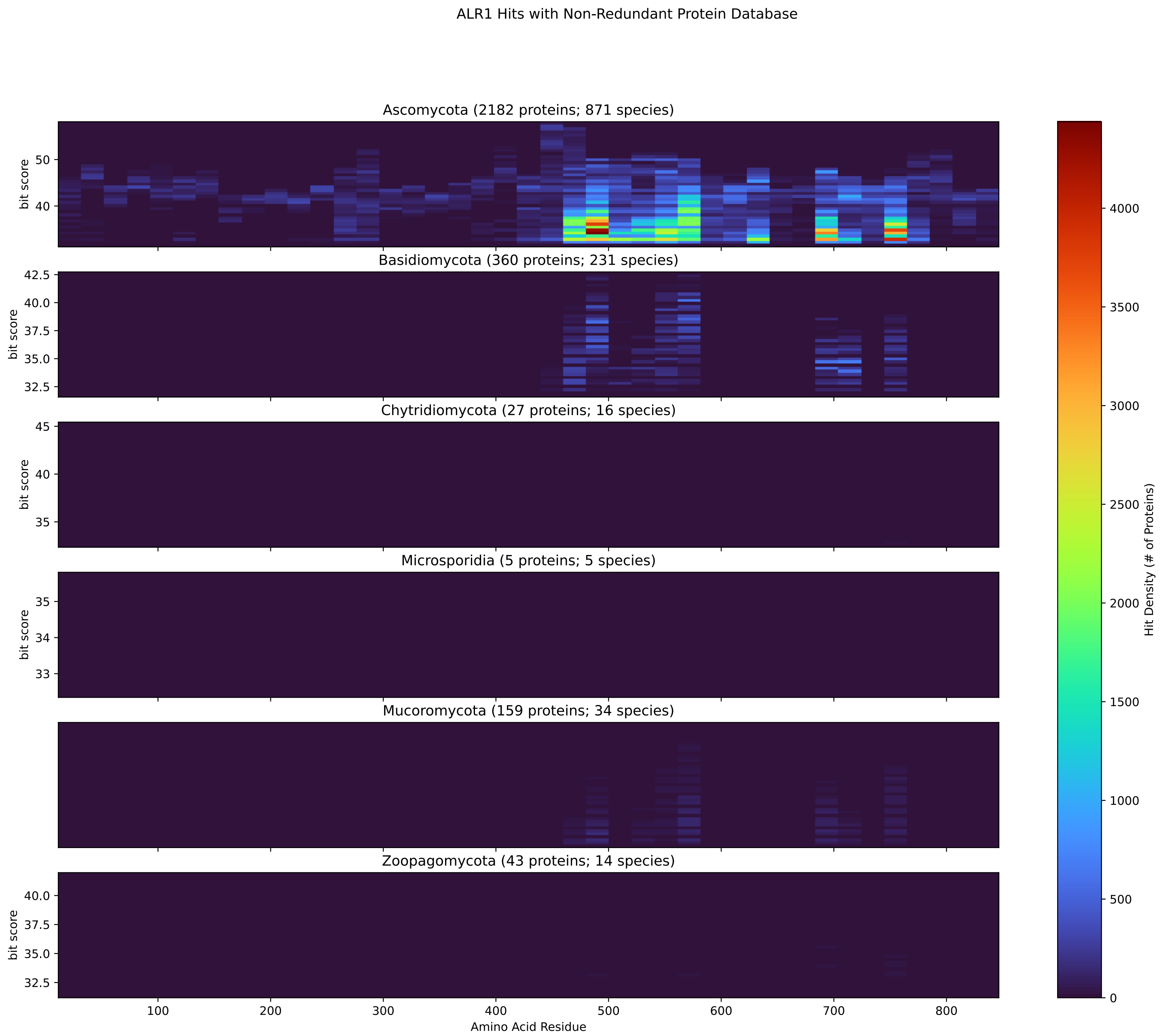

Figure S10: Non-redundant (NR) protein hits for Alr1 in the kingdom Fungi.

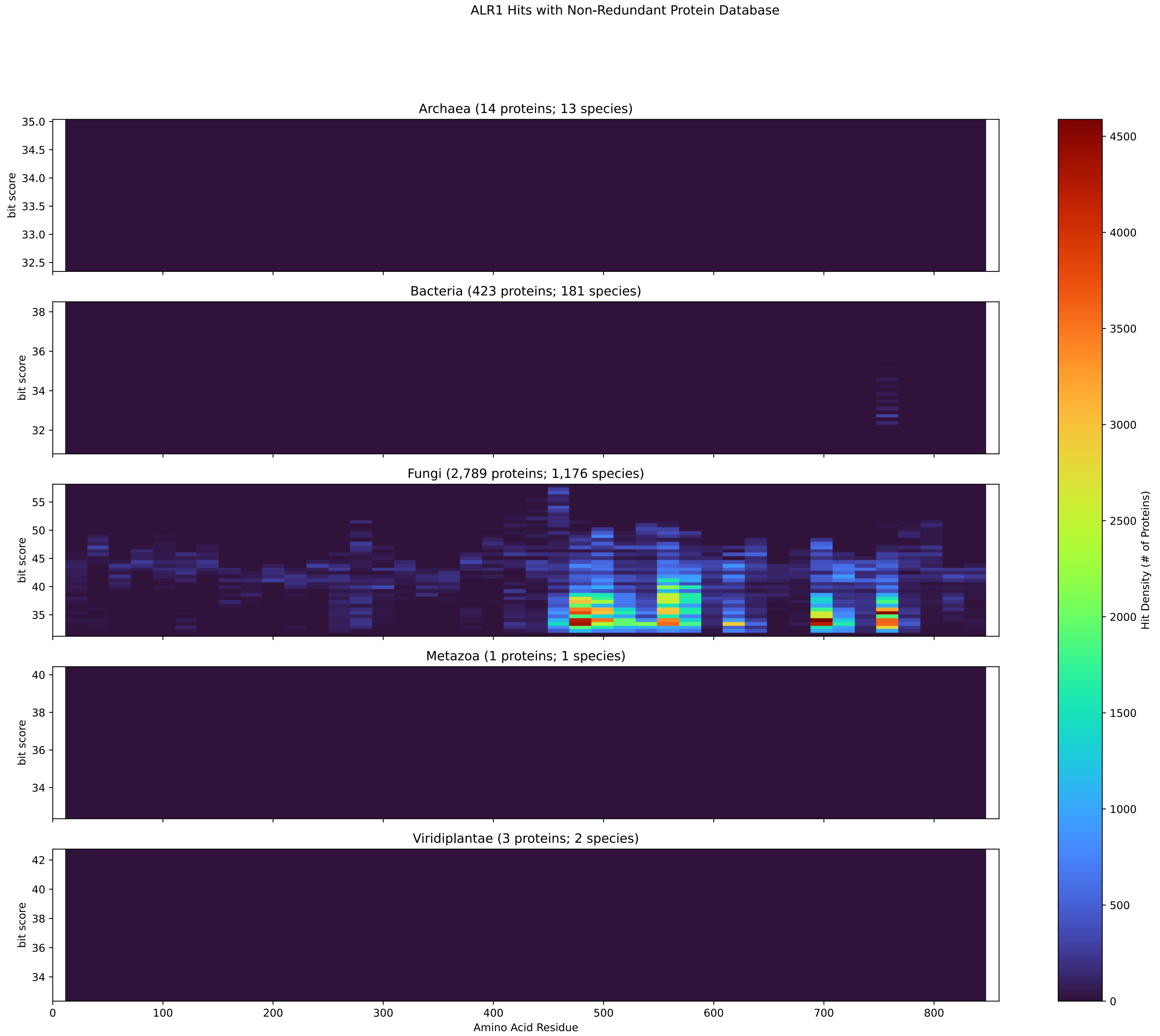

Figure S11: Non-redundant (NR) protein hits for DEG20010935/Alr1 at 20 amino acid length queries.

S2.2 Aur1

S2.2.1 WHO Critical Pathogens

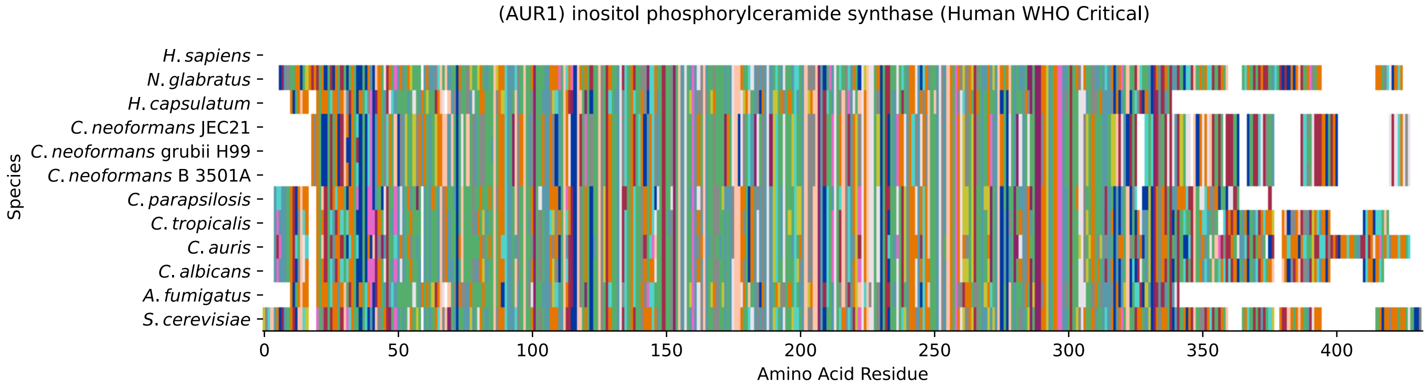

Figure S12: Multiple sequence alignment of yeast Aur1 (WHO Critical Pathogens). Cf. Figure S13 for alignment quality, and Figure S14 for Sneath similarity. Cf. Table S8 for protein names, and pairwise alignment metrics with yeast Aur1.

Aur1 MSA Quality

	A. f.	*C. a.*	*C. a.*	*C. p.*	*C. t.*	*C. n.*	*C. n.*	*C. n.*	*H. c.*	*N. g.*	*S. c.*
A. fumigatus	662.529	313.538	266.929	318.546	325.479	268.47	266.544	268.47	509.22	305.834	296.59
C. albicans	310.457	810.446	451.825	514.227	711.064	285.419	283.493	285.419	307.76	377.867	432.18
C. auris	272.322	466.848	843.958	443.351	465.307	280.411	281.182	280.411	271.937	362.459	390.578
C. parapsilosis	314.694	514.227	428.713	722.62	517.309	267.7	270.396	267.7	303.523	388.267	432.565
C. tropicalis	321.627	711.064	449.129	517.309	810.831	293.893	291.197	293.893	309.686	371.703	434.876
C. neoformans B 3501A	265.003	285.419	271.166	267.7	293.893	760.37	747.273	760.37	258.07	282.337	284.648
C. neoformans grubii H99	263.462	283.493	271.937	270.396	291.197	747.273	758.829	747.273	257.299	283.878	283.108
C. neoformans JEC21	265.003	285.419	271.166	267.7	293.893	760.37	747.273	760.37	258.07	282.337	284.648
H. capsulatum	499.975	307.76	262.692	303.523	309.686	258.07	257.299	258.07	653.284	294.664	296.204
N. glabratus	301.212	377.867	347.821	388.267	371.703	282.337	283.878	282.337	294.664	799.275	515.383
S. cerevisiae	293.123	432.18	376.326	432.565	434.876	284.648	283.108	284.648	296.204	515.383	817.379

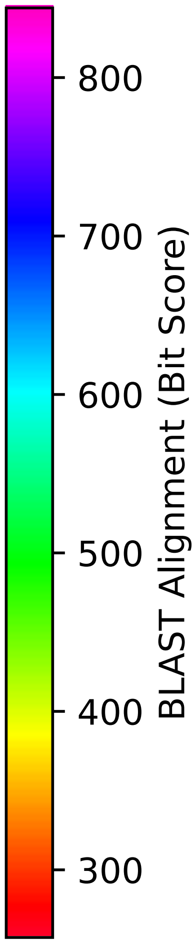

Figure S13: Multiple sequence alignment quality of Aur1 (WHO Critical Pathogens). Cf. Figure S12

Species	Hit Protein	Hit Length (a.a.)	evalue	align_len	bit_score	identity	positive	score	gaps	% identity	% positive
H.sapiens	-	-	-	-	-	-	-	-	-	-	-
N.glabratus	XP_448347.1 uncharacterized p-rotein CAGL0K02805g Nakaseomyc-es glabratus	393	0	393	516.153	252	302	1328	5	62.8	75.3
H.capsulatum	XP_045289424.1 aureobasidin r-esistance protein Aur1 Histopl-asma capsulatum G186AR	326	7.5e-98	326	297.36	148	211	760	2	36.9	52.6
C.neoformans.JEC21	XP_572823.1 inositolphosphory-lceramide synthase, putative C-ryptococcus neoformans var. ne-oformans JEC21	379	4.2e-93	379	285.804	154	225	730	14	38.4	56.1
C.neoformans.grubii.H99	XP_012051809.1 inositolphosph-orylceramide synthase Cryptoco-ccus neoformans var. grubii H9-9	379	1.4e-92	379	284.648	151	224	727	14	37.7	55.9
C.neoformans.B.3501A	XP_774053.1 hypothetical prot-ein CNBH0990 Cryptococcus neof-ormans var. neoformans B-3501A	379	4.1e-93	379	285.804	154	225	730	14	38.4	56.1
C.parapsilosis	XP_036664447.1 uncharacterize-d protein CPAR2 302580 Candida parapsilosis	357	1e-151	357	436.802	216	261	1122	2	53.9	65.1
C.tropicalis	XP_002551078.1 aureobasidin A resistance protein Candida tr-opicalis MYA-3404	397	8.4e-152	397	435.647	215	282	1119	5	53.6	70.3
C.auris	XP_028888388.2 inositol phosp-horylceramide synthase Candida auris	416	7.9e-129	416	376.326	208	271	965	23	51.9	67.6
C.albicans	XP_715708.1 inositol phosphor-ylceramide synthase Candida al-bicans SC5314	399	8.4e-151	399	433.335	219	279	1113	11	54.6	69.6
A.fumigatus	XP_754623.1 aureobasidin resi-stance protein Aur1 Aspergillu-s fumigatus Af293	329	1.3e-96	329	294.278	148	207	752	2	36.9	51.6

Table S8: Pairwise alignment info from yeast Aur1 (DEG20010598), cf. Figure S12.

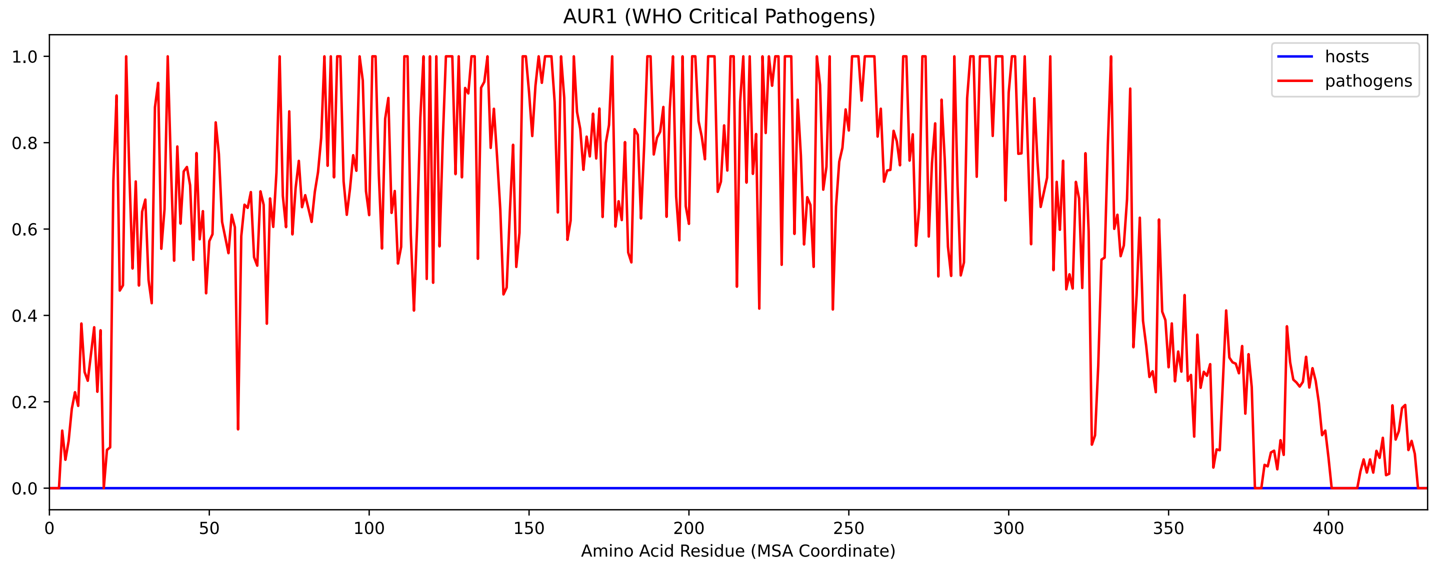

Figure S14: Sneath Similarity of Aur1 for WHO Critical Pathogens, cf. Figure S12

S2.2.2 Top 10 Agricultural Fungal Pathogens

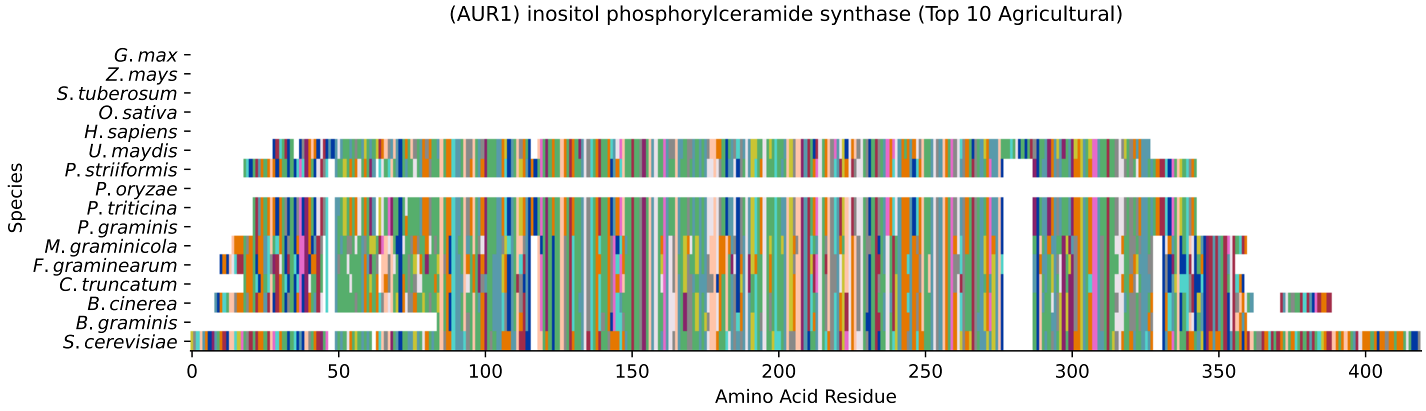

Figure S15: Multiple sequence alignment of yeast Aur1 (Top 10 Agricultural Fungal Pathogens). Cf. Figure S16 for alignment quality, and Figure S17 for Sneath similarity. Cf. Table S9 for protein names, and pairwise alignment metrics with yeast Aur1.

Aur1 MSA Quality

	B. g.	*B. c.*	*C. t.*	*F. g.*	*M. g.*	*P. g.*	*P. t.*	*P. o.*	*P. s.*	*U. m.*	*S. c.*
B. graminis	532.332	456.062	343.969	368.622	402.134	275.404	273.863	0	273.478	251.906	298.13
B. cinerea	457.603	717.998	397.512	409.838	472.241	308.531	307.375	0	306.22	278.485	322.398
C. truncatum	352.829	404.831	656.751	444.121	384.415	288.5	285.419	0	288.886	247.284	303.908
F. graminearum	368.622	402.134	422.935	671.389	379.407	280.796	280.026	0	275.404	244.588	285.804
M. graminicola	402.134	461.84	372.089	379.407	665.611	277.715	277.33	0	280.411	251.521	314.309
P. graminis	275.404	301.597	277.715	280.796	277.715	620.157	613.224	0	578.941	303.138	269.626
P. triticina	273.863	300.056	274.248	280.026	277.33	613.224	620.157	0	578.17	300.827	267.314
P. oryzae	0	0	0	0	0	0	0	0	0	0	0
P. striiformis	273.478	298.516	275.404	275.404	280.411	578.941	578.17	0	627.476	301.212	273.478
U. maydis	270.396	291.967	257.684	264.618	271.937	326.635	323.939	0	324.324	593.964	245.743
S. cerevisiae	298.13	319.316	291.582	285.804	314.309	269.626	267.314	0	273.478	220.705	817.379

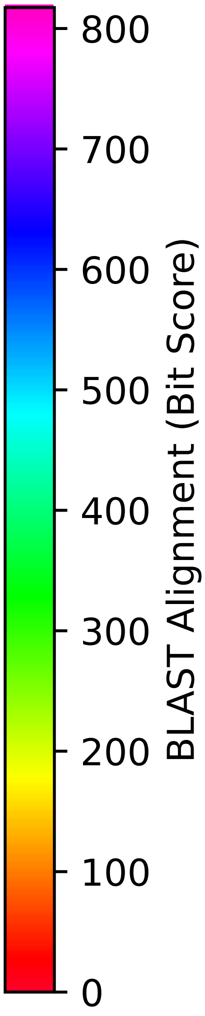

Figure S16: Multiple sequence alignment quality of Aur1 (Top 10 Agricultural Fungal Pathogens). Cf. Figure S15

Species	Hit Protein	Hit Length (a.a.)	evalue	align_len	bit_score	identity	positive	score	gaps	% identity	% positive
G.max	-	-	-	-	-	-	-	-	-	-	-
Z.mays	-	-	-	-	-	-	-	-	-	-	-
S.tuberosum	-	-	-	-	-	-	-	-	-	-	-
O.sativa	-	-	-	-	-	-	-	-	-	-	-
H.sapiens	-	-	-	-	-	-	-	-	-	-	-
U.maydis	XP_011387609.1 uncharacterized protein UMAG 01613 Ustilago maydis 521	295	2.2e-69	295	225.713	133	182	574	11	33.2	45.4
P.striiformis	XP_047811613.1 hypothetical protein Pst134EA 002784 Puccinia striiformis f. sp. tritici	312	1.6e-89	312	275.404	144	196	703	5	35.9	48.9
P.oryzae	-	-	-	-	-	-	-	-	-	-	-
P.triticina	XP_053018037.1 uncharacterized protein PtA15 2A799 Puccinia triticina	309	1.1e-87	309	271.552	143	193	693	5	35.7	48.1
P.graminis	XP_003333402.2 hypothetical protein PGTG 15186 Puccinia graminis f. sp. tritici CRL 75-36--700-3	309	4.6e-88	309	273.092	144	194	697	5	35.9	48.4
M.graminicola	ZTRI 6.447.mRNA-p1 — transcript=ZTRI 6.447.mRNA — gene=ZTRI 6.447 — organism=Zymoseptoria tritici IPO323 — gene product=-similar to aureobasidin resistance protein aur1 — transcript product=similar to aureobasidin resistance protein aur1 — location=Ztri chr 6:1662238-1663581(+) — protein length=447 — sequence SO=chromosome — SO=protein coding gene — is pseudo=false	330	2.1e-105	330	317.39	163	219	812	4	40.6	54.6
F.graminearum	XP_011315988.1 hypothetical protein FGSG 00338 Fusarium graminearum PH-1	331	7.1e-94	331	288.115	149	211	736	7	37.2	52.6
C.truncatum	XP_036579559.1 PAP2 superfamily protein Colletotrichum truncatum	323	3.8e-96	323	294.278	162	210	752	1	40.4	52.4
B.cinerea	XP_001546315.2 Bcaur1 Botrytis cinerea B05.10	364	2.2e-106	364	320.087	166	223	819	11	41.4	55.6
B.graminis	VDB89011.1 — transcript=BGT96224V316 LOCUS4784 t1 — gene=BGT-96224V316 LOCUS4784 — organism=Blumeria graminis f. sp. tritici 96224 — gene product=unspecified product — transcript product=unspecified product — location=LR026990:2935260-2936323(-) — protein length=337 — sequence SO=chromosome — SO=protein coding gene — is pseudo=false	261	3.1e-100	261	299.671	143	182	766	0	35.7	45.4

Table S9: Pairwise alignment info from yeast Aur1 (DEG20010598), cf. Figure S15.

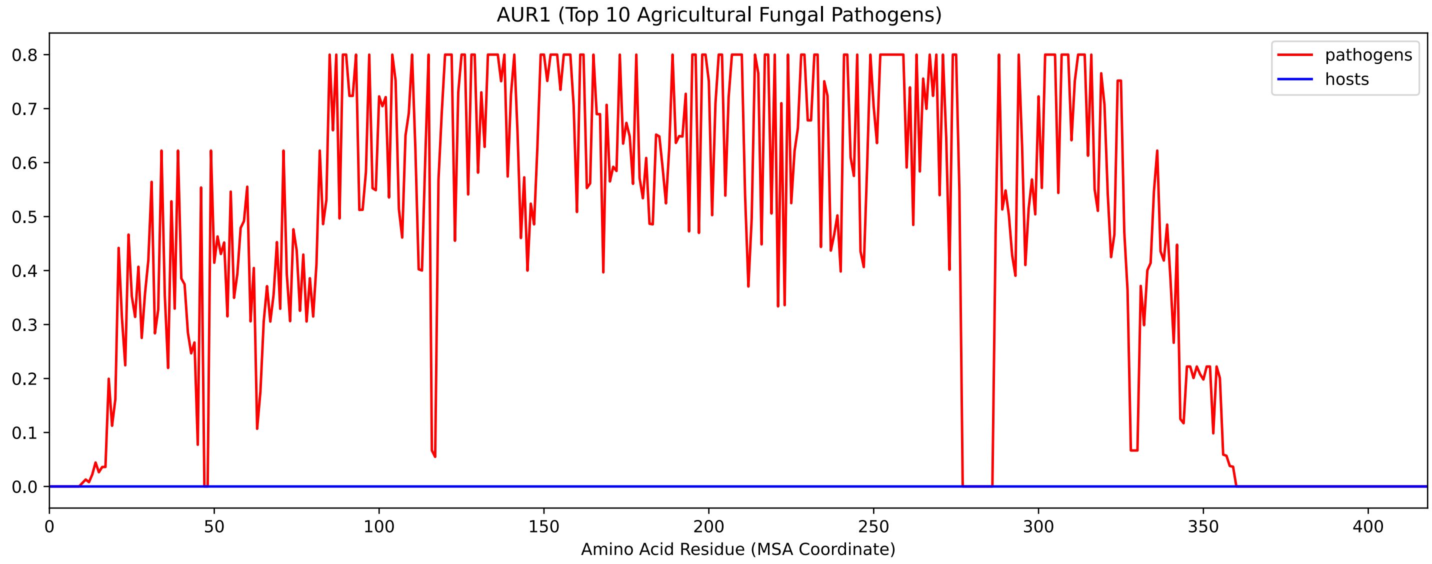

Figure S17: Sneath Similarity of Aur1 for Top 10 Agricultural Fungal Pathogens, cf. Figure S15

S2.2.3 NR

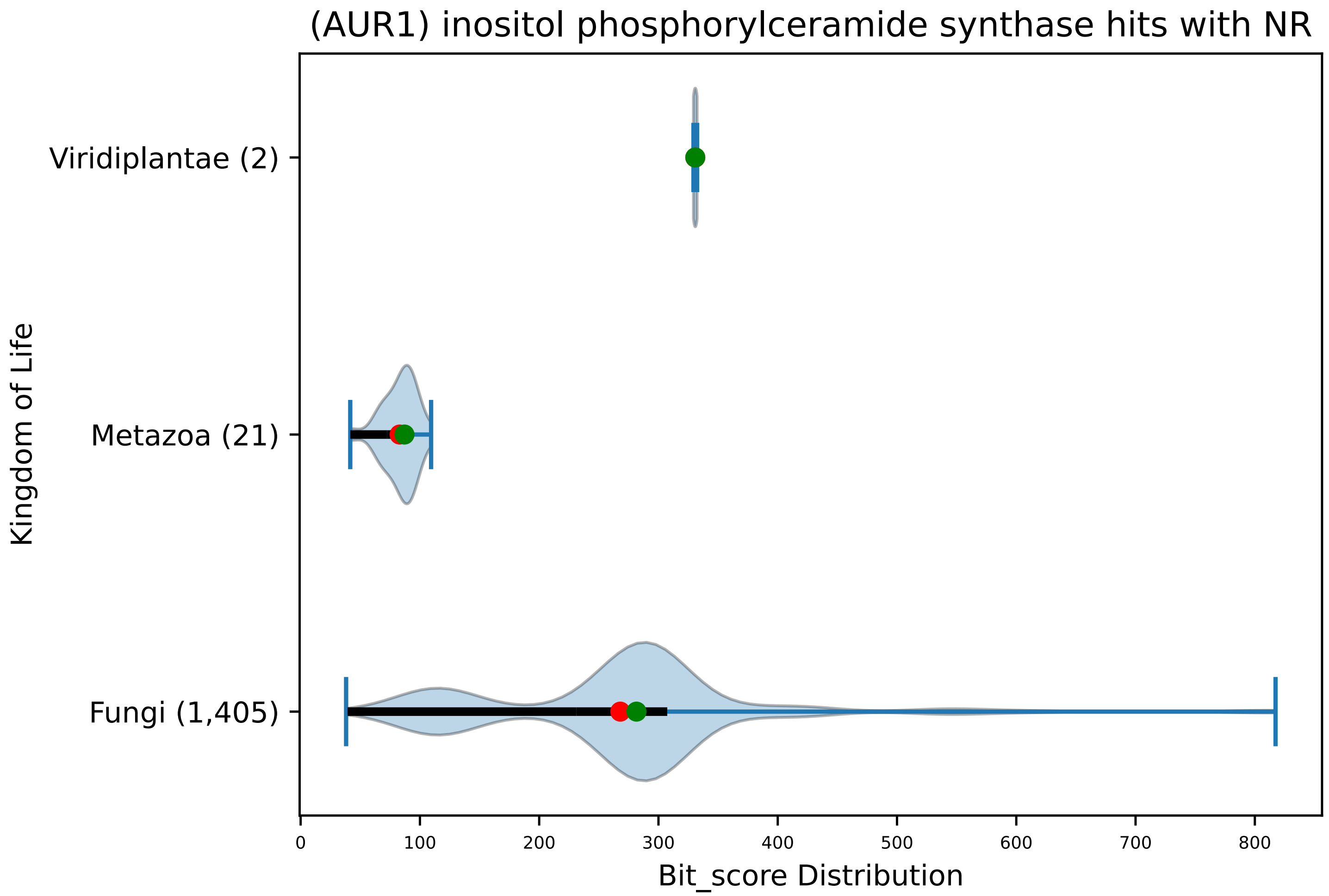

Figure S18: Non-redundant (NR) protein hits for DEG20010598/Aur1, with expectation value of no more than 0.1. Green points are medians, and red points are arithmetic means.

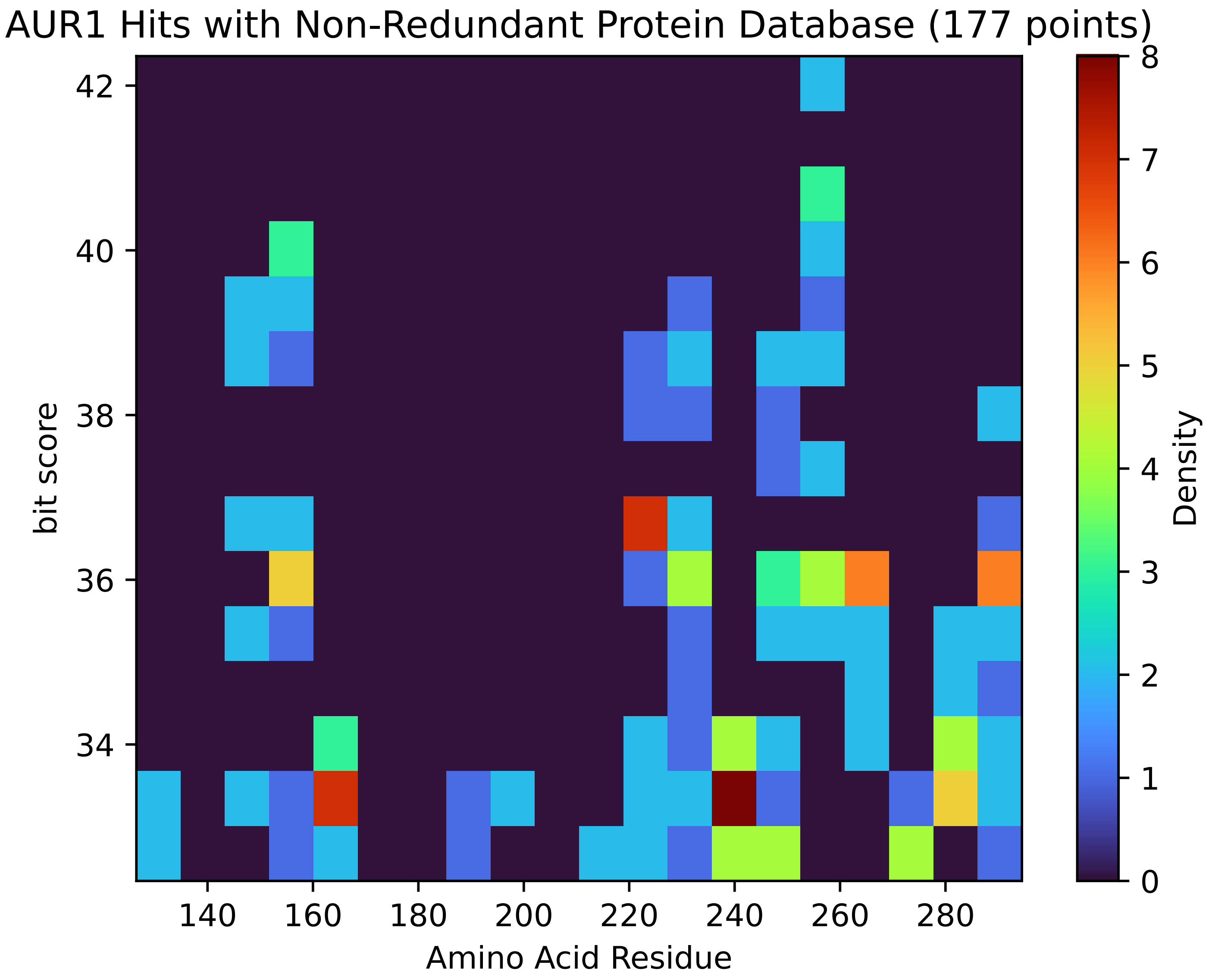

Figure S19: Non-redundant (NR) protein hits for Aur1 in the kingdom Viridiplantae.

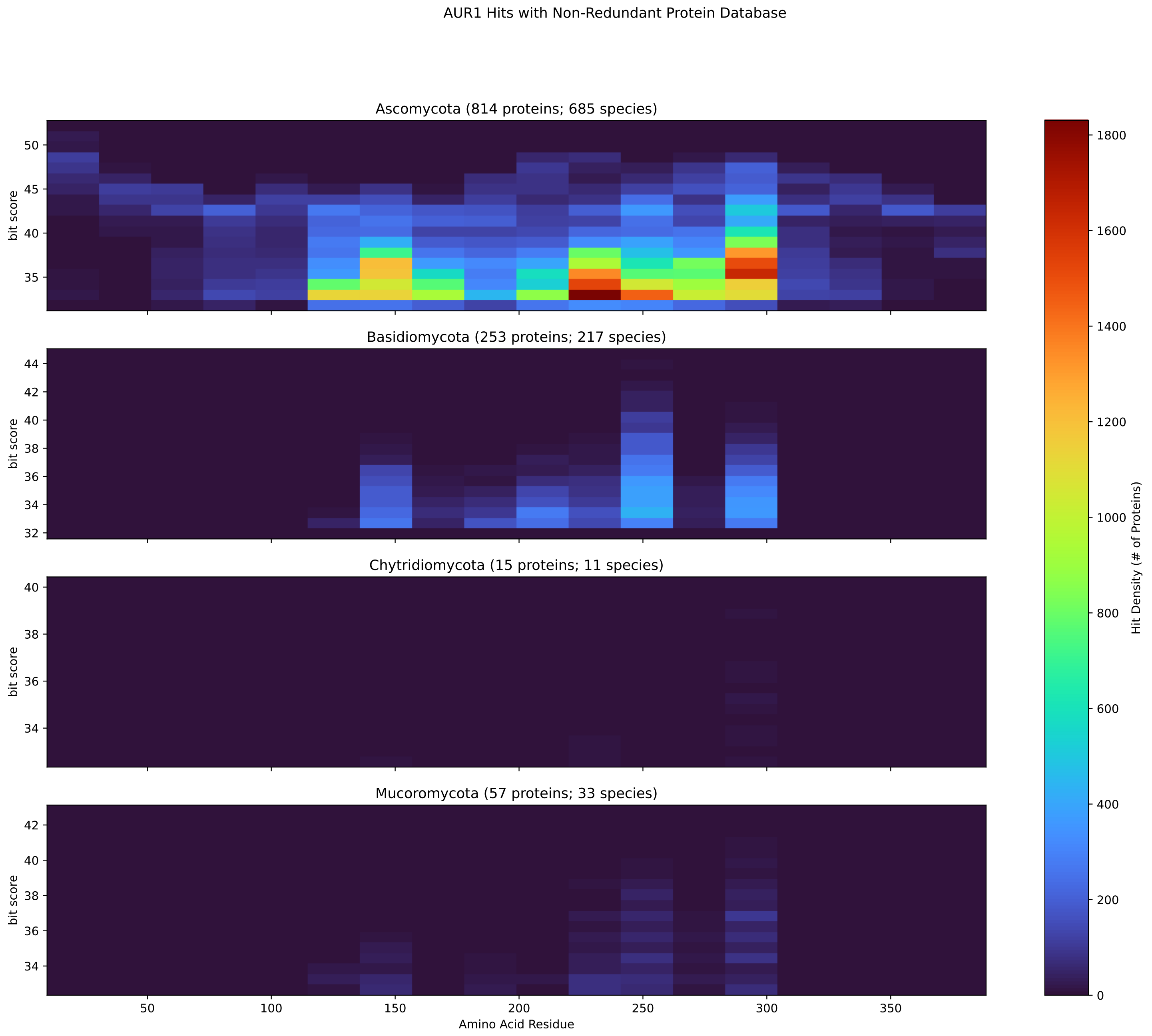

Figure S20: Non-redundant (NR) protein hits for Aur1 in the kingdom Fungi.

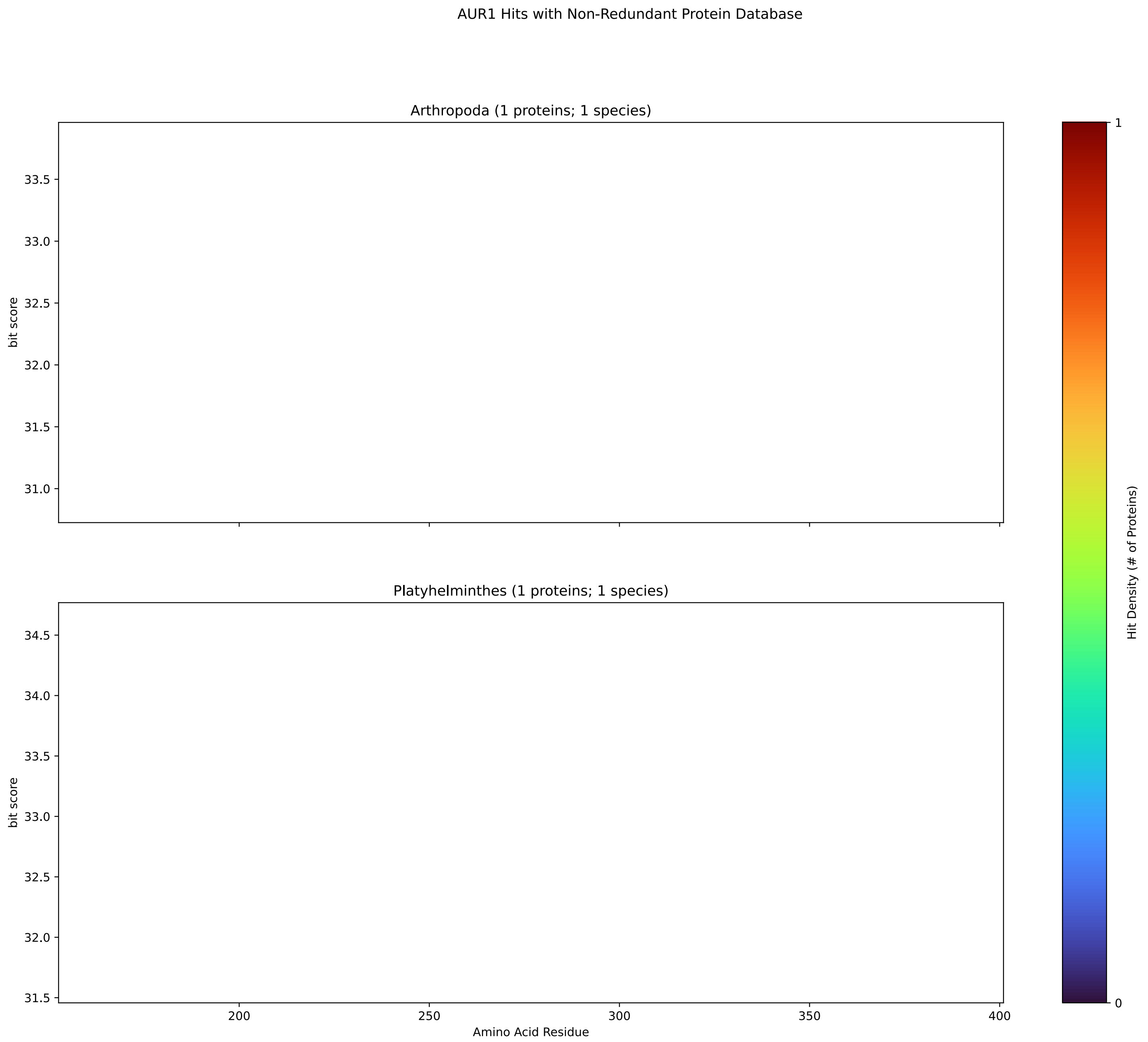

Figure S21: Non-redundant (NR) protein hits for Aur1 in the kingdom Metazoa.

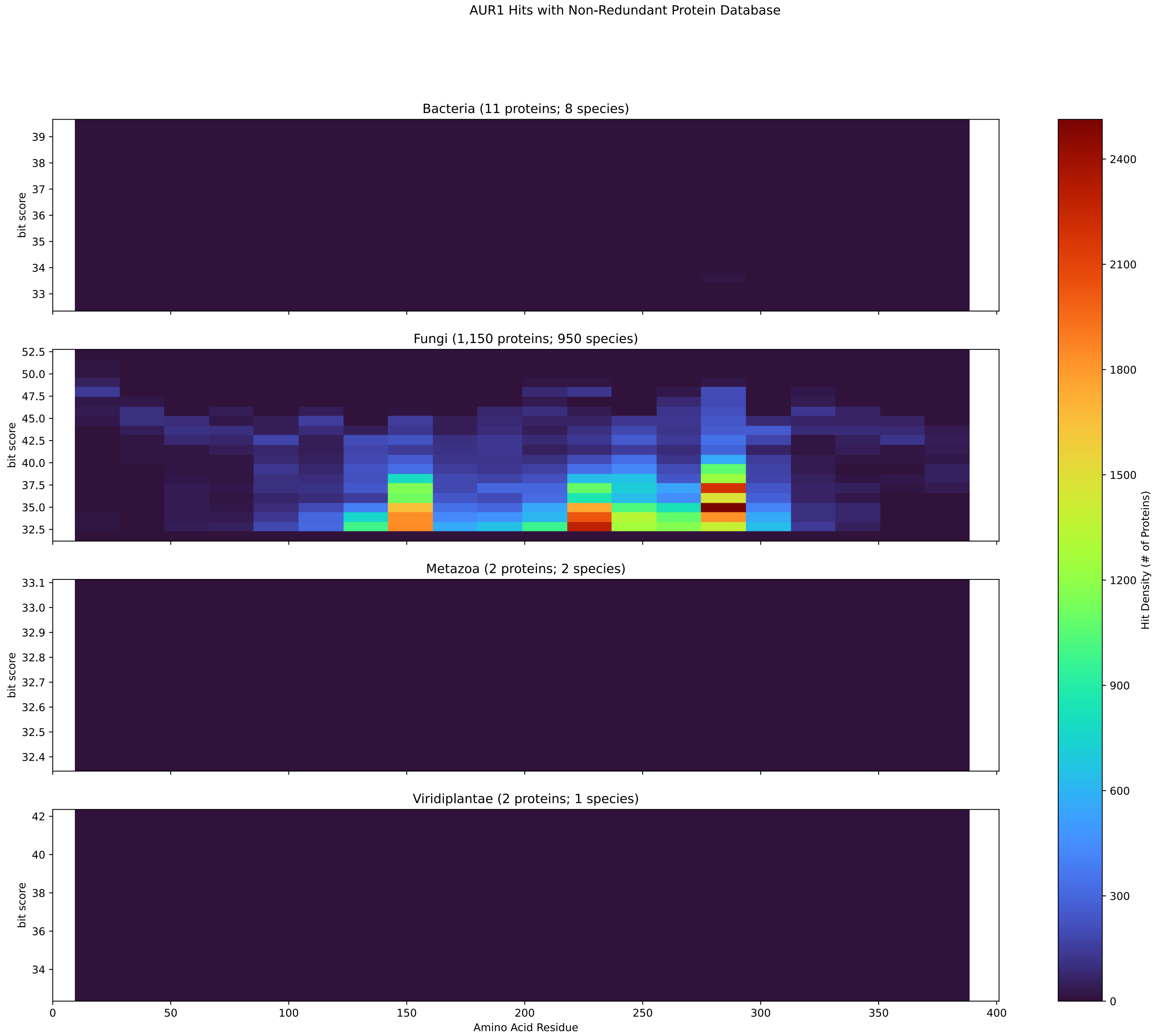

Figure S22: Non-redundant (NR) protein hits for DEG20010598/Aur1 at 20 amino acid length queries.

S2.3 Chs2

S2.3.1 WHO Critical Pathogens

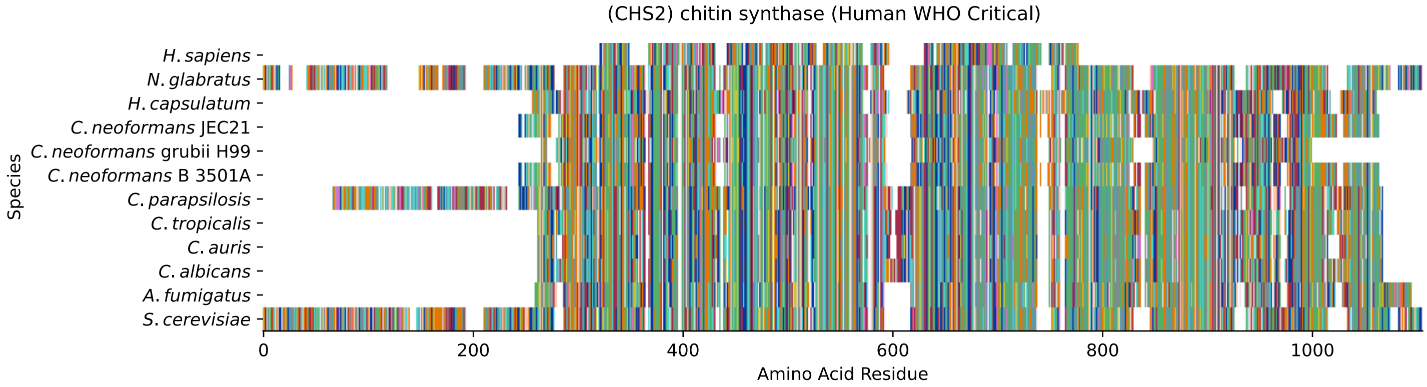

Figure S23: Multiple sequence alignment of yeast Chs2 (WHO Critical Pathogens). Cf. Figure S24 for alignment quality, and Figure S25 for Sneath similarity. Cf. Table S10 for protein names, and pairwise alignment metrics with yeast Chs2.

Chs2 MSA Quality

	A. f.	*C. a.*	*C. a.*	*C. p.*	*C. t.*	*C. n.*	*C. n.*	*C. n.*	*H. c.*	*N. g.*	*S. c.*
A. fumigatus	1571.99	717.998	690.263	827.395	712.99	632.484	583.948	632.484	616.305	629.402	647.892
C. albicans	717.998	1579.69	1051.58	849.736	1358.97	555.444	497.664	555.444	546.584	570.466	579.711
C. auris	690.263	1065.45	1541.17	801.971	1056.2	543.888	474.937	543.888	510.375	559.681	568.54
C. parapsilosis	832.787	857.825	787.334	1932.15	845.884	592.038	518.079	592.038	560.836	577.015	593.193
C. tropicalis	711.835	1378.62	1052.74	851.662	1556.96	551.592	486.108	551.592	539.65	563.533	588.186
C. neoformans B 3501A	637.491	561.607	548.895	588.571	555.829	1536.55	560.066	1536.55	529.25	569.696	598.971
C. neoformans grubii H99	583.948	497.664	475.322	517.694	486.878	550.821	1353.96	550.821	472.241	560.836	546.584
C. neoformans JEC21	637.491	561.607	548.895	588.571	555.829	1536.55	560.066	1536.55	529.25	569.696	598.971
H. capsulatum	616.305	546.584	508.064	556.599	538.495	523.857	472.241	523.857	1570.06	527.709	554.673
N. glabratus	629.402	570.466	558.525	575.859	562.762	565.074	560.836	565.074	527.709	1917.89	1487.63
S. cerevisiae	647.892	579.711	568.54	588.186	583.178	595.504	546.584	595.504	554.673	1487.63	2013.81

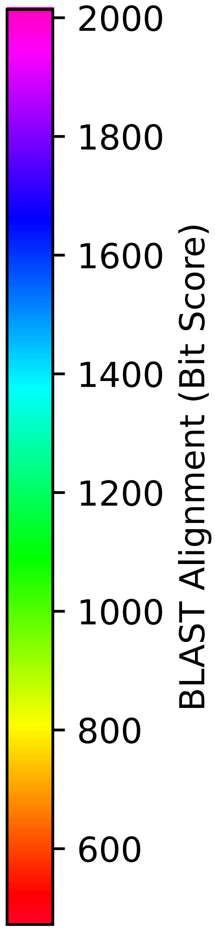

Figure S24: Multiple sequence alignment quality of Chs2 (WHO Critical Pathogens). Cf. Figure S23

Species	Hit Protein	Hit Length (a.a.)	evalue	align_len	bit_score	identity	positive	score	gaps	% identity	% positive
H.sapiens	NP_001186209.1 hyaluronan synthase 3 isoform a Homo sapiens	422	1.9e-05	422	51.2174	95	165	121	68	9.9	17.1
N.glabratus	XP_447459.1 uncharacterized protein CAGL0I04818g Nakaseomyces glabratus	964	0	964	1487.24	734	806	3849	53	76.2	83.7
H.capsulatum	XP_045283732.1 chitin synthase Histoplasma capsulatum G186AR	761	0	761	557.755	310	441	1436	73	32.2	45.8
C.neoformans.JEC21	XP_571995.1 chitin synthase 1-, putative Cryptococcus neoformans var. neoformans JEC21	744	0	744	598.586	310	450	1542	38	32.2	46.7
C.neoformans.grubii.H99	XP_012053352.1 chitin synthase Cryptococcus neoformans var. grubii H99	655	0	655	551.206	294	396	1419	20	30.5	41.1
C.neoformans.B.3501A	XP_774244.1 hypothetical protein CNBG2250 Cryptococcus neoformans var. neoformans B-3501A	744	0	744	598.586	310	450	1542	38	32.2	46.7
C.parapsilosis	XP_036668304.1 uncharacterized protein CPAR2 701490 Candida parapsilosis	958	0	958	589.726	357	501	1519	122	37.1	52.0
C.tropicalis	XP_002551423.1 chitin synthase 2 Candida tropicalis MYA-340-4	759	0	759	588.186	315	452	1515	68	32.7	46.9
C.auris	XP_028890166.1 hypothetical protein Candida auris	747	0	747	572.392	309	445	1474	57	32.1	46.2
C.albicans	XP_717760.2 Chs8p Candida albicans SC5314	767	0	767	583.948	319	456	1504	77	33.1	47.4
A.fumigatus	XP_749322.1 chitin synthase A Aspergillus fumigatus Af293	761	0	761	649.818	343	472	1675	51	35.6	49.0

Table S10: Pairwise alignment info from yeast Chs2 (DEG20010039), cf. Figure S23.

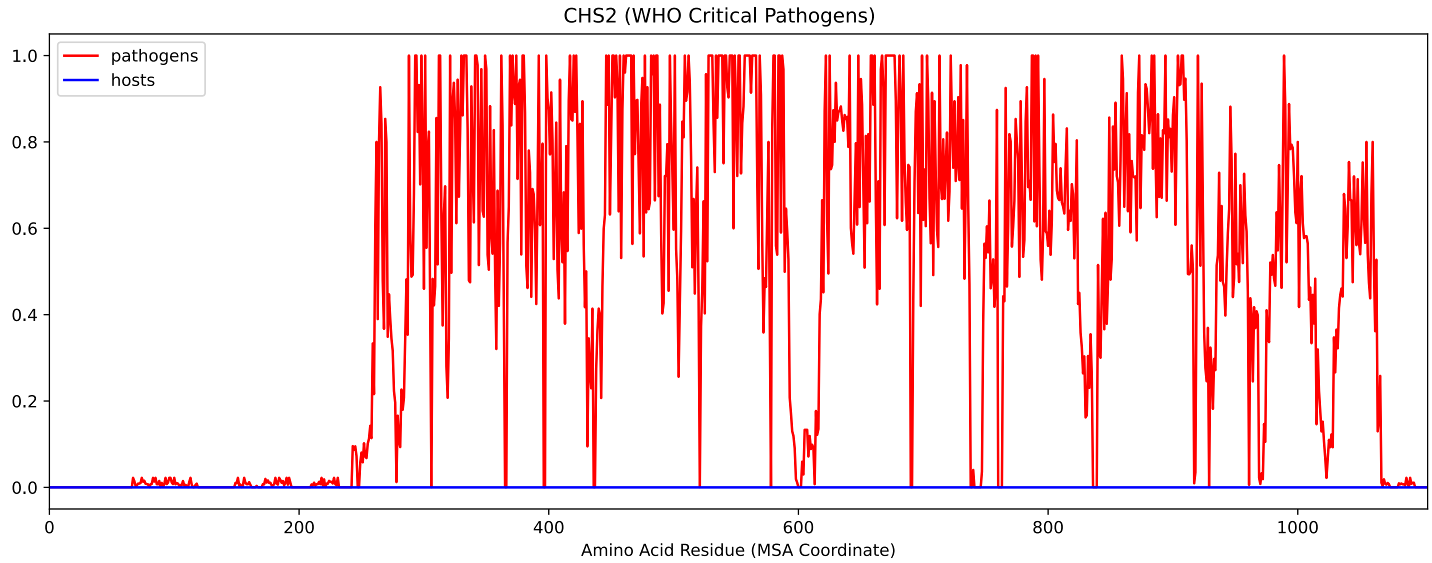

Figure S25: Sneath Similarity of Chs2 for WHO Critical Pathogens, cf. Figure S23

S2.3.2 Top 10 Agricultural Fungal Pathogens

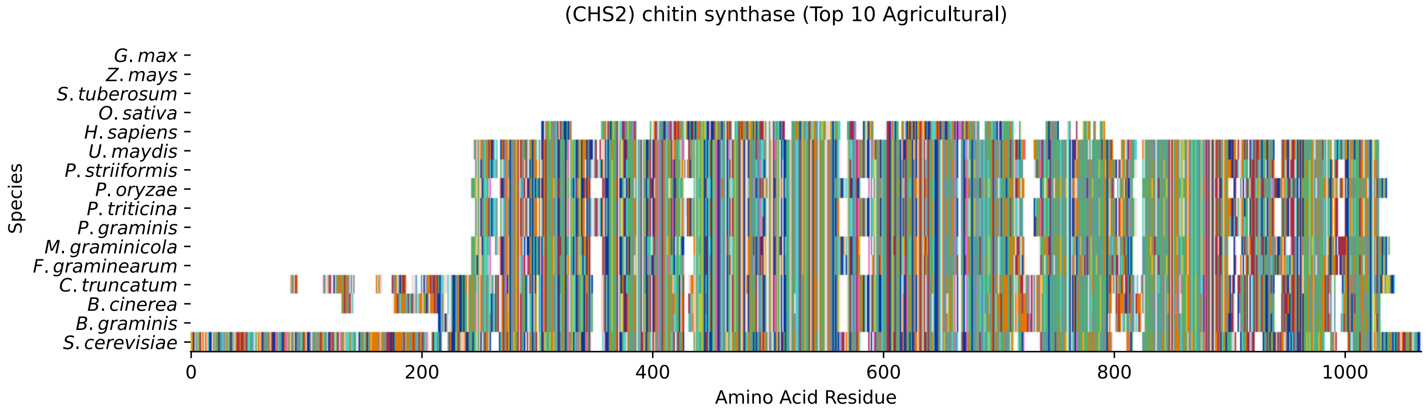

Figure S26: Multiple sequence alignment of yeast Chs2 (Top 10 Agricultural Fungal Pathogens). Cf. Figure S27 for alignment quality, and Figure S28 for Sneath similarity. Cf. Table S11 for protein names, and pairwise alignment metrics with yeast Chs2.

Chs2 MSA Quality

	B. g.	*B. c.*	*C. t.*	*F. g.*	*M. g.*	*P. g.*	*P. t.*	*P. o.*	*P. s.*	*U. m.*	*S. c.*
B. graminis	1612.43	1402.11	1264.98	602.823	608.601	869.766	871.307	598.971	873.618	941.799	558.91
B. cinerea	1425.22	1721.44	1267.29	578.17	595.89	865.144	871.692	578.17	872.848	923.309	555.829
C. truncatum	1264.98	1250.73	1788.08	602.823	608.216	860.136	857.825	587.03	859.751	894.034	562.762
F. graminearum	602.823	576.63	602.823	1519.6	1026.16	628.632	629.402	1237.63	618.616	625.165	628.632
M. graminicola	608.601	594.349	608.216	1026.16	1524.22	604.364	602.438	1067.37	598.201	619.772	632.484
P. graminis	869.766	858.21	860.136	628.632	604.364	1538.47	1508.43	618.616	1479.92	1047.73	545.428
P. triticina	871.307	863.218	857.825	629.402	602.438	1508.43	1536.93	620.157	1472.6	1053.51	544.658
P. oryzae	598.971	577.015	587.03	1237.63	1067.37	618.616	620.157	1509.97	620.157	617.461	640.573
P. striiformis	873.618	864.374	859.751	618.616	598.201	1479.92	1472.6	620.157	1541.17	1046.96	543.502
U. maydis	941.799	914.45	894.034	625.165	619.772	1047.73	1053.51	617.461	1046.96	1539.24	551.977
S. cerevisiae	558.91	551.592	562.762	628.632	632.484	545.428	544.658	640.573	543.502	551.977	2013.81

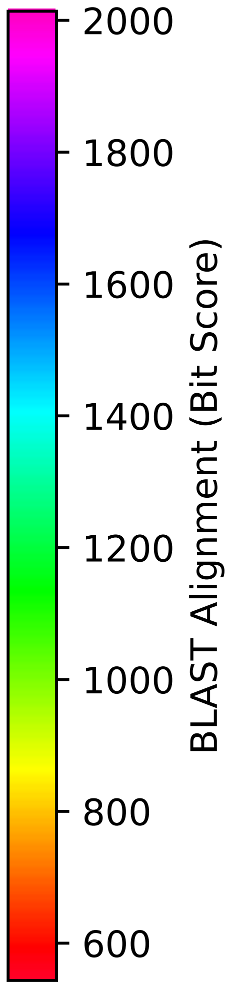

Figure S27: Multiple sequence alignment quality of Chs2 (Top 10 Agricultural Fungal Pathogens). Cf. Figure S26

Species	Hit Protein	Hit Length (a.a.)	evalue	align_len	bit_score	identity	positive	score	gaps	% identity	% positive
G.max	-	-	-	-	-	-	-	-	-	-	-
Z.mays	-	-	-	-	-	-	-	-	-	-	-
S.tuberosum	-	-	-	-	-	-	-	-	-	-	-
O.sativa	-	-	-	-	-	-	-	-	-	-	-
H.sapiens	NP_001186209.1 hyaluronan syn- thase 3 isoform a Homo sapiens	422	1.9e-05	422	51.2174	95	165	121	68	9.9	17.1
U.maydis	XP_011391524.1 chitin synthas- e 1 Ustilago maydis 521	761	0	761	555.444	307	447	1430	94	31.9	46.4
P.striiformis	XP_047799288.1 hypothetical p- rotein Pst134EA 026738 Puccini- a striiformis f. sp. tritici	753	0	753	546.584	306	437	1407	78	31.8	45.4
P.oryzae	mRNA M BR32 EuGene 00045151-p1 — transcript=mRNA M BR32 EuGe- ne 00045151 — gene=M BR32 EuGe- ne 00045151 — organism=Pyricul- aria oryzae BR32 — gene produc- t=unspecified product — transc- ript product=unspecified produ- ct — location=BR32 scaffold000- 04:267300-270156(-) — protein length=886 — sequence SO=super- contig — SO=protein coding gen- e — is pseudo=false	738	0	738	643.269	329	449	1658	54	34.2	46.6
P.triticina	XP_053028600.1 uncharacterize- d protein PtA15 18A101 Puccini- a triticina	754	0	754	547.74	302	435	1410	80	31.4	45.2
P.graminis	XP_003336234.2 chitin synthas- e Puccinia graminis f. sp. tri- tici CRL 75-36-700-3	754	0	754	548.895	303	434	1413	80	31.5	45.1
M.graminicola	ZTRI 1.444.mRNA-p1 — transcrip- t=ZTRI 1.444.mRNA — gene=ZTRI 1.444 — organism=Zymoseptoria tritici IPO323 — gene product=- similar to chitin synthase 2 — transcript product=similar to chitin synthase 2 — location=- Ztri chr 1:1478238-1481305(-) — protein length=946 — sequenc- e SO=chromosome — SO=protein c- oding gene — is pseudo=false	744	0	744	634.41	331	457	1635	57	34.4	47.5
F.graminearum	XP_011319287.1 chitin synthas- e 3 Fusarium graminearum PH-1	742	0	742	630.558	329	452	1625	58	34.2	46.9
C.truncatum	XP_036585044.1 chitin synthas- e Colletotrichum truncatum	888	0	888	562.762	338	486	1449	97	35.1	50.5
B.cinerea	XP_001557191.1 BcCHSIIIa Botr- ytis cinerea B05.10	835	0	835	553.132	326	467	1424	92	33.9	48.5
B.graminis	VCU40918.1 — transcript=BGT962- 24V316 LOCUS2169 t1 — gene=BGT- 96224V316 LOCUS2169 — organism- =Blumeria graminis f. sp. trit- ici 96224 — gene product=unspe- cified product — transcript pr- oduct=unspecified product — lo- cation=LR026987:2394926-239780- 7(+) — protein length=900 — se- quence SO=chromosome — SO=prot- ein coding gene — is pseudo=fa- lse	792	0	792	559.296	314	451	1440	87	32.6	46.8

Table S11: Pairwise alignment info from yeast Chs2 (DEG20010039), cf. Figure S26.

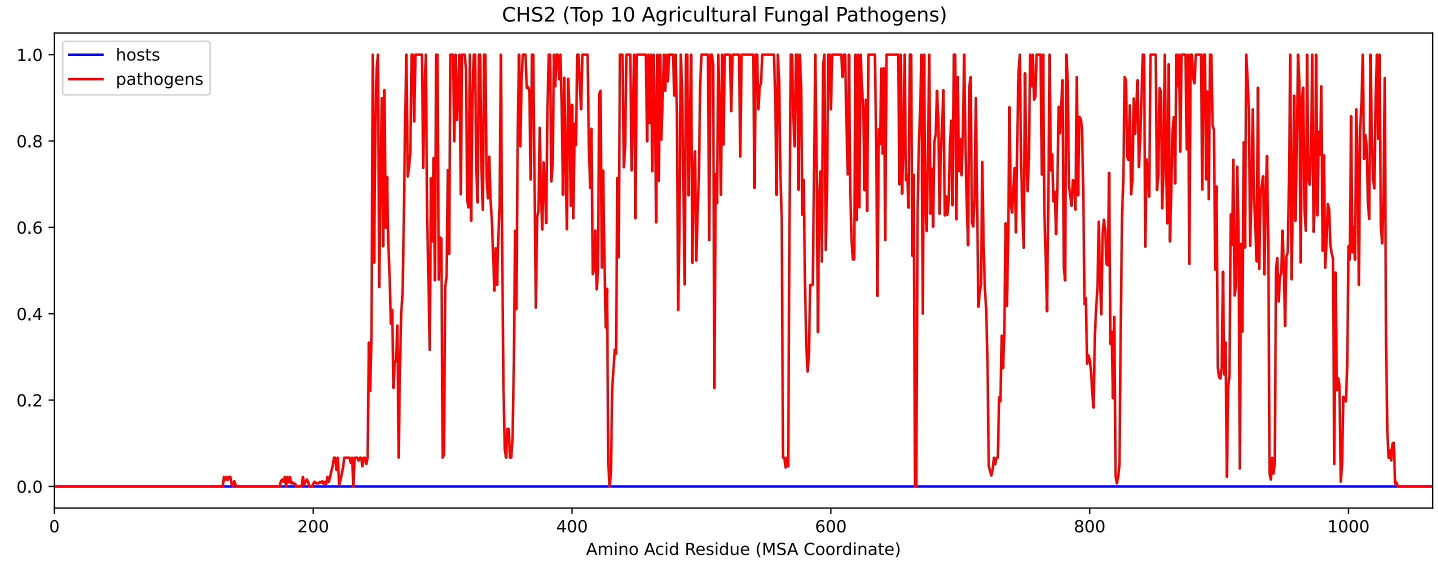

Figure S28: Sneath Similarity of Chs2 for Top 10 Agricultural Fungal Pathogens, cf. Figure S26

S2.3.3 NR

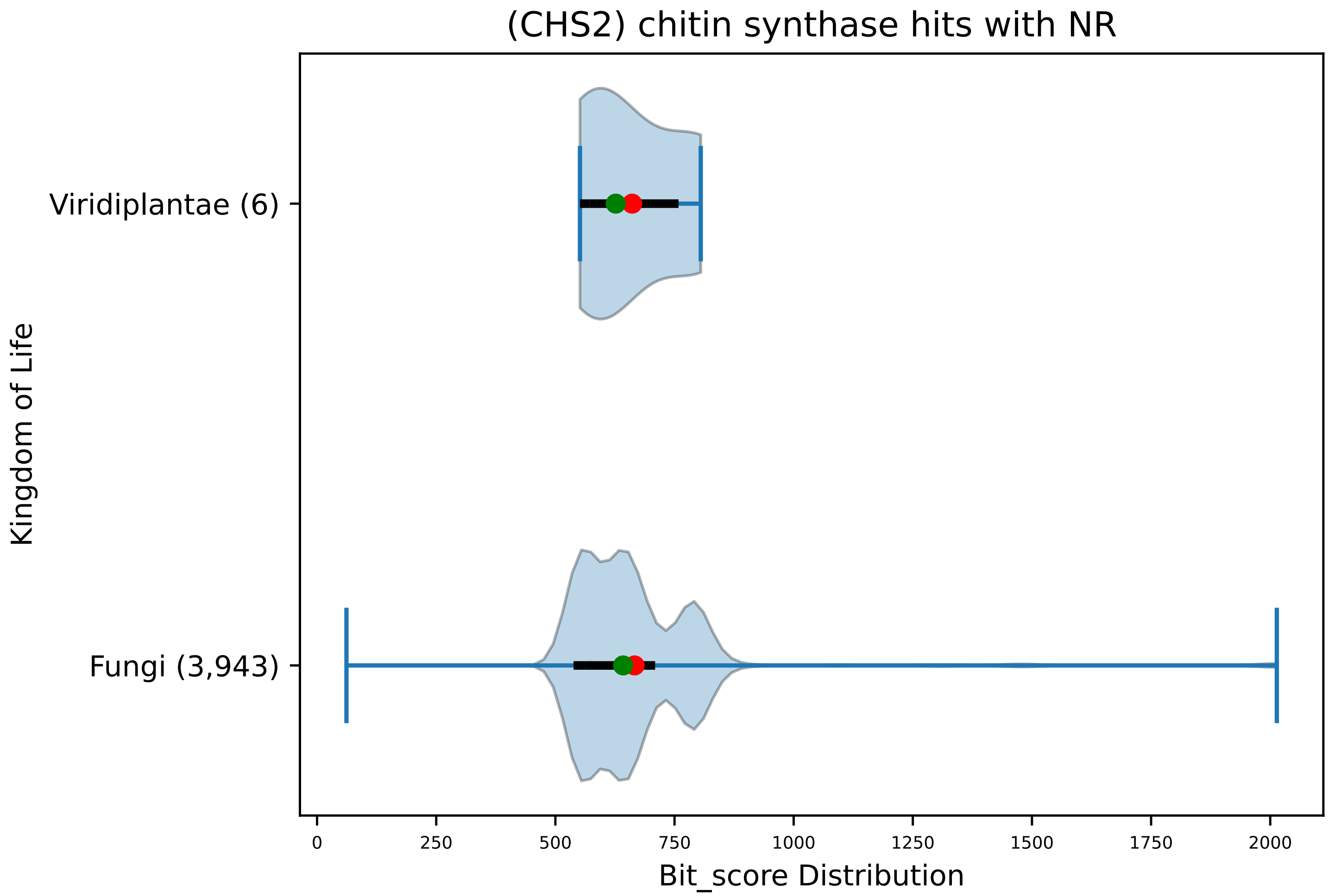

Figure S29: Non-redundant (NR) protein hits for DEG20010039/Chs2, with expectation value of no more than 0.1. Green points are medians, and red points are arithmetic means.

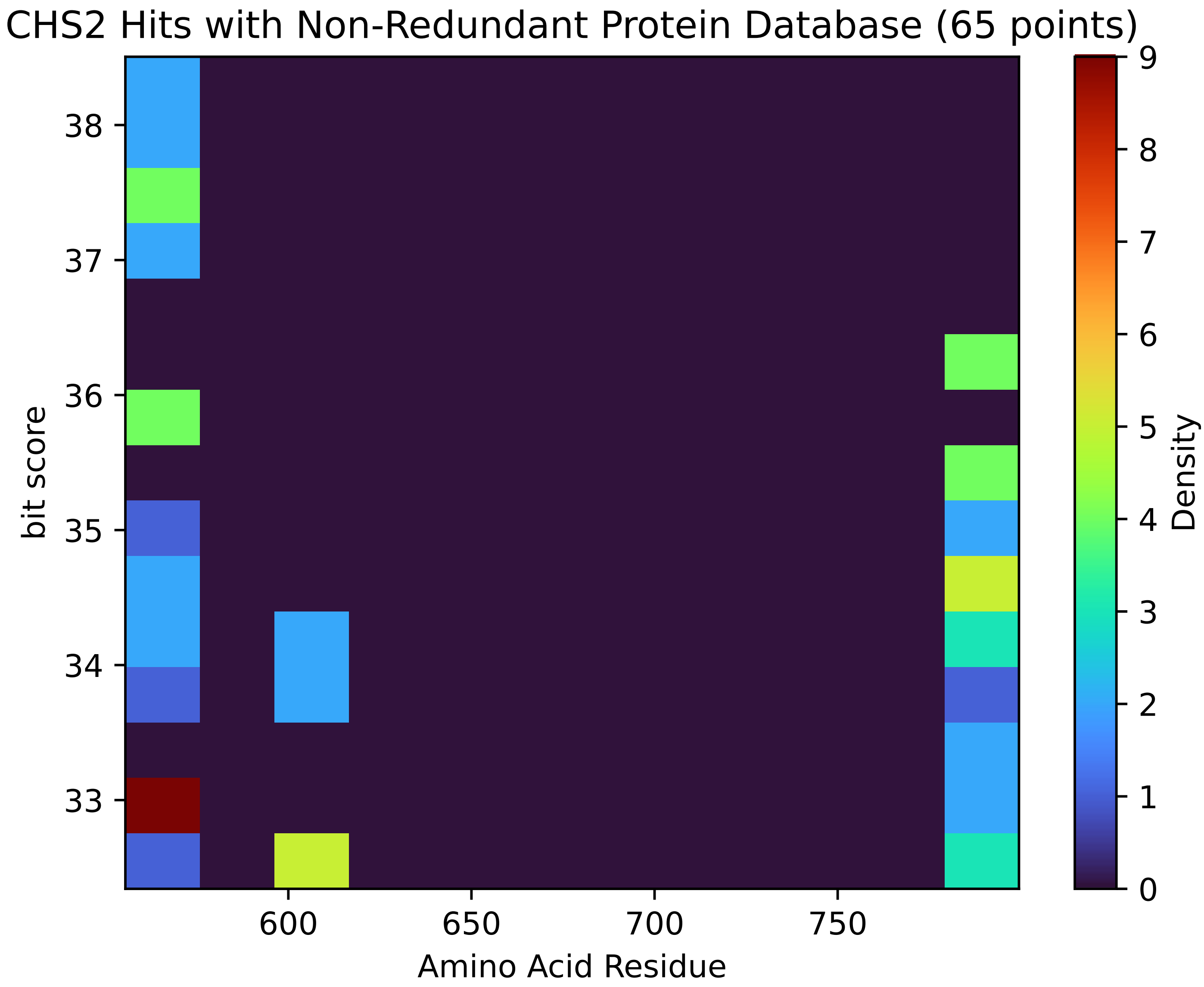

Figure S30: Non-redundant (NR) protein hits for Chs2 in the kingdom Viridiplantae.

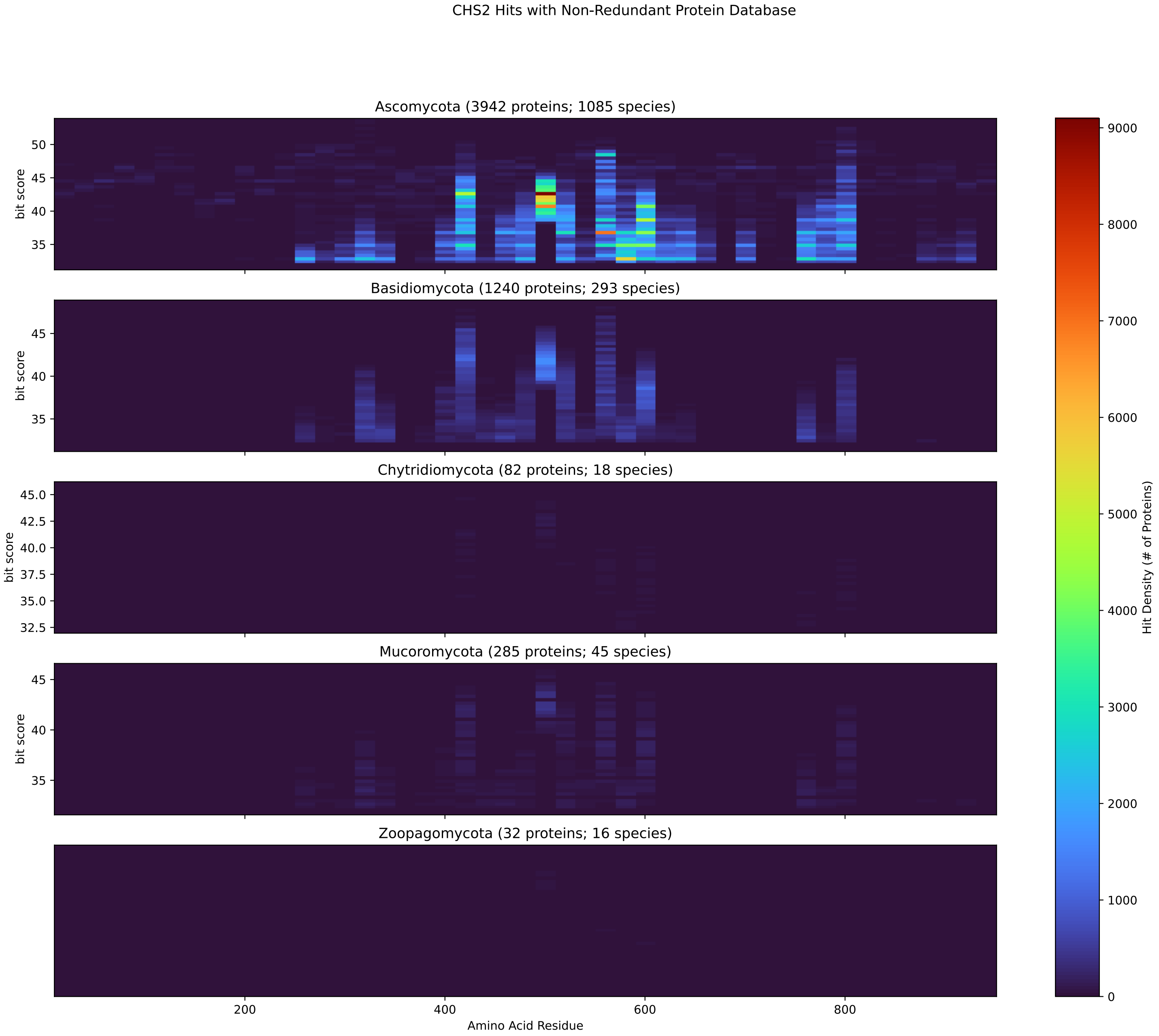

Figure S31: Non-redundant (NR) protein hits for Chs2 in the kingdom Fungi.

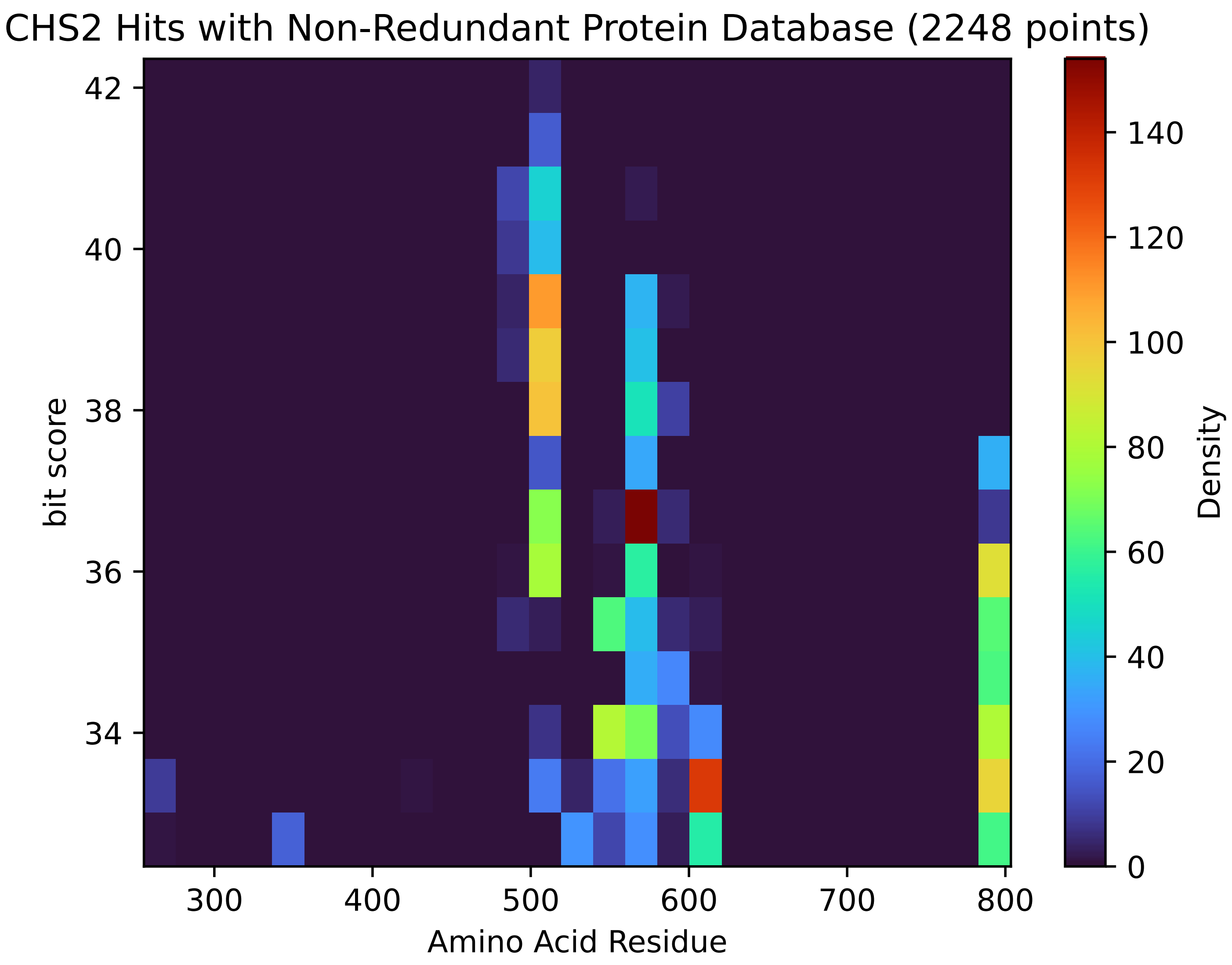

Figure S32: Non-redundant (NR) protein hits for Chs2 in the kingdom SAR.

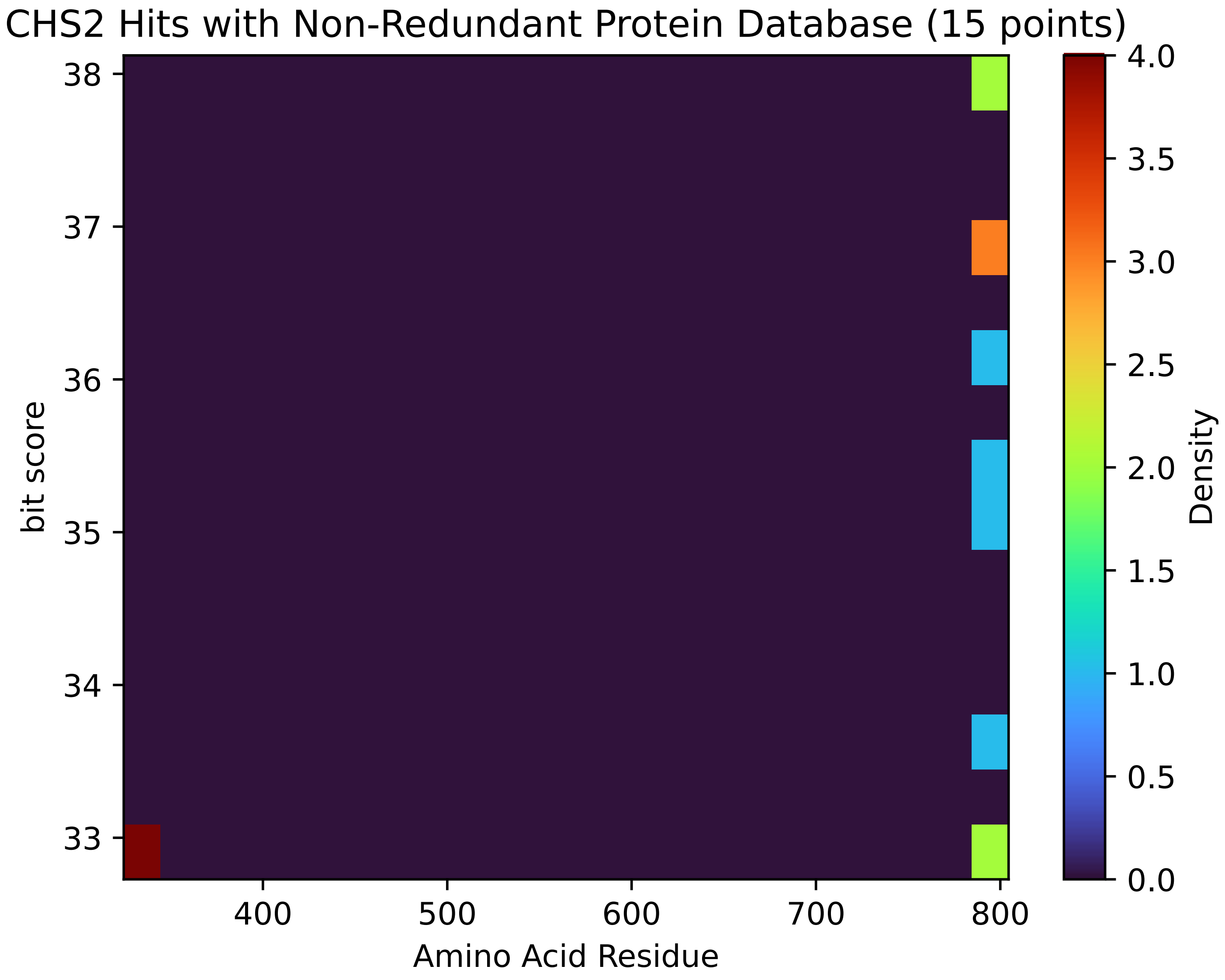

Figure S33: Non-redundant (NR) protein hits for Chs2 in the kingdom Metazoa.

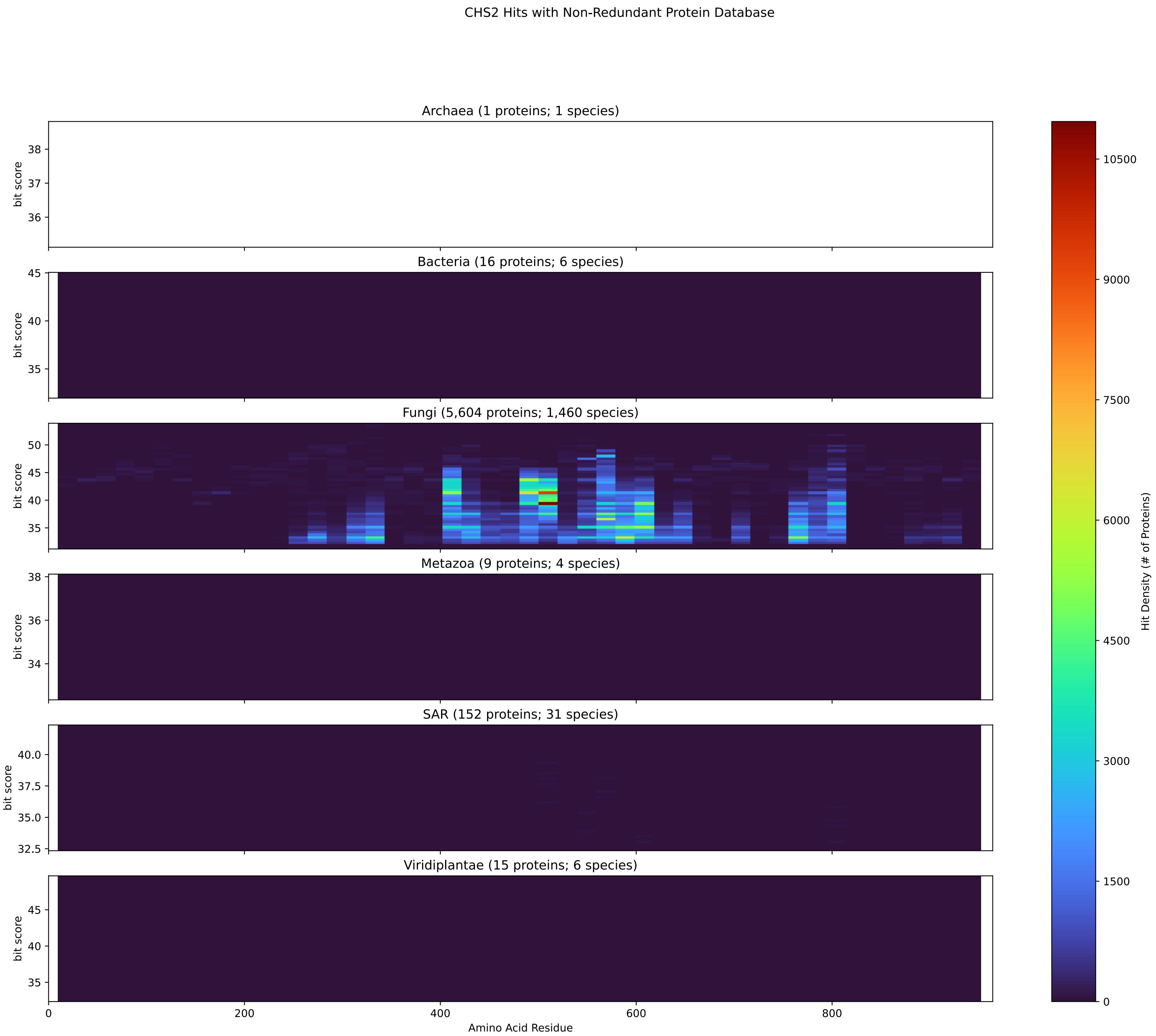

Figure S34: Non-redundant (NR) protein hits for DEG20010039/Chs2 at 20 amino acid length queries.

S2.4 Erg8

S2.4.1 WHO Critical Pathogens

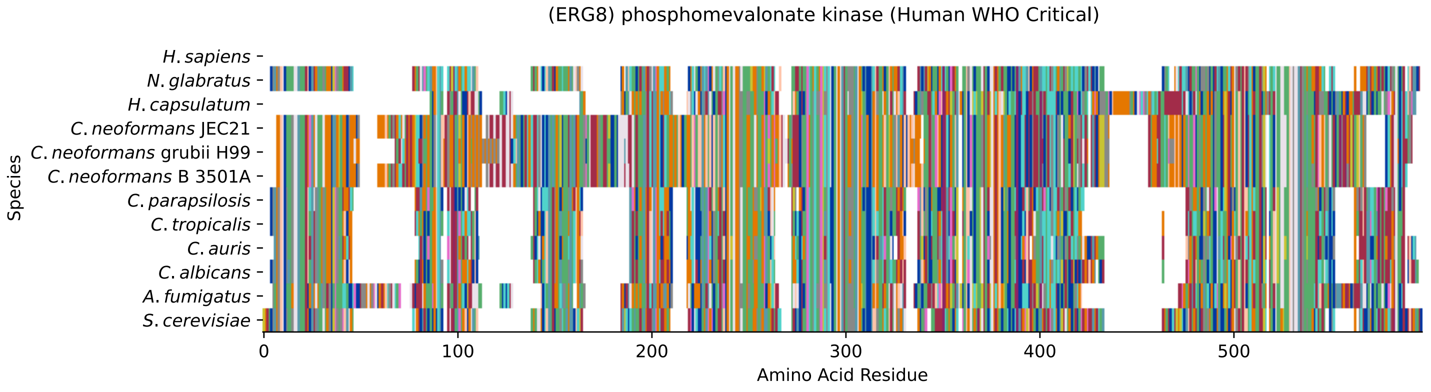

Figure S35: Multiple sequence alignment of yeast Erg8 (WHO Critical Pathogens). Cf. Figure S36 for alignment quality, and Figure S37 for Sneath similarity. Cf. Table S12 for protein names, and pairwise alignment metrics with yeast Erg8.

Erg8 MSA Quality

	A. f.	*C. a.*	*C. a.*	*C. p.*	*C. t.*	*C. n.*	*C. n.*	*C. n.*	*H. c.*	*N. g.*	*S. c.*
A. fumigatus	961.444	220.32	218.394	248.44	231.876	196.438	202.216	200.29	350.132	217.238	214.927
C. albicans	238.424	883.248	478.019	461.84	641.728	162.925	179.874	171.785	132.109	365.925	315.849
C. auris	214.927	444.121	876.7	415.231	439.113	158.303	176.022	165.622	126.331	370.548	327.02
C. parapsilosis	241.891	431.409	415.231	847.81	479.174	155.221	161.384	156.377	172.17	344.354	297.745
C. tropicalis	228.409	607.06	439.113	479.174	838.951	153.68	157.147	151.754	164.466	356.681	307.76
C. neoformans B 3501A	229.18	174.866	191.43	185.652	186.422	1092.41	887.871	1061.98	171.4	166.392	177.178
C. neoformans grubii H99	225.713	183.726	202.986	187.193	184.496	855.129	1081.24	878.241	173.326	193.356	178.718
C. neoformans JEC21	228.409	175.637	195.667	184.496	177.948	1061.98	897.116	1097.03	176.792	172.17	179.874
H. capsulatum	427.557	165.622	190.66	193.356	180.644	167.933	170.629	172.555	872.848	164.851	167.933
N. glabratus	213.772	337.806	370.548	344.354	356.681	134.806	160.229	135.961	150.984	909.442	409.068
S. cerevisiae	204.912	296.59	327.02	297.745	307.76	145.976	154.066	150.599	142.51	409.068	941.028

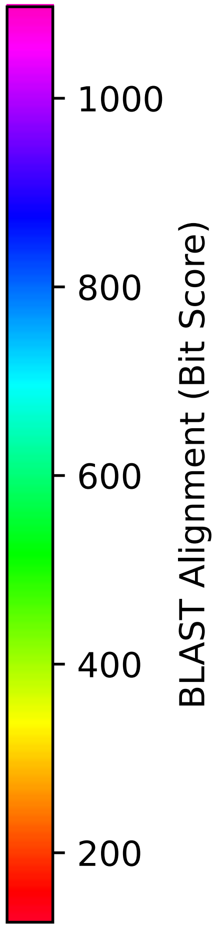

Figure S36: Multiple sequence alignment quality of Erg8 (WHO Critical Pathogens). Cf. Figure S35

Species	Hit Protein	Hit Length (a.a.)	evalue	align_len	bit_score	identity	positive	score	gaps	% identity	% positive
H.sapiens	-	-	-	-	-	-	-	-	-	-	-
N.glabratus	XP_446144.1 uncharacterized p-rotein CAGL0F03993g Nakaseomyc-es glabratus	449	4.9e-141	449	409.453	210	298	1051	11	46.6	66.1
H.capsulatum	XP_045287201.1 phosphomevalon-ate kinase Histoplasma capsula-tum G186AR	446	1.6e-37	446	142.51	132	202	358	72	29.3	44.8
C.neoformans.JEC21	XP_568385.1 expressed protein Cryptococcus neoformans var. neoformans JEC21	546	1.9e-40	546	150.599	159	240	379	115	35.3	53.2
C.neoformans.grubii.H99	XP_012053077.1 phosphomevalon-ate kinase Cryptococcus neofor-mans var. grubii H99	536	1.6e-41	536	153.68	156	244	387	102	34.6	54.1
C.neoformans.B.3501A	XP_772275.1 hypothetical prot-ein CNBM0120 Cryptococcus neof-ormans var. neoformans B-3501A	540	6.5e-39	540	146.362	152	237	368	108	33.7	52.5
C.parapsilosis	XP_036667432.1 uncharacterize-d protein CPAR2 400710 Candida parapsilosis	446	1.1e-97	446	297.745	179	256	761	44	39.7	56.8
C.tropicalis	XP_002545449.1 hypothetical p-rotein CTRG 00230 Candida trop-icalis MYA-3404	448	3.5e-101	448	307.375	182	265	786	46	40.4	58.8
C.auris	XP_028891858.2 phosphomevalon-ate kinase Candida auris	448	6.1e-109	448	327.02	197	255	837	33	43.7	56.5
C.albicans	XP_722678.1 phosphomevalonate kinase Candida albicans SC531-4	454	4.7e-97	454	296.59	191	269	758	35	42.4	59.6
A.fumigatus	XP_753576.1 phosphomevalonate kinase Aspergillus fumigatus Af293	487	5.3e-61	487	204.912	157	233	520	71	34.8	51.7

Table S12: Pairwise alignment info from yeast Erg8 (DEG20010822), cf. Figure S35.

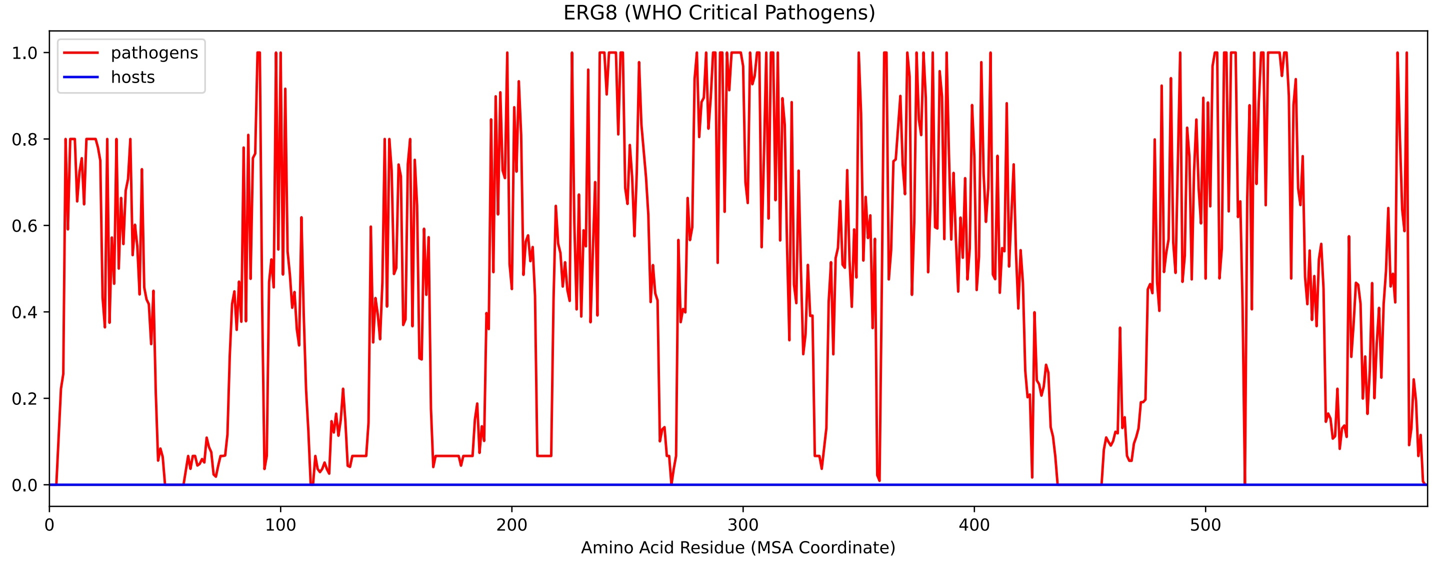

Figure S37: Sneath Similarity of Erg8 for WHO Critical Pathogens, cf. Figure S35

S2.4.2 Top 10 Agricultural Fungal Pathogens

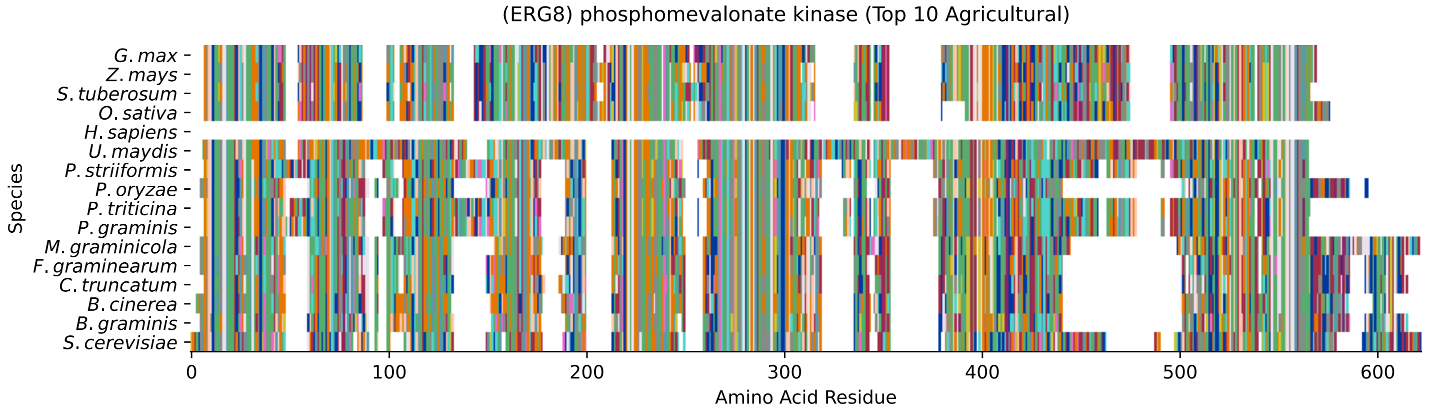

Figure S38: Multiple sequence alignment of yeast Erg8 (Top 10 Agricultural Fungal Pathogens). Cf. Figure S39 for alignment quality, and Figure S40 for Sneath similarity. Cf. Table S13 for protein names, and pairwise alignment metrics with yeast Erg8.

Erg8 MSA Quality

	B.g.	B.c.	C.t.	F.g.	M.g.	P.g.	P.t.	P.o.	P.s.	U.m.	S.c.
B.graminis	882.093	407.142	379.407	392.119	332.798	209.149	210.305	369.777	216.853	187.578	213.772
B.cinerea	391.734	863.218	483.411	483.026	401.749	222.246	222.246	454.136	226.098	180.259	228.794
C.truncatum	355.14	476.478	876.7	600.127	378.252	236.113	237.654	581.252	232.261	184.882	256.529
F.graminearum	370.933	477.633	600.127	874.774	390.578	225.328	229.565	540.421	216.468	174.096	251.521
M.graminicola	360.918	450.284	428.713	442.195	927.546	224.942	228.409	412.535	241.891	187.963	242.662
P.graminis	191.43	217.624	236.113	225.328	184.882	929.858	746.503	243.432	745.732	241.891	198.749
P.triticina	192.971	219.935	237.654	229.565	187.963	746.503	927.161	247.669	719.924	244.588	181.8
P.oryzae	347.051	450.669	581.252	540.421	365.155	243.432	247.669	822.772	241.121	181.415	245.358
P.striiformis	199.904	223.402	232.261	216.468	201.83	745.732	719.924	241.121	937.562	243.817	176.022
U.maydis	173.326	177.563	184.496	173.711	150.984	244.588	244.588	191.43	245.358	1056.59	155.992
S.cerevisiae	196.052	226.868	256.529	251.521	205.297	198.749	181.8	245.358	176.022	155.606	941.028

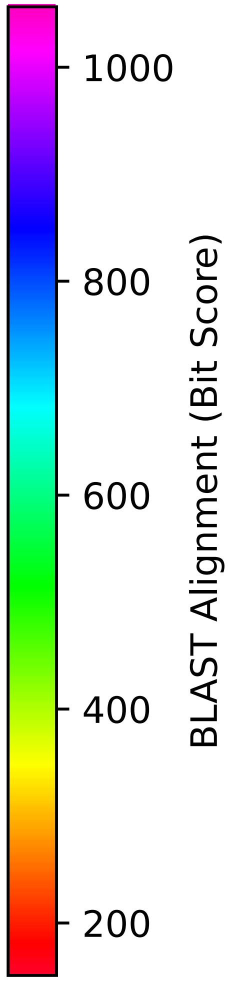

Figure S39: Multiple sequence alignment quality of Erg8 (Top 10 Agricultural Fungal Pathogens). Cf. Figure S38

Species	Hit Protein	Hit Length (a.a.)	evalue	align_len	bit_score	identity	positive	score	gaps	% identity	% positive
G.max	XP_003526704.1 phosphomevalonate kinase, peroxisomal isoform X3 Glycine max	462	2.3e-42	462	157.918	143	217	398	66	31.7	48.1
Z.mays	NP_001355169.1 phosphomevalonate kinase Zea mays	470	2.2e-51	470	182.185	151	217	461	78	33.5	48.1
S.tuberosum	XP_006352929.1 PREDICTED: phosphomevalonate kinase-like Solanum tuberosum	455	3.3e-53	455	186.422	149	222	472	56	33.0	49.2
O.sativa	XP_015632523.1 phosphomevalonate kinase, peroxisomal isoform X2 Oryza sativa Japonica Group	473	3.6e-48	473	172.94	148	212	437	81	32.8	47.0
H.sapiens	-	-	-	-	-	-	-	-	-	-	-
U.maydis	XP_011386538.1 phosphomevalonate kinase Ustilago maydis 521	526	9.7e-42	526	155.221	148	214	391	131	32.8	47.5
P.striiformis	XP_047806802.1 hypothetical protein Pst134EA 013927 Puccinia striiformis f. sp. tritici	459	2.6e-49	459	175.637	141	217	444	63	31.3	48.1
P.oryzae	mRNA M BR32 EuGene 00127411-p1 — transcript=mRNA M BR32 EuGene 00127411 — gene=M BR32 EuGene 00127411 — organism=Pyricularia oryzae BR32 — gene product=unspecified product — transcript product=unspecified product — location=BR32 scaffold000-23:241792-243188(+) — protein length=445 — sequence SO=supercontig — SO=protein coding gene — is pseudo=false	451	9.1e-77	451	245.358	164	242	625	58	36.4	53.7
P.triticina	XP_053022619.1 uncharacterized protein PtA15 7A793 Puccinia triticina	458	1.1e-51	458	182.185	144	222	461	63	31.9	49.2
P.graminis	XP_003888790.1 hypothetical protein PGTG 22505 Puccinia graminis f. sp. tritici CRL 75-36-700-3	460	7.7e-58	460	198.364	145	222	503	67	32.2	49.2
M.graminicola	ZTRI 9.521.mRNA-p1 — transcript=ZTRI 9.521.mRNA — gene=ZTRI 9.521 — organism=Zymoseptoria tritici IPO323 — gene product=similar to phosphomevalonate kinase — transcript product=similar to phosphomevalonate kinase — location=Ztri chr 9:17393-92-1740886(-) — protein length=464 — sequence SO=chromosome — SO=protein coding gene — is pseudo=false	478	4.3e-61	478	204.912	166	244	520	59	36.8	54.1
F.graminearum	XP_011327956.1 hypothetical protein FGSG 09764 Fusarium graminearum PH-1	461	4.6e-79	461	251.521	164	244	641	59	36.4	54.1
C.truncatum	XP_036588544.1 phosphomevalonate kinase Colletotrichum truncatum	467	1.5e-80	467	255.758	173	248	652	68	38.4	55.0
B.cinerea	XP_001553931.1 Bcerg8 Botrytis cinerea B05.10	460	1.9e-68	460	226.098	155	233	575	55	34.4	51.7
B.graminis	VDB92769.1 — transcript=BGT962-24V316 LOCUS6538 t1 — gene=BGT-96224V316 LOCUS6538 — organism=Blumeria graminis f. sp. tritici 96224 — gene product=unspecified product — transcript product=unspecified product — location=LR026992:3440777-344216-8(-) — protein length=438 — sequence SO=chromosome — SO=protein coding gene — is pseudo=false	462	3.7e-58	462	195.667	159	220	496	60	35.3	48.8

Table S13: Pairwise alignment info from yeast Erg8 (DEG20010822), cf. Figure S38.

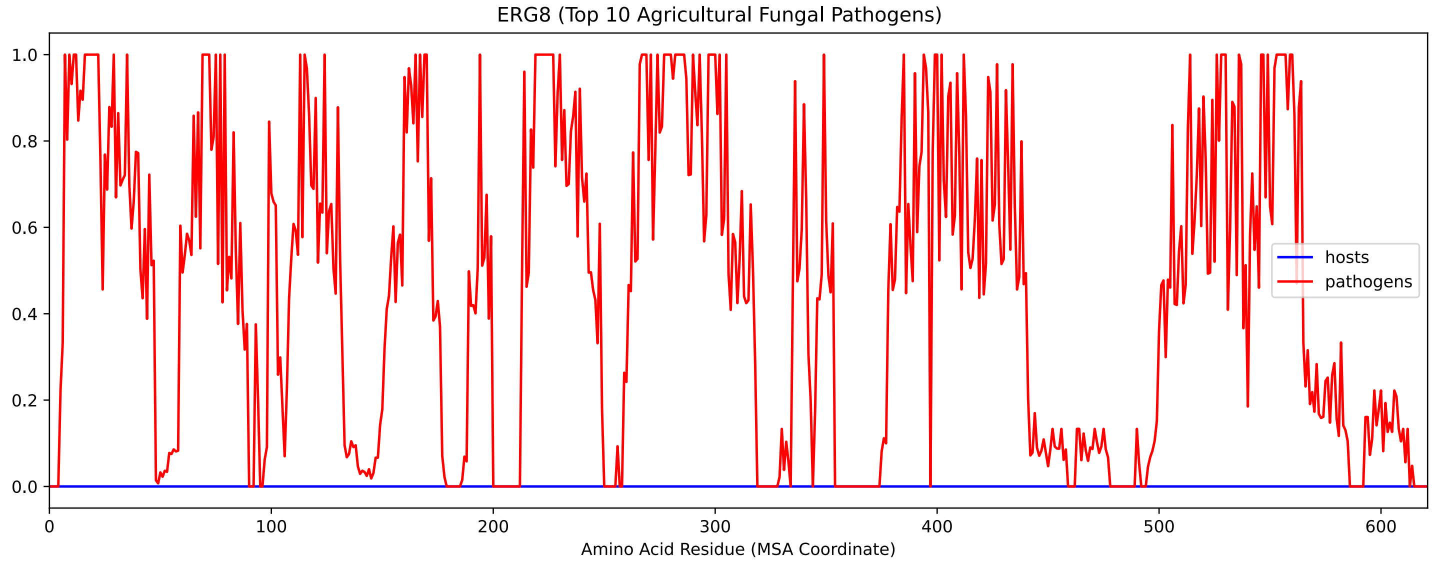

Figure S40: Sneath Similarity of Erg8 for Top 10 Agricultural Fungal Pathogens, cf. Figure S38

S2.4.3 NR

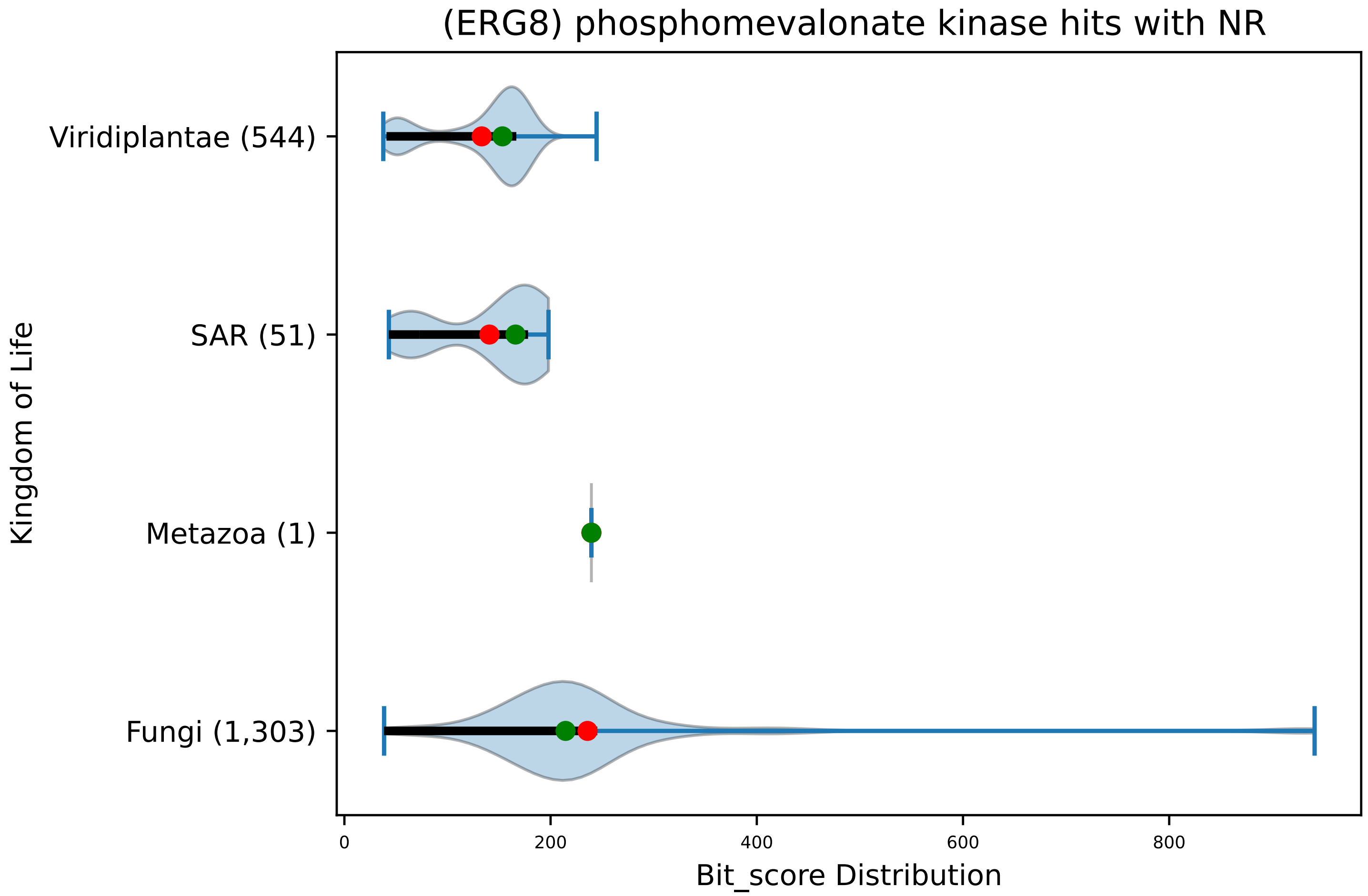

Figure S41: Non-redundant (NR) protein hits for DEG20010822/Erg8, with expectation value of no more than 0.1. Green points are medians, and red points are arithmetic means.

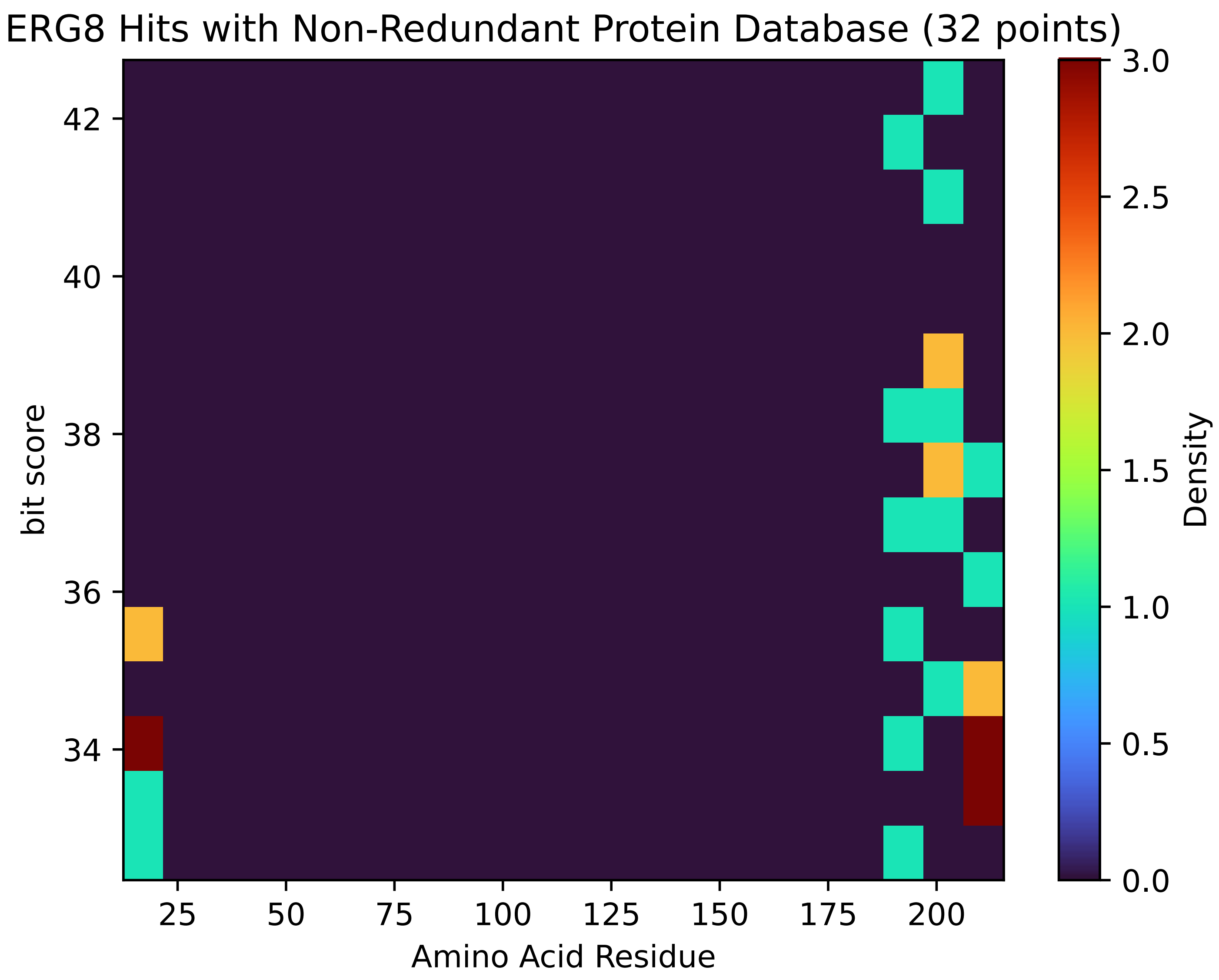

Figure S42: Non-redundant (NR) protein hits for Erg8 in the kingdom Metazoa.

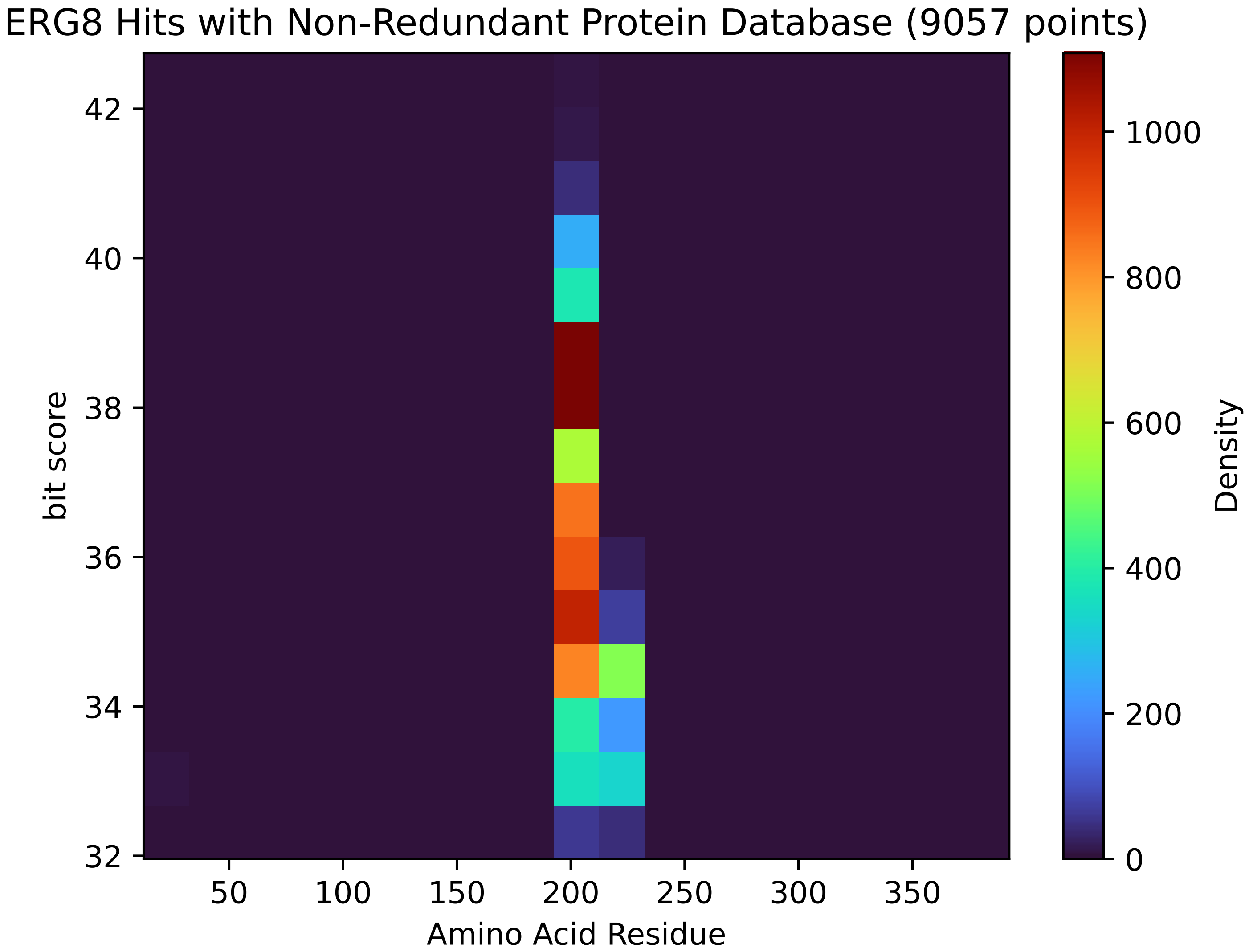

Figure S43: Non-redundant (NR) protein hits for Erg8 in the kingdom Viridiplantae.

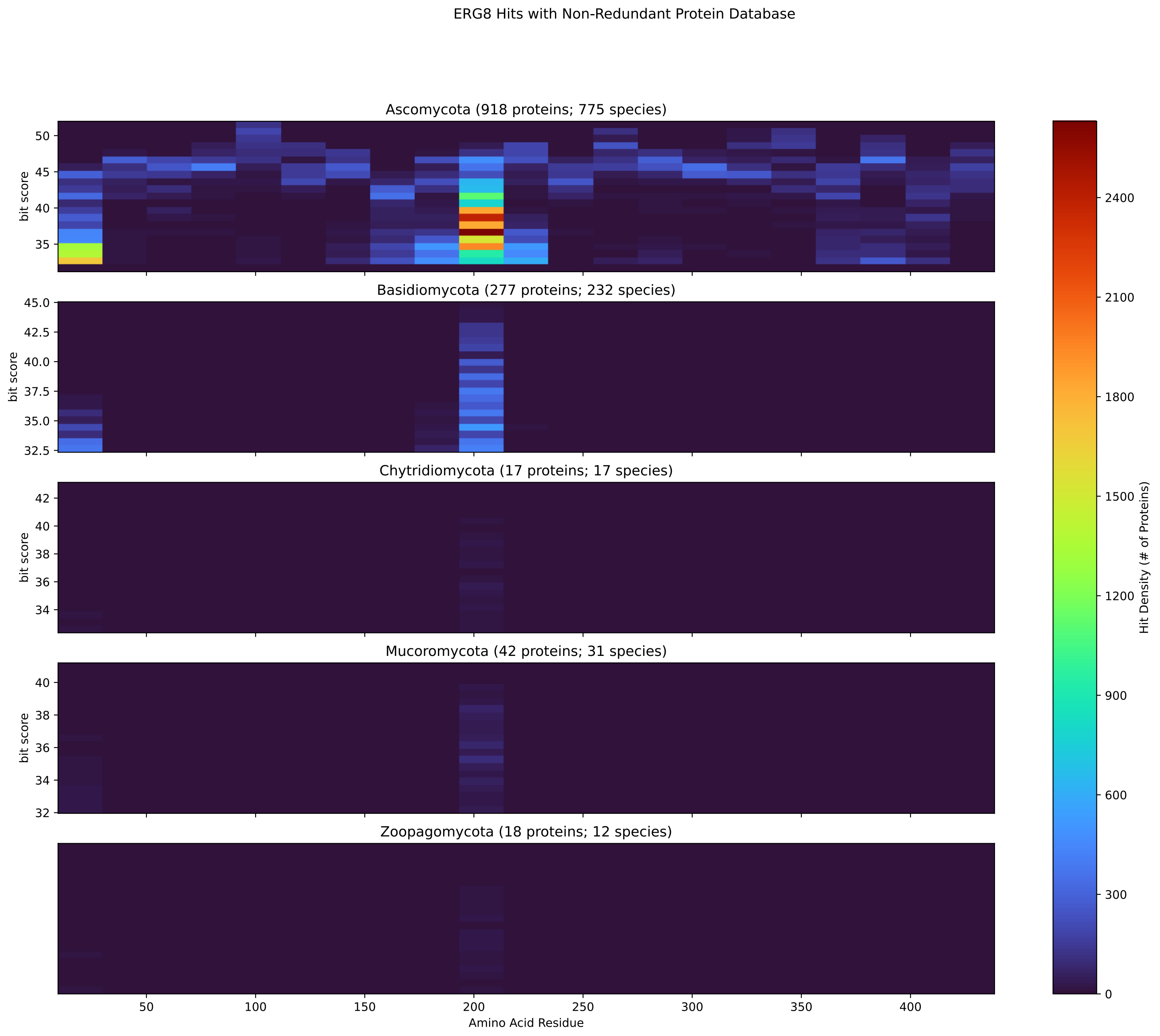

Figure S44: Non-redundant (NR) protein hits for Erg8 in the kingdom Fungi.

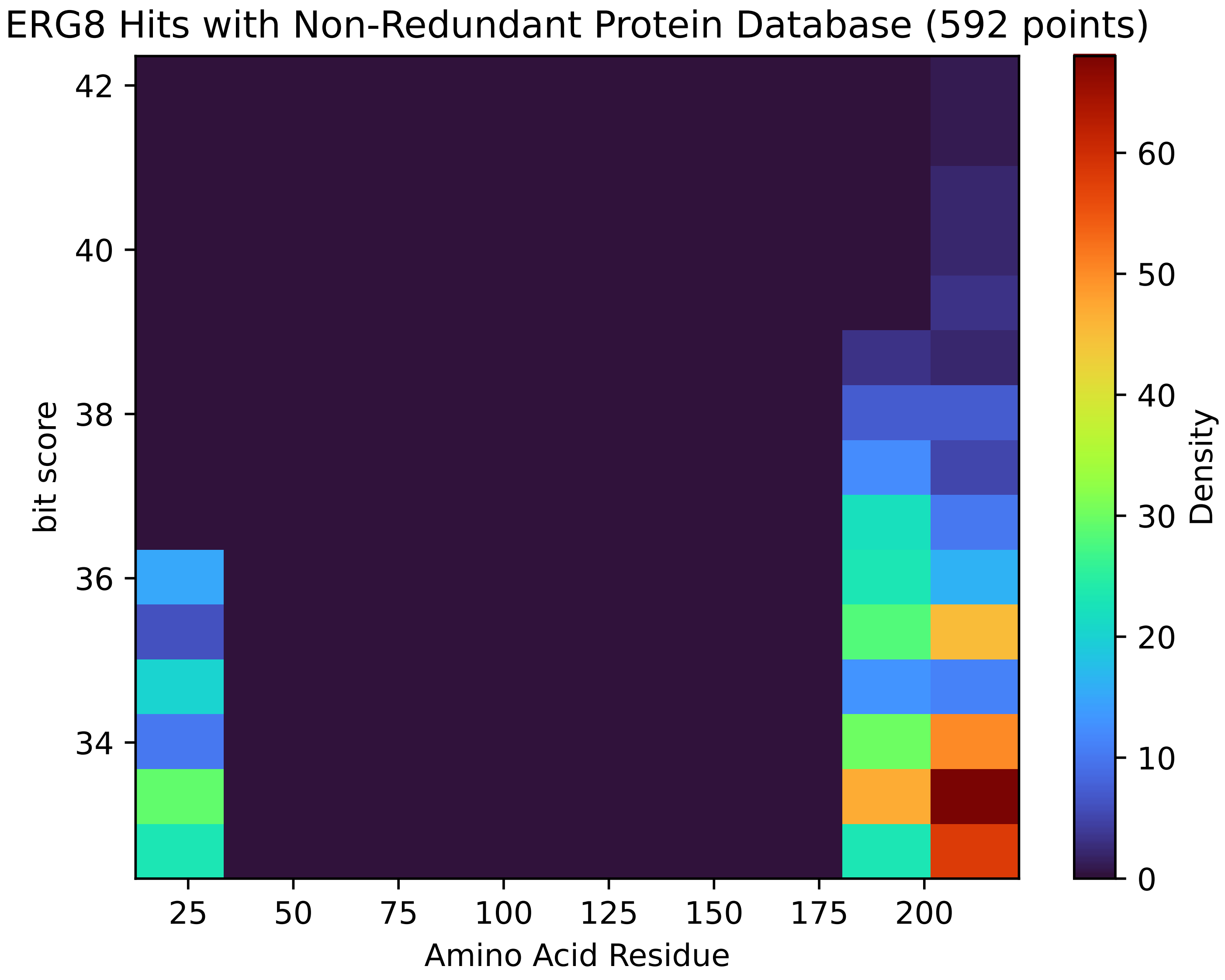

Figure S45: Non-redundant (NR) protein hits for Erg8 in the kingdom SAR.

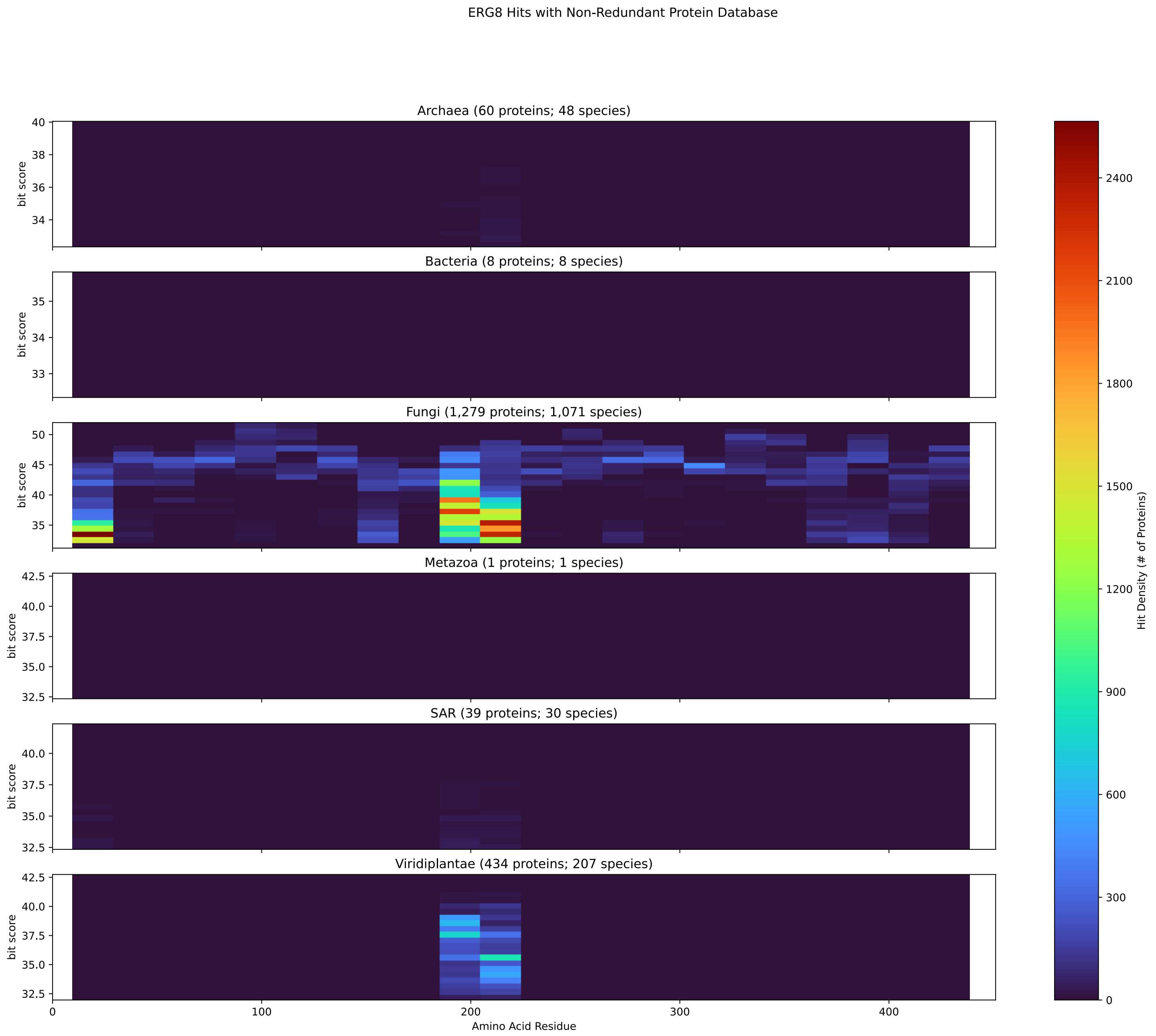

Figure S46: Non-redundant (NR) protein hits for DEG20010822/Erg8 at 20 amino acid length queries.

S2.5 Fas1

S2.5.1 WHO Critical Pathogens

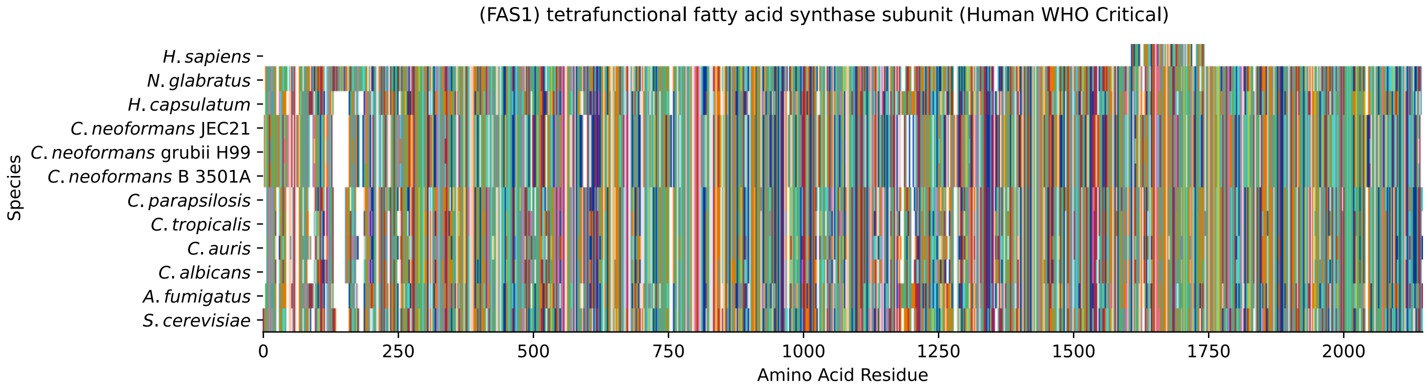

Figure S47: Multiple sequence alignment of yeast Fas1 (WHO Critical Pathogens). Cf. Figure S48 for alignment quality, and Figure S49 for Sneath similarity. Cf. Table S14 for protein names, and pairwise alignment metrics with yeast Fas1.

Fas1 MSA Quality

	A. f.	*C. a.*	*C. a.*	*C. p.*	*C. t.*	*C. n.*	*C. n.*	*C. n.*	*H. c.*	*N. g.*	*S. c.*
A. fumigatus	4259.91	2433.68	2511.1	2443.31	2441.77	1953.33	1931.38	1954.87	3672.48	2353.56	2397.85
C. albicans	2417.11	4202.51	3258.77	3476.8	3781.49	1830.84	1817.36	1830.45	2448.7	2713.72	2620.88
C. auris	2490.69	3258.77	4256.44	3270.72	3245.68	1869.36	1855.88	1869.74	2519.96	2764.18	2702.16
C. parapsilosis	2421.74	3476.8	3270.72	4229.48	3483.73	1780.38	1767.67	1780.38	2436.76	2681.75	2609.71
C. tropicalis	2423.28	3781.49	3245.68	3483.73	4207.91	1826.22	1807.34	1824.29	2447.16	2686.75	2628.59
C. neoformans B 3501A	1935.61	1830.84	1869.36	1780.38	1826.22	4265.3	4184.79	4259.52	1973.75	1797.71	1788.08
C. neoformans grubii H99	1932.53	1831.61	1872.06	1785	1823.52	4184.79	4266.46	4187.11	1974.52	1797.71	1785.39
C. neoformans JEC21	1936.77	1830.45	1869.74	1780.38	1824.29	4259.52	4187.11	4268.77	1978.37	1799.64	1789.62
H. capsulatum	3632.8	2448.7	2519.96	2436.76	2447.16	1973.75	1958.34	1978.37	4257.98	2357.79	2371.66
N. glabratus	2340.07	2713.72	2764.18	2681.75	2686.75	1797.71	1779.61	1799.64	2357.79	4296.12	3236.43
S. cerevisiae	2383.99	2624.35	2709.09	2615.49	2630.9	1795.4	1774.99	1795.4	2385.53	3248.76	4237.18

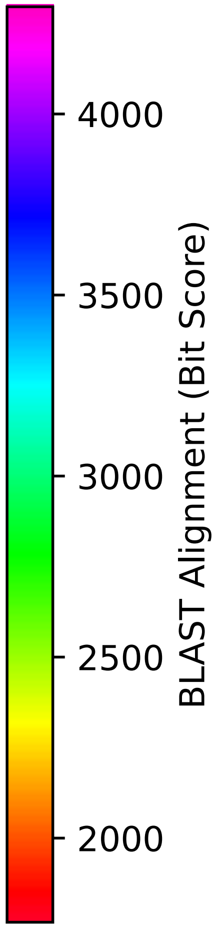

Figure S48: Multiple sequence alignment quality of Fas1 (WHO Critical Pathogens). Cf. Figure S47

Species	Hit Protein	Hit Length (a.a.)	evalue	align_len	bit_score	identity	positive	score	gaps	% identity	% positive
H.sapiens	NP_001278957.1 peroxisomal mu-ltifunctional enzyme type 2 is-oform 5 Homo sapiens	133	2.1e-06	133	55.4546	40	64	132	9	2.0	3.1
N.glabratus	XP_445436.1 uncharacterized p-rotein CAGL0D00528g Nakaseomyc-es glabratus	2083	0	2083	3247.6	1545	1782	8419	44	75.3	86.9
H.capsulatum	XP_045283903.1 fatty acid syn-thase beta subunit dehydratase Histoplasma capsulatum G186AR	2069	0	2069	2385.14	1151	1510	6180	34	56.1	73.6
C.neoformans.JEC21	XP_571100.1 fatty-acid syntha-se complex protein, putative C-ryptococcus neoformans var. ne-oformans JEC21	2090	0	2090	1798.1	933	1327	4656	66	45.5	64.7
C.neoformans.grubii.H99	XP_012049942.1 fatty acid syn-thase subunit beta, fungi type Cryptococcus neoformans var. grubii H99	2089	0	2089	1774.6	924	1316	4595	64	45.1	64.2
C.neoformans.B.3501A	XP_775164.1 hypothetical prot-ein CNBE4370 Cryptococcus neof-ormans var. neoformans B-3501A	2090	0	2090	1795.4	933	1327	4649	66	45.5	64.7
C.parapsilosis	XP_036664454.1 uncharacterize-d protein CPAR2 302650 Candida parapsilosis	2064	0	2064	2615.49	1280	1576	6778	42	62.4	76.8
C.tropicalis	XP_002550943.1 fatty acid syn-thase beta subunit dehydratase Candida tropicalis MYA-3404	2059	0	2059	2630.51	1279	1576	6817	40	62.4	76.8
C.auris	XP_028891947.2 fatty acid syn-thase subunit beta Candida aur-is	2058	0	2058	2711.02	1291	1601	7026	16	62.9	78.1
C.albicans	XP_716817.1 tetrafunctional f-atty acid synthase subunit Can-dida albicans SC5314	2061	0	2061	2624.74	1278	1586	6802	46	62.3	77.3
A.fumigatus	XP_748739.1 fatty acid syntha-se beta subunit, putative Aspe-rgillus fumigatus Af293	2071	0	2071	2387.07	1173	1510	6185	37	57.2	73.6

Table S14: Pairwise alignment info from yeast Fas1 (DEG20010641), cf. Figure S47.

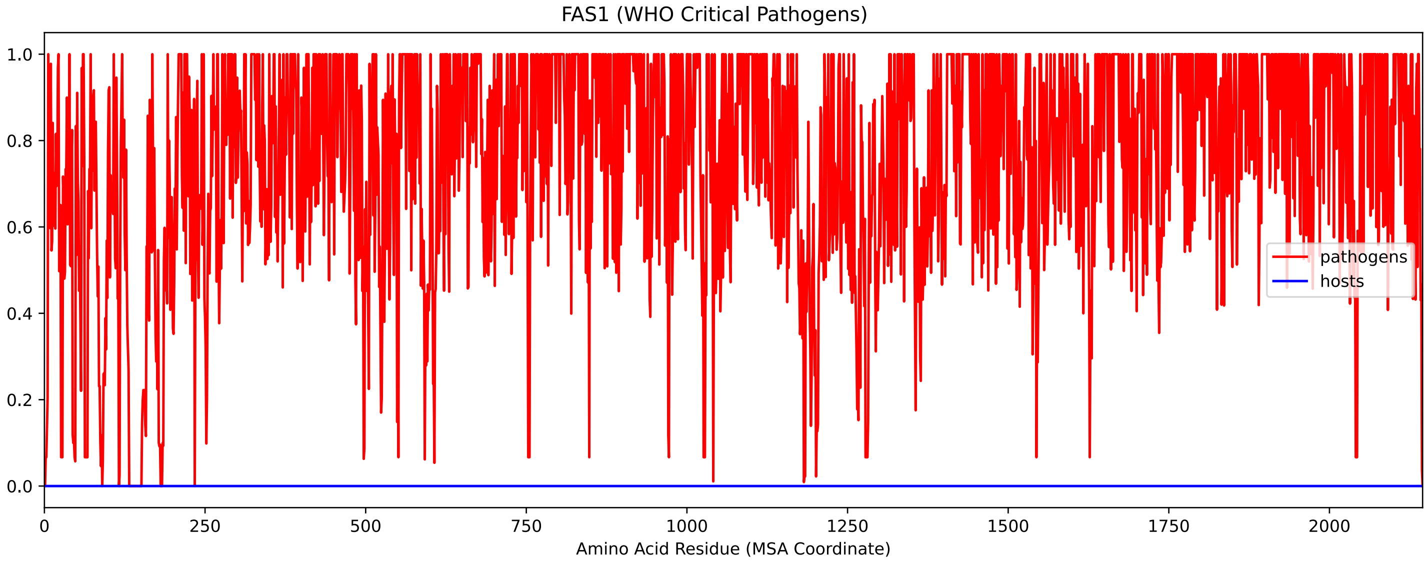

Figure S49: Sneath Similarity of Fas1 for WHO Critical Pathogens, cf. Figure S47

S2.5.2 Top 10 Agricultural Fungal Pathogens

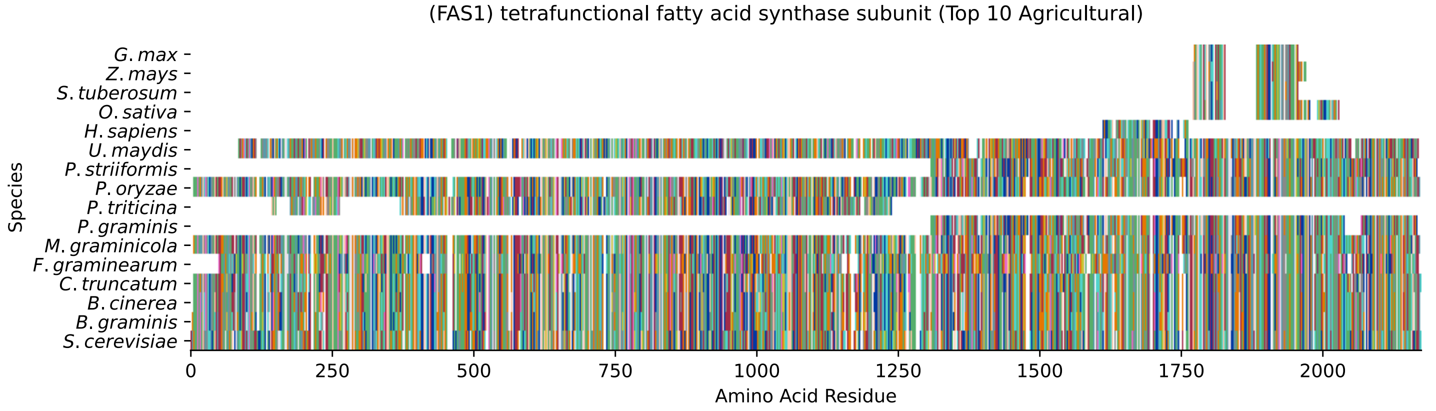

Figure S50: Multiple sequence alignment of yeast Fas1 (Top 10 Agricultural Fungal Pathogens). Cf. Figure S51 for alignment quality, and Figure S52 for Sneath similarity. Cf. Table S15 for protein names, and pairwise alignment metrics with yeast Fas1.

Fas1 MSA Quality

	B. g.	*B. c.*	*C. t.*	*F. g.*	*M. g.*	*P. g.*	*P. t.*	*P. o.*	*P. s.*	*U. m.*	*S. c.*
B. graminis	4277.63	3644.74	3292.67	1448.72	3231.81	729.939	728.783	3298.45	779.63	1353.96	2367.42
B. cinerea	3644.74	4262.6	3456.77	1434.08	3377.8	739.569	737.258	3434.81	790.415	1324.3	2409.41
C. truncatum	3303.84	3472.17	4263.76	1430.62	3163.24	734.561	726.472	3437.89	768.459	1333.16	2326.98
F. graminearum	1461.43	1444.1	1433.31	4100.05	1413.67	433.335	535.798	1403.65	434.491	968.763	1263.83
M. graminicola	3231.81	3377.8	3155.93	1400.19	4274.55	711.449	739.954	3121.26	776.548	1322.38	2353.56
P. graminis	729.939	739.569	734.176	432.95	711.449	1688.32	0	721.85	1579.69	517.694	670.233
P. triticina	736.102	745.347	737.643	536.954	752.281	0	1930.22	730.324	0	711.064	720.309
P. oryzae	3307.69	3446.37	3437.51	1402.5	3131.66	721.85	720.694	4271.46	770.385	1309.28	2352.01
P. striiformis	779.63	790.415	768.074	434.876	776.548	1579.69	0	770.385	1746.87	532.717	723.776
U. maydis	1353.96	1324.3	1327.38	959.133	1322.38	517.694	699.893	1302.35	532.717	4087.34	1247.26
S. cerevisiae	2376.28	2418.65	2328.9	1259.59	2357.02	670.618	720.309	2360.1	724.161	1250.34	4237.18

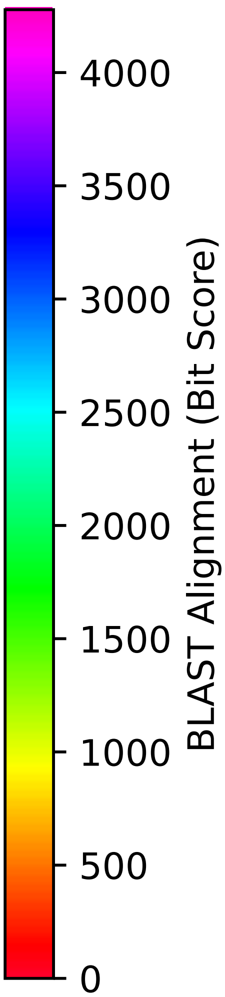

Figure S51: Multiple sequence alignment quality of Fas1 (Top 10 Agricultural Fungal Pathogens). Cf. Figure S50

Species	Hit Protein	Hit Length (a.a.)	evalue	align_len	bit_score	identity	positive	score	gaps	% identity	% positive
G.max	NP_001238312.2 malonyltransferase Glycine max	184	2.3e-05	184	50.0618	52	73	118	63	2.5	3.6
Z.mays	NP_001150334.1 uncharacterized protein LOC100283964 Zea mays	203	0.00035	203	46.2098	52	82	108	68	2.5	4.0
S.tuberosum	XP_006348452.1 PREDICTED: malonyl CoA-acyl carrier protein transacylase Solanum tuberosum	187	3.6e-05	187	48.521	52	74	114	63	2.5	3.6
O.sativa	XP_015628213.1 uncharacterized protein LOC4332547 Oryza sativa Japonica Group	256	0.00017	256	46.595	63	101	109	73	3.1	4.9
H.sapiens	NP_001278957.1 peroxisomal multifunctional enzyme type 2 isoform 5 Homo sapiens	133	2.1e-06	133	55.4546	40	64	132	9	2.0	3.1
U.maydis	XP_011392728.1 fatty acid synthase FAS2 Ustilago maydis 521	2052	0	2052	1249.57	742	1122	3232	159	36.2	54.7
P.striiformis	XP_047808761.1 hypothetical protein Pst134EA 009828 Puccinia striiformis f. sp. tritici	845	0	845	725.702	394	543	1872	32	19.2	26.5
P.oryzae	mRNA M BR32 EuGene 00072671-p1 — transcript=mRNA M BR32 EuGene 00072671 — gene=M BR32 EuGene 00072671 — organism=Pyricularia oryzae BR32 — gene product=unspecified product — transcript product=unspecified product — location=BR32 scaffold000-07:1168071-1174501(+) — protein length=2120 — sequence SO=supercontig — SO=protein coding gene — is pseudo=false	2074	0	2074	2357.79	1160	1502	6109	40	56.6	73.2
P.triticina	XP_053022848.1 uncharacterized protein PtA15 8A197 Puccinia triticina	979	0	979	722.235	406	556	1863	75	19.8	27.1
P.graminis	XP_003325251.2 fatty acid synthase subunit beta Puccinia graminis f. sp. tritici CRL 75-3-6-700-3	846	0	846	671.774	375	518	1732	62	18.3	25.3
M.graminicola	ZTRI 3.48.mRNA-p1 — transcript=ZTRI 3.48.mRNA — gene=ZTRI 3.48 — organism=Zymoseptoria tritici IPO323 — gene product=similar to fatty acid synthase subunit beta dehydratase — transcript product=similar to fatty acid synthase subunit beta dehydratase — location=Ztri chr 3:163778-170201(+) — protein length=2121 — sequence SO=chromosome — SO=protein coding gene — is pseudo=false	2068	0	2068	2360.49	1142	1498	6116	33	55.7	73.0
F.graminearum	XP_011315624.1 hypothetical protein FGSG 11656 Fusarium graminearum PH-1	2049	0	2049	1257.28	742	1121	3252	123	36.2	54.7
C.truncatum	XP_036581307.1 fatty acid synthase beta subunit dehydratase Colletotrichum truncatum	2074	0	2074	2330.06	1147	1490	6037	40	55.9	72.6
B.cinerea	XP_024545929.1 hypothetical protein BCIN 01g00450 Botrytis cinerea B05.10	2069	0	2069	2420.58	1174	1520	6272	34	57.2	74.1
B.graminis	VCU38897.1 — transcript=BGT96224V316 LOCUS150 t1 — gene=BGT96224V316 LOCUS150 — organism=Blumeria graminis f. sp. tritici 96224 — gene product=unspecified product — transcript product=unspecified product — location=LR026984:2368020-2374354(-) — protein length=2096 — sequence SO=chromosome — SO=protein coding gene — is pseudo=false	2072	0	2072	2378.21	1162	1508	6162	36	56.7	73.5

Table S15: Pairwise alignment info from yeast Fas1 (DEG20010641), cf. Figure S50.

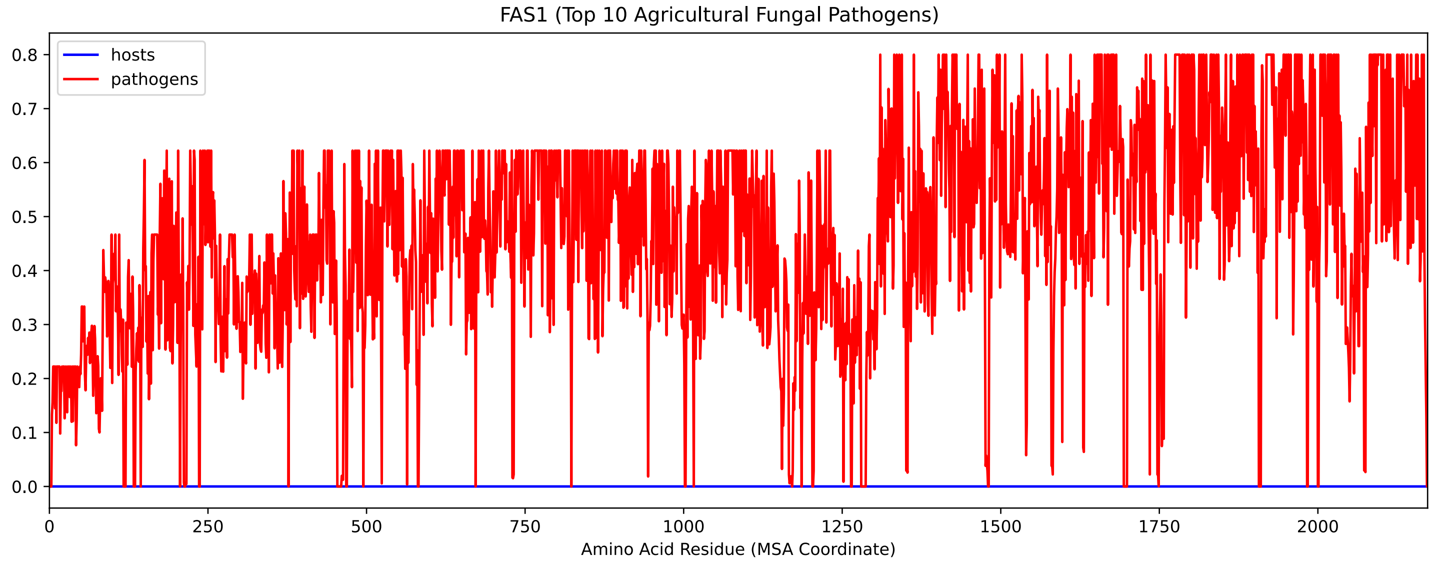

Figure S52: Sneath Similarity of Fas1 for Top 10 Agricultural Fungal Pathogens, cf. Figure S50

S2.5.3 NR

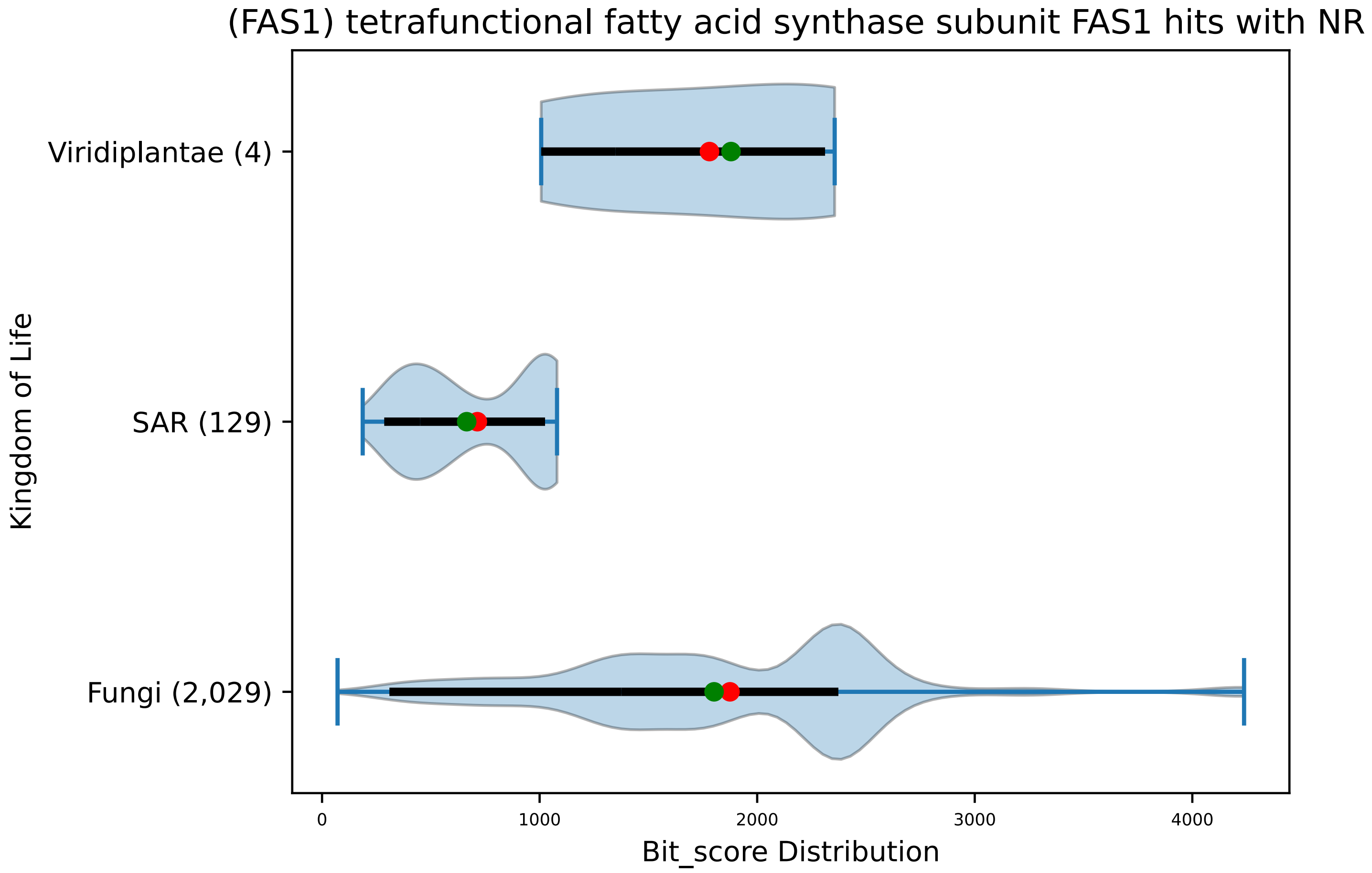

Figure S53: Non-redundant (NR) protein hits for DEG20010641/Fas1, with expectation value of no more than 0.1. Green points are medians, and red points are arithmetic means.

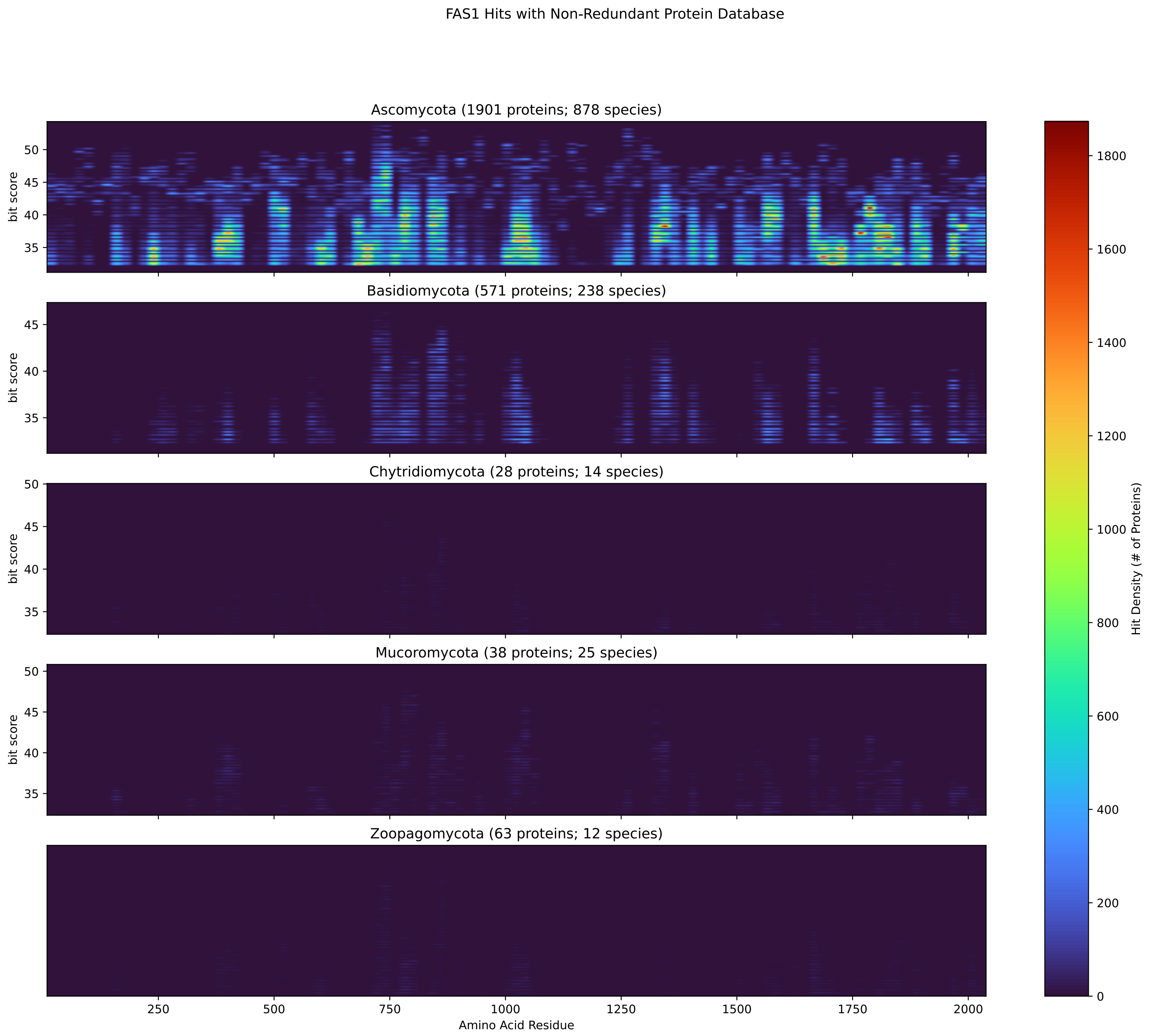

Figure S54: Non-redundant (NR) protein hits for Fas1 in the kingdom Fungi.

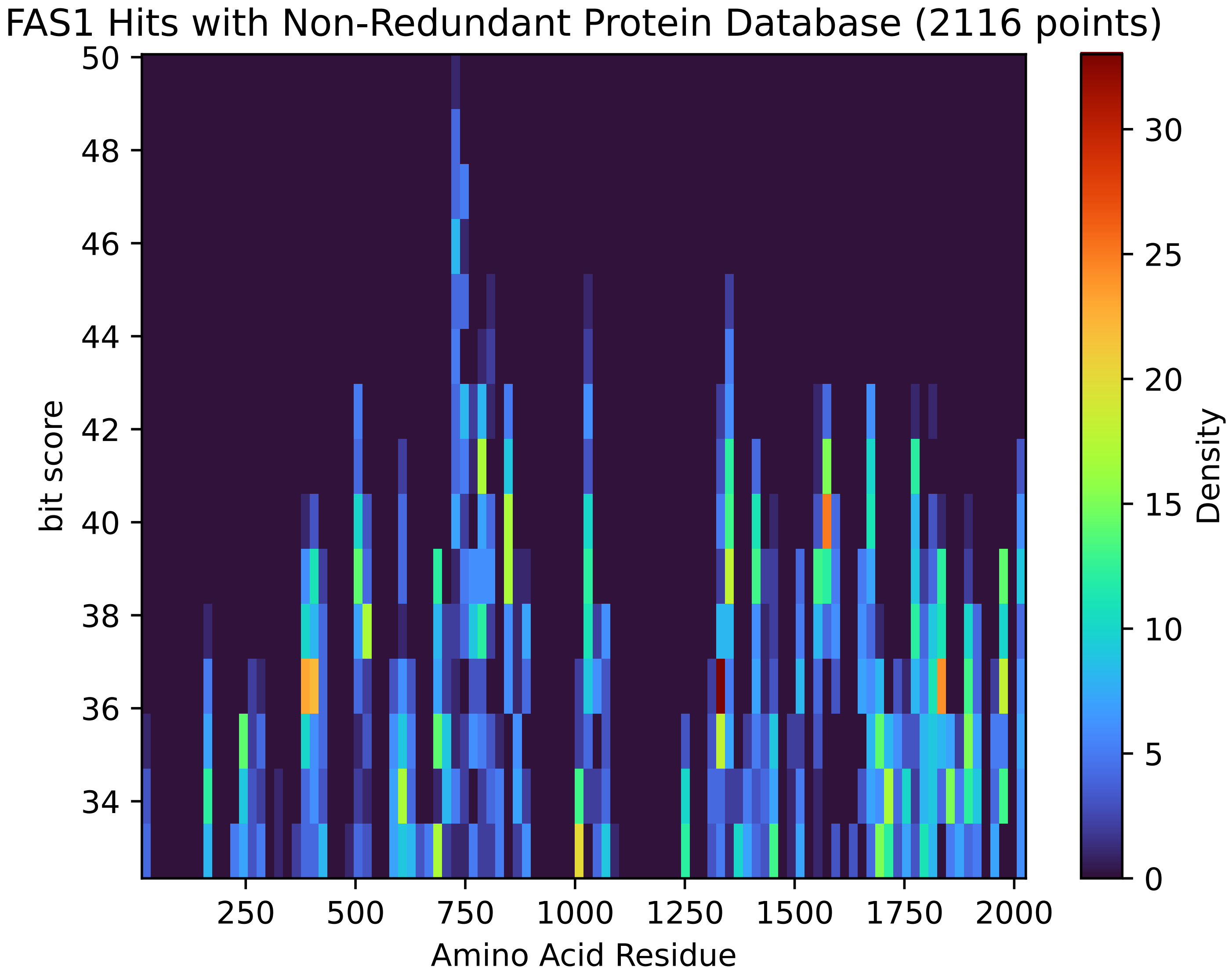

Figure S55: Non-redundant (NR) protein hits for Fas1 in the kingdom Viridiplantae.

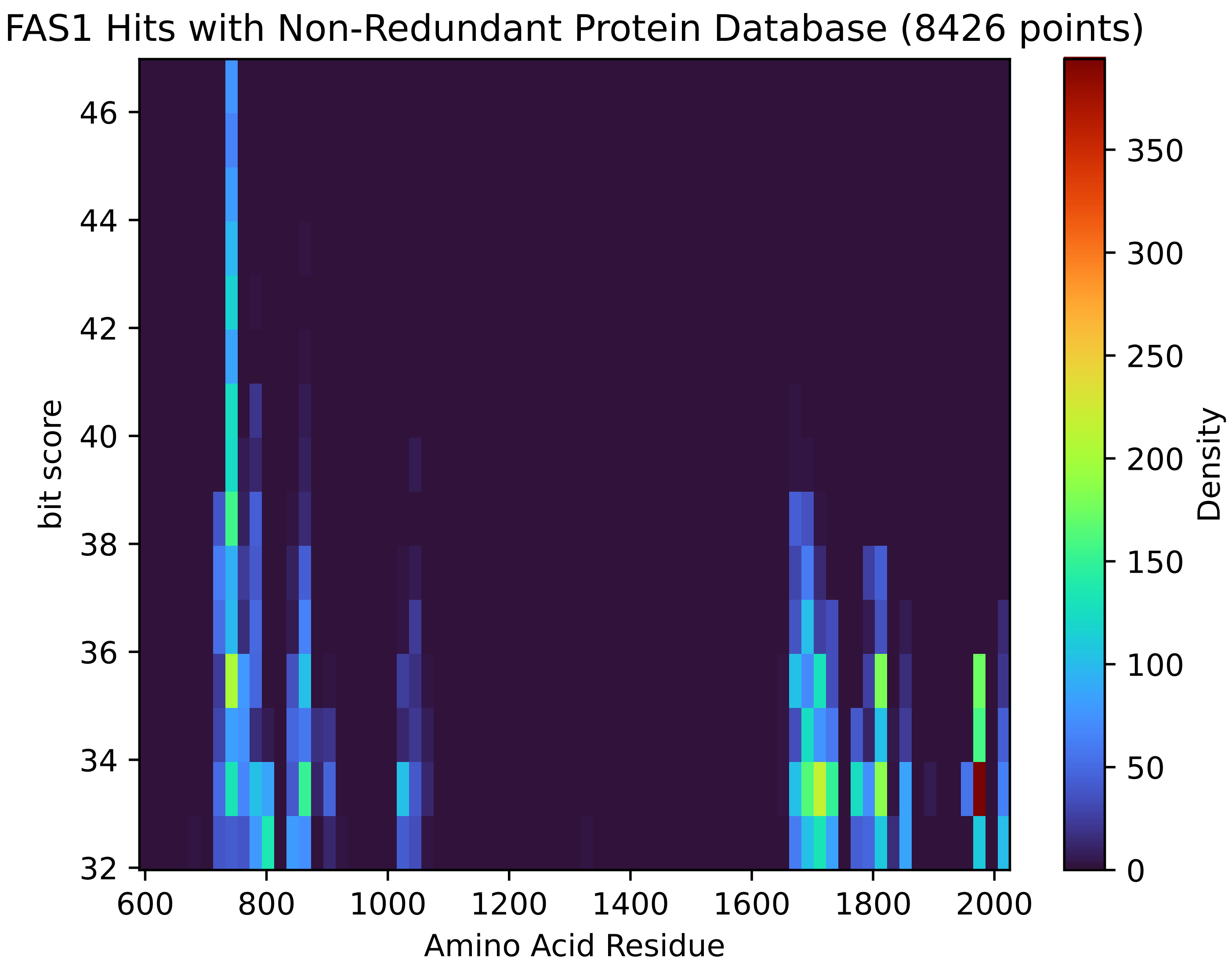

Figure S56: Non-redundant (NR) protein hits for Fas1 in the kingdom SAR.

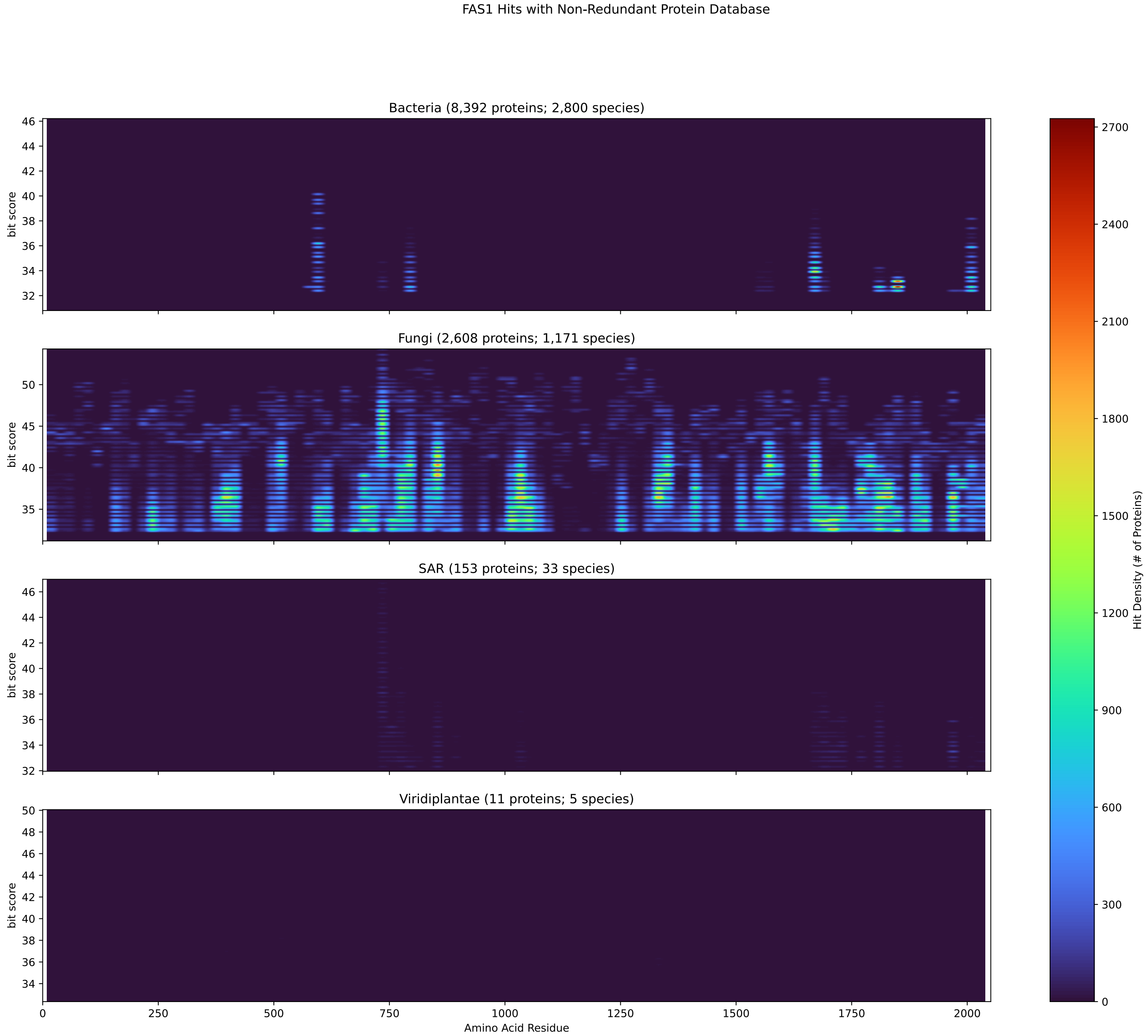

Figure S57: Non-redundant (NR) protein hits for DEG20010641/Fas1 at 20 amino acid length queries.

S2.6 Fas2

S2.6.1 WHO Critical Pathogens

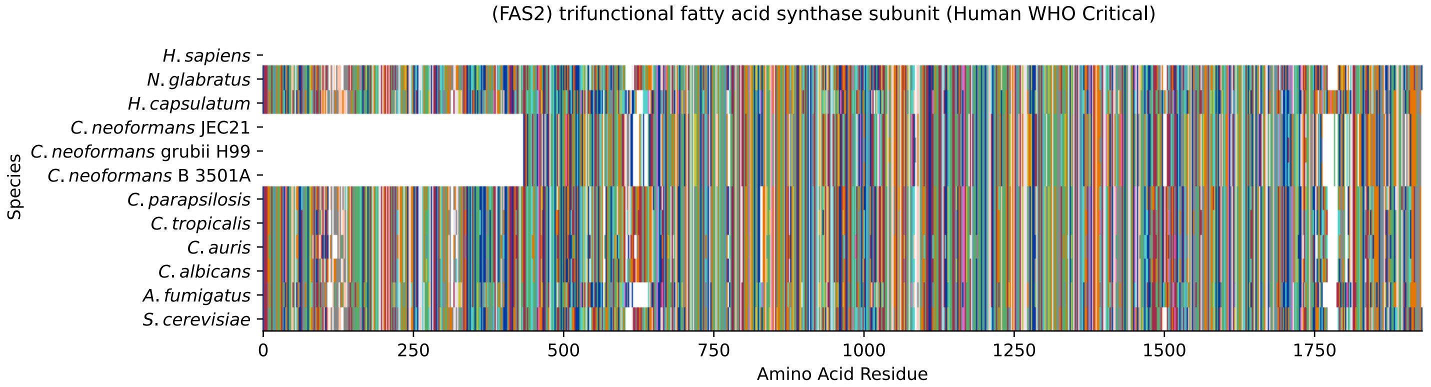

Figure S58: Multiple sequence alignment of yeast Fas2 (WHO Critical Pathogens). Cf. Figure S59 for alignment quality, and Figure S60 for Sneath similarity. Cf. Table S16 for protein names, and pairwise alignment metrics with yeast Fas2.

Fas2 MSA Quality

	A. f.	*C. a.*	*C. a.*	*C. p.*	*C. t.*	*C. n.*	*C. n.*	*C. n.*	*H. c.*	*N. g.*	*S. c.*
A. fumigatus	3842.74	2488.38	2437.53	2421.35	2487.22	1565.05	1565.44	1565.05	3117.41	2419.81	2377.44
C. albicans	2484.91	3889.73	3194.06	3346.98	3537.66	1434.47	1436.01	1434.47	2402.09	2714.49	2709.48
C. auris	2439.07	3210.62	3875.48	3163.24	3219.48	1464.9	1463.74	1464.9	2374.36	2674.04	2682.13
C. parapsilosis	2442.54	3374.72	3179.81	3895.89	3382.42	1432.54	1436.4	1432.54	2360.1	2663.64	2672.5
C. tropicalis	2479.52	3533.81	3200.99	3345.83	3892.81	1441.02	1442.17	1441.02	2407.48	2725.27	2714.87
C. neoformans B 3501A	1582.77	1451.42	1476.07	1437.17	1459.51	2954.08	2914.41	2954.08	1488.78	1442.17	1457.97
C. neoformans grubii H99	1582.39	1452.19	1472.6	1437.94	1459.12	2914.41	2949.84	2914.41	1490.32	1447.57	1455.66
C. neoformans JEC21	1582.77	1451.42	1476.07	1437.17	1459.51	2954.08	2914.41	2954.08	1488.78	1442.17	1457.97
H. capsulatum	3137.05	2430.21	2394.39	2360.1	2439.45	1504.58	1503.8	1504.58	3944.81	2335.84	2339.69
N. glabratus	2420.19	2728.35	2668.65	2645.15	2743.76	1422.14	1427.92	1422.14	2313.88	3887.42	3219.1
S. cerevisiae	2385.91	2712.95	2671.73	2649.77	2722.58	1440.25	1436.78	1440.25	2321.2	3225.65	3890.5

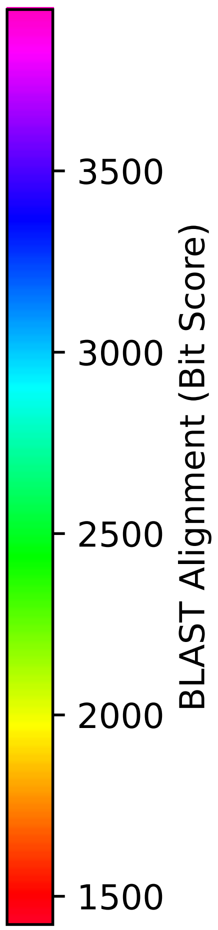

Figure S59: Multiple sequence alignment quality of Fas2 (WHO Critical Pathogens). Cf. Figure S58

Species	Hit Protein	Hit Length (a.a.)	evalue	align_len	bit_score	identity	positive	score	gaps	% identity	% positive
H.sapiens	-	-	-	-	-	-	-	-	-	-	-
N.glabratus	XP_445956.1 uncharacterized p-rotein CAGL0E06138g Nakaseomyc-es glabratus	1886	0	1886	3224.49	1554	1723	8359	5	82.4	91.3
H.capsulatum	XP_045283904.1 fatty acid syn-thase subunit alpha Histoplasm-a capsulatum G186AR	1916	0	1916	2321.2	1158	1460	6014	48	61.4	77.4
C.neoformans.JEC21	XP_571099.1 fatty-acid syntha-se complex protein, putative C-ryptococcus neoformans var. ne-oformans JEC21	1469	0	1469	1441.02	736	989	3729	55	39.0	52.4
C.neoformans.grubii.H99	XP_012049943.1 fatty acid syn-thase subunit alpha, fungi typ-e Cryptococcus neoformans var. grubii H99	1463	0	1463	1436.78	736	985	3718	43	39.0	52.2
C.neoformans.B.3501A	XP_775163.1 hypothetical prot-ein CNBE4360 Cryptococcus neof-ormans var. neoformans B-3501A	1469	0	1469	1441.02	736	989	3729	55	39.0	52.4
C.parapsilosis	XP_036665363.1 uncharacterize-d protein CPAR2 807400 Candida parapsilosis	1900	0	1900	2651.31	1294	1565	6871	34	68.6	82.9
C.tropicalis	XP_002548204.1 fatty acid syn-thase alpha subunit Candida tr-opicalis MYA-3404	1899	0	1899	2721.42	1319	1584	7053	33	69.9	83.9
C.auris	XP_028891558.2 fatty acid syn-thase subunit alpha Candida au-ris	1898	0	1898	2671.73	1311	1567	6924	38	69.5	83.0
C.albicans	XP_723161.2 trifunctional fat-ty acid synthase subunit Candi-da albicans SC5314	1900	0	1900	2712.95	1313	1588	7031	34	69.6	84.2
A.fumigatus	XP_748738.1 fatty acid syntha-se alpha subunit FasA Aspergil-lus fumigatus Af293	1894	0	1894	2387.07	1167	1470	6185	49	61.8	77.9

Table S16: Pairwise alignment info from yeast Fas2 (DEG20011054), cf. Figure S58.

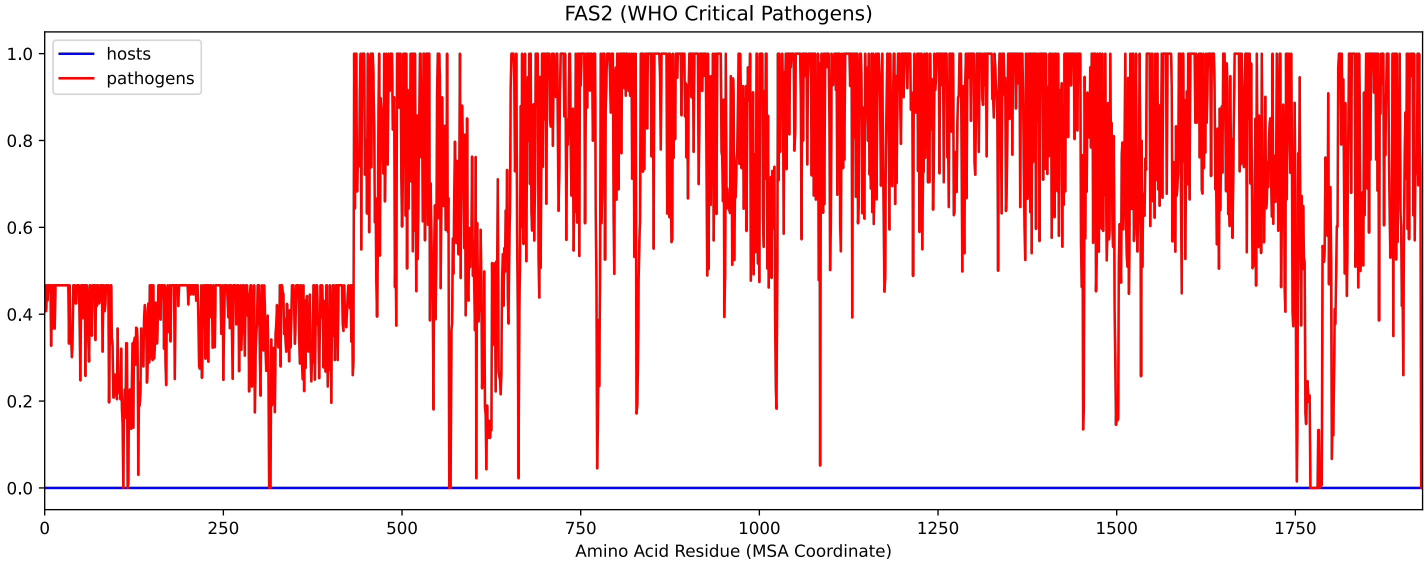

Figure S60: Sneath Similarity of Fas2 for WHO Critical Pathogens, cf. Figure S58

S2.6.2 Top 10 Agricultural Fungal Pathogens

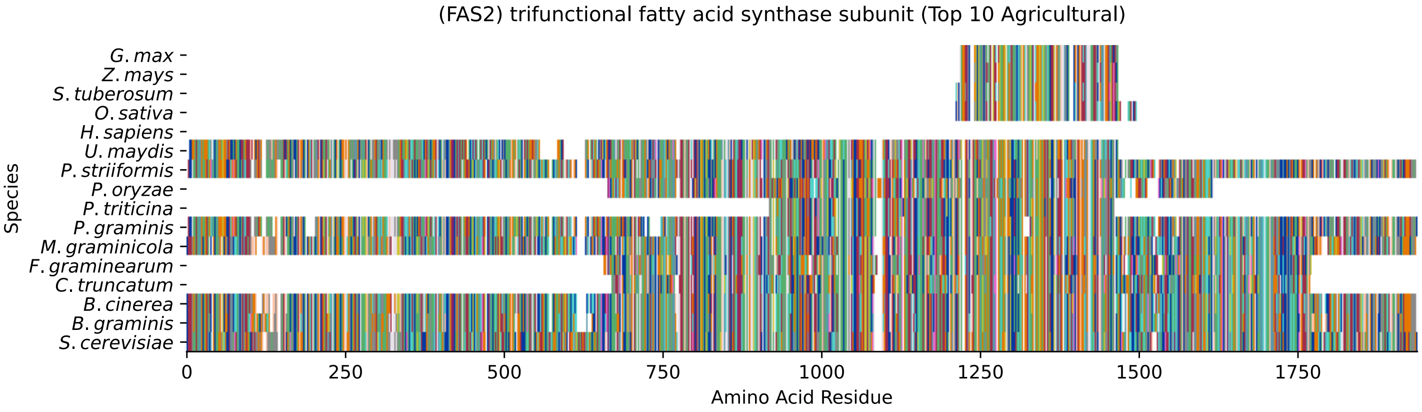

Figure S61: Multiple sequence alignment of yeast Fas2 (Top 10 Agricultural Fungal Pathogens). Cf. Figure S62 for alignment quality, and Figure S63 for Sneath similarity. Cf. Table S17 for protein names, and pairwise alignment metrics with yeast Fas2.

Fas2 MSA Quality

	B. g.	*B. c.*	*C. t.*	*F. g.*	*M. g.*	*P. g.*	*P. t.*	*P. o.*	*P. s.*	*U. m.*	*S. c.*
B. graminis	3860.07	3311.16	1193.33	1192.18	3006.08	1768.05	615.92	863.603	1937.15	1090.1	2399.78
B. cinerea	3341.21	3853.52	1193.33	1191.79	3124.72	1769.59	613.224	878.241	1947.55	1067.37	2422.51
C. truncatum	1216.45	1215.29	2209.88	1026.16	1218.37	903.664	483.411	800.431	983.4	703.36	1118.61
F. graminearum	1202.19	1201.04	1010.75	2237.61	1181.01	895.575	495.352	769.615	980.704	687.182	1130.16
M. graminicola	3057.31	3151.69	1206.43	1177.93	3860.07	1794.63	613.224	872.848	1964.5	1070.07	2330.44
P. graminis	1794.63	1776.91	883.634	887.486	1793.09	3749.52	988.408	650.203	3353.53	1013.45	1649.41
P. triticina	615.92	613.609	483.026	495.738	613.224	988.023	1081.24	392.504	1030.01	554.288	600.127
P. oryzae	887.1	900.968	801.971	788.875	895.96	670.233	412.535	1855.11	761.911	639.802	852.432
P. striiformis	1976.44	1962.96	987.252	993.03	1970.67	3369.33	1030.01	758.444	3826.56	1149.42	1813.12
U. maydis	1109.75	1074.31	684.871	687.567	1074.69	1019.99	555.058	619.387	1129.78	2762.64	1056.59
S. cerevisiae	2450.63	2455.63	1100.12	1124	2352.01	1661.74	601.282	830.861	1813.51	1070.84	3890.5

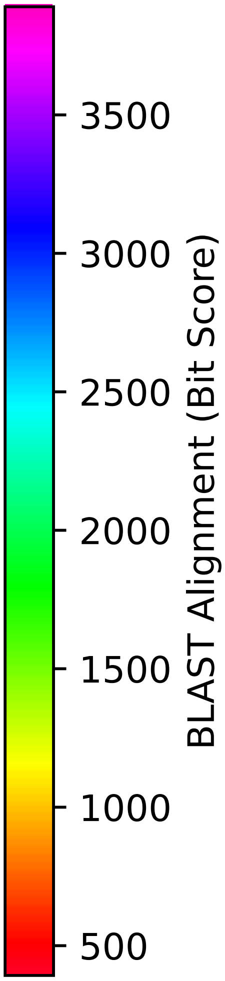

Figure S62: Multiple sequence alignment quality of Fas2 (Top 10 Agricultural Fungal Pathogens). Cf. Figure S61

Species	Hit Protein	Hit Length (a.a.)	evalue	align_len	bit_score	identity	positive	score	gaps	% identity	% positive
G.max	NP_001236069.1 plastid 3-keto--acyl-ACP synthase II-B precur-sor Glycine max	255	1.8e-06	255	53.9138	67	111	128	34	3.6	5.9
Z.mays	NP_001169617.1 3-oxoacyl-acyl--carrier-protein synthase II, chloroplastic-like precursor Z-ea mays	255	1e-05	255	51.2174	61	106	121	34	3.2	5.6
S.tuberosum	XP_006345248.1 PREDICTED: 3-o-xoacyl-acyl-carrier-protein sy-nthase II, chloroplastic-like Solanum tuberosum	254	4.2e-07	254	55.0694	65	112	131	22	3.4	5.9
O.sativa	XP_015630630.1 3-oxoacyl-acyl--carrier-protein synthase II, chloroplastic-like Oryza sativ-a Japonica Group	278	1.1e-05	278	50.8322	63	112	120	34	3.3	5.9
H.sapiens	-	-	-	-	-	-	-	-	-	-	-
U.maydis	XP_011392728.1 fatty acid syn-thase FAS2 Ustilago maydis 521	1432	0	1432	1070.07	601	851	2766	91	31.8	45.1
P.striiformis	XP_047808761.1 hypothetical p-rotein Pst134EA 009828 Puccini-a striiformis f. sp. tritici	1894	0	1894	1812.74	947	1279	4694	54	50.2	67.8
P.oryzae	mRNA M BR32 EuGene 00106401-p1 — transcript=mRNA M BR32 EuGe-ne 00106401 — gene=M BR32 EuGe-ne 00106401 — organism=Pyricul-aria oryzae BR32 — gene produc-t=unspecified product — transc-ript product=unspecified produ-ct — location=BR32 scaffold000-14:1001674-1005232(-) — protei-n length=1167 — sequence SO=su-percontig — SO=protein coding gene — is pseudo=false	937	0	937	833.558	426	617	2152	50	22.6	32.7
P.triticina	XP_053019002.1 uncharacterize-d protein PtA15 3A818 Puccinia triticina	524	0	524	601.282	289	388	1549	7	15.3	20.6
P.graminis	XP_003325251.2 fatty acid syn-thase subunit beta Puccinia gr-aminis f. sp. tritici CRL 75-3-6-700-3	1899	0	1899	1659.43	902	1225	4296	105	47.8	64.9
M.graminicola	ZTRI 3.47.mRNA-p1 — transcript-=ZTRI 3.47.mRNA — gene=ZTRI 3.-47 — organism=Zymoseptoria tri-tici IPO323 — gene product=sim-ilar to fatty acid synthase su-bunit alpha — transcript produ-ct=similar to fatty acid synth-ase subunit alpha — location=Z-tri chr 3:156668-162355(-) — p-rotein length=1859 — sequence SO=chromosome — SO=protein cod-ing gene — is pseudo=false	1902	0	1902	2352.01	1149	1459	6094	63	60.9	77.3
F.graminearum	XP_011315622.1 hypothetical p-rotein FGSG 00036 Fusarium gra-minearum PH-1	1091	0	1091	1127.46	548	761	2915	22	29.0	40.3
C.truncatum	XP_036575084.1 fatty acid syn-thase subunit alpha reductase Colletotrichum truncatum	1080	0	1080	1102.04	540	762	2849	25	28.6	40.4
B.cinerea	XP_024545928.1 Bcfas2 Botryti-s cinerea B05.10	1899	0	1899	2455.63	1188	1483	6363	56	63.0	78.6
B.graminis	VCU38898.1 — transcript=BGT962-24V316 LOCUS151 t1 — gene=BGT9-6224V316 LOCUS151 — organism=B-lumeria graminis f. sp. tritic-i 96224 — gene product=unspeci-fied product — transcript prod-uct=unspecified product — loca-tion=LR026984:2375831-2381518(-+) — protein length=1864 — seq-uence SO=chromosome — SO=prote-in coding gene — is pseudo=fal-se	1904	0	1904	2449.86	1186	1482	6348	60	62.9	78.5

Table S17: Pairwise alignment info from yeast Fas2 (DEG20011054), cf. Figure S61.

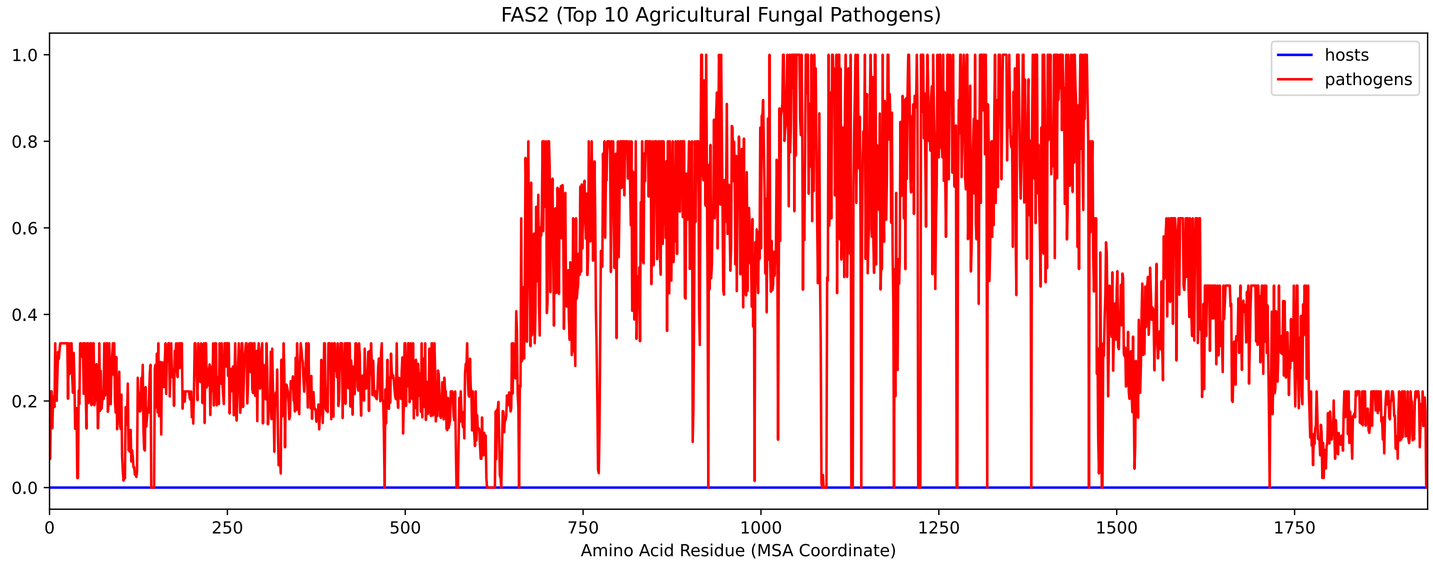

Figure S63: Sneath Similarity of Fas2 for Top 10 Agricultural Fungal Pathogens, cf. Figure S61

S2.6.3 NR

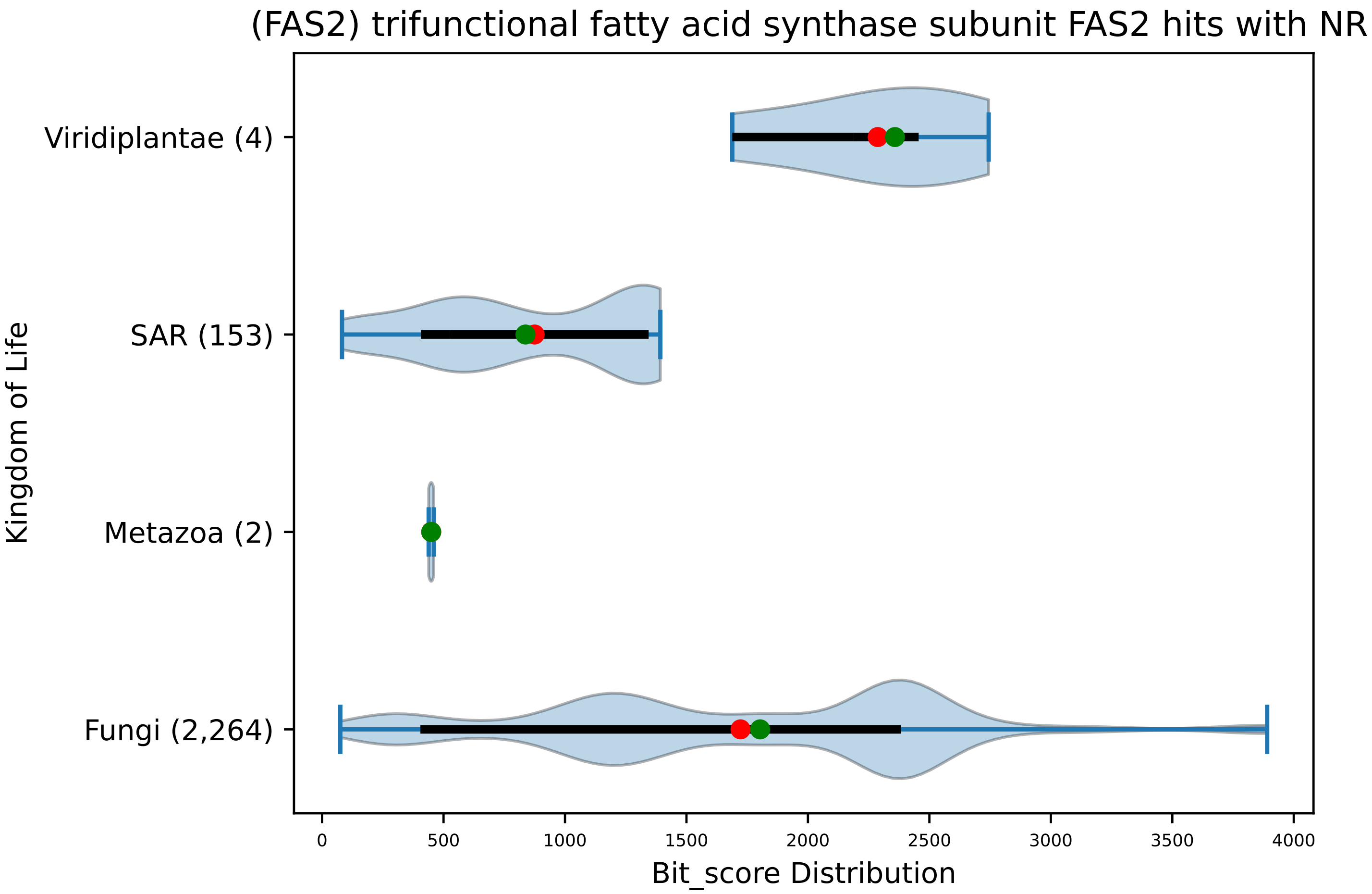

Figure S64: Non-redundant (NR) protein hits for DEG20011054/Fas2, with expectation value of no more than 0.1. Green points are medians, and red points are arithmetic means.

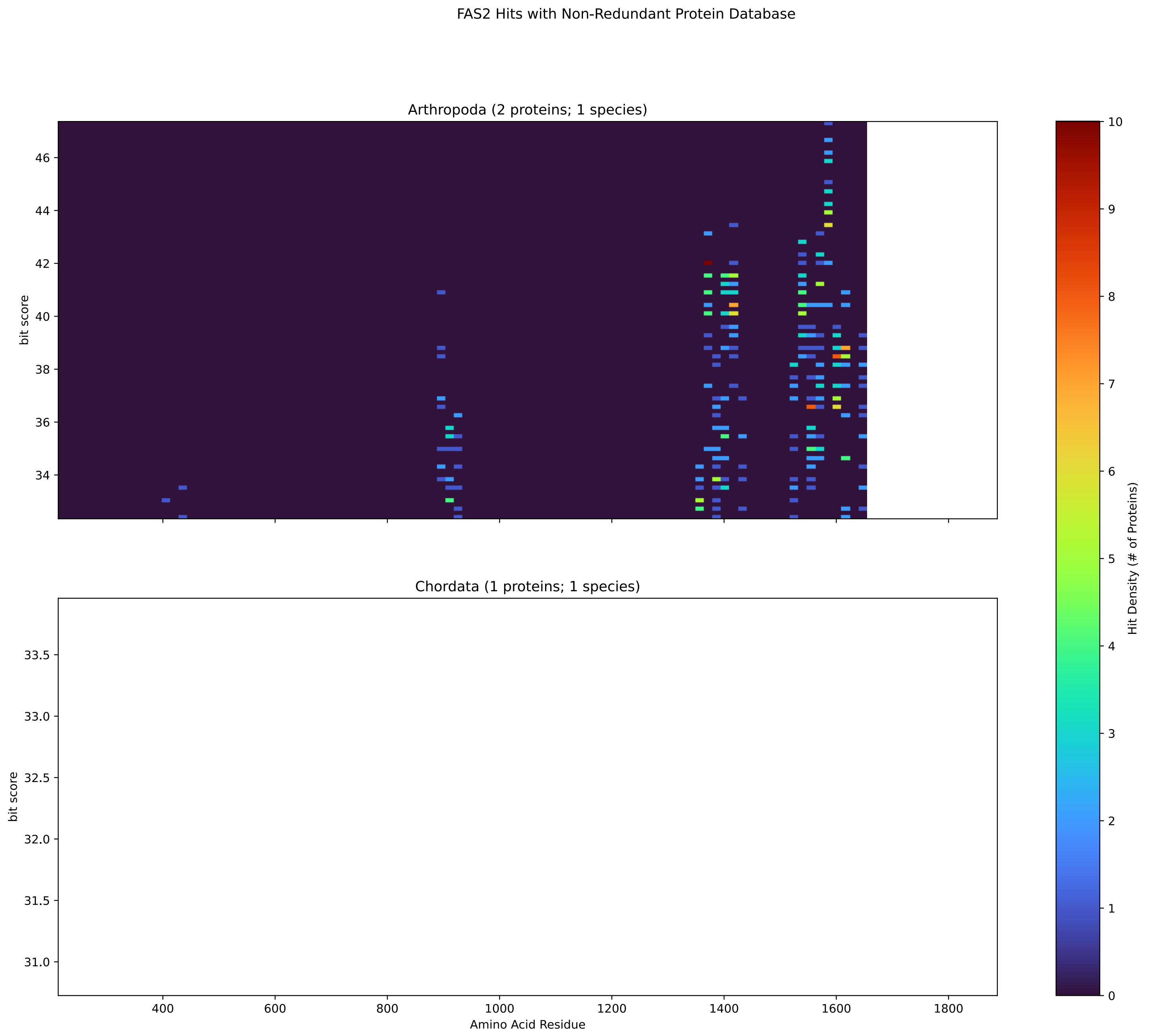

Figure S65: Non-redundant (NR) protein hits for Fas2 in the kingdom Metazoa.

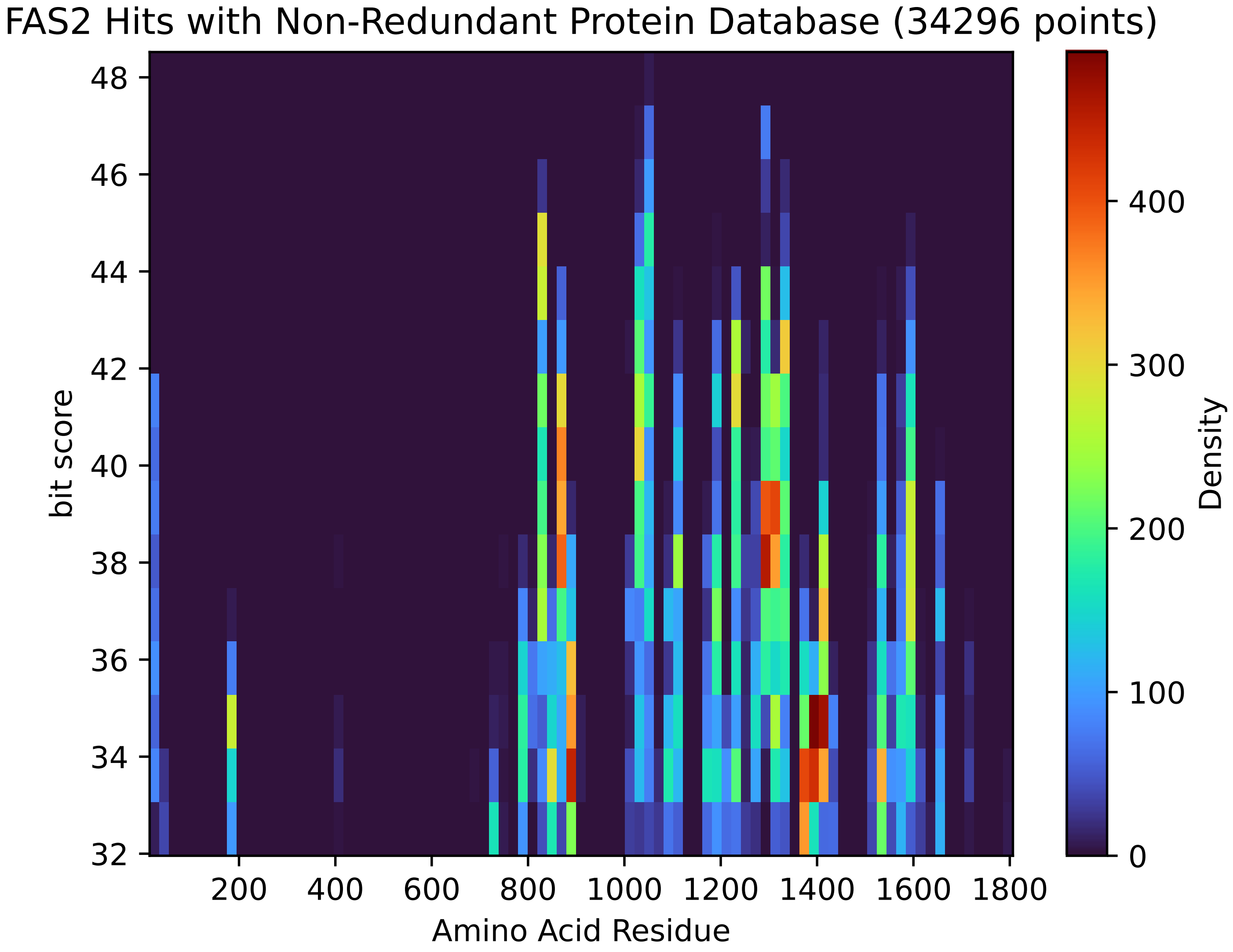

Figure S66: Non-redundant (NR) protein hits for Fas2 in the kingdom SAR.

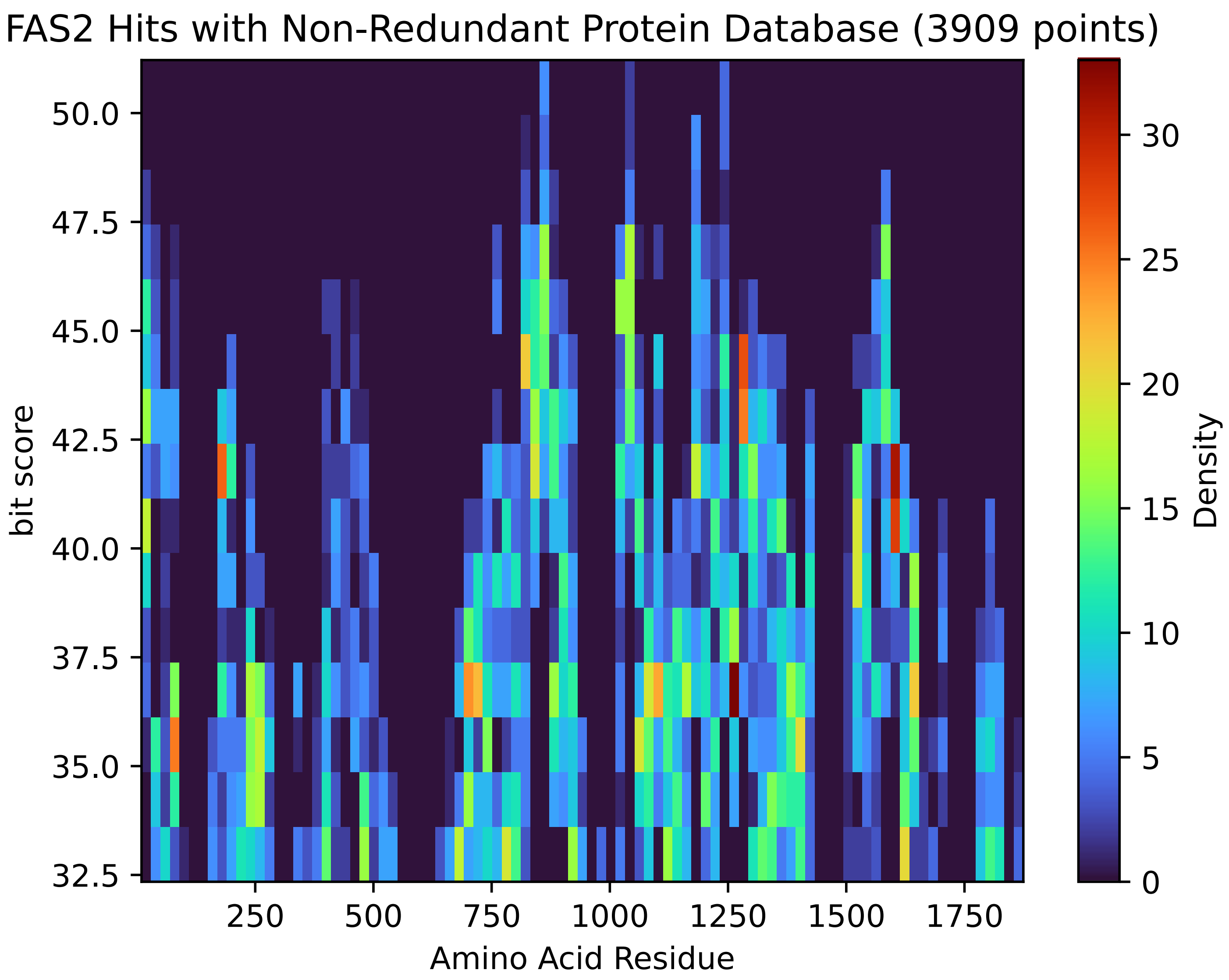

Figure S67: Non-redundant (NR) protein hits for Fas2 in the kingdom Viridiplantae.

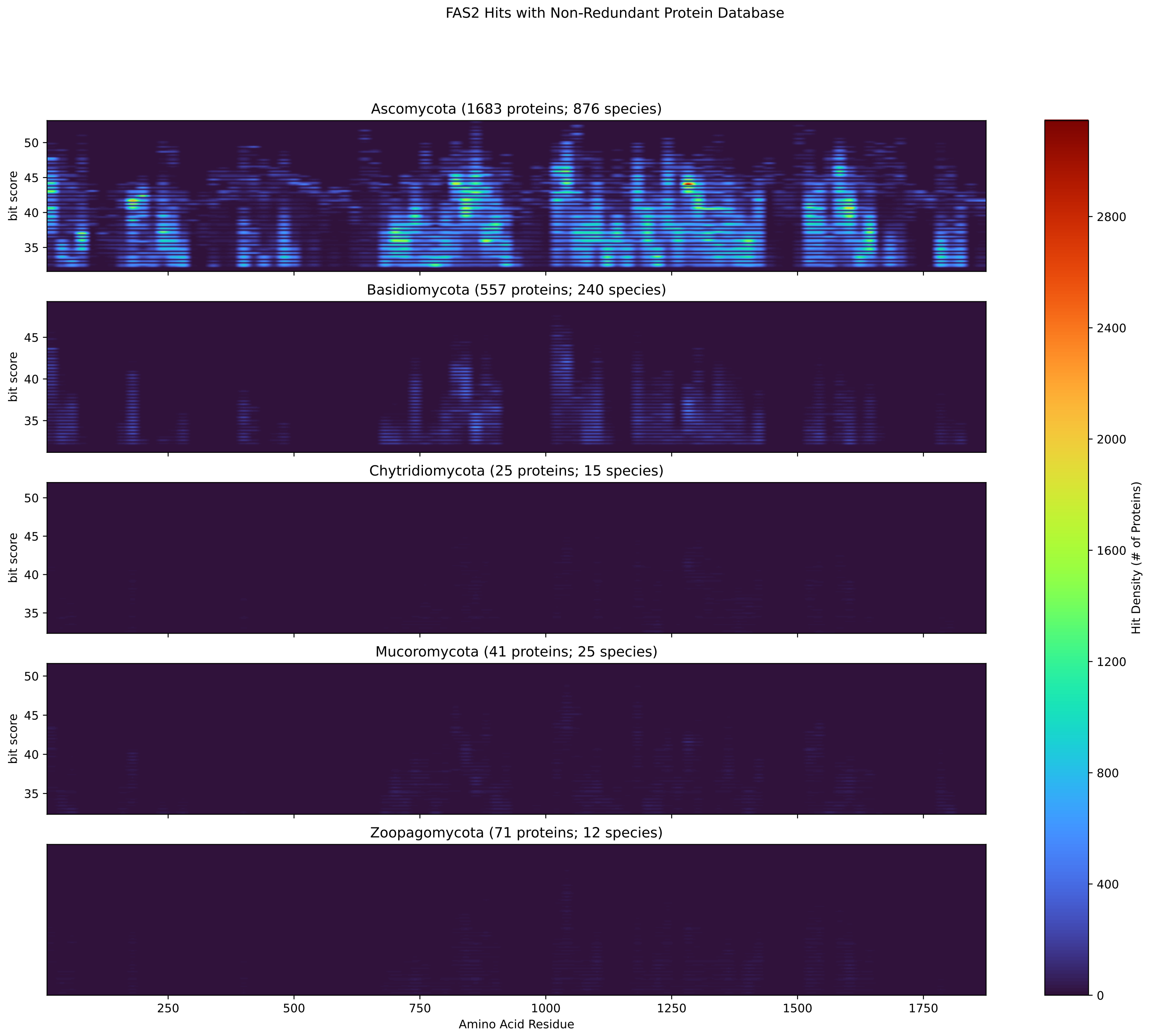

Figure S68: Non-redundant (NR) protein hits for Fas2 in the kingdom Fungi.

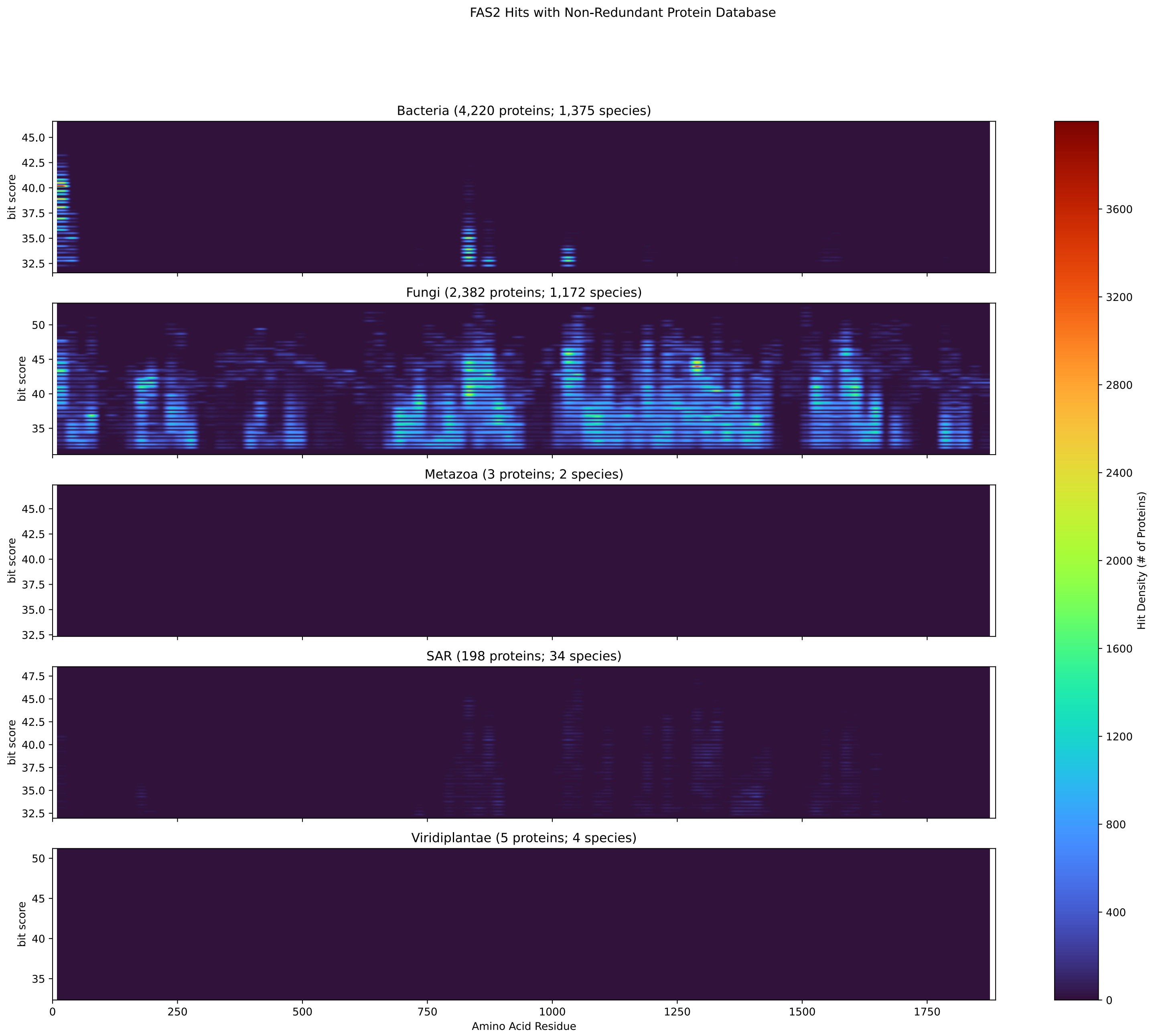

Figure S69: Non-redundant (NR) protein hits for DEG20011054/Fas2 at 20 amino acid length queries.

S2.7 Fba1

S2.7.1 WHO Critical Pathogens

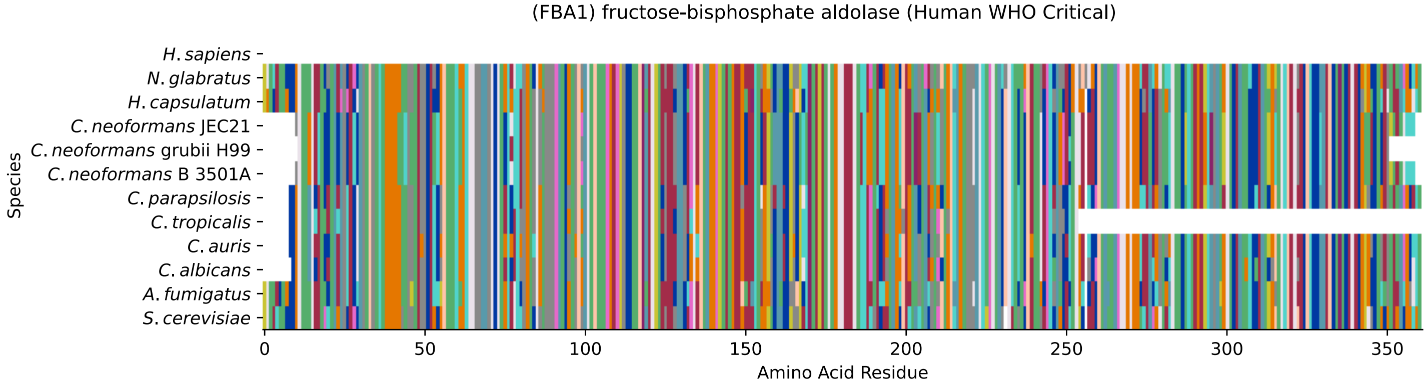

Figure S70: Multiple sequence alignment of yeast Fba1 (WHO Critical Pathogens). Cf. Figure S71 for alignment quality, and Figure S72 for Sneath similarity. Cf. Table S18 for protein names, and pairwise alignment metrics with yeast Fba1.

Fba1 MSA Quality

	A. f.	*C. a.*	*C. a.*	*C. p.*	*C. t.*	*C. n.*	*C. n.*	*C. n.*	*H. c.*	*N. g.*	*S. c.*
A. fumigatus	746.503	515.383	555.444	512.686	363.999	498.819	502.286	498.819	628.246	557.37	530.02
C. albicans	516.153	728.013	650.588	642.499	450.284	481.1	482.256	481.1	506.908	590.497	548.51
C. auris	536.569	631.328	733.791	624.78	427.172	502.671	505.753	502.671	526.939	609.372	572.007
C. parapsilosis	512.686	641.343	643.654	730.324	434.106	493.812	494.967	493.812	512.301	579.326	552.747
C. tropicalis	365.54	451.44	446.047	435.647	508.449	348.591	351.673	348.591	357.451	413.69	394.045
C. neoformans B 3501A	499.204	480.33	519.235	493.812	347.051	719.153	687.952	719.153	504.212	520.39	508.834
C. neoformans grubii H99	502.671	481.485	522.316	494.197	350.132	687.952	701.434	687.952	499.975	523.472	514.227
C. neoformans JEC21	499.204	480.33	519.235	493.812	347.051	719.153	687.952	719.153	504.212	520.39	508.834
H. capsulatum	629.017	506.908	542.347	512.686	356.681	504.982	500.745	504.982	742.265	543.117	526.168
N. glabratus	540.036	572.392	609.372	561.992	395.971	504.212	507.679	504.212	528.094	749.969	631.328
S. cerevisiae	512.686	531.176	572.007	535.413	376.711	491.115	496.123	491.115	509.22	631.328	749.584

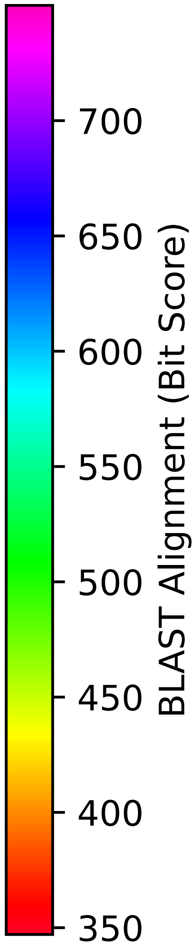

Figure S71: Multiple sequence alignment quality of Fba1 (WHO Critical Pathogens). Cf. Figure S70

Species	Hit Protein	Hit Length (a.a.)	evalue	align_len	bit_score	identity	positive	score	gaps	% identity	% positive
H.sapiens	-	-	-	-	-	-	-	-	-	-	-
N.glabratus	XP_448879.1 uncharacterized p-rotein CAGL0L02497g Nakaseomyc-es glabratus	361	0	361	631.328	300	332	1627	2	83.6	92.5
H.capsulatum	XP_045285275.1 fructose 1,6-b-iphosphate aldolase Histoplasm-a capsulatum G186AR	360	0	360	509.22	246	288	1310	1	68.5	80.2
C.neoformans.JEC21	XP_568771.1 fructose-bisphosp-hate aldolase, putative Crypto-coccus neoformans var. neoform-ans JEC21	348	5e-176	348	491.5	237	284	1264	1	66.0	79.1
C.neoformans.grubii.H99	XP_012047721.1 fructose-bisph-osphate aldolase 1 Cryptococcu-s neoformans var. grubii H99	339	8.6e-178	339	496.123	238	284	1276	1	66.3	79.1
C.neoformans.B.3501A	XP_777012.1 hypothetical prot-ein CNBB5380 Cryptococcus neof-ormans var. neoformans B-3501A	348	4.8e-176	348	491.5	237	284	1264	1	66.0	79.1
C.parapsilosis	XP_036667483.1 uncharacterize-d protein CPAR2 401230 Candida parapsilosis	352	0	352	535.413	257	299	1378	1	71.6	83.3
C.tropicalis	XP_002545430.1 fructose-bisph-osphate aldolase Candida tropi-calis MYA-3404	246	9.7e-133	246	379.793	185	215	974	1	51.5	59.9
C.auris	XP_028888316.1 fructose-bisph-osphate aldolase Candida auris	352	0	352	572.007	263	310	1473	1	73.3	86.4
C.albicans	XP_722690.1 fructose-bisphosp-hate aldolase Candida albicans SC5314	351	0	351	531.176	257	300	1367	1	71.6	83.6
A.fumigatus	XP_754452.1 fructose-bisphosp-hate aldolase, class II Asperg-illus fumigatus Af293	360	0	360	512.686	246	290	1319	1	68.5	80.8

Table S18: Pairwise alignment info from yeast Fba1 (DEG20010617), cf. Figure S70.

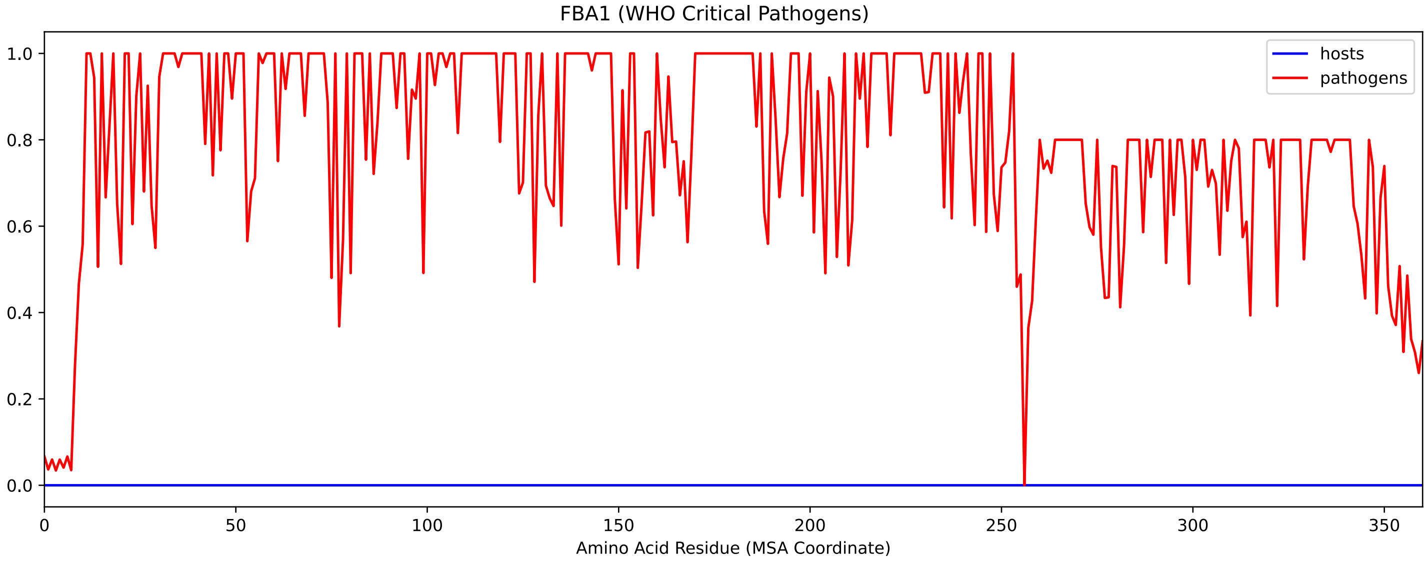

Figure S72: Sneath Similarity of Fba1 for WHO Critical Pathogens, cf. Figure S70

S2.7.2 Top 10 Agricultural Fungal Pathogens

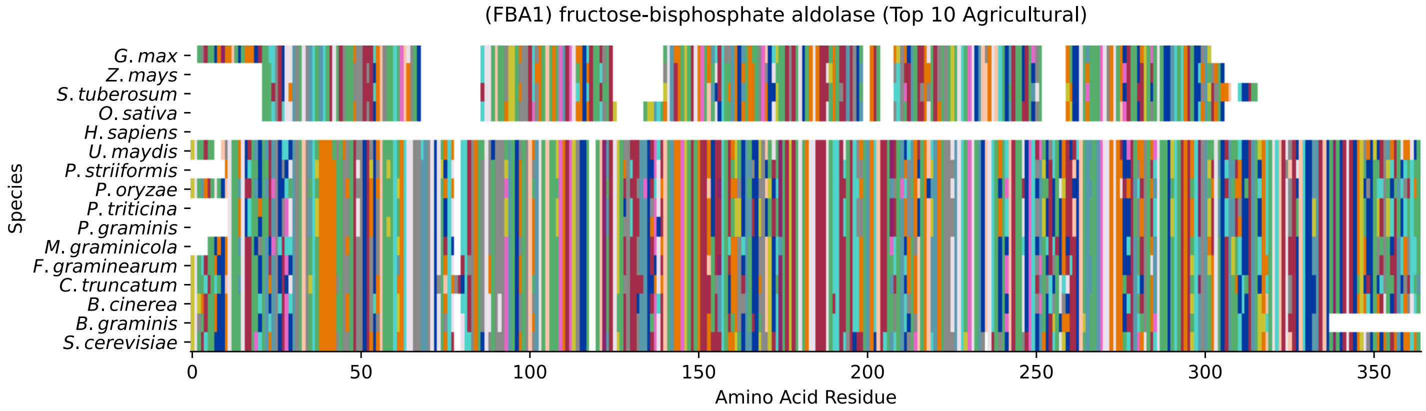

Figure S73: Multiple sequence alignment of yeast Fba1 (Top 10 Agricultural Fungal Pathogens). Cf. Figure S74 for alignment quality, and Figure S75 for Sneath similarity. Cf. Table S19 for protein names, and pairwise alignment metrics with yeast Fba1.

Fba1 MSA Quality

	B. g.	B. c.	C. t.	F. g.	M. g.	P. g.	P. t.	P. o.	P. s.	U. m.	S. c.
B. graminis	685.256	585.489	499.59	579.326	536.954	453.366	454.907	516.538	449.129	494.582	500.745
B. cinerea	585.489	744.191	543.888	628.246	579.711	471.855	473.781	540.806	464.922	520.776	534.258
C. truncatum	516.153	560.066	745.347	556.214	519.235	466.463	465.692	557.37	463.381	495.738	486.108
F. graminearum	579.326	628.246	536.954	746.117	596.275	486.108	489.574	556.984	484.182	521.161	524.628
M. graminicola	554.673	596.66	518.85	615.15	734.561	466.077	470.7	558.91	470.315	509.605	525.783
P. graminis	470.315	488.804	466.463	504.982	466.077	729.554	713.761	458.373	671.003	547.354	478.789
P. triticina	472.626	490.73	465.692	508.449	471.085	713.761	728.013	458.373	663.685	540.421	484.182
P. oryzae	533.487	558.14	556.984	575.859	558.91	458.373	457.988	746.503	454.521	501.13	503.827
P. striiformis	466.077	482.256	462.611	503.827	470.7	671.003	663.685	454.907	729.939	544.658	483.026
U. maydis	494.582	520.776	481.485	521.161	494.582	531.561	525.013	485.337	529.635	744.577	501.13
S. cerevisiae	500.745	534.258	469.929	524.628	508.064	461.455	466.077	485.723	464.537	501.13	749.584

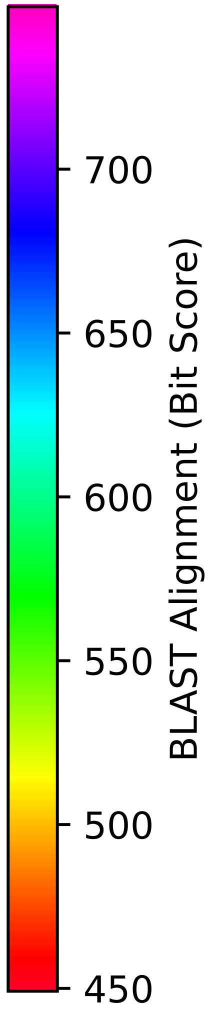

Figure S74: Multiple sequence alignment quality of Fba1 (Top 10 Agricultural Fungal Pathogens). Cf. Figure S73

Species	Hit Protein	Hit Length (a.a.)	evalue	align_len	bit_score	identity	positive	score	gaps	% identity	% positive
G.max	XP_003530061.1 uncharacterized protein LOC100779987 isoform X1 Glycine max	297	3.2e-09	297	60.077	76	123	144	45	21.2	34.3
Z.mays	NP_001333690.1 uncharacterized protein LOC100280420 Zea mays	281	4e-06	281	49.6766	74	113	117	43	20.6	31.5
S.tuberosum	XP_006341517.1 PREDICTED: uncharacterized protein LOC102593631 Solanum tuberosum	292	6.5e-07	292	51.6026	80	118	122	47	22.3	32.9
O.sativa	XP_015642840.1 uncharacterized protein LOC4340684 isoform X1 Oryza sativa Japonica Group	285	2.5e-07	285	53.1434	77	116	126	44	21.4	32.3
H.sapiens	-	-	-	-	-	-	-	-	-	-	-
U.maydis	XP_011386477.1 putative fructose-bisphosphate aldolase FBA1 Ustilago maydis 521	360	8.2e-180	360	501.13	231	287	1289	3	64.3	79.9
P.striiformis	XP_047806328.1 hypothetical protein Pst134EA 013487 Puccinia striiformis f. sp. tritici	350	2.2e-164	350	464.151	218	279	1193	1	60.7	77.7
P.oryzae	mRNA M BR32 EuGene 00018841-p1 — transcript=mRNA M BR32 EuGene 00018841 — gene=M BR32 EuGene 00018841 — organism=Pyricularia oryzae BR32 — gene product=unspecified product — transcript product=unspecified product — location=BR32 scaffold000-02:792025-793370(-) — protein length=360 — sequence SO=supercontig — SO=protein coding gene — is pseudo=false	360	1.5e-173	360	485.723	238	290	1249	1	66.3	80.8
P.triticina	XP_053028624.1 uncharacterized protein PtA15 18A126 Puccinia triticina	349	1.2e-165	349	467.233	221	277	1201	1	61.6	77.2
P.graminis	XP_003320224.1 fructose-bisphosphate aldolase, class II Puccinia graminis f. sp. tritici CRL 75-36-700-3	349	6.8e-164	349	461.455	218	276	1186	1	60.7	76.9
M.graminicola	ZTRI 2.441.mRNA-p1 — transcript=ZTRI 2.441.mRNA — gene=ZTRI 2.441 — organism=Zymoseptoria tritici IPO323 — gene product=similar to fructose-bisphosphate aldolase — transcript product=similar to fructose-bisphosphate aldolase — location=Ztri chr 2:1394657-1395855(-) — protein length=359 — sequence SO=chromosome — SO=protein coding gene — is pseudo=false	355	0	355	508.834	243	295	1309	1	67.7	82.2
F.graminearum	XP_011318737.1 fructose-bisphosphate aldolase Fusarium graminearum PH-1	360	0	360	524.628	243	293	1350	1	67.7	81.6
C.truncatum	XP_036586175.1 fructose-bisphosphate aldolase Colletotrichum truncatum	362	8.2e-161	362	468.774	232	278	1205	3	64.6	77.4
B.cinerea	XP_001556818.1 Bcfba1 Botrytis cinerea B05.10	360	0	360	534.258	250	291	1375	1	69.6	81.1
B.graminis	VDB89263.1 — transcript=BGT962-24V316 LOCUS4968 t1 — gene=BGT-96224V316 LOCUS4968 — organism=Blumeria graminis f. sp. tritici 96224 — gene product=unspecified product — transcript product=unspecified product — location=LR026990:8163448-8164635(+) — protein length=344 — sequence SO=chromosome — SO=protein coding gene — is pseudo=false	333	1e-179	333	500.36	235	273	1287	1	65.5	76.0

Table S19: Pairwise alignment info from yeast Fba1 (DEG20010617), cf. Figure S73.

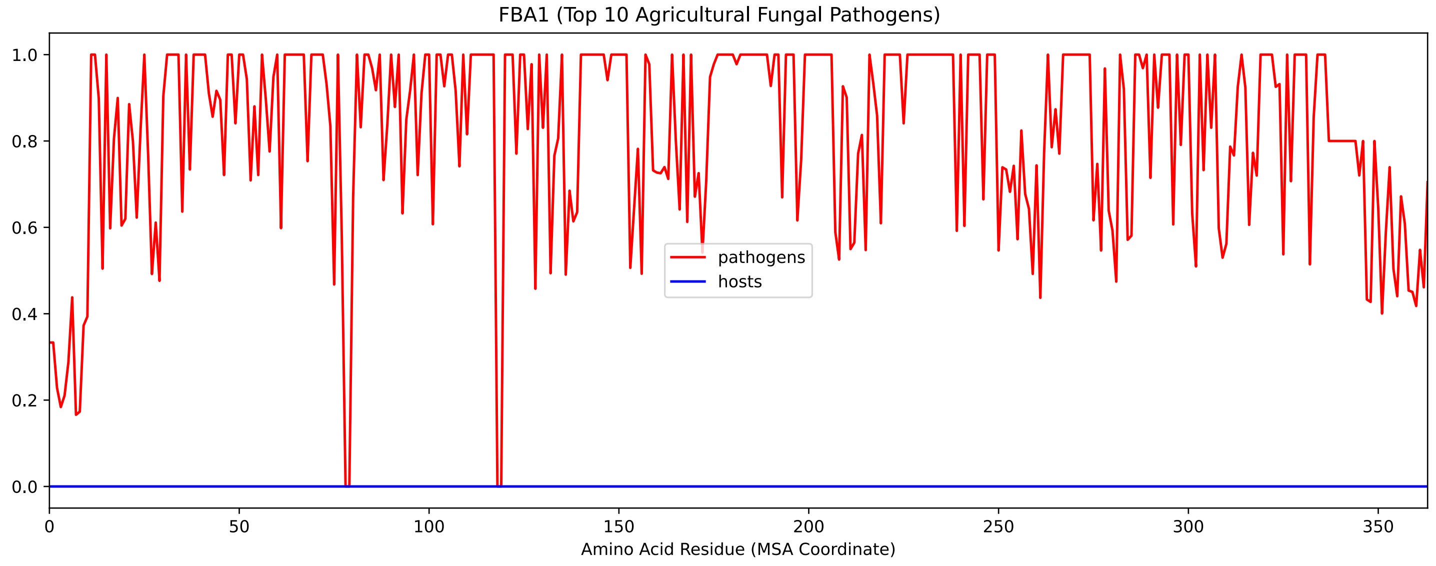

Figure S75: Sneath Similarity of Fba1 for Top 10 Agricultural Fungal Pathogens, cf. Figure S73

S2.7.3 NR

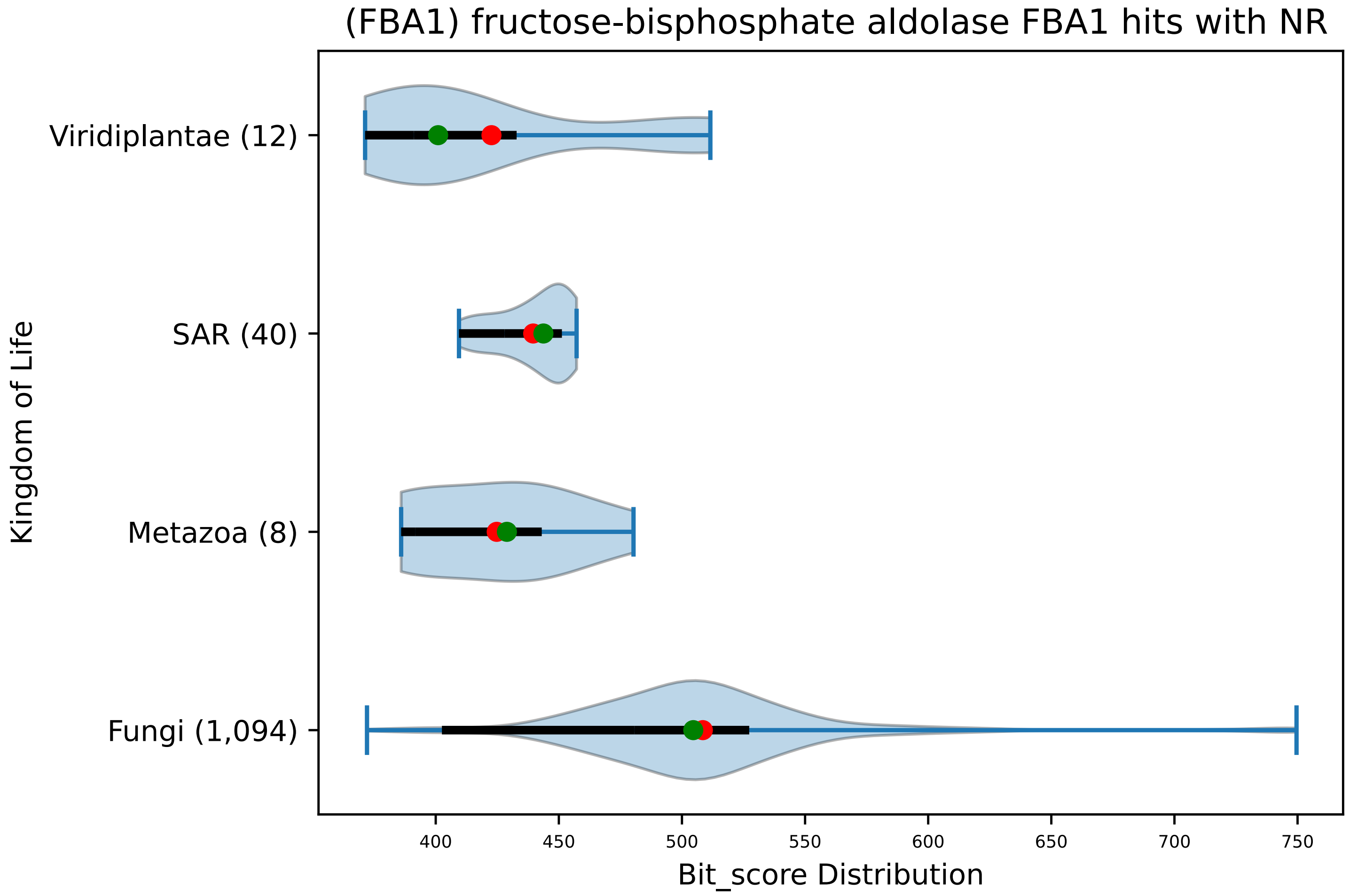

Figure S76: Non-redundant (NR) protein hits for DEG20010617/Fba1, with expectation value of no more than 0.1. Green points are medians, and red points are arithmetic means.

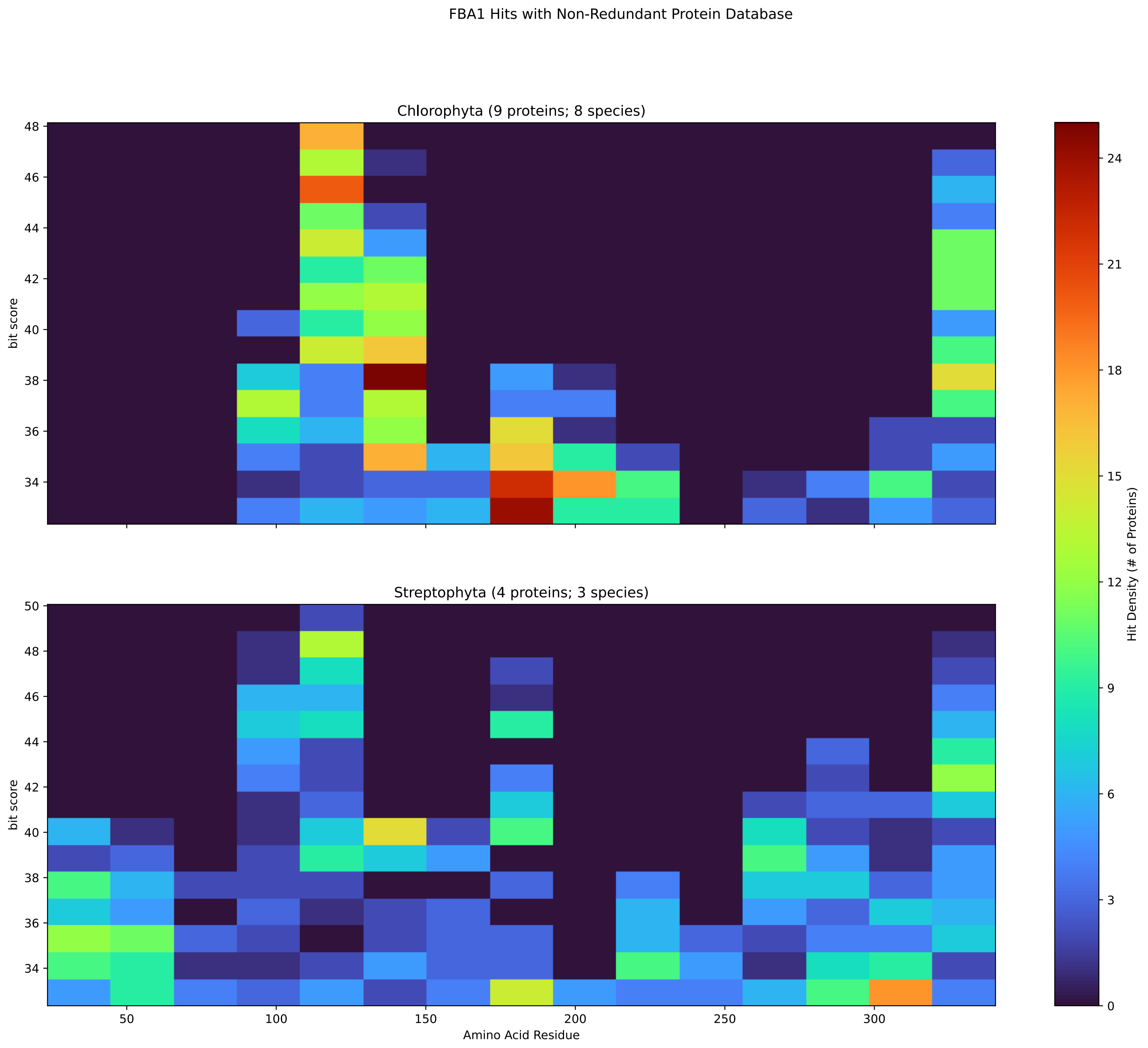

Figure S77: Non-redundant (NR) protein hits for Fba1 in the kingdom Viridiplantae.

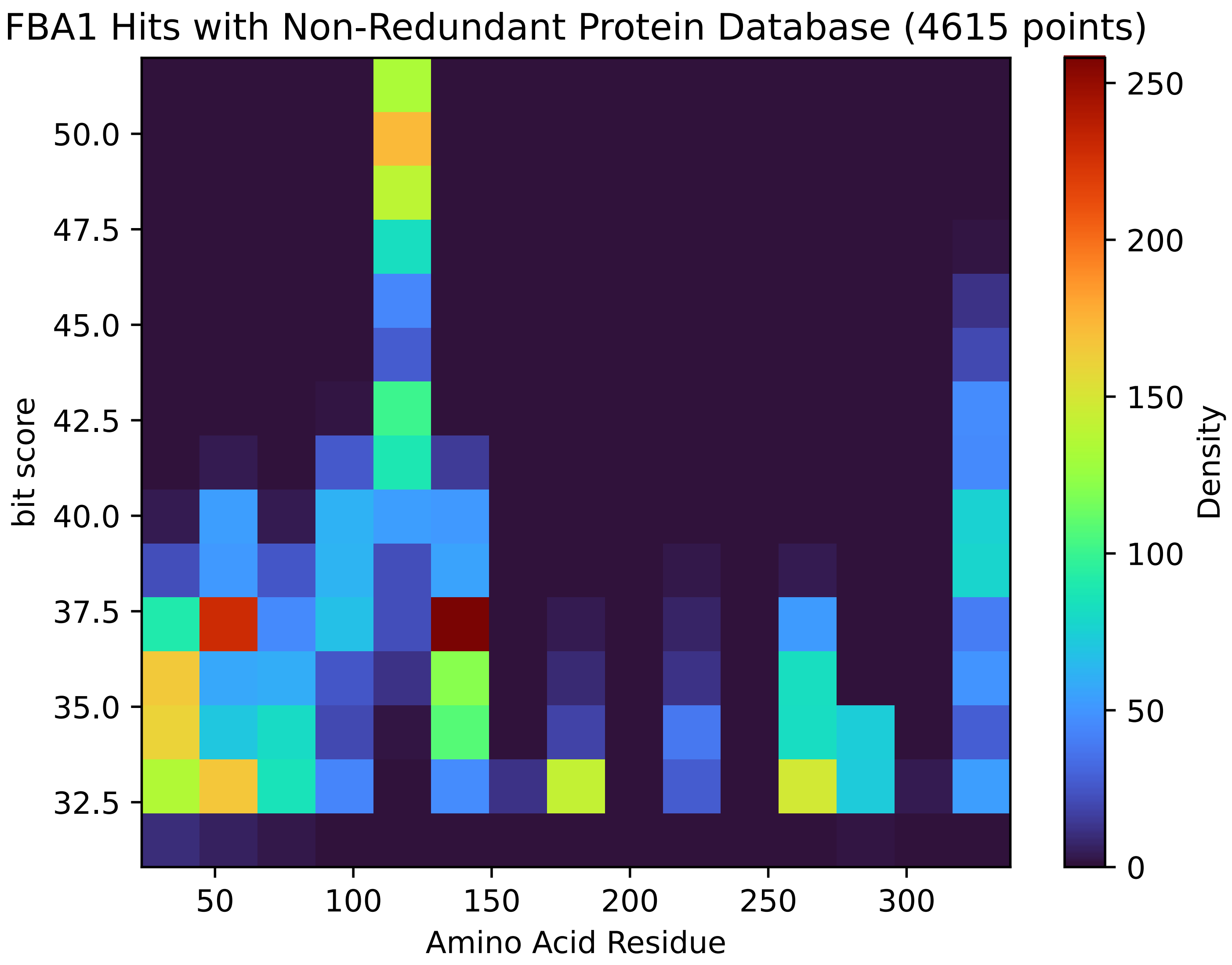

Figure S78: Non-redundant (NR) protein hits for Fba1 in the kingdom SAR.

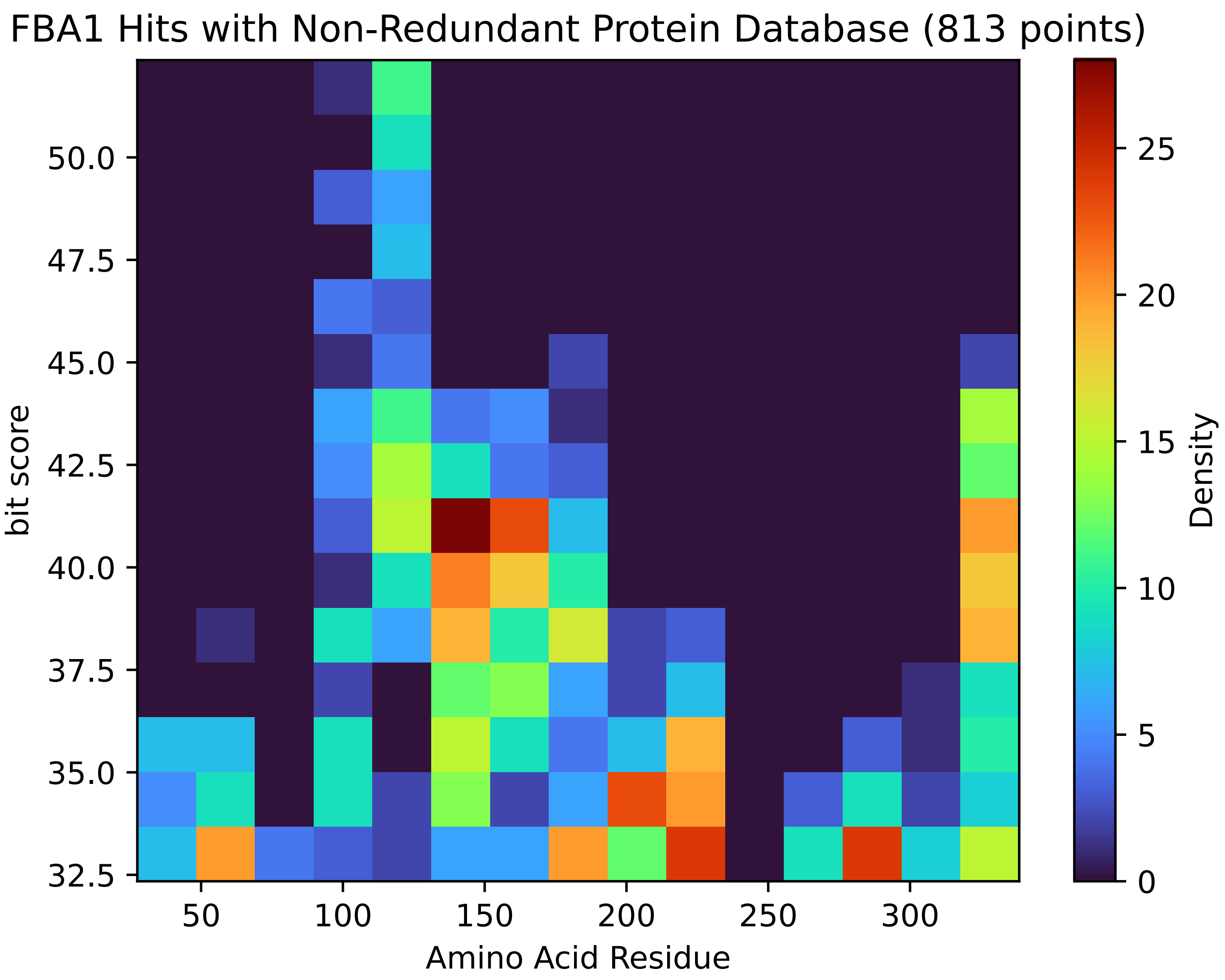

Figure S79: Non-redundant (NR) protein hits for Fba1 in the kingdom Metazoa.

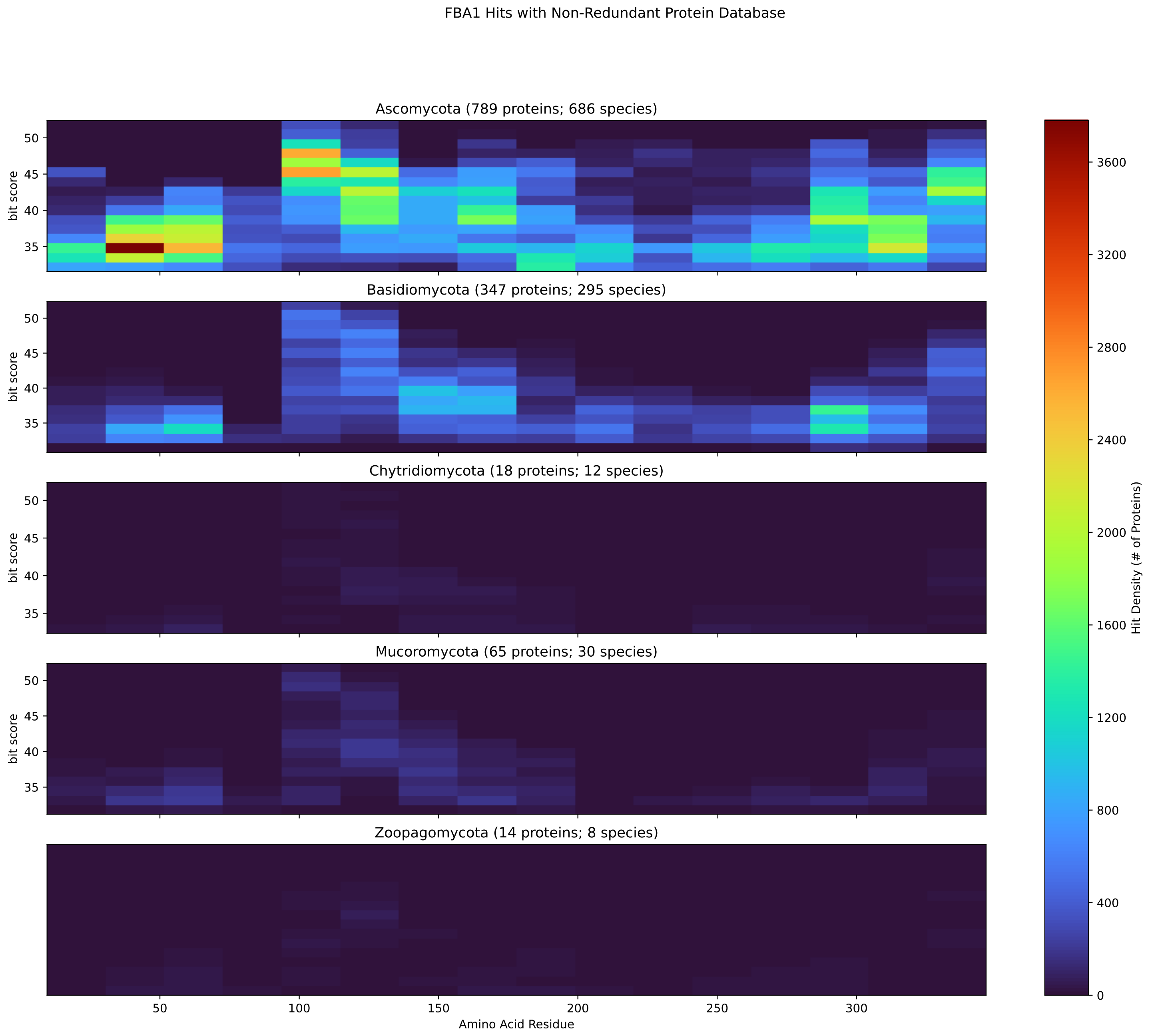

Figure S80: Non-redundant (NR) protein hits for Fba1 in the kingdom Fungi.

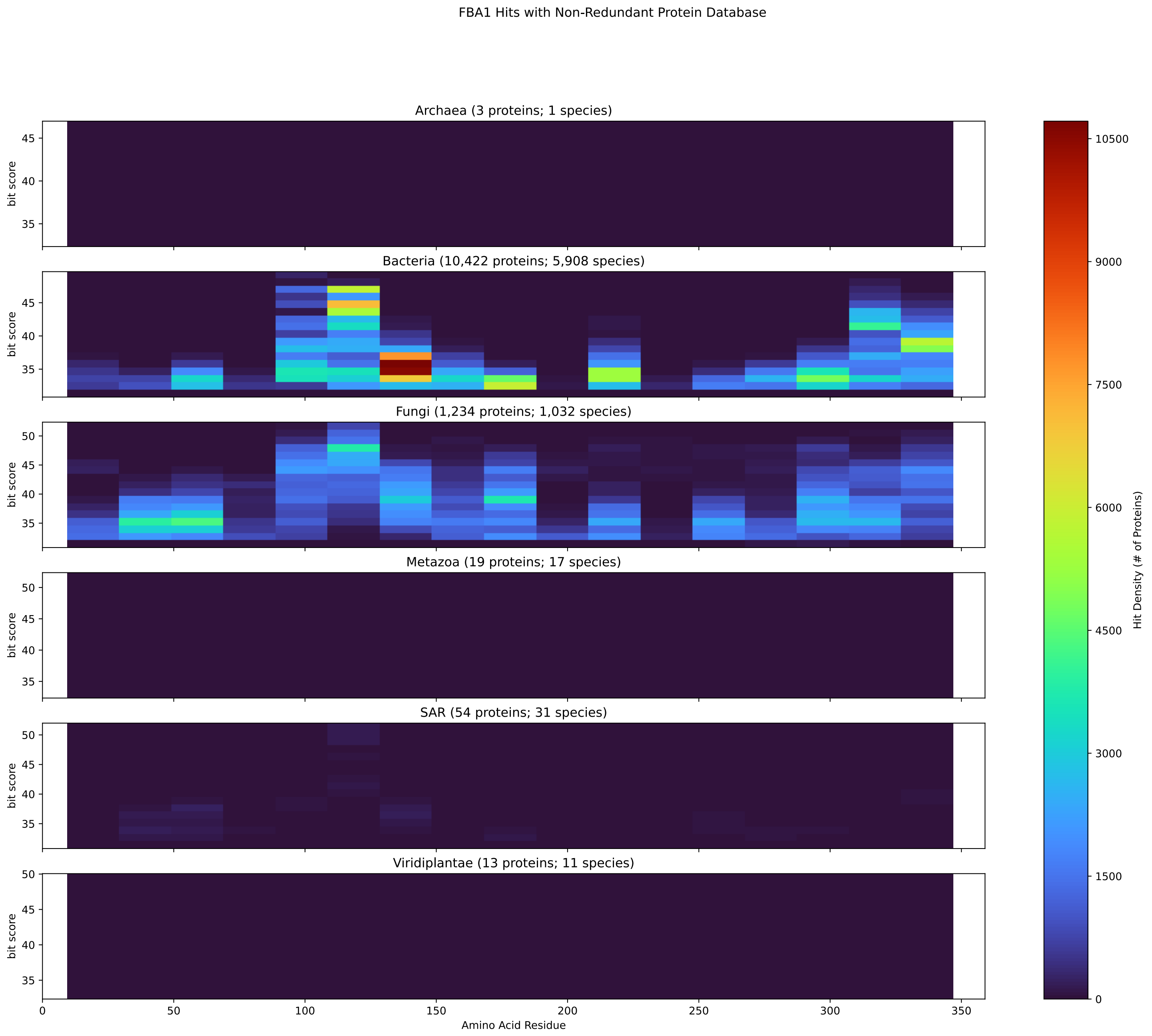

Figure S81: Non-redundant (NR) protein hits for DEG20010617/Fba1 at 20 amino acid length queries.

S2.8 Fcy21

S2.8.1 WHO Critical Pathogens

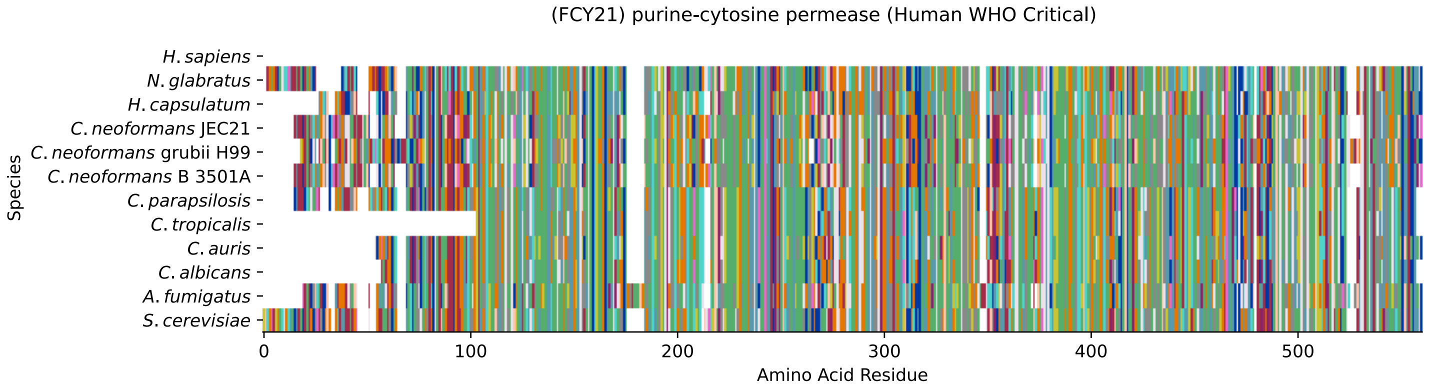

Figure S82: Multiple sequence alignment of yeast Fcy21 (WHO Critical Pathogens). Cf. Figure S83 for alignment quality, and Figure S84 for Sneath similarity. Cf. Table S20 for protein names, and pairwise alignment metrics with yeast Fcy21.

Fcy21 MSA Quality

	A. f.	*C. a.*	*C. a.*	*C. p.*	*C. t.*	*C. n.*	*C. n.*	*C. n.*	*H. c.*	*N. g.*	*S. c.*
A. fumigatus	1047.73	413.305	379.022	408.683	371.703	327.405	322.783	327.405	501.13	352.443	412.535
C. albicans	424.476	969.148	682.559	786.563	748.814	290.426	293.508	290.426	394.045	443.736	552.362
C. auris	379.022	672.929	990.334	658.677	608.986	266.929	301.982	266.929	382.489	443.351	533.872
C. parapsilosis	419.083	786.949	668.307	1042.72	723.391	298.13	285.419	298.13	388.267	454.907	544.273
C. tropicalis	382.489	748.814	618.231	723.005	878.241	261.922	261.922	261.922	374.015	421.009	509.99
C. neoformans B 3501A	338.961	295.049	278.485	301.212	265.774	1047.73	306.22	1047.73	319.316	293.508	305.064
C. neoformans grubii H99	348.206	318.546	333.569	310.457	286.189	323.168	1057.36	323.168	364.77	314.694	323.168
C. neoformans JEC21	338.961	295.049	278.485	301.212	265.774	1047.73	306.22	1047.73	319.316	293.508	305.064
H. capsulatum	501.13	382.104	382.489	376.711	363.229	305.834	330.102	305.834	1005.36	363.999	387.111
N. glabratus	358.607	445.277	454.136	456.447	422.165	285.419	296.204	285.419	369.777	1053.51	633.639
S. cerevisiae	412.535	543.117	533.872	534.643	500.745	295.049	291.197	295.049	387.111	620.542	1078.16

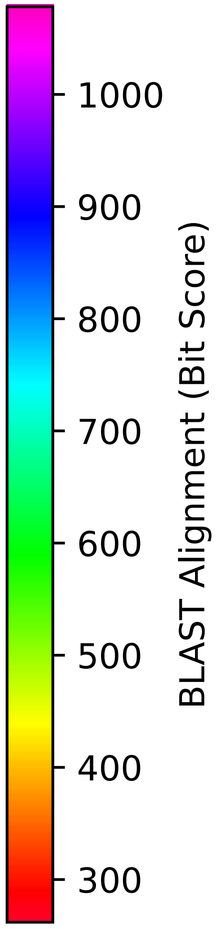

Figure S83: Multiple sequence alignment quality of Fcy21 (WHO Critical Pathogens). Cf. Figure S82

Species	Hit Protein	Hit Length (a.a.)	evalue	align_len	bit_score	identity	positive	score	gaps	% identity	% positive
H.sapiens	-	-	-	-	-	-	-	-	-	-	-
N.glabratus	XP_445191.1 uncharacterized p-rotein CAGL0C00231g Nakaseomyc-es glabratus	530	0	530	621.313	303	394	1601	14	57.4	74.6
H.capsulatum	XP_045291446.1 purine cytosin-e permease Fcy2 Histoplasma ca-psulatum G186AR	503	5.4e-130	503	387.111	198	293	993	6	37.5	55.5
C.neoformans.JEC21	XP_572600.1 cytosine-purine p-ermease, putative Cryptococcus neoformans var. neoformans JE-C21	525	4.4e-94	525	294.664	184	278	753	20	34.8	52.7
C.neoformans.grubii.H99	XP_012052683.1 cytosine perme-ase Cryptococcus neoformans va-r. grubii H99	528	1.1e-92	528	291.197	191	288	744	23	36.2	54.5
C.neoformans.B.3501A	XP_773950.1 hypothetical prot-ein CNBH4020 Cryptococcus neof-ormans var. neoformans B-3501A	525	4.3e-94	525	294.664	184	278	753	20	34.8	52.7
C.parapsilosis	XP_036665281.1 uncharacterize-d protein CPAR2 806580 Candida parapsilosis	511	0	511	535.413	264	358	1378	3	50.0	67.8
C.tropicalis	XP_002547752.1 purine-cytosin-e permease FCY2 Candida tropic-alis MYA-3404	437	9.4e-176	437	500.745	247	322	1288	2	46.8	61.0
C.auris	XP_028891542.1 hypothetical p-rotein Candida auris	485	0	485	535.028	258	349	1377	2	48.9	66.1
C.albicans	XP_714531.2 purine-cytosine p-ermease Candida albicans SC531-4	479	0	479	542.732	269	349	1397	2	50.9	66.1
A.fumigatus	XP_755319.1 purine-cytosine p-ermease Aspergillus fumigatus Af293	521	8.4e-140	521	412.92	222	319	1060	23	42.0	60.4

Table S20: Pairwise alignment info from yeast Fcy21 (DEG20010294), cf. Figure S82.

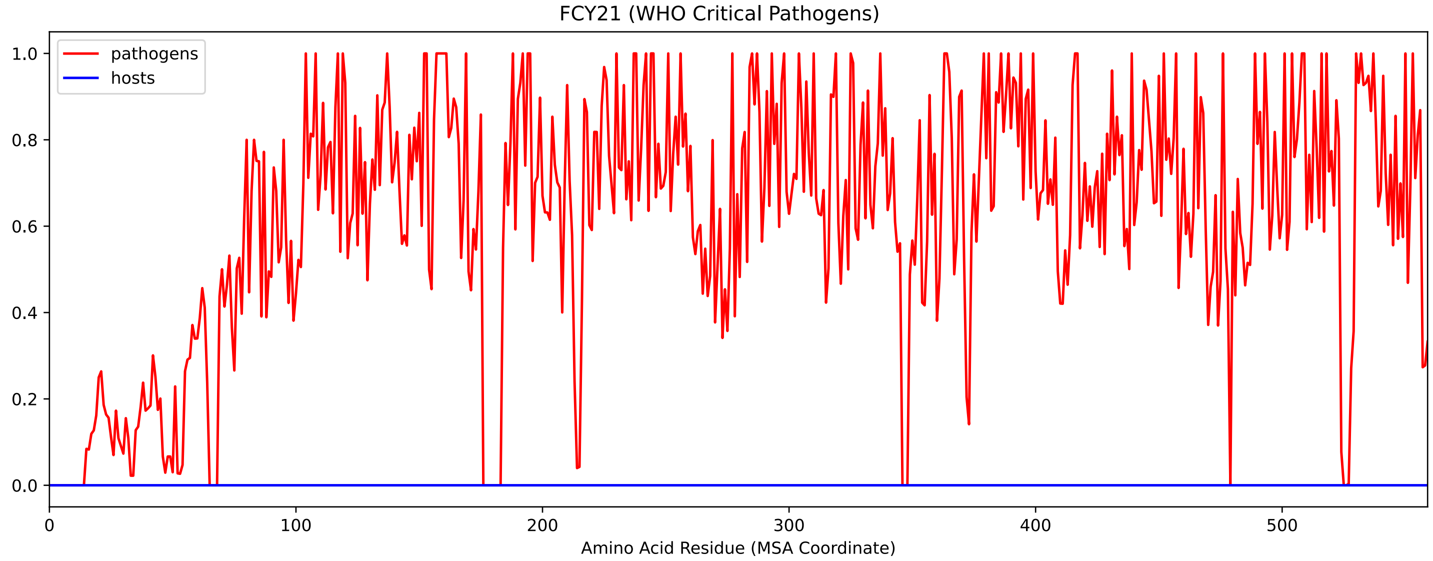

Figure S84: Sneath Similarity of Fcy21 for WHO Critical Pathogens, cf. Figure S82

S2.8.2 Top 10 Agricultural Fungal Pathogens

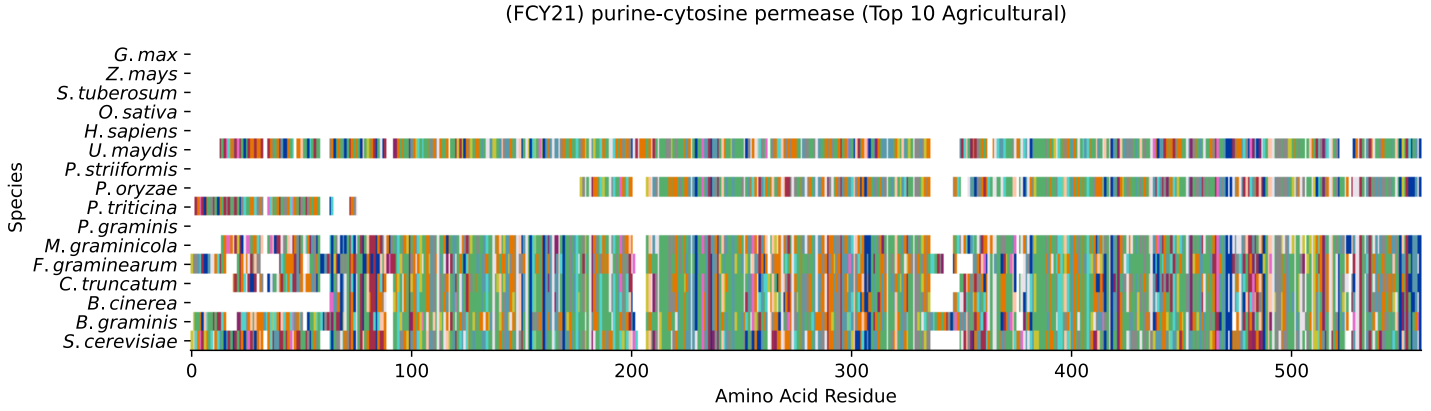

Figure S85: Multiple sequence alignment of yeast Fcy21 (Top 10 Agricultural Fungal Pathogens). Cf. Figure S86 for alignment quality, and Figure S87 for Sneath similarity. Cf. Table S21 for protein names, and pairwise alignment metrics with yeast Fcy21.

Fcy21 MSA Quality

	B.g.	*B.c.*	*C.t.*	*F.g.*	*M.g.*	*P.g.*	*P.t.*	*P.o.*	*P.s.*	*U.m.*	*S.c.*
B.graminis	1088.17	248.054	262.307	335.88	223.402	0	0	198.749	0	179.489	221.09
B.cinerea	237.654	946.421	634.41	252.677	570.852	0	0	451.825	0	232.646	387.882
C.truncatum	250.751	634.41	1028.47	250.366	601.668	0	0	474.167	0	241.506	416.001
F.graminearum	323.168	252.677	250.366	1056.59	226.483	0	0	210.305	0	201.06	265.003
M.graminicola	224.942	585.104	617.461	239.58	1045.03	0	0	422.935	0	236.113	390.963
P.graminis	0	0	0	0	0	0	0	0	0	0	0
P.triticina	0	0	0	0	0	0	111.309	0	0	0	34.2686
P.oryzae	186.037	451.825	474.167	210.305	411.379	0	0	720.309	0	182.57	280.796
P.striiformis	0	0	0	0	0	0	0	0	0	0	0
U.maydis	169.859	232.646	241.506	201.06	232.646	0	0	182.57	0	1034.25	208.764
S.cerevisiae	212.616	387.882	416.001	265.003	378.252	0	34.2686	280.796	0	208.764	1078.16

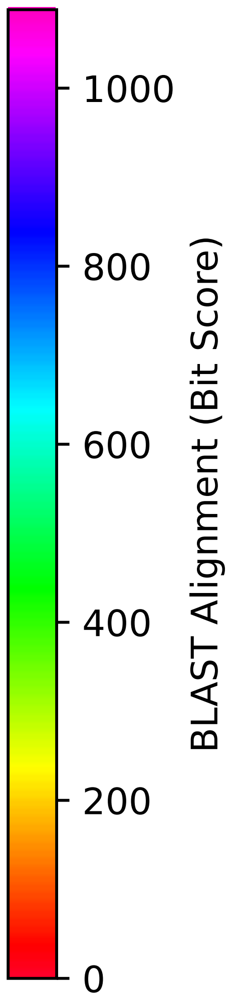

Figure S86: Multiple sequence alignment quality of Fcy21 (Top 10 Agricultural Fungal Pathogens). Cf. Figure S85

Species	Hit Protein	Hit Length (a.a.)	evalue	align_len	bit_score	identity	positive	score	gaps	% identity	% positive
G.max	-	-	-	-	-	-	-	-	-	-	-
Z.mays	-	-	-	-	-	-	-	-	-	-	-
S.tuberosum	-	-	-	-	-	-	-	-	-	-	-
O.sativa	-	-	-	-	-	-	-	-	-	-	-
H.sapiens	-	-	-	-	-	-	-	-	-	-	-
U.maydis	XP_011390683.1 uncharacterize-d protein UMAG 04197 Ustilago maydis 521	520	2.8e-61	520	208.764	144	260	530	20	27.3	49.2
P.striiformis	-	-	-	-	-	-	-	-	-	-	-
P.oryzae	mRNA M BR32 EuGene 00017951-p1 — transcript=mRNA M BR32 EuGe-ne 00017951 — gene=M BR32 EuGe-ne 00017951 — organism=Pyricul-aria oryzae BR32 — gene produc-t=unspecified product — transc-ript product=unspecified produ-ct — location=BR32 scaffold000-02:511879-513124(-) — protein length=359 — sequence SO=super-contig — SO=protein coding gen-e — is pseudo=false	362	1.2e-90	362	280.796	152	222	717	5	28.8	42.0
P.triticina	XP_053023736.1 uncharacterize-d protein PtA15 9A306 Puccinia triticina	61	0.085	61	34.2686	20	33	77	0	3.8	6.2
P.graminis	-	-	-	-	-	-	-	-	-	-	-
M.graminicola	ZTRI 1.37.mRNA-p1 — transcript-=ZTRI 1.37.mRNA — gene=ZTRI 1.-37 — organism=Zymoseptoria tri-tici IPO323 — gene product=hyp-othetical protein — transcript product=hypothetical protein — location=Ztri chr 1:223670-2-26762(-) — protein length=626 — sequence SO=chromosome — SO=-protein coding gene — is pseud-o=false	522	9.4e-125	522	377.867	209	309	969	11	39.6	58.5
F.graminearum	XP_011320615.1 hypothetical p-rotein FGSG 13426 Fusarium gra-minearum PH-1	535	2e-82	535	265.003	183	272	676	22	34.7	51.5
C.truncatum	XP_036587609.1 purine-cytosin-e permease (NCS1 nucleoside tr-ansporter) Colletotrichum trun-catum	513	6.7e-141	513	415.616	215	315	1067	10	40.7	59.7
B.cinerea	XP_001559974.1 hypothetical p-rotein BCIN 05g03050 Botrytis cinerea B05.10	475	3.8e-130	475	388.267	205	289	996	7	38.8	54.7
B.graminis	VCU39960.1 — transcript=BGT962-24V316 LOCUS1207 t1 — gene=BGT-96224V316 LOCUS1207 — organism-=Blumeria graminis f. sp. trit-ici 96224 — gene product=unspe-cified product — transcript pr-oduct=unspecified product — lo-cation=LR026985:8766531-876835-2(+) — protein length=534 — se-quence SO=chromosome — SO=prot-ein coding gene — is pseudo=fa-lse	543	1.1e-62	543	212.616	165	263	540	30	31.2	49.8

Table S21: Pairwise alignment info from yeast Fcy21 (DEG20010294), cf. Figure S85.

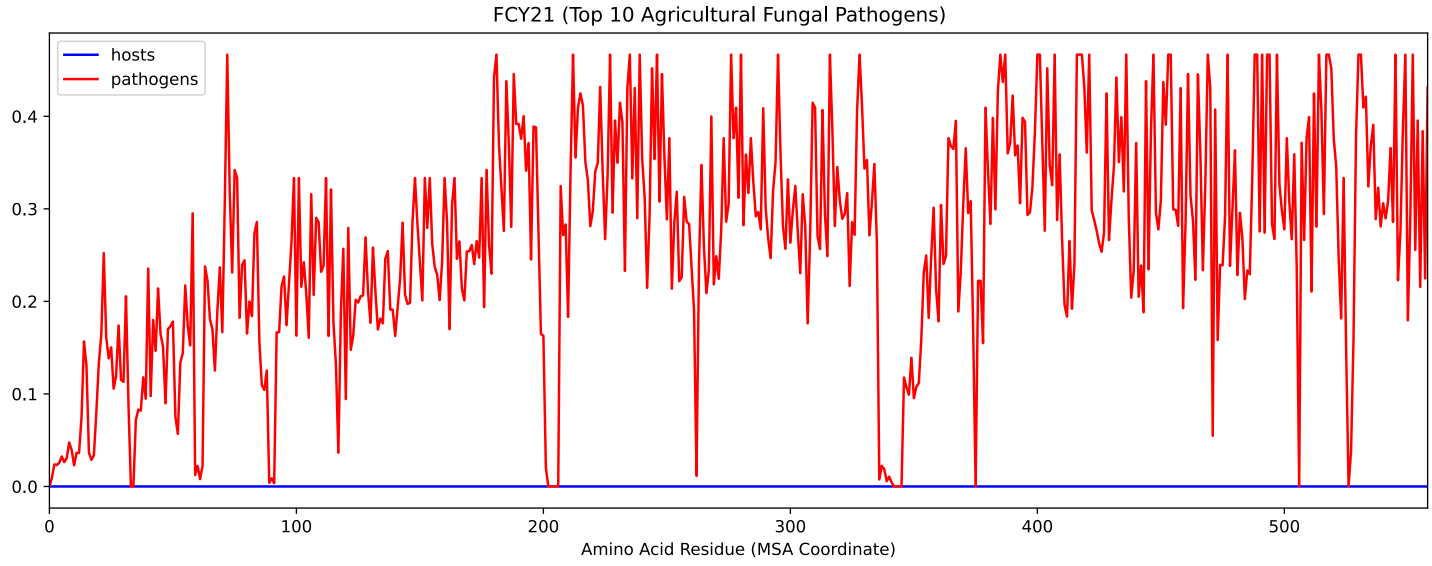

Figure S87: Sneath Similarity of Fcy21 for Top 10 Agricultural Fungal Pathogens, cf. Figure S85

S2.8.3 NR

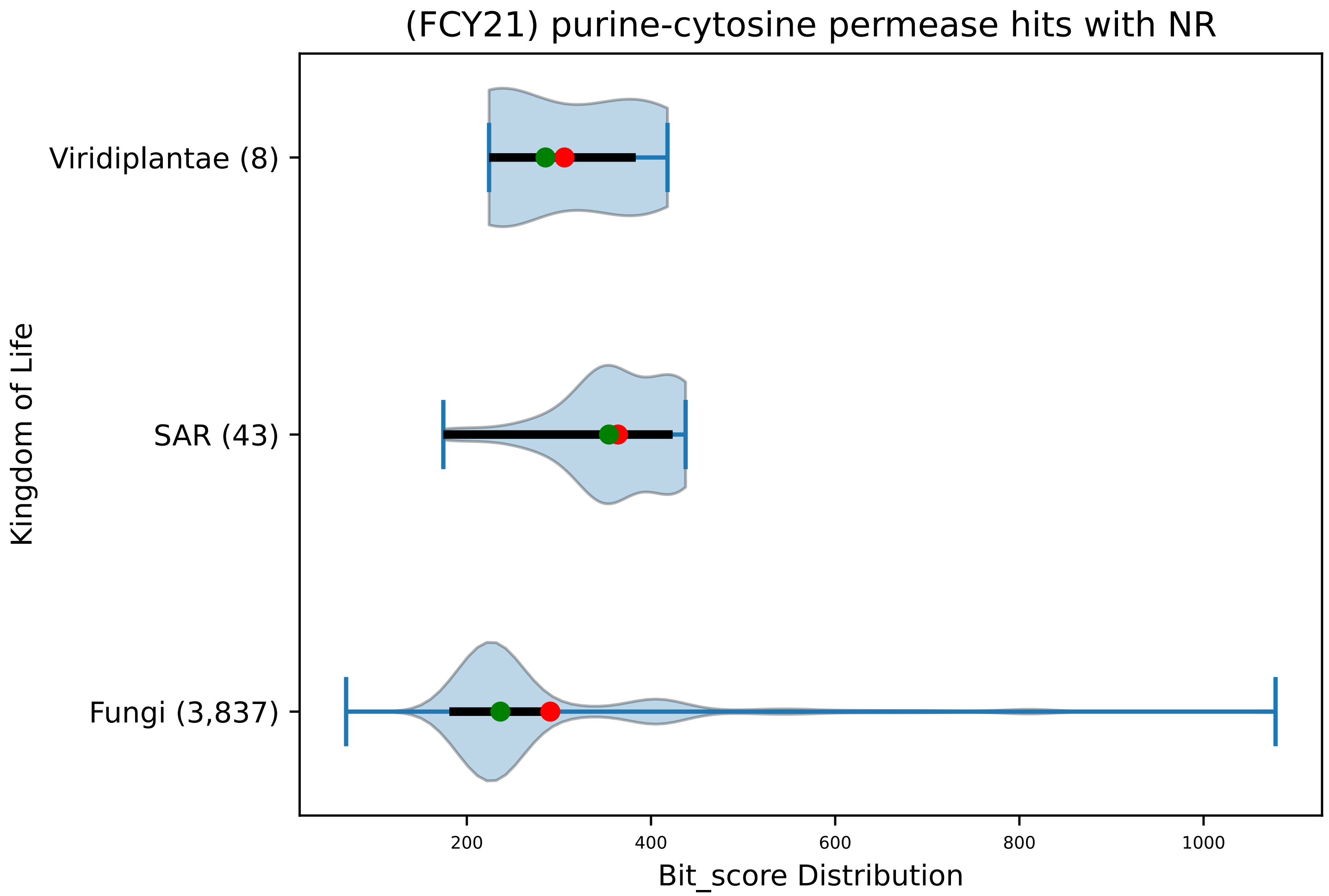

Figure S88: Non-redundant (NR) protein hits for DEG20010294/Fcy21, with expectation value of no more than 0.1. Green points are medians, and red points are arithmetic means.

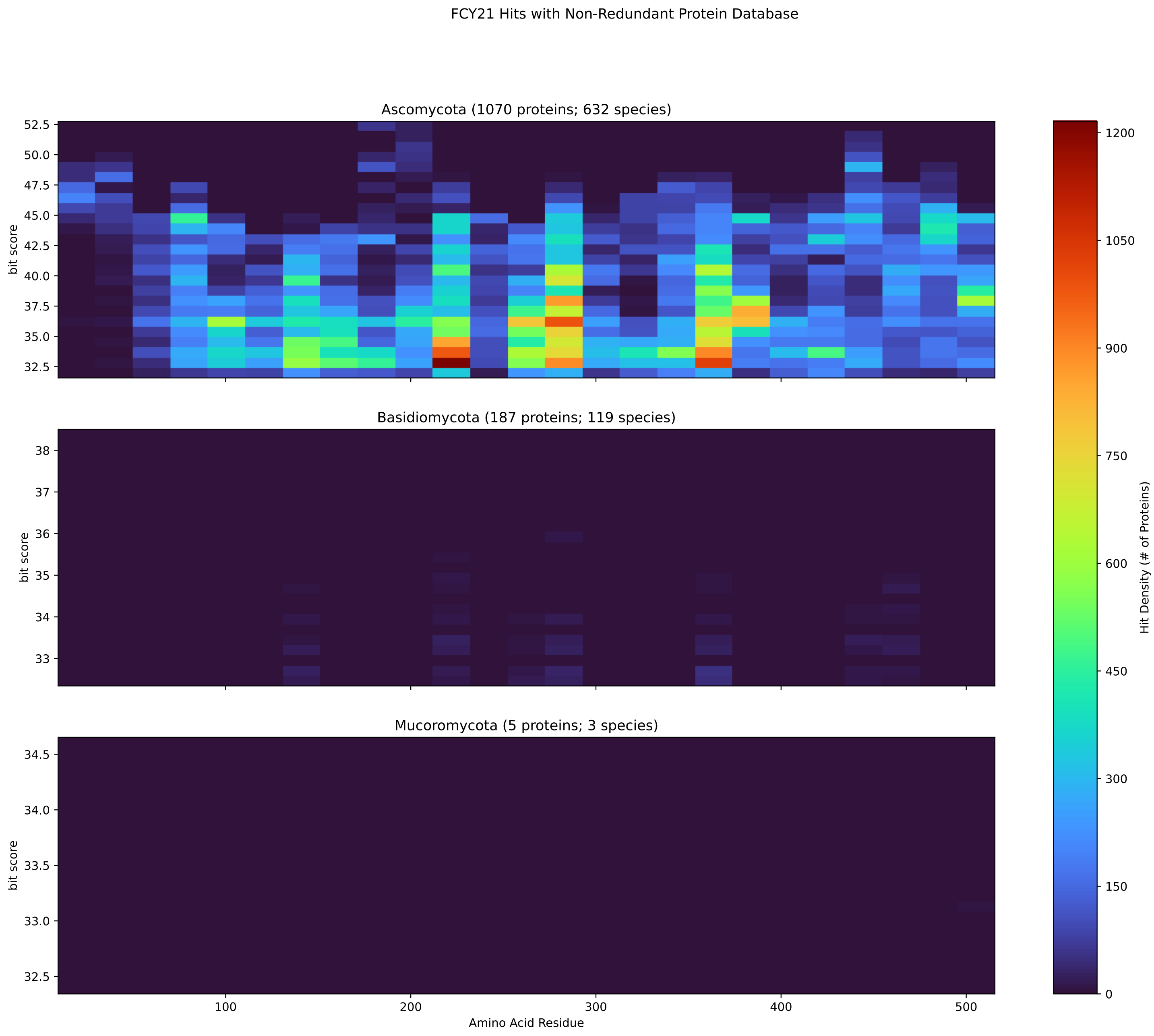

Figure S89: Non-redundant (NR) protein hits for Fcy21 in the kingdom Fungi.

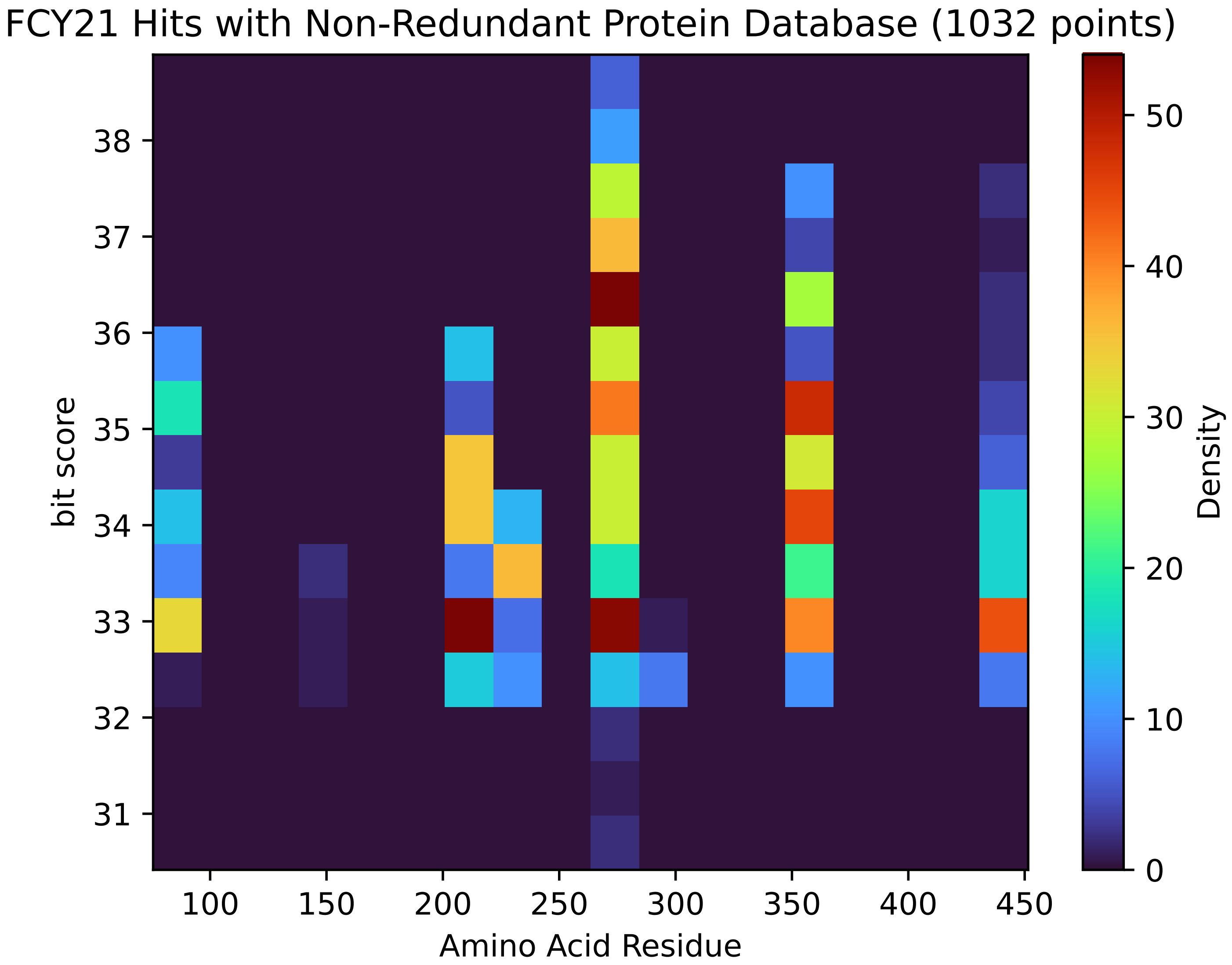

Figure S90: Non-redundant (NR) protein hits for Fcy21 in the kingdom SAR.

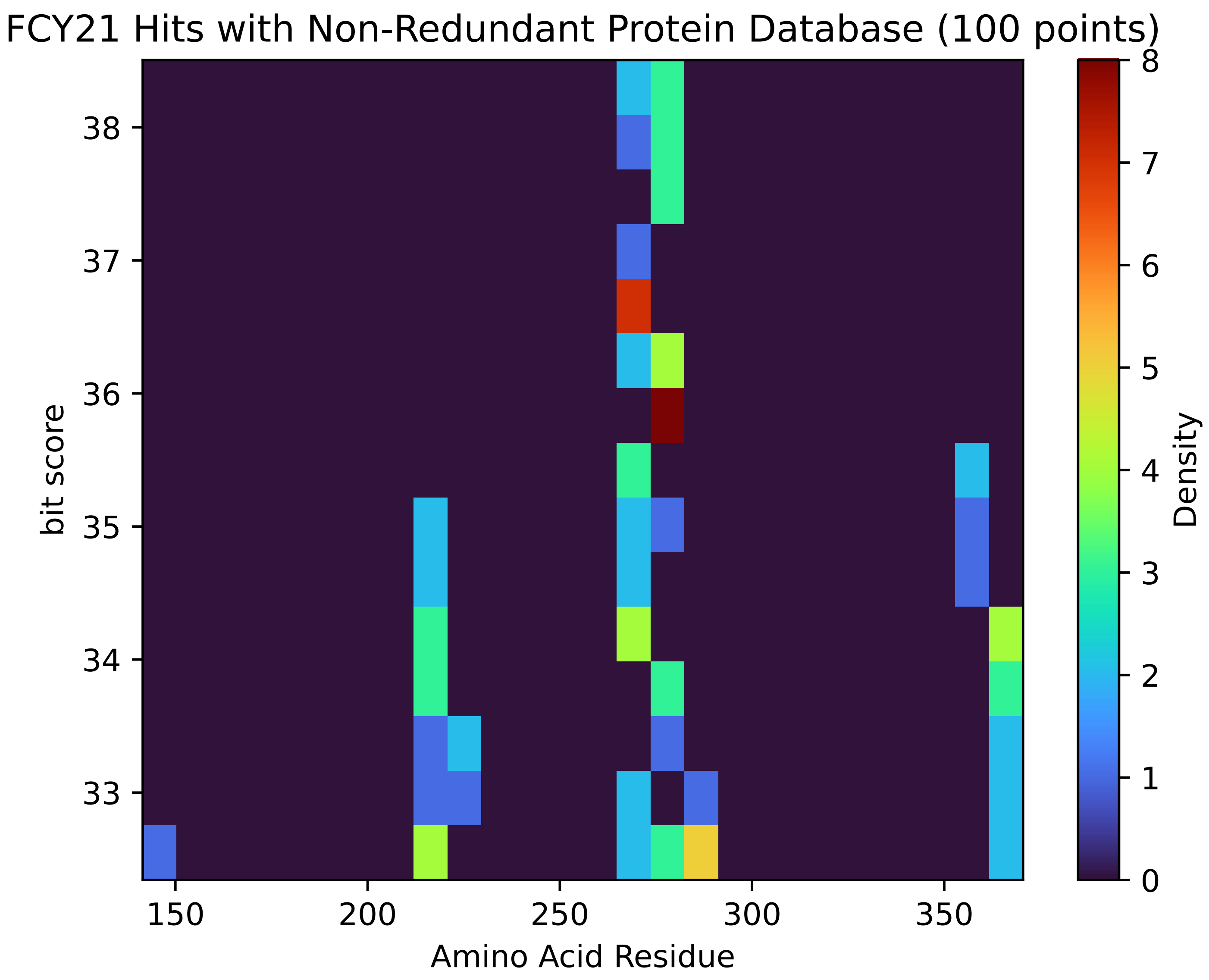

Figure S91: Non-redundant (NR) protein hits for Fcy21 in the kingdom Viridiplantae.

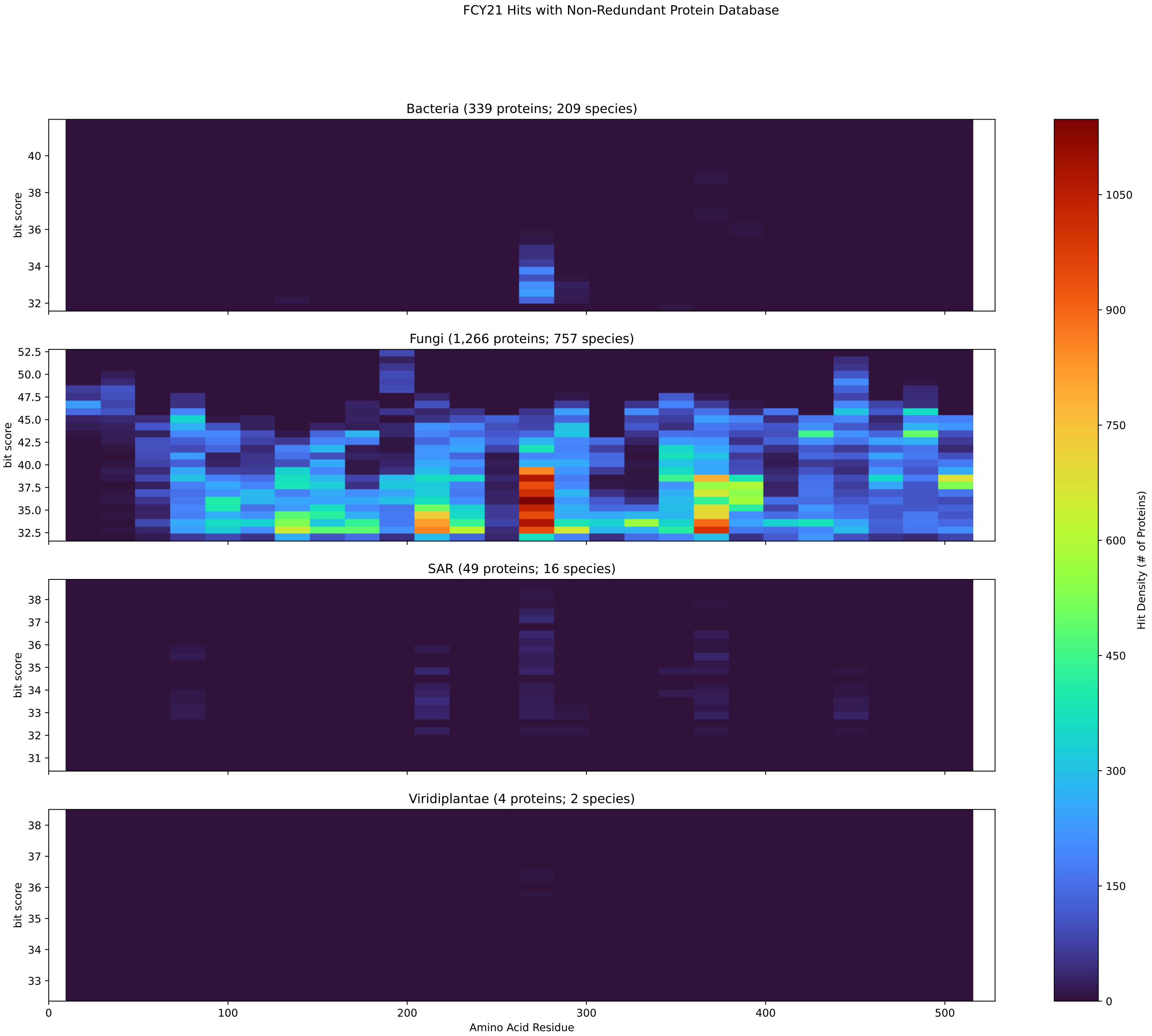

Figure S92: Non-redundant (NR) protein hits for DEG20010294/Fcy21 at 20 amino acid length queries.

S2.9 Fol1

S2.9.1 WHO Critical Pathogens

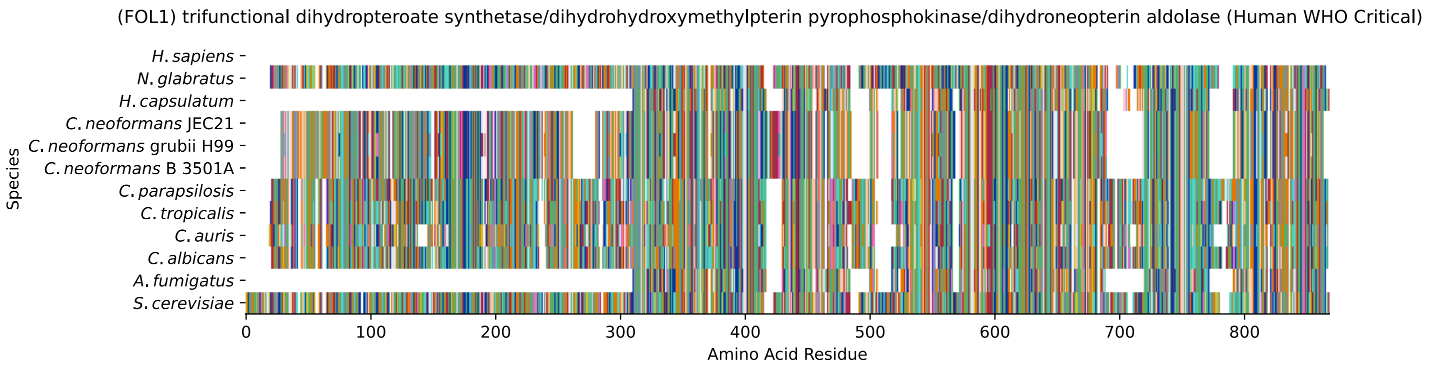

Figure S93: Multiple sequence alignment of yeast Fol1 (WHO Critical Pathogens). Cf. Figure S94 for alignment quality, and Figure S95 for Sneath similarity. Cf. Table S22 for protein names, and pairwise alignment metrics with yeast Fol1.

Fol1 MSA Quality

	A. f.	*C. a.*	*C. a.*	*C. p.*	*C. t.*	*C. n.*	*C. n.*	*C. n.*	*H. c.*	*N. g.*	*S. c.*
A. fumigatus	920.228	378.252	360.533	331.257	374.785	315.464	326.25	313.153	587.03	303.523	331.643
C. albicans	380.563	1606.65	754.592	1057.74	1261.9	310.071	318.546	313.538	342.428	497.278	534.258
C. auris	355.14	742.265	1570.44	723.391	761.911	288.5	279.641	282.722	324.324	496.508	506.138
C. parapsilosis	326.635	1041.95	723.391	1624.37	1060.06	268.085	265.774	266.544	305.449	501.13	522.702
C. tropicalis	369.392	1243.41	761.911	1060.06	1615.9	291.967	296.59	296.975	313.923	509.99	547.74
C. neoformans B 3501A	308.145	298.901	288.5	268.085	291.967	1463.74	1382.08	1454.5	299.671	315.849	320.857
C. neoformans grubii H99	320.472	308.531	279.641	265.774	296.59	1382.08	1469.52	1382.85	301.982	311.227	313.923
C. neoformans JEC21	307.375	302.753	282.722	266.544	296.975	1454.5	1382.85	1464.51	297.36	313.538	317.005
H. capsulatum	598.201	350.517	343.584	323.553	332.798	320.472	324.709	318.931	955.281	273.863	310.457
N. glabratus	298.516	483.411	496.508	501.13	509.99	315.849	311.227	313.538	256.144	1634.77	854.744
S. cerevisiae	327.02	521.931	506.138	522.702	547.74	320.857	313.923	317.005	292.352	854.744	1704.88

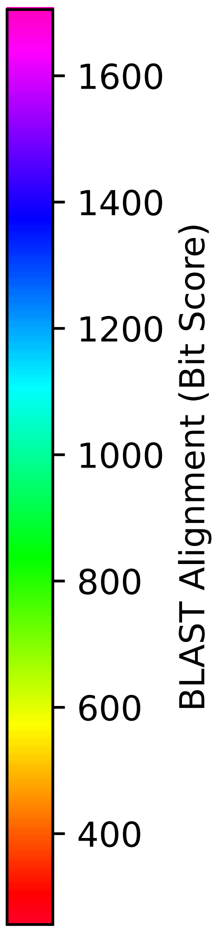

Figure S94: Multiple sequence alignment quality of Fol1 (WHO Critical Pathogens). Cf. Figure S93

Species	Hit Protein	Hit Length (a.a.)	evalue	align_len	bit_score	identity	positive	score	gaps	% identity	% positive
H.sapiens	-	-	-	-	-	-	-	-	-	-	-
N.glabratus	XP_448052.1 uncharacterized p-rotein CAGL0J07920g Nakaseomyc-es glabratus	806	0	806	855.129	417	561	2208	21	50.6	68.1
H.capsulatum	XP_045283569.1 folic acid syn-thesis protein Histoplasma cap-sulatum G186AR	528	2e-88	528	292.738	198	280	748	63	24.0	34.0
C.neoformans.JEC21	XP_570005.1 folic acid and de-rivative biosynthesis-related protein, putative Cryptococcus neoformans var. neoformans JE-C21	818	6e-97	818	317.005	260	386	811	126	31.6	46.8
C.neoformans.grubii.H99	XP_012048132.1 dihydropteroat-e synthase Cryptococcus neofor-mans var. grubii H99	819	9.2e-96	819	313.923	263	391	803	128	31.9	47.5
C.neoformans.B.3501A	XP_776823.1 hypothetical prot-ein CNBC3140 Cryptococcus neof-ormans var. neoformans B-3501A	818	1.5e-98	818	321.242	261	387	822	126	31.7	47.0
C.parapsilosis	XP_036664526.1 uncharacterize-d protein CPAR2 303390 Candida parapsilosis	828	4.7e-175	828	522.702	317	488	1345	65	38.5	59.2
C.tropicalis	XP_002551057.1 conserved hypo-thetical protein Candida tropi-calis MYA-3404	833	0	833	548.51	334	495	1412	73	40.5	60.1
C.auris	XP_028891915.2 dihydropteroat-e synthase Candida auris	823	4.9e-169	823	505.753	324	452	1301	83	39.3	54.9
C.albicans	XP_714207.2 trifunctional dih-ydropteroate synthetase/dihydr-ohydroxymethylpterin pyrophosp-hokinase/dihydroneopterin aldo-lase Candida albicans SC5314	829	4.5e-175	829	522.702	322	494	1345	72	39.1	60.0
A.fumigatus	XP_755317.1 folic acid synthe-sis protein Aspergillus fumiga-tus Af293	527	6.1e-104	527	327.405	206	290	838	79	25.0	35.2

Table S22: Pairwise alignment info from yeast Fol1 (DEG20010889), cf. Figure S93.

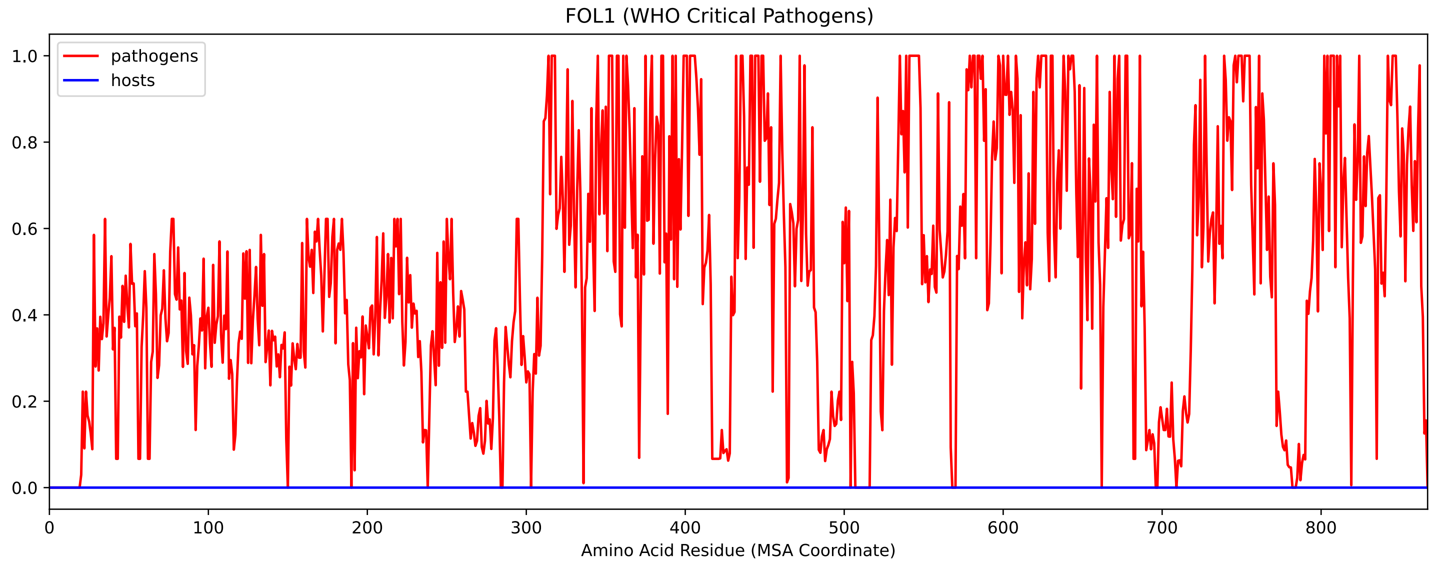

Figure S95: Sneath Similarity of Fol1 for WHO Critical Pathogens, cf. Figure S93

S2.9.2 Top 10 Agricultural Fungal Pathogens

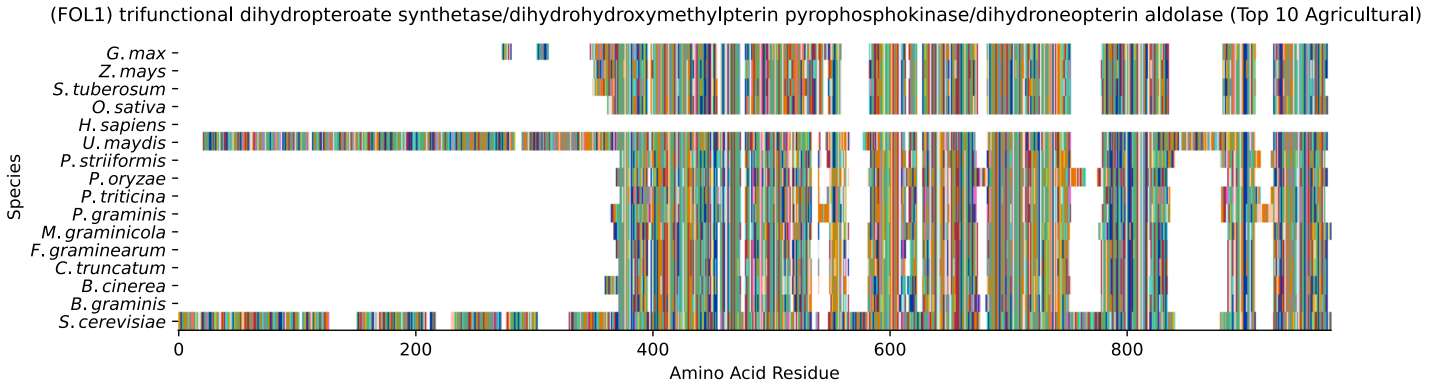

Figure S96: Multiple sequence alignment of yeast Fol1 (Top 10 Agricultural Fungal Pathogens). Cf. Figure S97 for alignment quality, and Figure S98 for Sneath similarity. Cf. Table S23 for protein names, and pairwise alignment metrics with yeast Fol1.

Fol1 MSA Quality

	B. g.	B. c.	C. t.	F. g.	M. g.	P. g.	P. t.	P. o.	P. s.	U. m.	S. c.
B. graminis	919.842	575.859	521.161	545.814	441.039	261.922	271.552	491.5	277.715	278.1	283.493
B. cinerea	544.273	942.184	619.772	622.083	488.034	285.419	300.442	581.252	295.049	330.102	323.553
C. truncatum	512.301	640.188	910.983	653.284	485.337	271.166	289.656	580.096	285.419	313.153	301.212
F. graminearum	528.865	637.876	648.662	905.975	498.819	284.648	291.967	586.645	291.967	300.442	299.286
M. graminicola	414.461	488.034	463.766	484.567	946.421	293.508	286.189	438.728	316.235	320.857	311.612
P. graminis	279.256	310.071	279.256	300.442	318.931	977.622	657.907	265.388	677.552	362.073	256.529
P. triticina	280.796	317.005	289.656	303.138	304.294	637.106	923.309	269.24	620.542	334.724	248.44
P. oryzae	502.671	624.78	603.594	614.379	479.559	269.626	279.641	962.214	289.271	295.819	302.368
P. striiformis	278.1	308.916	280.411	295.819	328.561	654.44	613.609	272.707	948.347	367.466	263.462
U. maydis	277.33	350.517	310.071	306.22	338.191	357.066	332.028	280.026	374.4	1731.07	326.25
S. cerevisiae	263.077	323.553	286.96	286.96	311.612	241.506	236.498	273.863	252.677	303.138	1704.88

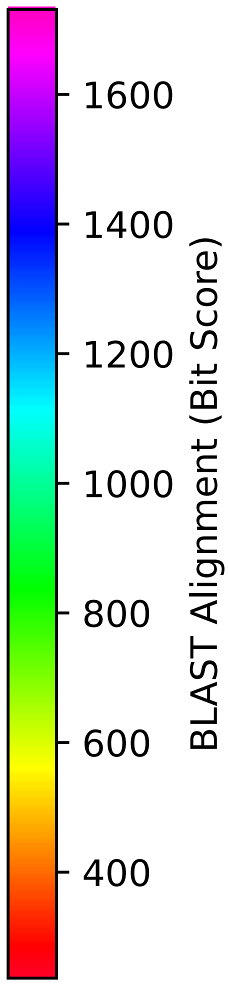

Figure S97: Multiple sequence alignment quality of Fol1 (Top 10 Agricultural Fungal Pathogens). Cf. Figure S96

Species	Hit Protein	Hit Length (a.a.)	evalue	align_len	bit_score	identity	positive	score	gaps	% identity	% positive
G.max	XP_003518782.1 folate synthesis bifunctional protein, mitochondrial Glycine max	574	8.8e-75	574	254.218	180	290	648	74	21.8	35.2
Z.mays	NP_001146490.1 Folate synthesis bifunctional protein, mitochondrial-like Zea mays	546	2.9e-66	546	230.72	166	267	587	68	20.1	32.4
S.tuberosum	XP_006348669.1 PREDICTED: folate synthesis bifunctional protein, mitochondrial-like Solanum tuberosum	559	1.1e-72	559	247.284	181	301	630	86	22.0	36.5
O.sativa	XP_015646629.1 folate synthesis bifunctional protein, mitochondrial isoform X6 Oryza sativa Japonica Group	539	1.8e-72	539	247.669	173	274	631	76	21.0	33.3
H.sapiens	-	-	-	-	-	-	-	-	-	-	-
U.maydis	XP_011388224.1 trifunctional dihydropteroate synthetase/dihydrohydroxymethylpterin pyrophosphokinase/dihydroneopterin aldolase FOL1 Ustilago maydis 521	923	1.1e-90	923	303.523	272	430	776	196	33.0	52.2
P.striiformis	XP_047809565.1 hypothetical protein Pst134EA 007376 Puccinia striiformis f. sp. tritici	529	1.2e-74	529	252.292	174	280	643	72	21.1	34.0
P.oryzae	mRNA M BR32 EuGene 00010841-p1 — transcript=mRNA M BR32 EuGene 00010841 — gene=M BR32 EuGene 00010841 — organism=Pyricularia oryzae BR32 — gene product=unspecified product — transcript product=unspecified product — location=BR32 scaffold000-01:3219749-3221562(+) — protein length=577 — sequence SO=supercontig — SO=protein coding gene — is pseudo=false	539	2.2e-82	539	274.248	192	288	700	77	23.3	35.0
P.triticina	XP_053018629.1 uncharacterized protein PtA15 3A441 Puccinia triticina	530	2.3e-69	530	236.113	174	260	601	83	21.1	31.6
P.graminis	XP_003324553.1 hypothetical protein PGTG 05359 Puccinia graminis f. sp. tritici CRL 75-36-700-3	546	5.7e-71	546	241.891	177	278	616	83	21.5	33.7
M.graminicola	ZTRI 4.793.mRNA-p1 — transcript=ZTRI 4.793.mRNA — gene=ZTRI 4.793 — organism=Zymoseptoria tritici IPO323 — gene product=hypothetical protein — transcript product=hypothetical protein — location=Ztri chr 4:25441-78-2550684(-) — protein length=2100 — sequence SO=chromosome — SO=protein coding gene — is pseudo=false	533	2.5e-90	533	311.997	196	291	798	79	23.8	35.3
F.graminearum	XP_011327992.1 hypothetical protein FGSG 09710 Fusarium graminearum PH-1	527	2.8e-87	527	286.189	192	275	731	83	23.3	33.4
C.truncatum	XP_036583196.1 dihydropteroate synthase, partial Colletotrichum truncatum	527	9.6e-87	527	287.345	195	271	734	86	23.7	32.9
B.cinerea	XP_024553509.1 Bcfol1 Botrytis cinerea B05.10	542	2e-101	542	323.168	201	293	827	84	24.4	35.6
B.graminis	VDB89524.1 — transcript=BGT962-24V316 LOCUS5151 t1 — gene=BGT-96224V316 LOCUS5151 — organism=Blumeria graminis f. sp. tritici 96224 — gene product=unspecified product — transcript product=unspecified product — location=LR026990:10590963-10592-601(+) — protein length=513 — sequence SO=chromosome — SO=protein coding gene — is pseudo=false	527	2.7e-79	527	263.077	191	275	671	85	23.2	33.4

Table S23: Pairwise alignment info from yeast Fol1 (DEG20010889), cf. Figure S96.

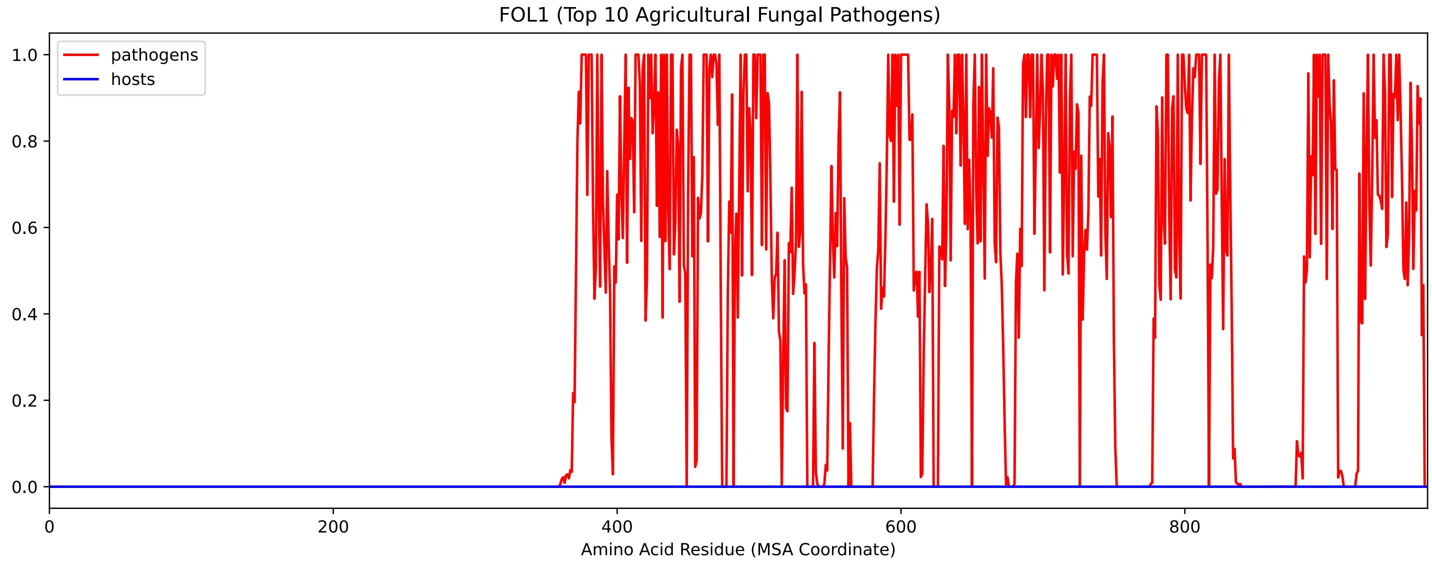

Figure S98: Sneath Similarity of Fol1 for Top 10 Agricultural Fungal Pathogens, cf. Figure S96

S2.9.3 NR

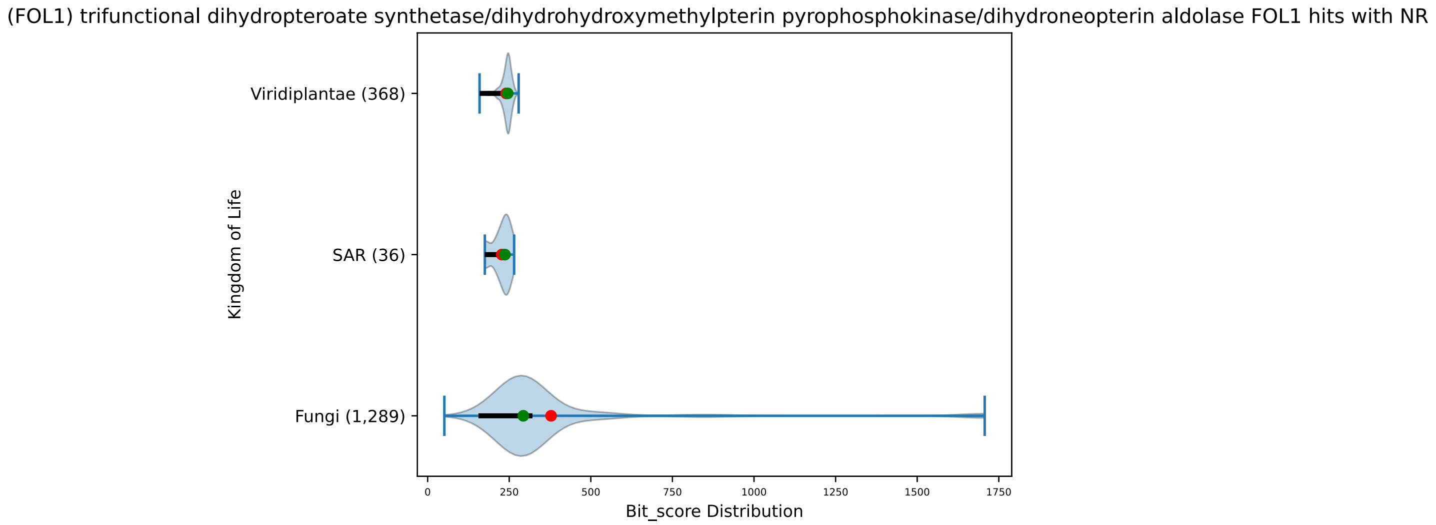

Figure S99: Non-redundant (NR) protein hits for DEG20010889/Fol1, with expectation value of no more than 0.1. Green points are medians, and red points are arithmetic means.

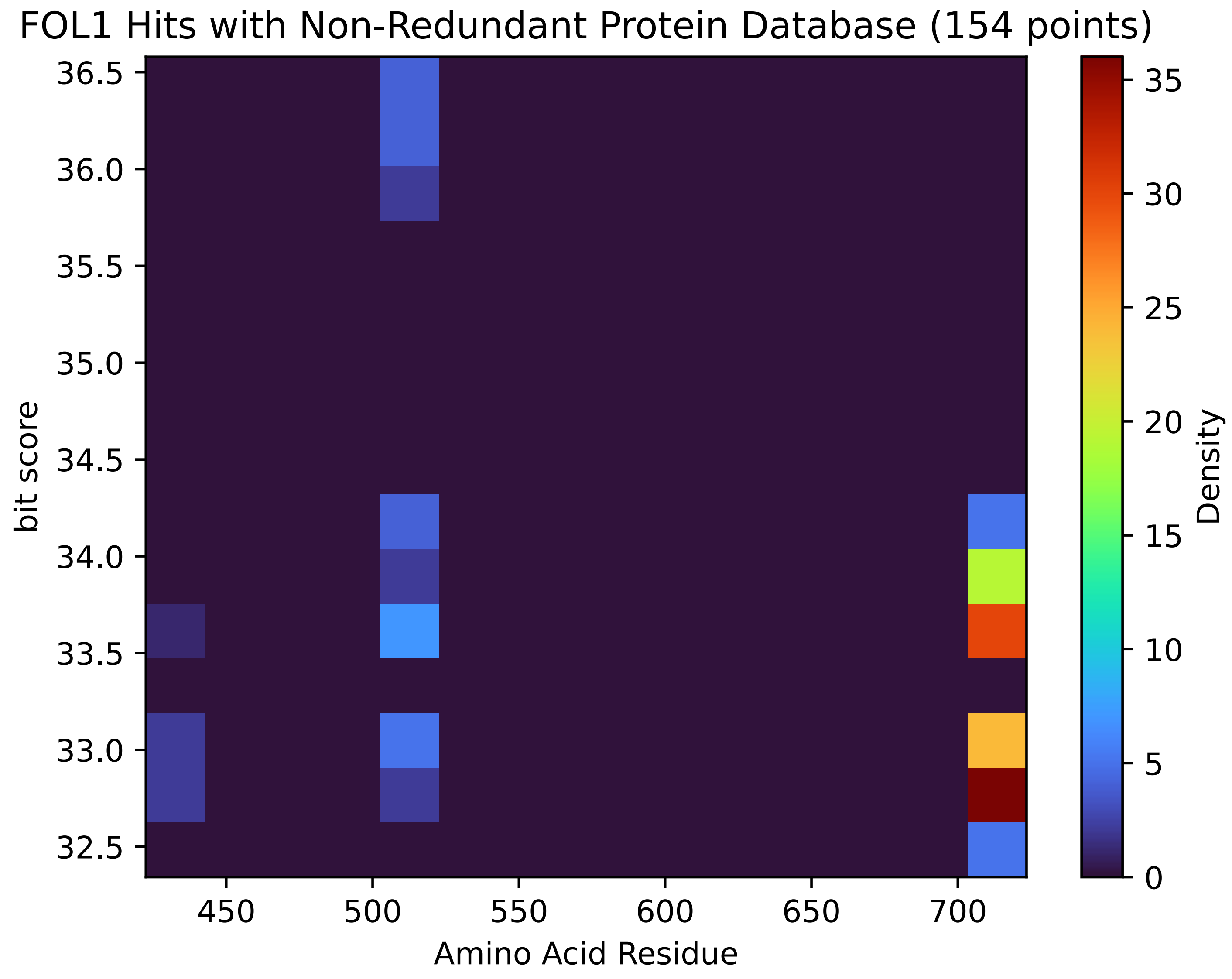

Figure S100: Non-redundant (NR) protein hits for Fol1 in the kingdom SAR.

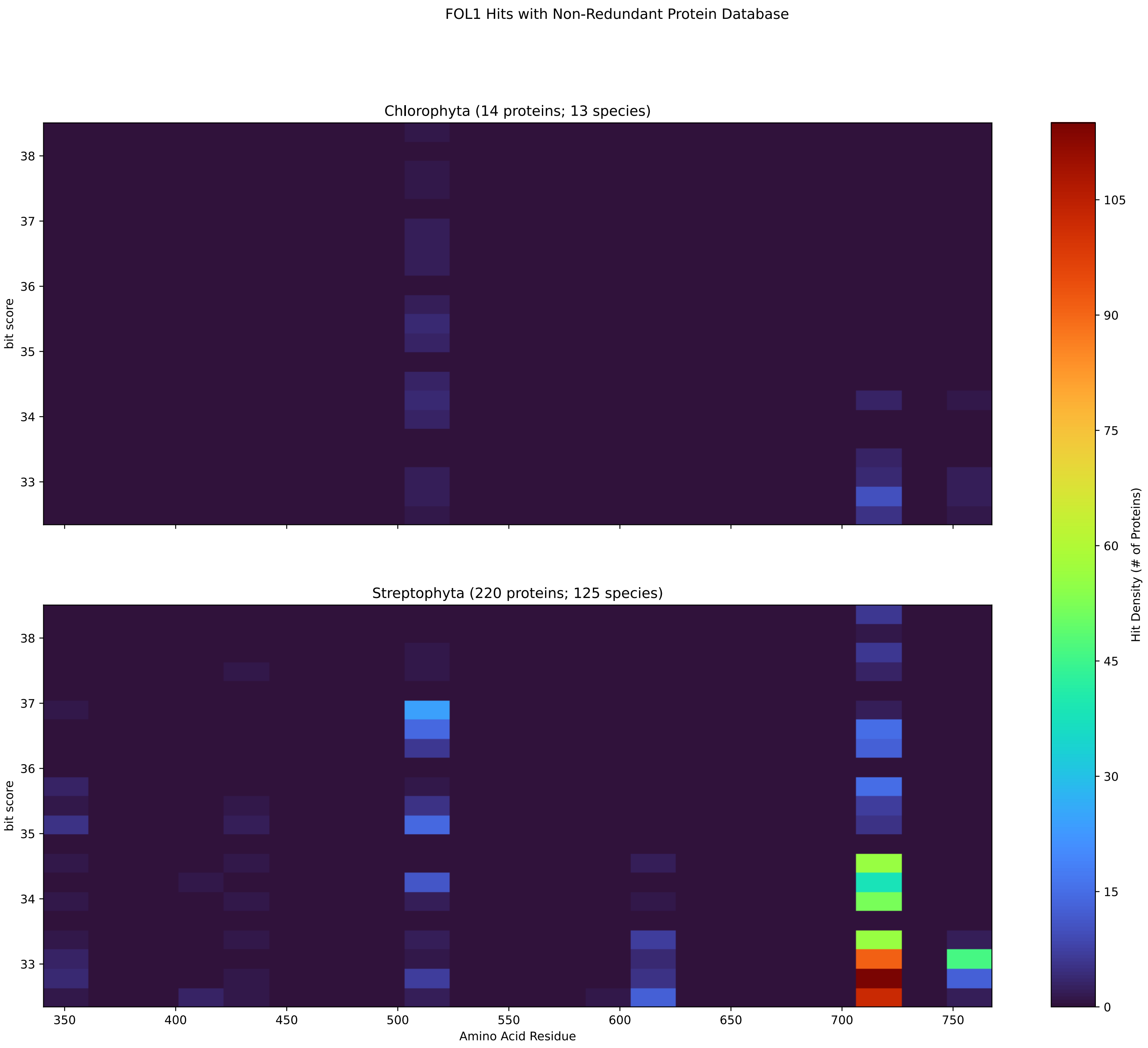

Figure S101: Non-redundant (NR) protein hits for Fol1 in the kingdom Viridiplantae.

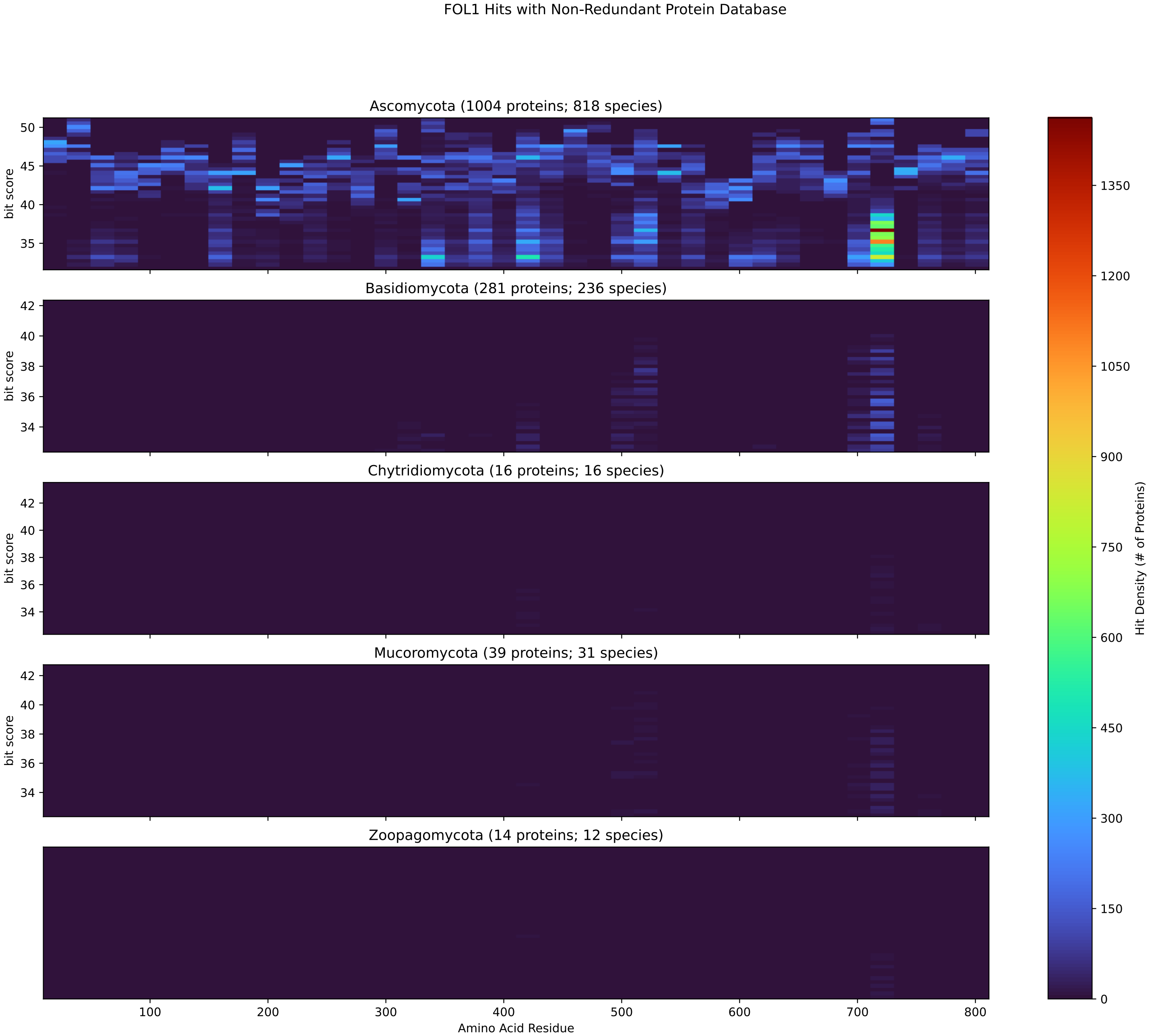

Figure S102: Non-redundant (NR) protein hits for Fol1 in the kingdom Fungi.

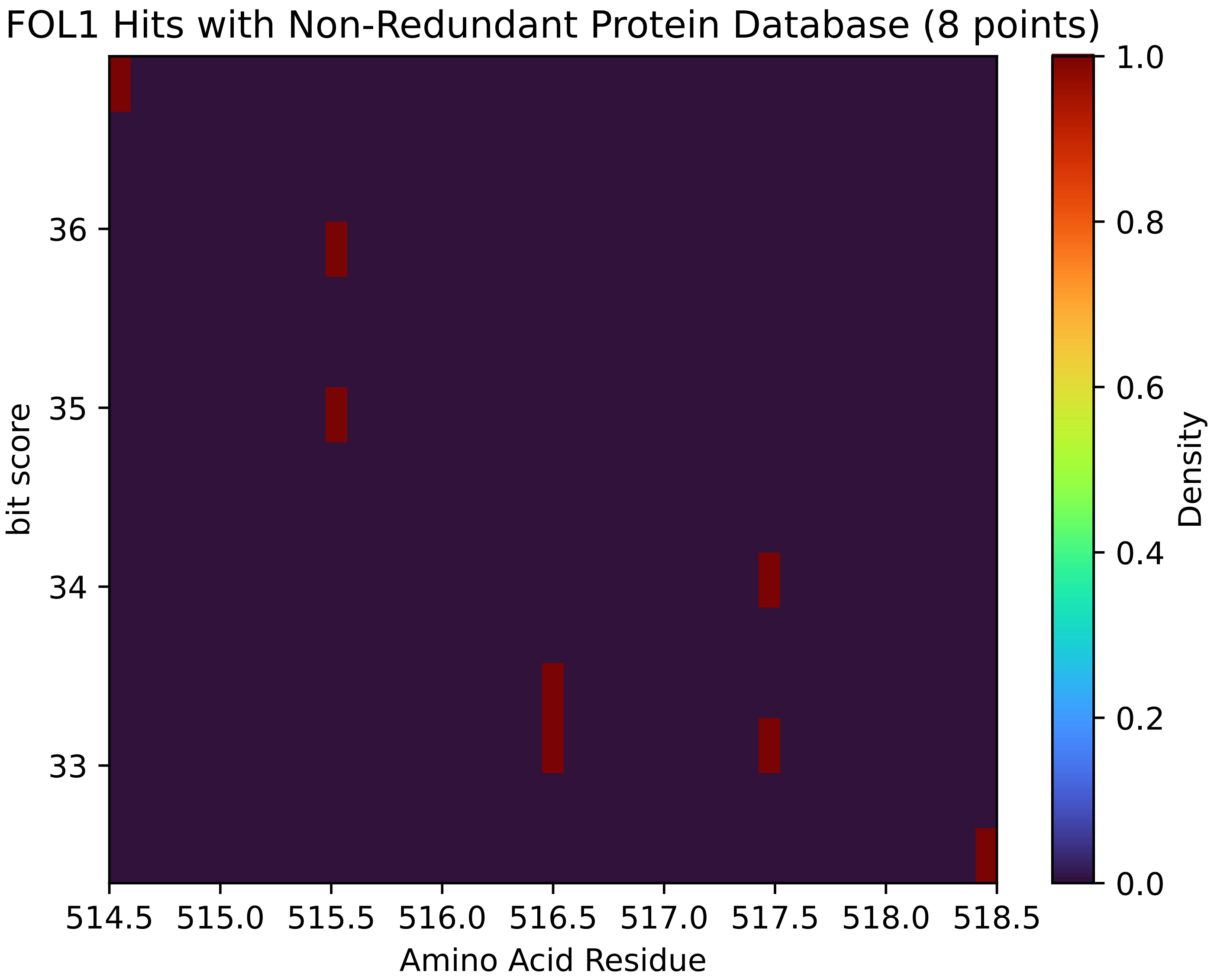

Figure S103: Non-redundant (NR) protein hits for Fol1 in the kingdom Metazoa.

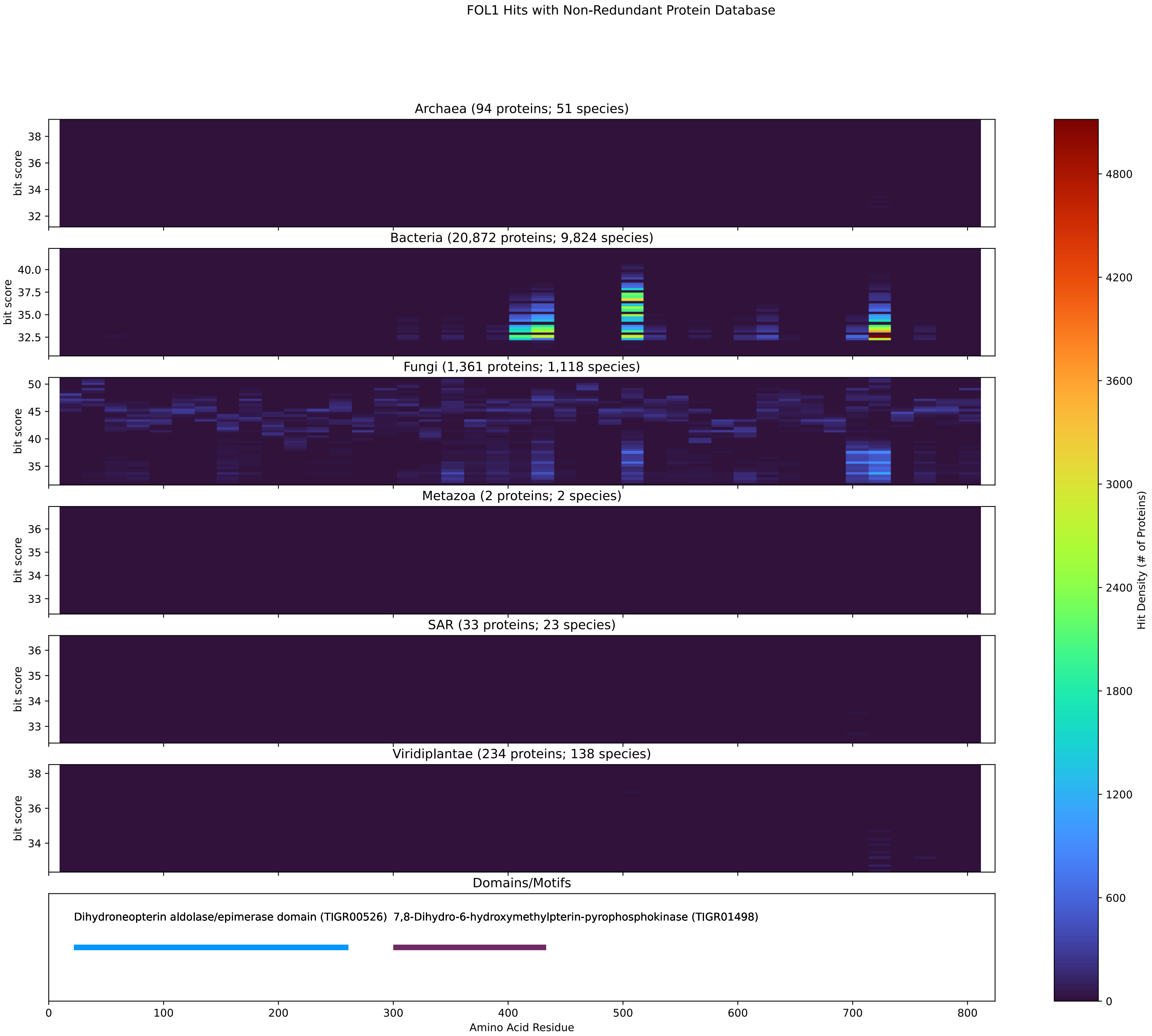

Figure S104: Non-redundant (NR) protein hits for DEG20010889/Fol1 at 20 amino acid length queries.

S2.10 Ilv3

S2.10.1 WHO Critical Pathogens

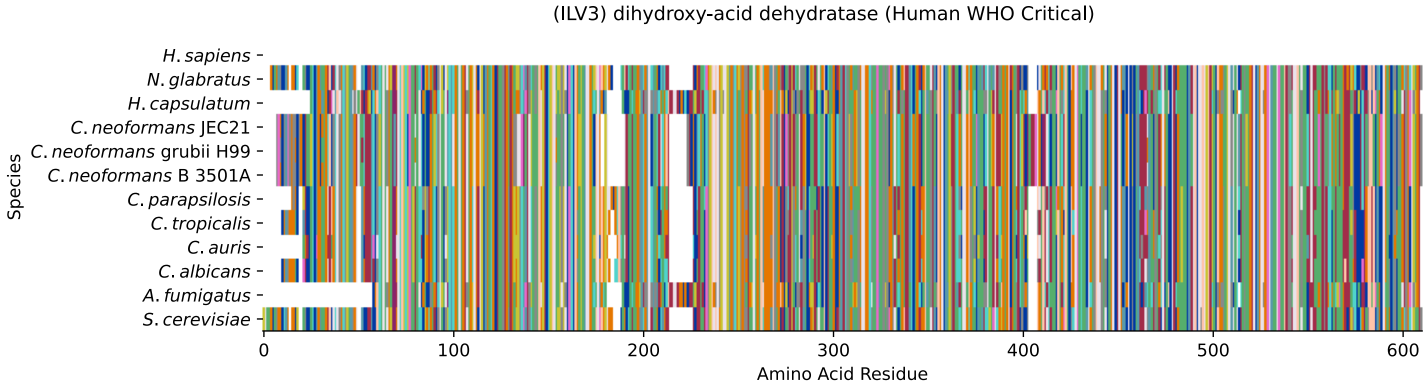

Figure S105: Multiple sequence alignment of yeast Ilv3 (WHO Critical Pathogens). Cf. Figure S106 for alignment quality, and Figure S107 for Sneath similarity. Cf. Table S24 for protein names, and pairwise alignment metrics with yeast Ilv3.

Ilv3 MSA Quality

	A. f.	*C. a.*	*C. a.*	*C. p.*	*C. t.*	*C. n.*	*C. n.*	*C. n.*	*H. c.*	*N. g.*	*S. c.*
A. fumigatus	1112.06	611.683	623.239	620.157	616.305	586.26	588.186	586.26	960.674	618.616	617.461
C. albicans	597.045	1187.17	989.564	1038.1	1115.52	681.019	683.33	681.019	626.32	897.886	882.863
C. auris	608.986	989.564	1164.06	987.638	993.801	673.315	676.011	673.315	646.351	893.264	877.47
C. parapsilosis	605.134	1038.1	987.638	1180.24	1039.25	669.078	673.315	669.078	640.188	877.856	879.782
C. tropicalis	600.897	1115.52	993.801	1039.25	1186.02	690.263	694.115	690.263	632.098	889.797	885.56
C. neoformans B 3501A	566.229	681.019	673.315	669.078	690.263	1204.5	1190.64	1204.5	600.127	681.019	691.034
C. neoformans grubii H99	568.155	683.33	676.011	673.315	694.115	1190.64	1205.66	1190.64	602.823	685.641	692.96
C. neoformans JEC21	566.229	681.019	673.315	669.078	690.263	1204.5	1190.64	1204.5	600.127	681.019	691.034
H. capsulatum	936.406	626.32	646.351	640.188	632.098	600.127	602.823	600.127	1181.39	622.468	634.41
N. glabratus	604.364	897.886	893.264	877.856	889.797	681.019	685.641	681.019	622.468	1202.58	1050.04
S. cerevisiae	602.823	882.863	877.47	879.782	885.56	691.034	692.96	691.034	634.41	1050.04	1210.28

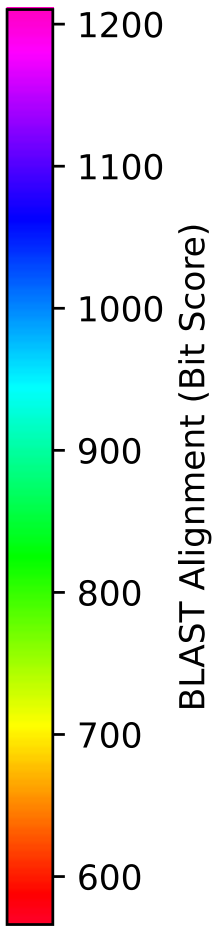

Figure S106: Multiple sequence alignment quality of Ilv3 (WHO Critical Pathogens). Cf. Figure S105

Species	Hit Protein	Hit Length (a.a.)	evalue	align_len	bit_score	identity	positive	score	gaps	% identity	% positive
H.sapiens	-	-	-	-	-	-	-	-	-	-	-
N.glabratus	XP_445144.1 uncharacterized protein CAGL0B03993g Nakaseomyces glabratus	581	0	581	1051.2	495	538	2717	0	84.6	92.0
H.capsulatum	XP_045291535.1 dihydroxy-acid dehydratase Histoplasma capsulatum G186AR	576	0	576	634.024	313	406	1634	19	53.5	69.4
C.neoformans.JEC21	XP_572335.1 dihydroxy-acid dehydratase, putative Cryptococcus neoformans var. neoformans JEC21	590	0	590	692.189	341	433	1785	18	58.3	74.0
C.neoformans.grubii.H99	XP_012053809.1 dihydroxy-acid dehydratase Cryptococcus neoformans var. grubii H99	590	0	590	694.115	343	437	1790	18	58.6	74.7
C.neoformans.B.3501A	XP_772279.1 hypothetical protein CNBL1470 Cryptococcus neoformans var. neoformans B-3501A	590	0	590	692.189	341	433	1785	18	58.3	74.0
C.parapsilosis	XP_036663159.1 uncharacterized protein CPAR2 100130 Candida parapsilosis	575	0	575	880.167	420	486	2273	5	71.8	83.1
C.tropicalis	XP_002546669.1 dihydroxy-acid dehydratase, mitochondrial precursor Candida tropicalis MYA-3404	577	0	577	884.789	420	490	2285	3	71.8	83.8
C.auris	XP_028892424.1 dihydroxy-acid dehydratase, mitochondrial Candida auris	566	0	566	878.241	410	486	2268	0	70.1	83.1
C.albicans	XP_721948.1 dihydroxy-acid dehydratase Candida albicans SC5314	577	0	577	882.093	418	486	2278	3	71.5	83.1
A.fumigatus	XP_750105.1 mitochondrial dihydroxy acid dehydratase, putative Aspergillus fumigatus Af293	543	0	543	602.438	301	385	1552	17	51.5	65.8

Table S24: Pairwise alignment info from yeast Ilv3 (DEG20010579), cf. Figure S105.

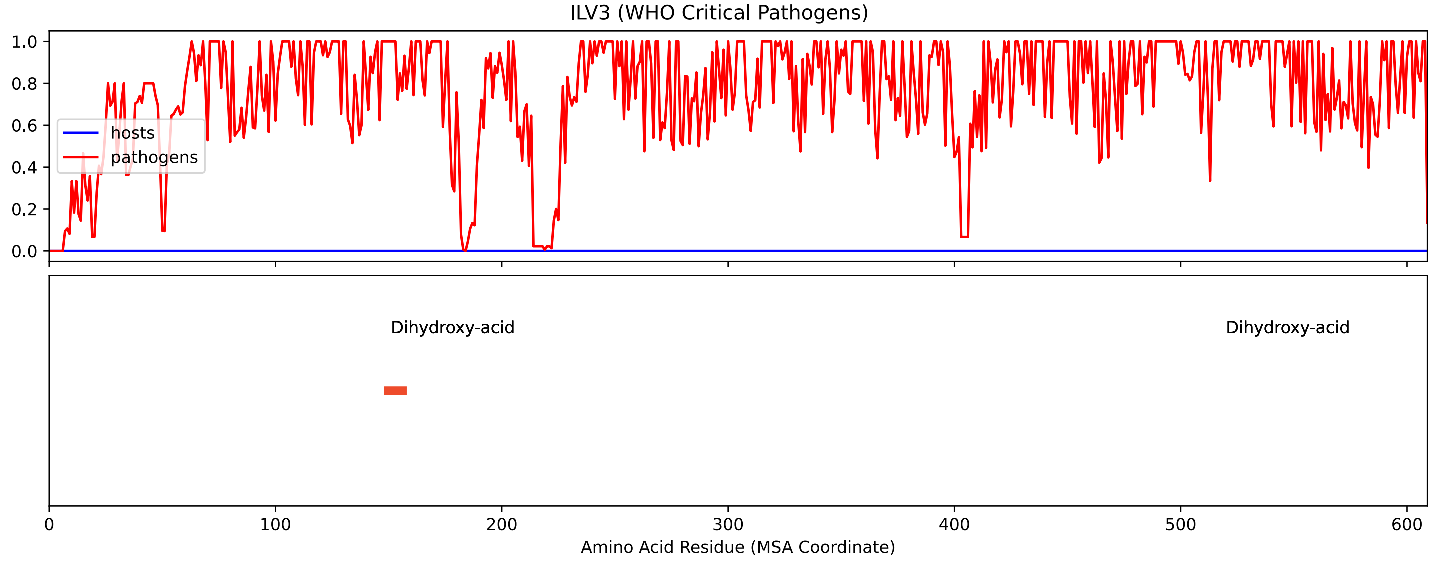

Figure S107: Sneath Similarity of Ilv3 for WHO Critical Pathogens, cf. Figure S105

S2.10.2 Top 10 Agricultural Fungal Pathogens

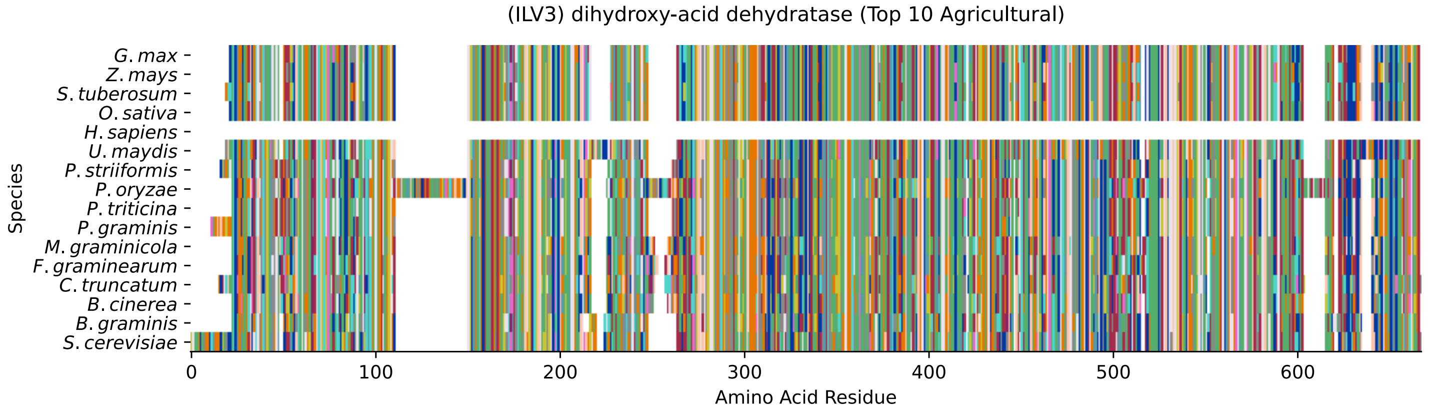

Figure S108: Multiple sequence alignment of yeast Ilv3 (Top 10 Agricultural Fungal Pathogens). Cf. Figure S109 for alignment quality, and Figure S110 for Sneath similarity. Cf. Table S25 for protein names, and pairwise alignment metrics with yeast Ilv3.

Ilv3 MSA Quality

	B. g.	*B. c.*	*C. t.*	*F. g.*	*M. g.*	*P. g.*	*P. t.*	*P. o.*	*P. s.*	*U. m.*	*S. c.*
B. graminis	1144.8	629.787	583.948	639.032	614.764	682.559	685.256	609.372	680.248	631.328	754.977
B. cinerea	629.787	1174.07	759.985	880.937	867.84	577.4	577.015	873.233	572.778	609.372	620.928
C. truncatum	597.816	776.933	1193.33	765.763	811.216	561.607	557.37	717.227	558.91	584.334	605.134
F. graminearum	639.032	880.937	749.584	1182.16	824.313	621.698	632.098	917.531	620.542	627.091	639.032
M. graminicola	614.764	867.84	793.112	824.313	1170.61	595.119	590.497	792.727	593.578	584.719	622.854
P. graminis	702.975	598.201	569.311	643.654	616.305	1167.14	1076.62	597.816	1108.98	621.698	681.789
P. triticina	706.442	598.971	563.918	653.67	611.298	1076.62	1145.18	595.89	1106.28	633.254	692.189
P. oryzae	609.372	873.233	705.286	917.531	792.727	577.015	574.704	1287.71	571.622	579.711	593.578
P. striiformis	701.049	593.578	565.844	640.958	615.535	1108.98	1106.28	592.808	1153.66	622.854	682.174
U. maydis	631.328	609.372	569.311	627.091	584.719	599.742	610.912	579.711	600.512	1181.01	634.41
S. cerevisiae	754.977	620.928	593.193	639.032	622.854	663.685	673.315	593.578	663.3	634.41	1210.28

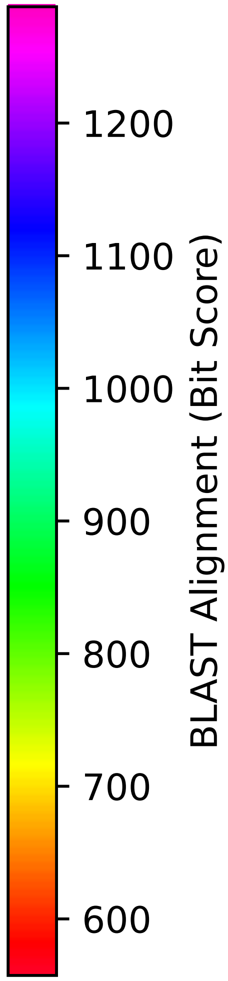

Figure S109: Multiple sequence alignment quality of Ilv3 (Top 10 Agricultural Fungal Pathogens). Cf. Figure S108

Species	Hit Protein	Hit Length (a.a.)	evalue	align_len	bit_score	identity	positive	score	gaps	% identity	% positive
G.max	NP_001276144.1 putative dihydroxy-acid dehydratase, mitochondrial-like Glycine max	564	0	564	686.797	335	416	1771	9	57.3	71.1
Z.mays	NP_001141560.1 Dihydroxy-acid dehydratase chloroplastic Zea mays	564	0	564	682.945	335	415	1761	9	57.3	70.9
S.tuberosum	XP_006346583.1 PREDICTED: dihydroxy-acid dehydratase, chloroplastic-like Solanum tuberosum	566	0	566	679.863	331	418	1753	9	56.6	71.5
O.sativa	XP_015649747.1 dihydroxy-acid dehydratase, chloroplastic Oryza sativa Japonica Group	564	0	564	689.108	334	420	1777	9	57.1	71.8
H.sapiens	-	-	-	-	-	-	-	-	-	-	-
U.maydis	XP_011389372.1 putative dihydroxy-acid dehydratase Ustilago maydis 521	577	0	577	634.41	315	401	1635	15	53.8	68.5
P.striiformis	XP_047805603.1 hypothetical protein Pst134EA 015851 Puccinia striiformis f. sp. tritici	573	0	573	662.529	336	414	1708	10	57.4	70.8
P.oryzae	mRNA M BR32 EuGene 00030511-p1 — transcript=mRNA M BR32 EuGene 00030511 — gene=M BR32 EuGene 00030511 — organism=Pyricularia oryzae BR32 — gene product=unspecified product — transcript product=unspecified product — location=BR32 scaffold00002:4030791-4032964(+) — protein length=660 — sequence SO=supercontig — SO=protein coding gene — is pseudo=false	631	0	631	592.808	311	400	1527	74	53.2	68.4
P.triticina	XP_053023065.1 uncharacterized protein PtA15 8A414 Puccinia triticina	567	0	567	673.315	341	415	1736	10	58.3	70.9
P.graminis	XP_003327990.2 dihydroxy-acid dehydratase Puccinia graminis f. sp. tritici CRL 75-36-700–3	578	0	578	663.685	337	417	1711	10	57.6	71.3
M.graminicola	ZTRI 1.138.mRNA-p1 — transcript=ZTRI 1.138.mRNA — gene=ZTRI 1.138 — organism=Zymoseptoria tritici IPO323 — gene product=similar to dihydroxy-acid dehydratase — transcript product=similar to dihydroxy-acid dehydratase — location=Ztri chr 1:550543-552333(+) — protein length=596 — sequence SO=chromosome — SO=protein coding gene — is pseudo=false	573	0	573	622.468	310	398	1604	16	53.0	68.0
F.graminearum	XP_011318673.1 dihydroxy-acid dehydratase Fusarium graminearum PH-1	577	0	577	638.262	317	405	1645	20	54.2	69.2
C.truncatum	XP_036575211.1 dihydroxy-acid dehydratase Colletotrichum truncatum	583	0	583	592.423	304	410	1526	18	52.0	70.1
B.cinerea	XP_024553827.1 hypothetical protein BCIN 16g02700 Botrytis cinerea B05.10	572	0	572	619.387	303	401	1596	15	51.8	68.5
B.graminis	VDB86357.1 — transcript=BGT96224V316 LOCUS3920 t1 — gene=BGT96224V316 LOCUS3920 — organism=Blumeria graminis f. sp. tritici 96224 — gene product=unspecified product — transcript product=unspecified product — location=LR026988:19021675-19023529(+) — protein length=601 — sequence SO=chromosome — SO=protein coding gene — is pseudo=false	564	0	564	754.592	364	444	1947	5	62.2	75.9

Table S25: Pairwise alignment info from yeast Ilv3 (DEG20010579), cf. Figure S108.

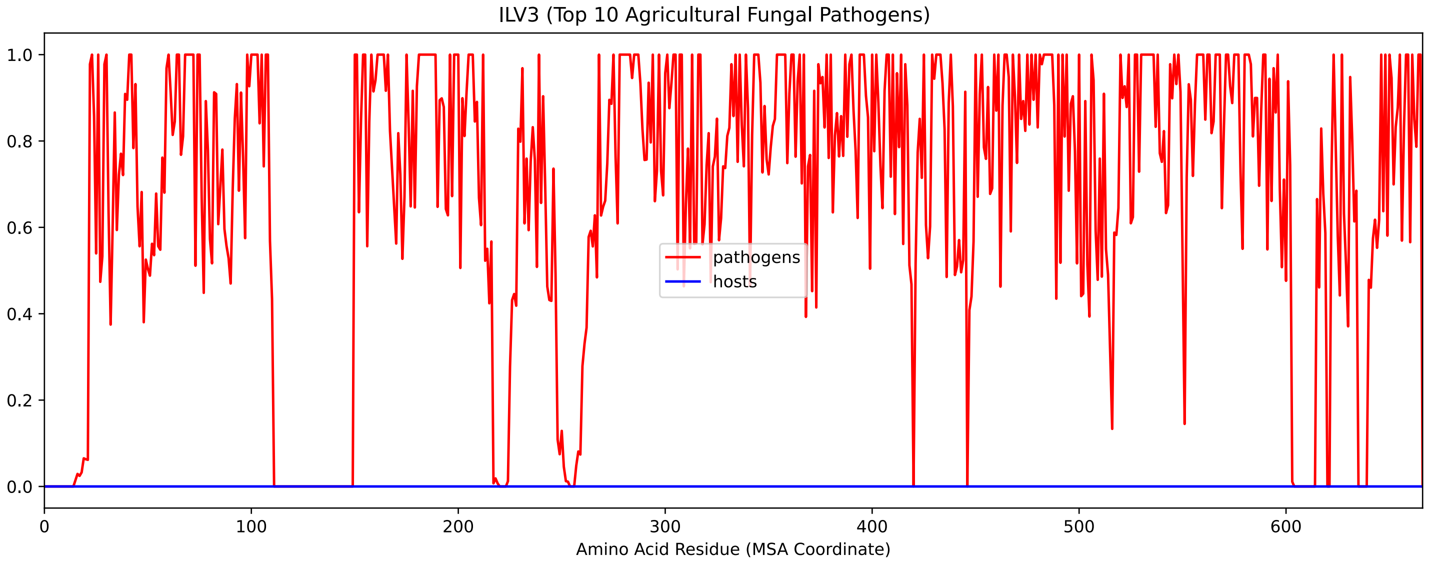

Figure S110: Sneath Similarity of Ilv3 for Top 10 Agricultural Fungal Pathogens, cf. Figure S108

S2.10.3 NR

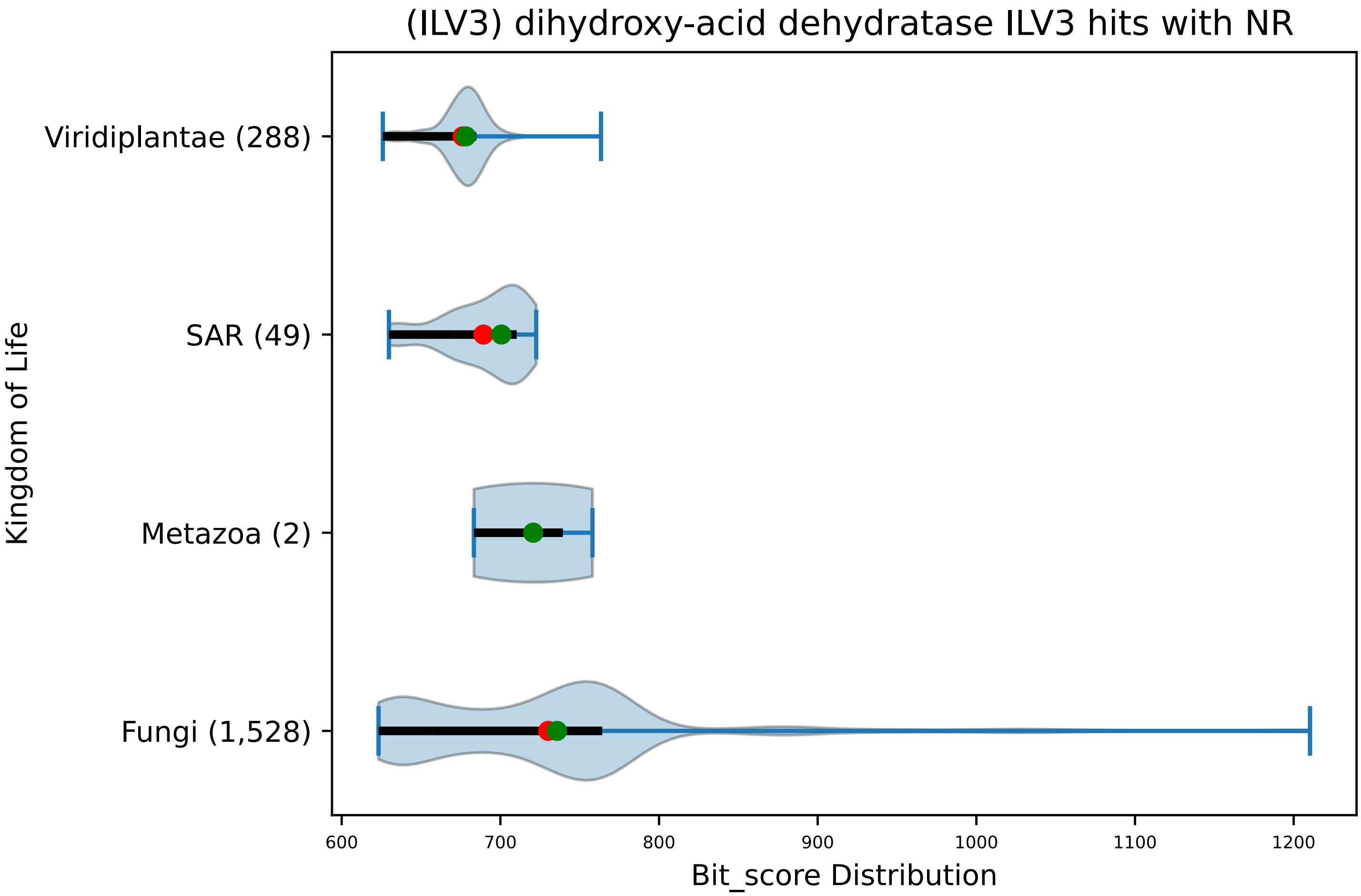

Figure S111: Non-redundant (NR) protein hits for DEG20010579/Ilv3, with expectation value of no more than 0.1. Green points are medians, and red points are arithmetic means.

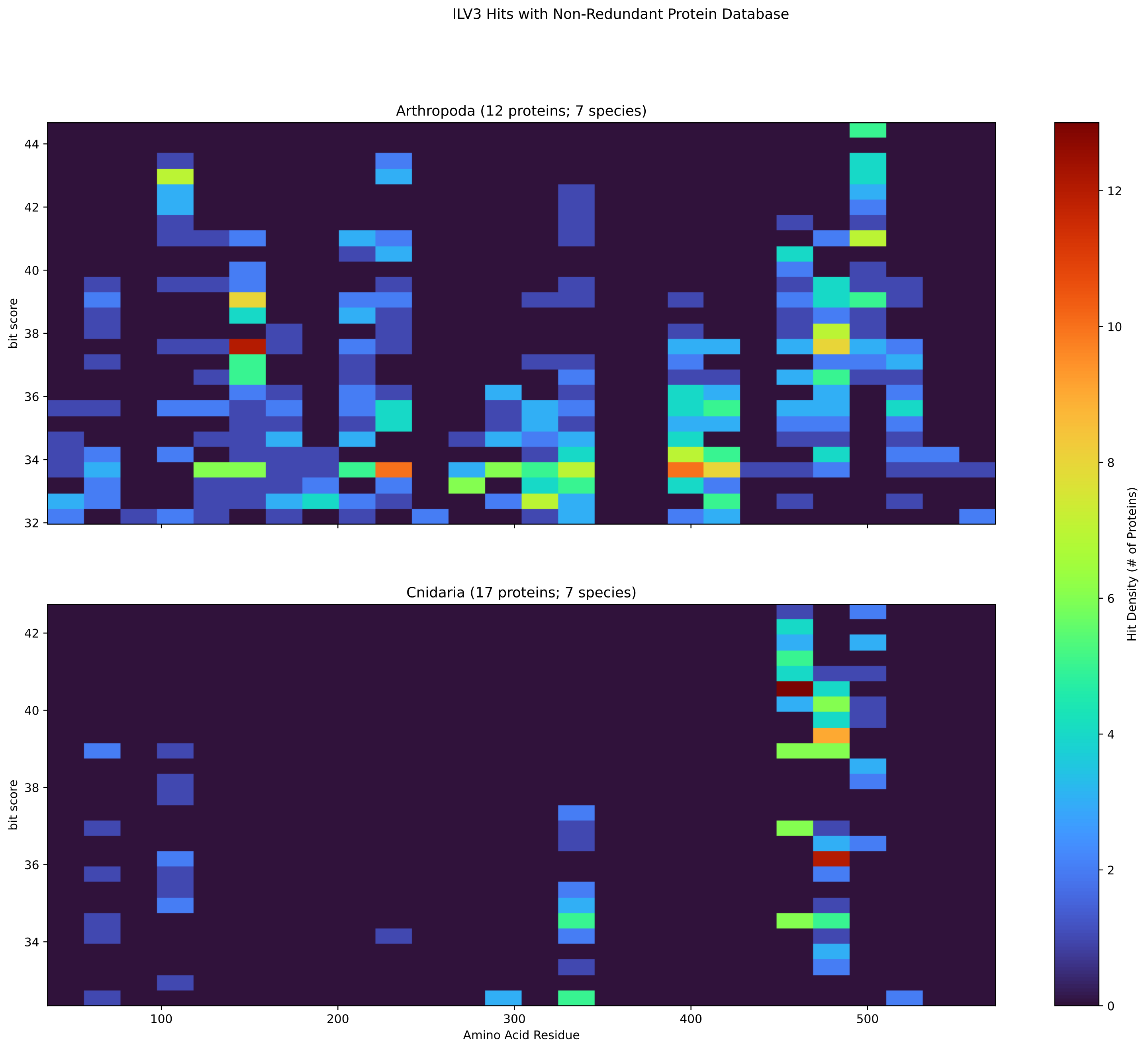

Figure S112: Non-redundant (NR) protein hits for Ilv3 in the kingdom Metazoa.

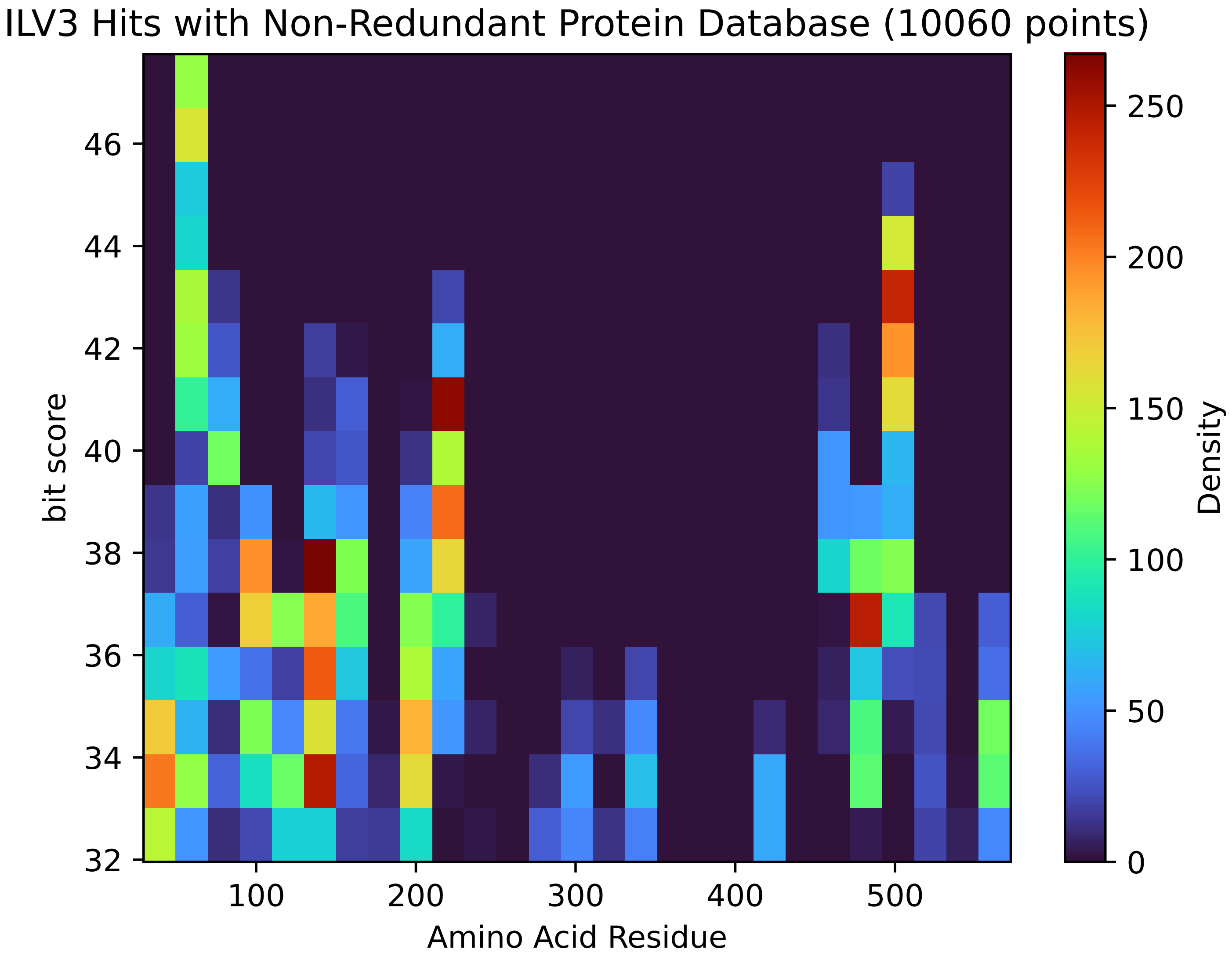

Figure S113: Non-redundant (NR) protein hits for Ilv3 in the kingdom SAR.

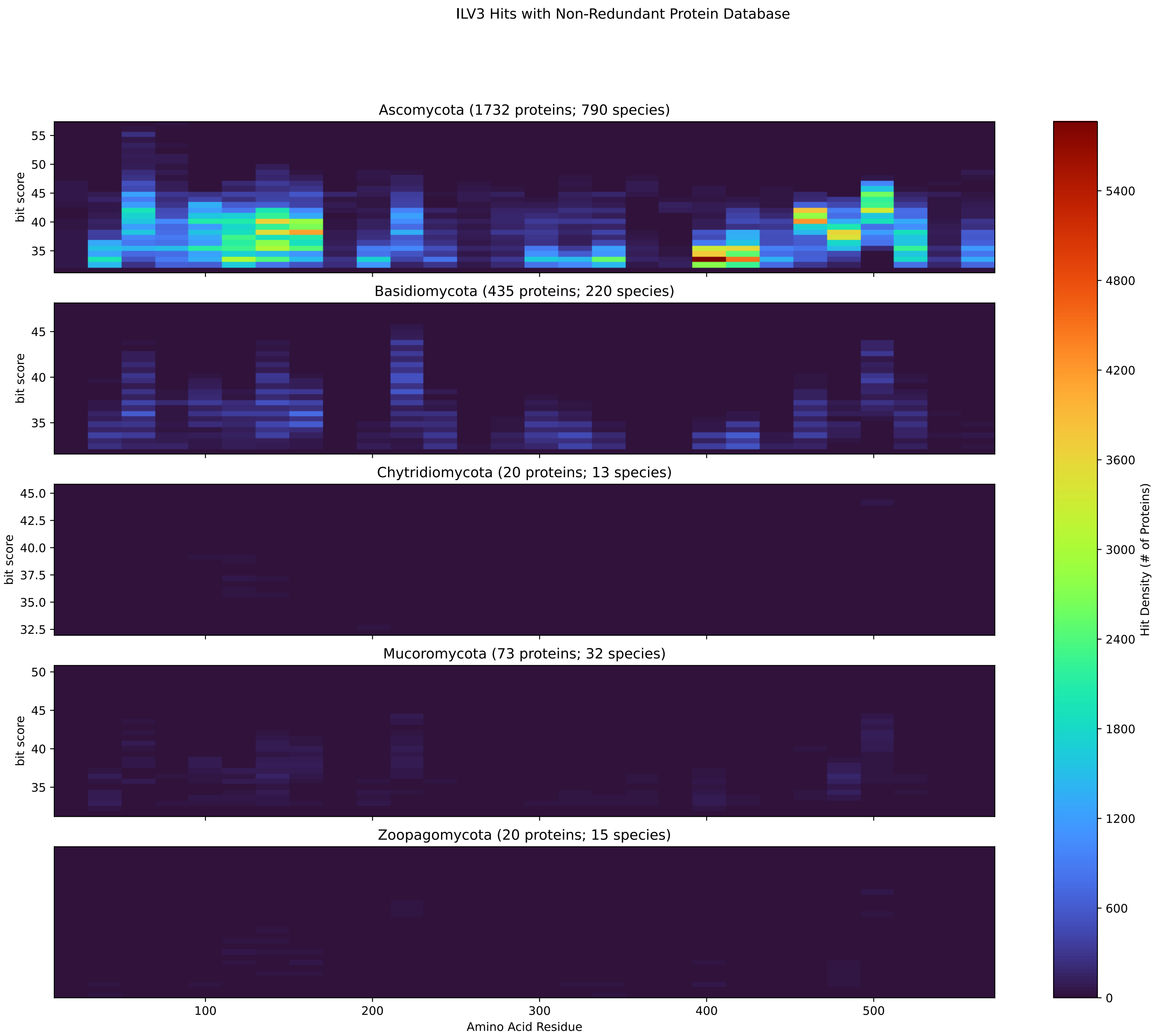

Figure S114: Non-redundant (NR) protein hits for Ilv3 in the kingdom Fungi.

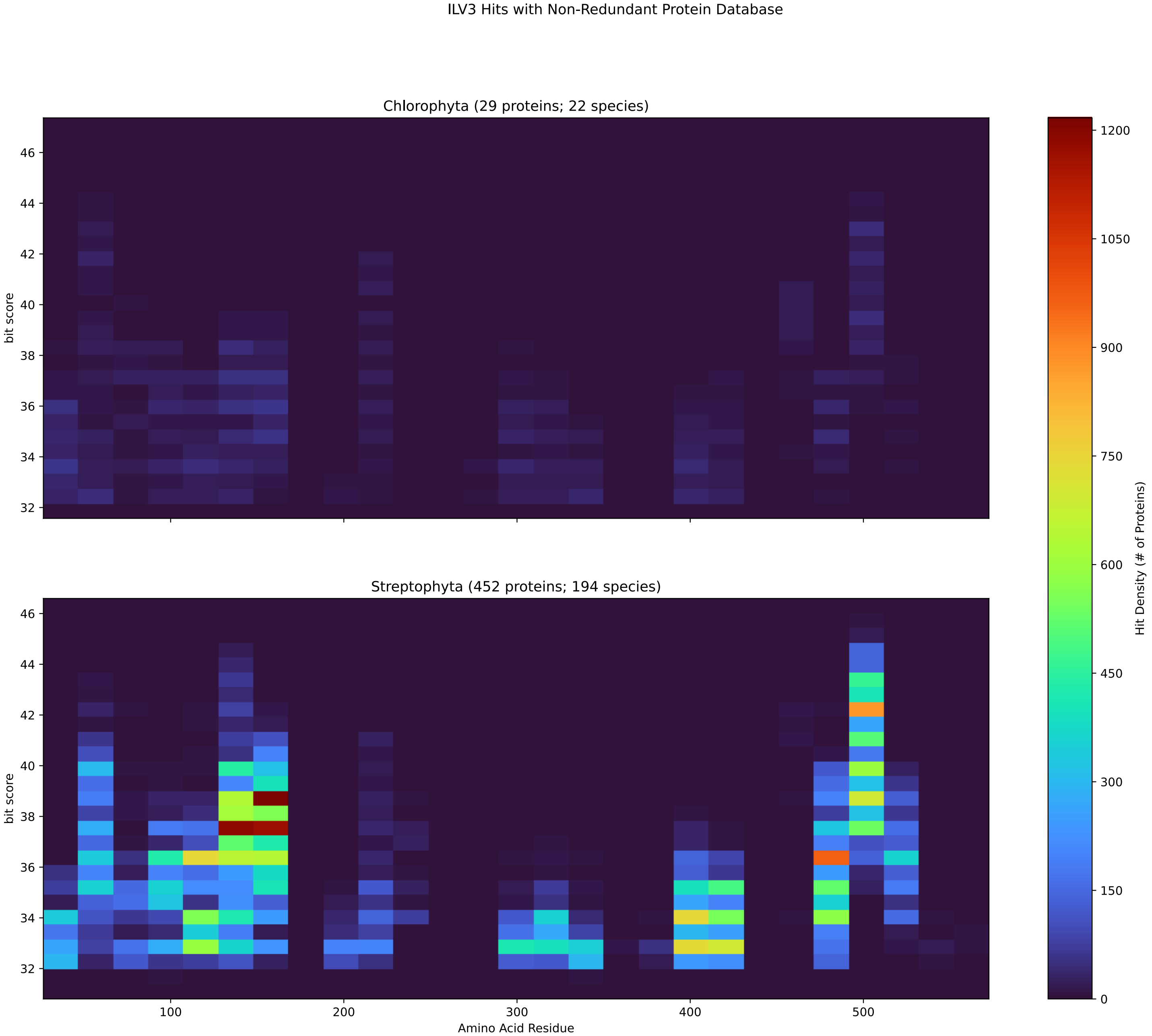

Figure S115: Non-redundant (NR) protein hits for Ilv3 in the kingdom Viridiplantae.

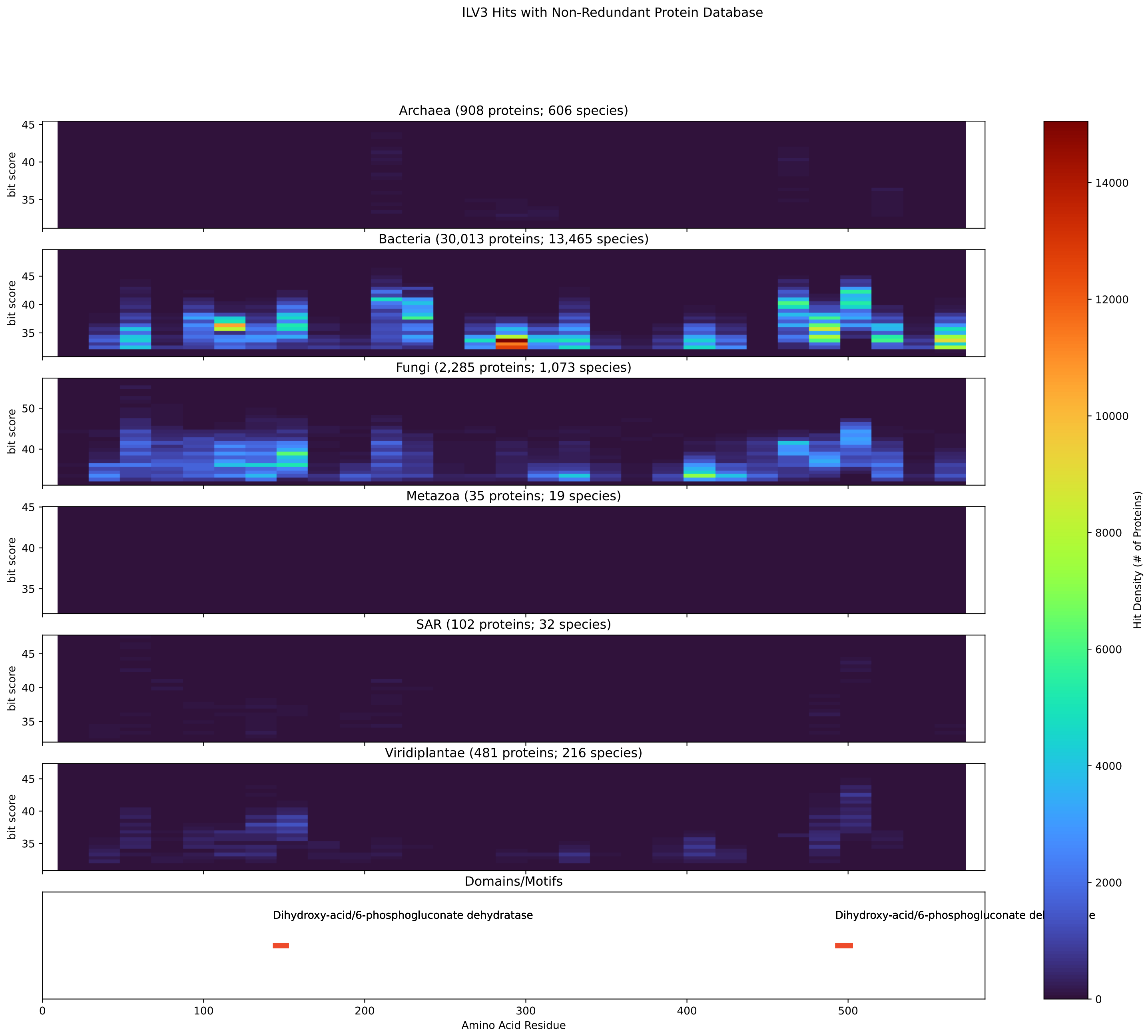

Figure S116: Non-redundant (NR) protein hits for DEG20010579/Ilv3 at 20 amino acid length queries.

S2.11 Ilv5

S2.11.1 WHO Critical Pathogens

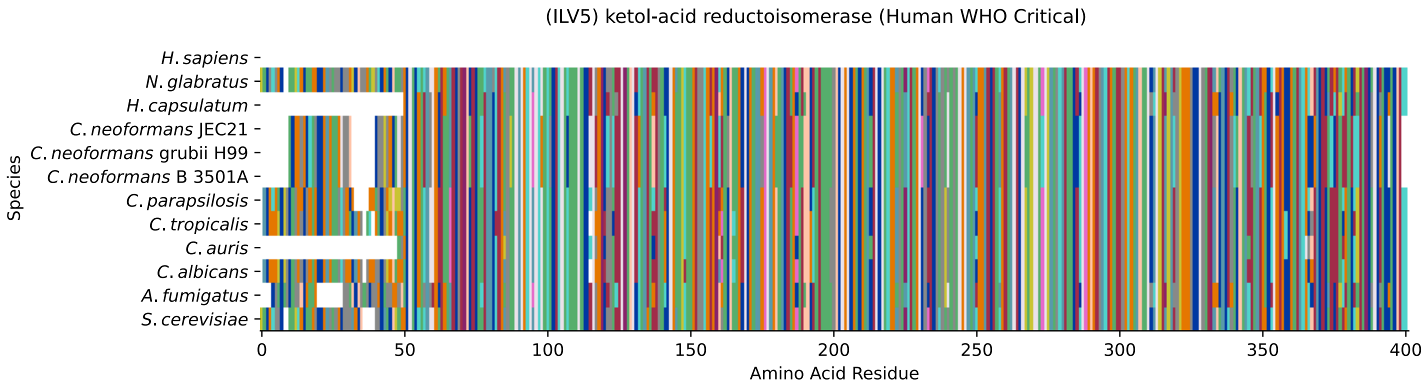

Figure S117: Multiple sequence alignment of yeast Ilv5 (WHO Critical Pathogens). Cf. Figure S118 for alignment quality, and Figure S119 for Sneath similarity. Cf. Table S26 for protein names, and pairwise alignment metrics with yeast Ilv5.

Ilv5 MSA Quality

	A. f.	*C. a.*	*C. a.*	*C. p.*	*C. t.*	*C. n.*	*C. n.*	*C. n.*	*H. c.*	*N. g.*	*S. c.*
A. fumigatus	799.66	560.451	555.058	582.022	554.673	574.704	574.704	574.704	685.256	577.015	569.696
C. albicans	584.719	825.083	624.78	717.613	755.747	561.607	561.992	561.607	586.645	650.203	637.106
C. auris	591.652	642.499	728.783	667.537	638.262	552.362	552.362	552.362	600.127	627.476	628.246
C. parapsilosis	607.06	717.613	646.736	819.691	708.368	572.778	573.163	572.778	605.134	663.685	658.292
C. tropicalis	579.326	755.747	620.928	708.368	822.002	558.14	558.525	558.14	573.933	637.876	621.313
C. neoformans B 3501A	574.704	541.576	517.694	552.747	537.724	786.178	785.793	786.178	560.836	536.954	534.258
C. neoformans grubii H99	574.704	541.962	518.079	553.132	538.11	785.793	786.178	785.793	560.451	537.724	534.258
C. neoformans JEC21	574.704	541.576	517.694	552.747	537.724	786.178	785.793	786.178	560.836	536.954	534.258
H. capsulatum	685.256	563.148	563.533	581.637	550.821	560.836	560.451	560.836	723.391	585.104	582.022
N. glabratus	601.282	650.588	611.298	664.455	639.032	558.525	558.525	558.525	608.216	830.091	731.865
S. cerevisiae	593.964	637.491	611.298	659.062	621.698	554.673	555.058	554.673	605.905	731.48	821.231

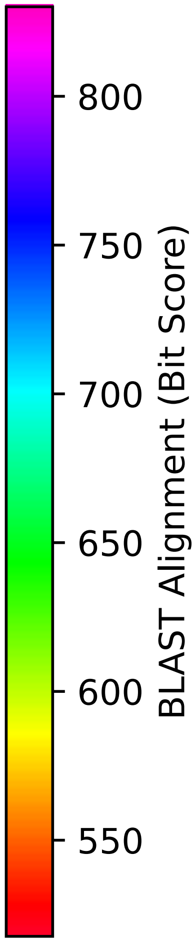

Figure S118: Multiple sequence alignment quality of Ilv5 (WHO Critical Pathogens). Cf. Figure S117

Species	Hit Protein	Hit Length (a.a.)	evalue	align_len	bit_score	identity	positive	score	gaps	% identity	% positive
H.sapiens	-	-	-	-	-	-	-	-	-	-	-
N.glabratus	XP_445105.1 uncharacterized p-rotein CAGL0B03047g Nakaseomyc-es glabratus	400	0	400	731.48	358	383	1887	6	90.6	97.0
H.capsulatum	XP_045283741.1 ketol-acid red-uctoisomerase Histoplasma caps-ulatum G186AR	351	0	351	606.675	287	315	1563	0	72.7	79.7
C.neoformans.JEC21	XP_571345.1 ketol-acid reduct-oisomerase, putative Cryptococ-cus neoformans var. neoformans JEC21	392	0	392	556.599	268	317	1433	11	67.8	80.3
C.neoformans.grubii.H99	XP_012050476.1 ketol-acid red-uctoisomerase, mitochondrial C-ryptococcus neoformans var. gr-ubii H99	392	0	392	556.599	268	317	1433	11	67.8	80.3
C.neoformans.B.3501A	XP_774791.1 hypothetical prot-ein CNBF2210 Cryptococcus neof-ormans var. neoformans B-3501A	392	0	392	556.599	268	317	1433	11	67.8	80.3
C.parapsilosis	XP_036663080.1 uncharacterize-d protein CPAR2 603140 Candida parapsilosis	396	0	396	659.062	322	358	1699	3	81.5	90.6
C.tropicalis	XP_002548652.1 ketol-acid red-uctoisomerase, mitochondrial p-recursor Candida tropicalis MY-A-3404	398	0	398	621.698	307	354	1602	5	77.7	89.6
C.auris	XP_028890742.2 ketol-acid red-uctoisomerase, mitochondrial C-andida auris	353	0	353	614.764	308	333	1584	0	78.0	84.3
C.albicans	XP_714297.2 ketol-acid reduct-oisomerase Candida albicans SC-5314	399	0	399	637.876	315	357	1644	6	79.7	90.4
A.fumigatus	XP_754177.1 Ketol-acid reduct-oisomerase Aspergillus fumigat-us Af293	388	0	388	595.89	279	325	1535	0	70.6	82.3

Table S26: Pairwise alignment info from yeast Ilv5 (DEG20010747), cf. Figure S117.

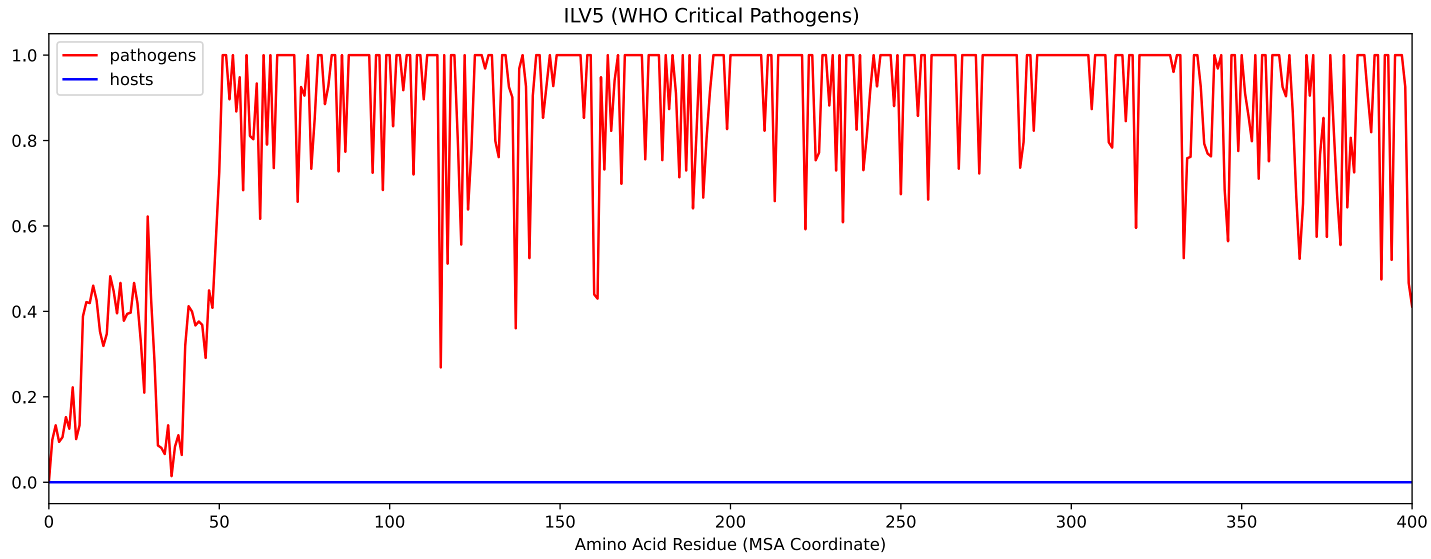

Figure S119: Sneath Similarity of Ilv5 for WHO Critical Pathogens, cf. Figure S117

S2.11.2 Top 10 Agricultural Fungal Pathogens

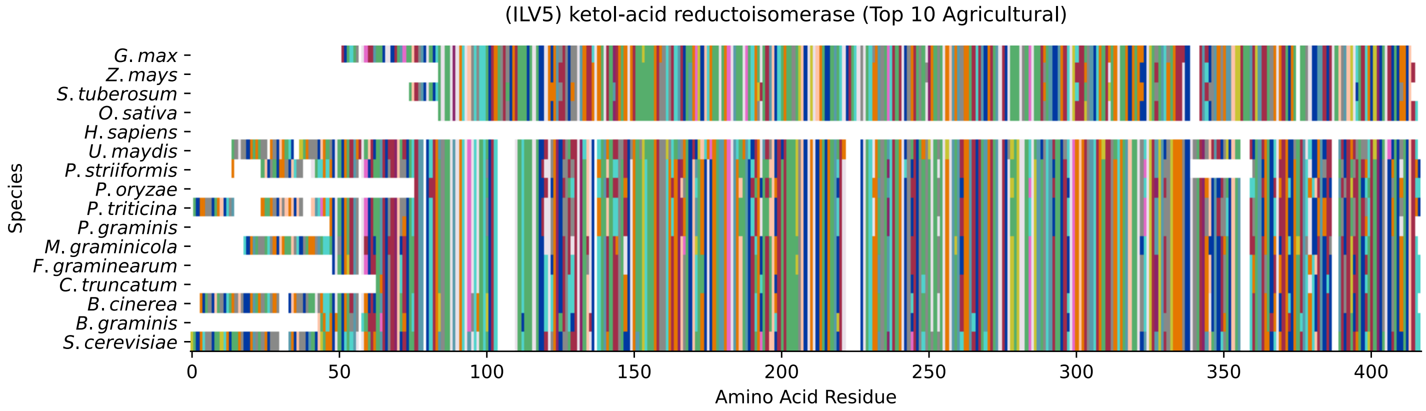

Figure S120: Multiple sequence alignment of yeast Ilv5 (Top 10 Agricultural Fungal Pathogens). Cf. Figure S121 for alignment quality, and Figure S122 for Sneath similarity. Cf. Table S27 for protein names, and pairwise alignment metrics with yeast Ilv5.

Ilv5 MSA Quality

	B. g.	*B. c.*	*C. t.*	*F. g.*	*M. g.*	*P. g.*	*P. t.*	*P. o.*	*P. s.*	*U. m.*	*S. c.*
B. graminis	738.799	662.144	600.897	613.609	600.512	534.258	533.487	586.26	504.212	469.929	526.939
B. cinerea	662.529	810.446	631.328	655.596	652.899	555.444	555.058	616.305	533.102	505.753	567
C. truncatum	600.897	630.943	697.197	654.825	605.52	530.02	532.717	621.313	493.426	485.337	553.518
F. graminearum	613.609	654.44	654.825	725.317	632.098	550.821	553.903	640.573	514.227	487.649	563.918
M. graminicola	600.512	640.573	605.52	632.098	783.097	550.436	555.444	587.03	518.85	494.582	550.436
P. graminis	534.258	554.673	530.02	550.821	550.436	718.768	711.064	502.671	666.766	509.22	510.76
P. triticina	533.487	554.673	533.102	553.518	555.444	711.064	794.653	505.753	677.937	516.153	520.005
P. oryzae	586.26	615.15	621.313	640.573	587.03	502.671	505.753	670.233	466.463	461.84	533.487
P. striiformis	504.212	523.857	493.426	514.227	518.85	666.766	673.7	466.463	730.709	471.085	476.863
U. maydis	469.929	496.508	485.337	487.649	494.582	509.22	514.998	461.84	471.085	791.571	477.633
S. cerevisiae	545.814	579.326	576.63	587.03	574.318	533.487	541.191	556.984	499.59	501.516	821.231

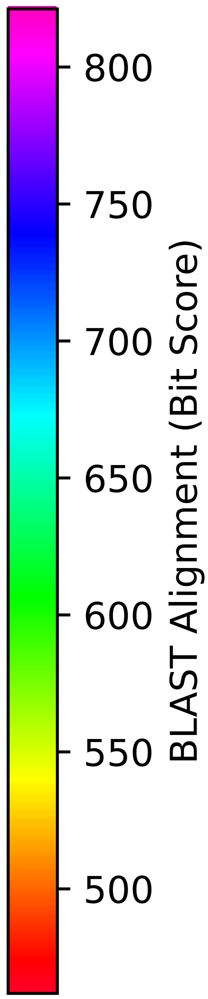

Figure S121: Multiple sequence alignment quality of Ilv5 (Top 10 Agricultural Fungal Pathogens). Cf. Figure S120

Species	Hit Protein	Hit Length (a.a.)	evalue	align_len	bit_score	identity	positive	score	gaps	% identity	% positive
G.max	NP_001276286.2 ketol-acid reductoisomerase, chloroplastic-like Glycine max	363	4e-43	363	159.844	115	179	403	24	29.1	45.3
Z.mays	NP_001169144.1 ketol-acid reductoisomerase, chloroplastic-like Zea mays	332	6e-44	332	160.614	109	169	405	23	27.6	42.8
S.tuberosum	XP_006348854.1 PREDICTED: ketol-acid reductoisomerase, chloroplastic Solanum tuberosum	341	8.8e-43	341	157.918	109	171	398	24	27.6	43.3
O.sativa	XP_015640421.1 ketol-acid reductoisomerase, chloroplastic Oryza sativa Japonica Group	333	2.3e-43	333	159.458	108	168	402	23	27.3	42.5
H.sapiens	-	-	-	-	-	-	-	-	-	-	-
U.maydis	XP_011391733.1 putative ketol--acid reductoisomerase Ustilago maydis 521	382	3.8e-179	382	503.056	243	290	1294	3	61.5	73.4
P.striiformis	XP_047803961.1 hypothetical protein Pst134EA 017323 Puccinia striiformis f. sp. tritici	375	2.9e-178	375	499.59	249	288	1285	21	63.0	72.9
P.oryzae	mRNA M BR32 EuGene 00014811-p1 — transcript=mRNA M BR32 EuGene 00014811 — gene=M BR32 EuGene 00014811 — organism=Pyricularia oryzae BR32 — gene product=unspecified product — transcript product=unspecified product — location=BR32 scaffold000-01:4410728-4411788(+) — protein length=324 — sequence SO=supercontig — SO=protein coding gene — is pseudo=false	323	0	323	556.984	260	293	1434	0	65.8	74.2
P.triticina	XP_053025405.1 uncharacterized protein PtA15 11A542 Puccinia triticina	394	0	394	540.421	268	309	1391	9	67.8	78.2
P.graminis	XP_003323363.1 ketol-acid reductoisomerase, mitochondrial Puccinia graminis f. sp. tritici CRL 75-36-700-3	349	0	349	535.798	254	290	1379	0	64.3	73.4
M.graminicola	ZTRI 1.395.mRNA-p1 — transcript=ZTRI 1.395.mRNA — gene=ZTRI 1.395 — organism=Zymoseptoria tritici IPO323 — gene product=-similar to ketol-acid reductoisomerase — transcript product=-similar to ketol-acid reductoisomerase — location=Ztri chr 1-:1345028-1346413(-) — protein length=403 — sequence SO=chromosome — SO=protein coding gene — is pseudo=false	382	0	382	576.244	275	316	1484	9	69.6	80.0
F.graminearum	XP_011319056.1 ketol-acid reductoisomerase Fusarium graminearum PH-1	350	0	350	588.956	278	310	1517	0	70.4	78.5
C.truncatum	XP_036585043.1 ketol-acid reductoisomerase Colletotrichum truncatum	336	0	336	576.63	269	305	1485	0	68.1	77.2
B.cinerea	XP_001557193.1 Bcilv5 Botrytis cinerea B05.10	391	0	391	579.711	277	320	1493	0	70.1	81.0
B.graminis	VCU40915.1 — transcript=BGT962-24V316 LOCUS2166 t1 — gene=BGT-96224V316 LOCUS2166 — organism=Blumeria graminis f. sp. tritici 96224 — gene product=unspecified product — transcript product=unspecified product — location=LR026987:2357263-235874-1(+) — protein length=402 — sequence SO=chromosome — SO=protein coding gene — is pseudo=false	355	0	355	547.74	255	297	1410	0	64.6	75.2

Table S27: Pairwise alignment info from yeast Ilv5 (DEG20010747), cf. Figure S120.

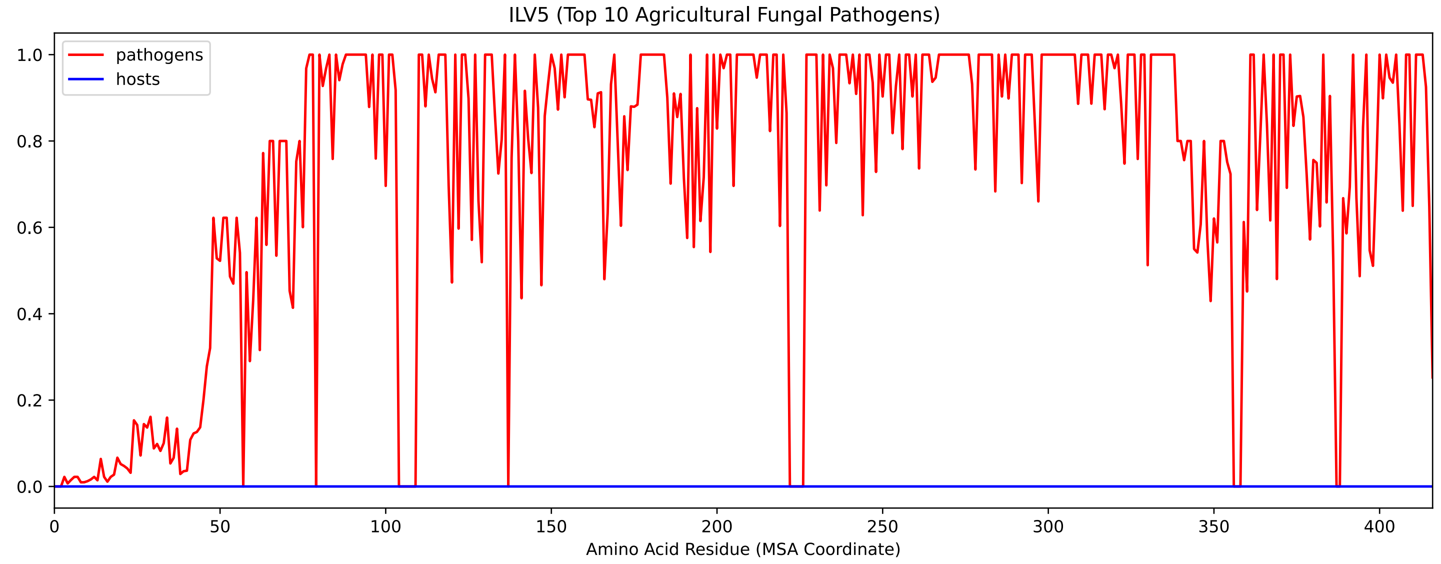

Figure S122: Sneath Similarity of Ilv5 for Top 10 Agricultural Fungal Pathogens, cf. Figure S120

S2.11.3 NR

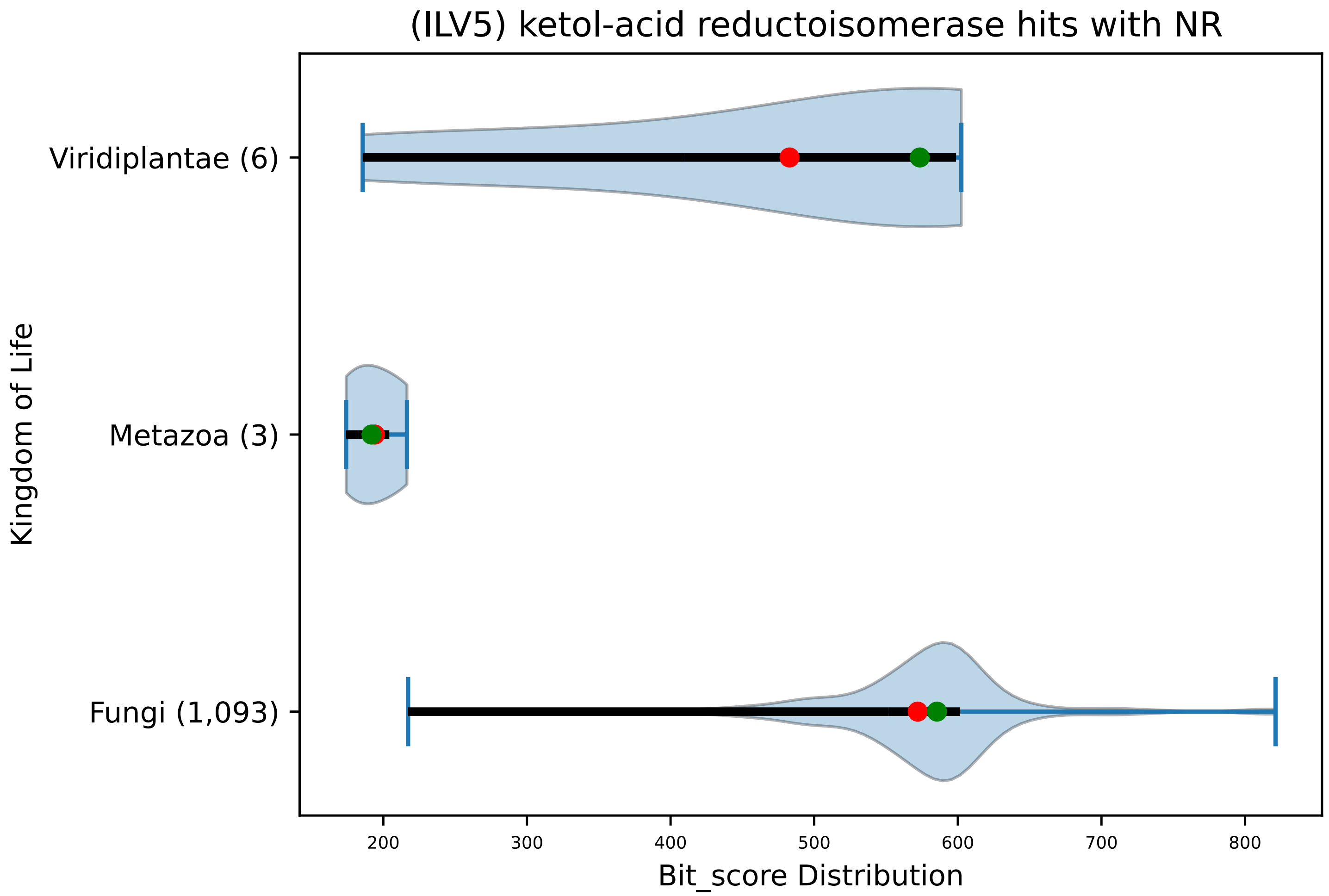

Figure S123: Non-redundant (NR) protein hits for DEG20010747/Ilv5, with expectation value of no more than 0.1. Green points are medians, and red points are arithmetic means.

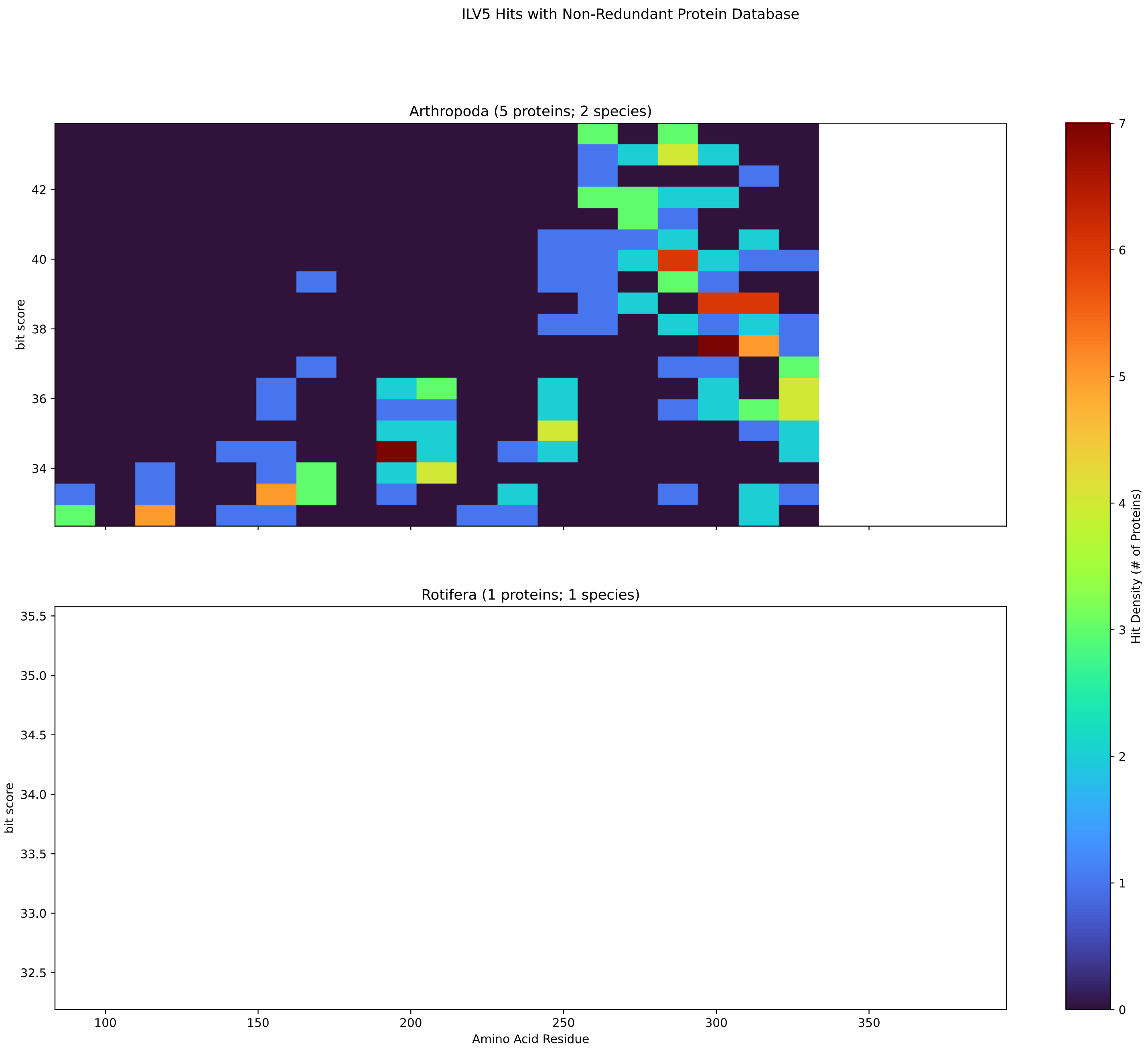

Figure S124: Non-redundant (NR) protein hits for Ilv5 in the kingdom Metazoa.

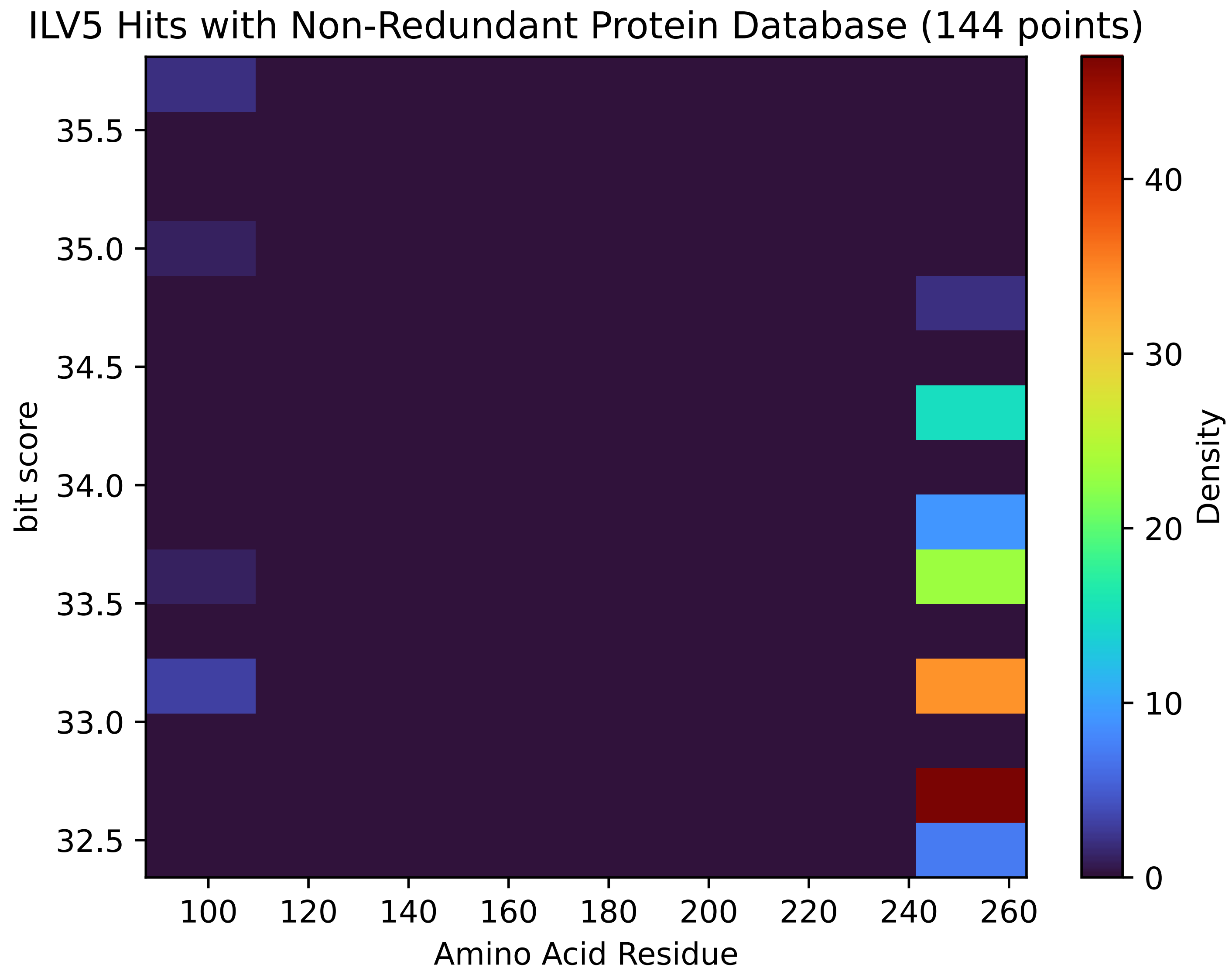

Figure S125: Non-redundant (NR) protein hits for Ilv5 in the kingdom SAR.

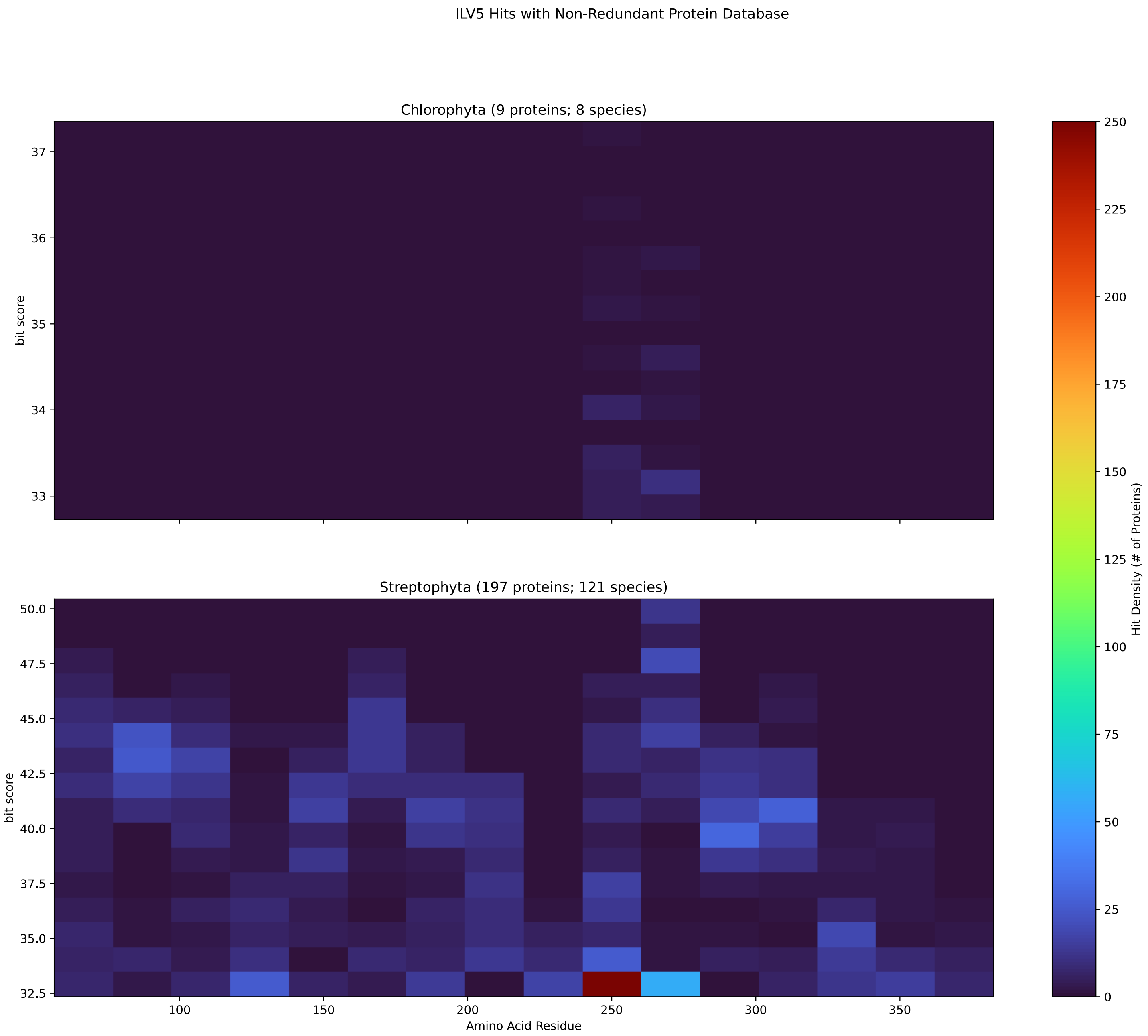

Figure S126: Non-redundant (NR) protein hits for Ilv5 in the kingdom Viridiplantae.

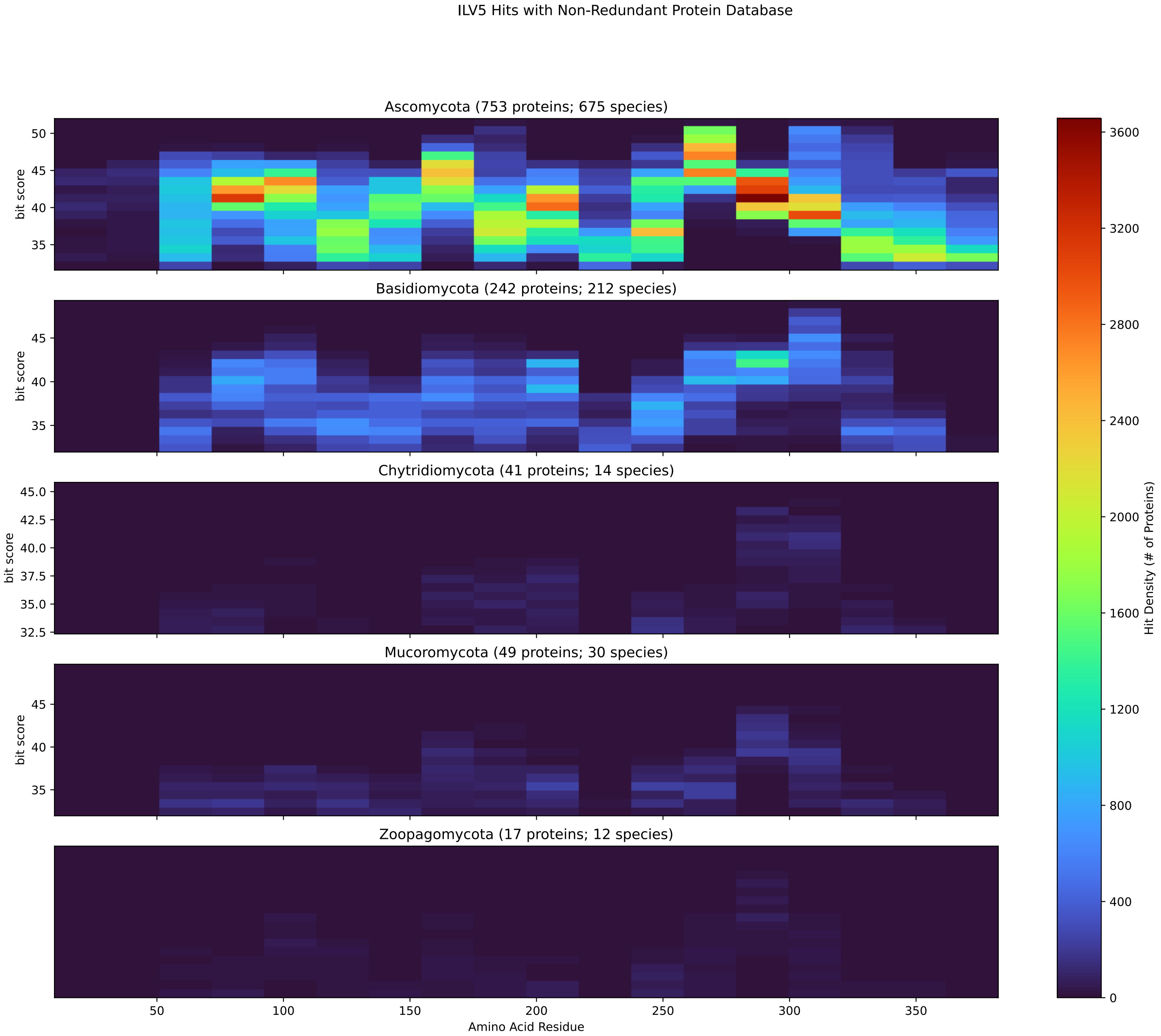

Figure S127: Non-redundant (NR) protein hits for Ilv5 in the kingdom Fungi.

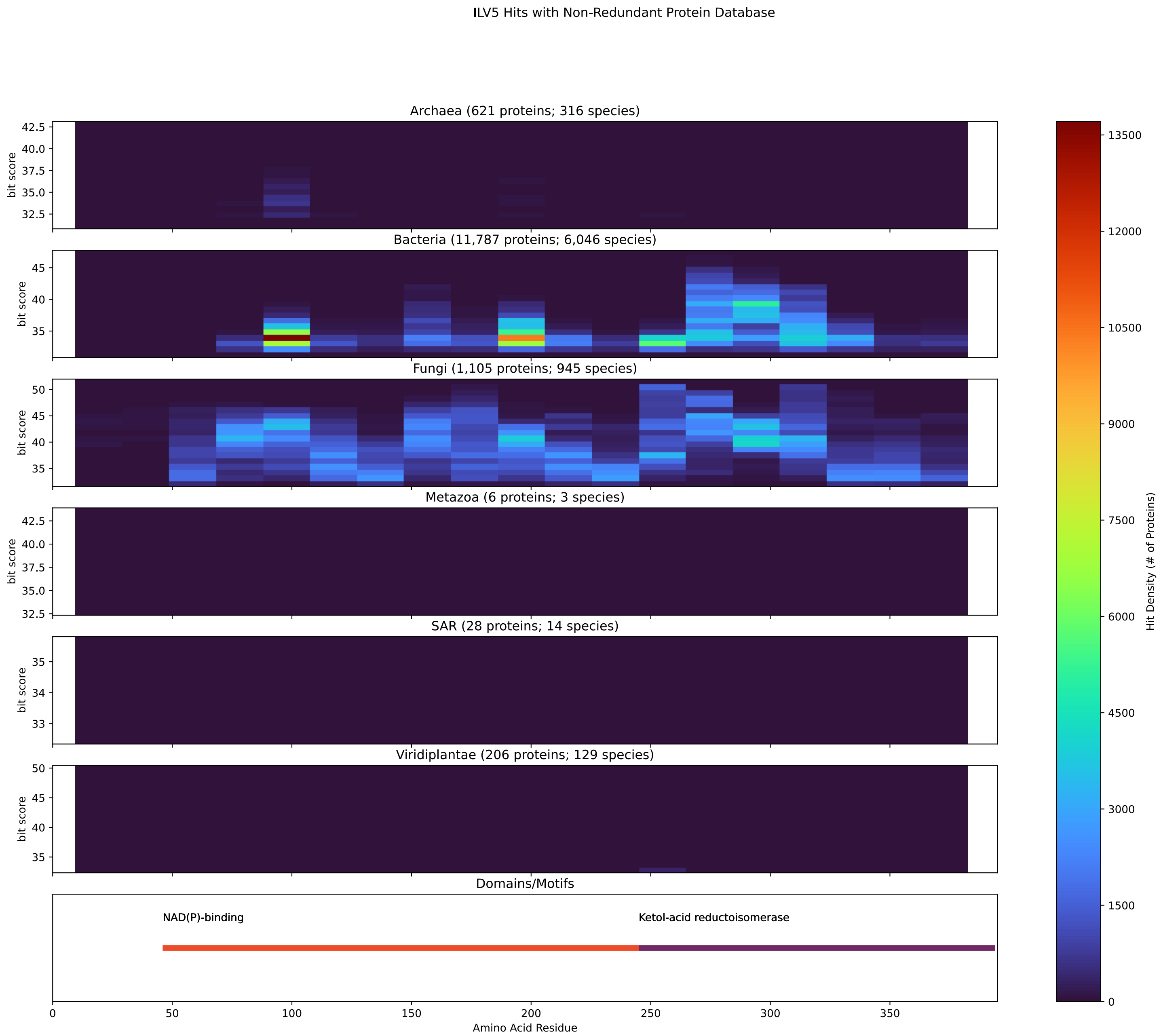

Figure S128: Non-redundant (NR) protein hits for DEG20010747/Ilv5 at 20 amino acid length queries.

S2.12 Rib3

S2.12.1 WHO Critical Pathogens

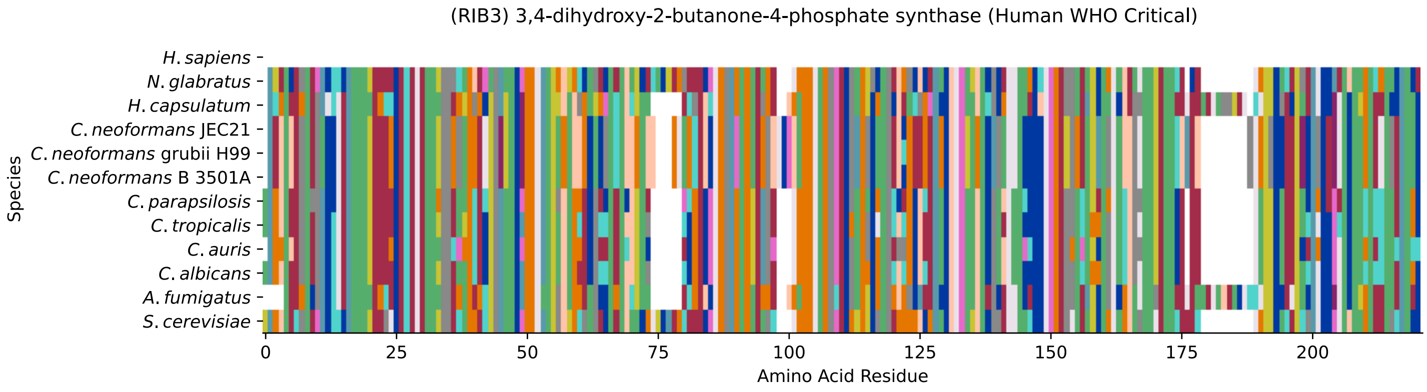

Figure S129: Multiple sequence alignment of yeast Rib3 (WHO Critical Pathogens). Cf. Figure S130 for alignment quality, and Figure S131 for Sneath similarity. Cf. Table S28 for protein names, and pairwise alignment metrics with yeast Rib3.

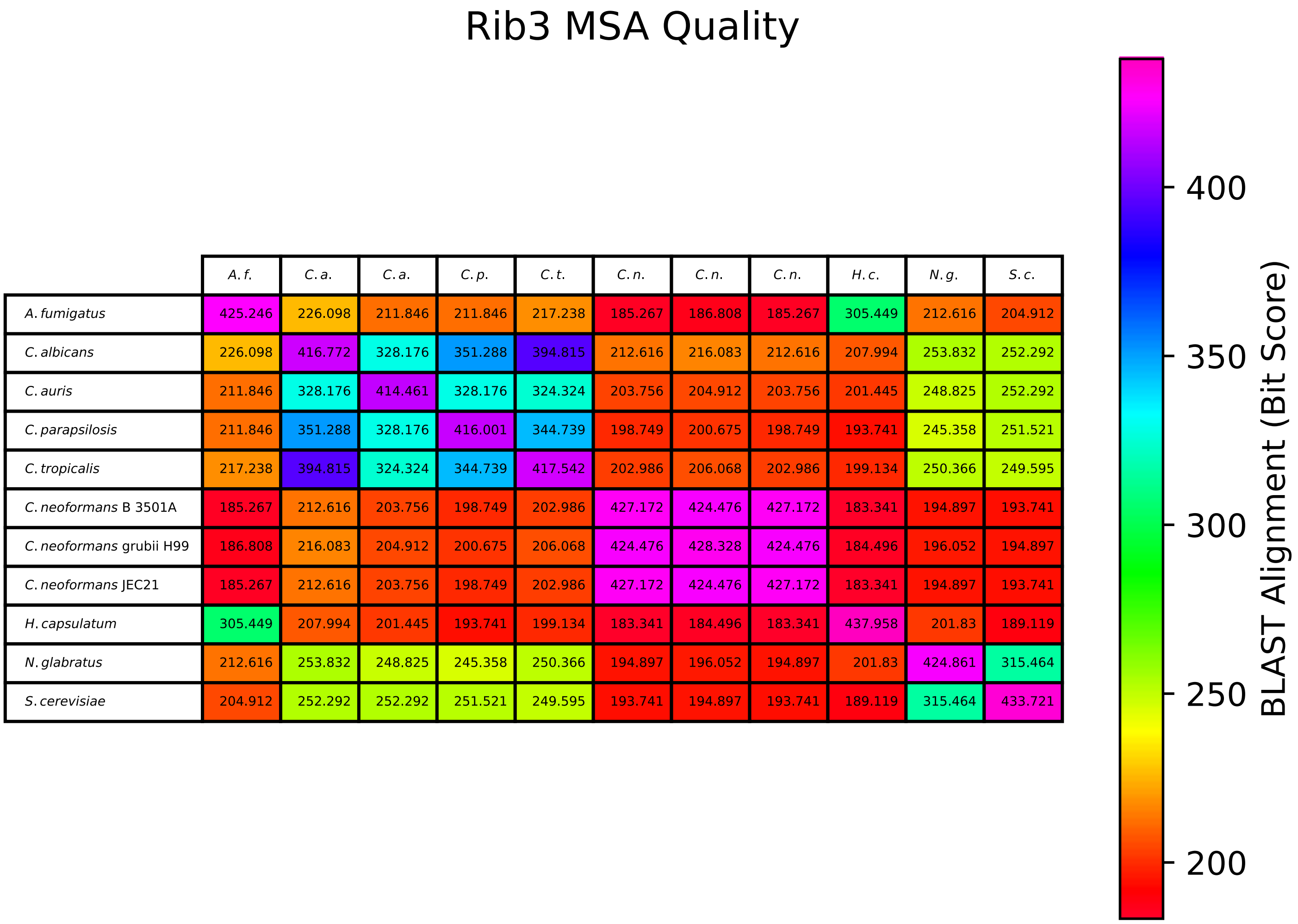

	A. f.	*C. a.*	*C. a.*	*C. p.*	*C. t.*	*C. n.*	*C. n.*	*C. n.*	*H. c.*	*N. g.*	*S. c.*
A. fumigatus	425.246	226.098	211.846	211.846	217.238	185.267	186.808	185.267	305.449	212.616	204.912
C. albicans	226.098	416.772	328.176	351.288	394.815	212.616	216.083	212.616	207.994	253.832	252.292
C. auris	211.846	328.176	414.461	328.176	324.324	203.756	204.912	203.756	201.445	248.825	252.292
C. parapsilosis	211.846	351.288	328.176	416.001	344.739	198.749	200.675	198.749	193.741	245.358	251.521
C. tropicalis	217.238	394.815	324.324	344.739	417.542	202.986	206.068	202.986	199.134	250.366	249.595
C. neoformans B 3501A	185.267	212.616	203.756	198.749	202.986	427.172	424.476	427.172	183.341	194.897	193.741
C. neoformans grubii H99	186.808	216.083	204.912	200.675	206.068	424.476	428.328	424.476	184.496	196.052	194.897
C. neoformans JEC21	185.267	212.616	203.756	198.749	202.986	427.172	424.476	427.172	183.341	194.897	193.741
H. capsulatum	305.449	207.994	201.445	193.741	199.134	183.341	184.496	183.341	437.958	201.83	189.119
N. glabratus	212.616	253.832	248.825	245.358	250.366	194.897	196.052	194.897	201.83	424.861	315.464
S. cerevisiae	204.912	252.292	252.292	251.521	249.595	193.741	194.897	193.741	189.119	315.464	433.721

Figure S130: Multiple sequence alignment quality of Rib3 (WHO Critical Pathogens). Cf. Figure S129

Species	Hit Protein	Hit Length (a.a.)	evalue	align_len	bit_score	identity	positive	score	gaps	% identity	% positive
H.sapiens	-	-	-	-	-	-	-	-	-	-	-
N.glabratus	XP_447577.1 uncharacterized protein CAGL0I07557g Nakaseomyces glabratus	207	2.2e-111	207	315.464	150	171	807	1	72.1	82.2
H.capsulatum	XP_045289685.1 3,4-dihydroxy–2-butanone 4-phosphate synthase Histoplasma capsulatum G186AR	217	4.5e-61	217	189.119	98	136	479	16	47.1	65.4
C.neoformans.JEC21	XP_570768.1 3,4 dihydroxy-2-butanone-4-phosphate synthase, putative Cryptococcus neoformans var. neoformans JEC21	211	3.8e-63	211	194.512	99	133	493	7	47.6	63.9
C.neoformans.grubii.H99	XP_012050191.1 3,4-dihydroxy–2-butanone-4-phosphate synthase Cryptococcus neoformans var. grubii H99	211	2.1e-63	211	195.282	100	134	495	7	48.1	64.4
C.neoformans.B.3501A	XP_775331.1 hypothetical protein CNBE0490 Cryptococcus neoformans var. neoformans B-3501A	211	3.6e-63	211	194.512	99	133	493	7	47.6	63.9
C.parapsilosis	XP_036668231.1 uncharacterized protein CPAR2 700750 Candida parapsilosis	209	2.8e-86	209	251.906	124	155	642	9	59.6	74.5
C.tropicalis	XP_002549222.1 3,4-dihydroxy–2-butanone 4-phosphate synthase Candida tropicalis MYA-3404	208	2.1e-85	208	249.595	122	153	636	7	58.7	73.6
C.auris	XP_028891318.1 3,4-dihydroxy–2-butanone 4-phosphate synthase Candida auris	208	1.2e-86	208	252.677	123	156	644	9	59.1	75.0
C.albicans	XP_716297.2 3,4-dihydroxy-2-butanone-4-phosphate synthase Candida albicans SC5314	208	1.7e-86	208	252.292	122	157	643	7	58.7	75.5
A.fumigatus	XP_751191.1 3,4-dihydroxy-2-butanone 4-phosphate synthase Aspergillus fumigatus Af293	215	3.3e-67	215	204.912	104	142	520	17	50.0	68.3

Table S28: Pairwise alignment info from yeast Rib3 (DEG20010261), cf. Figure S129.

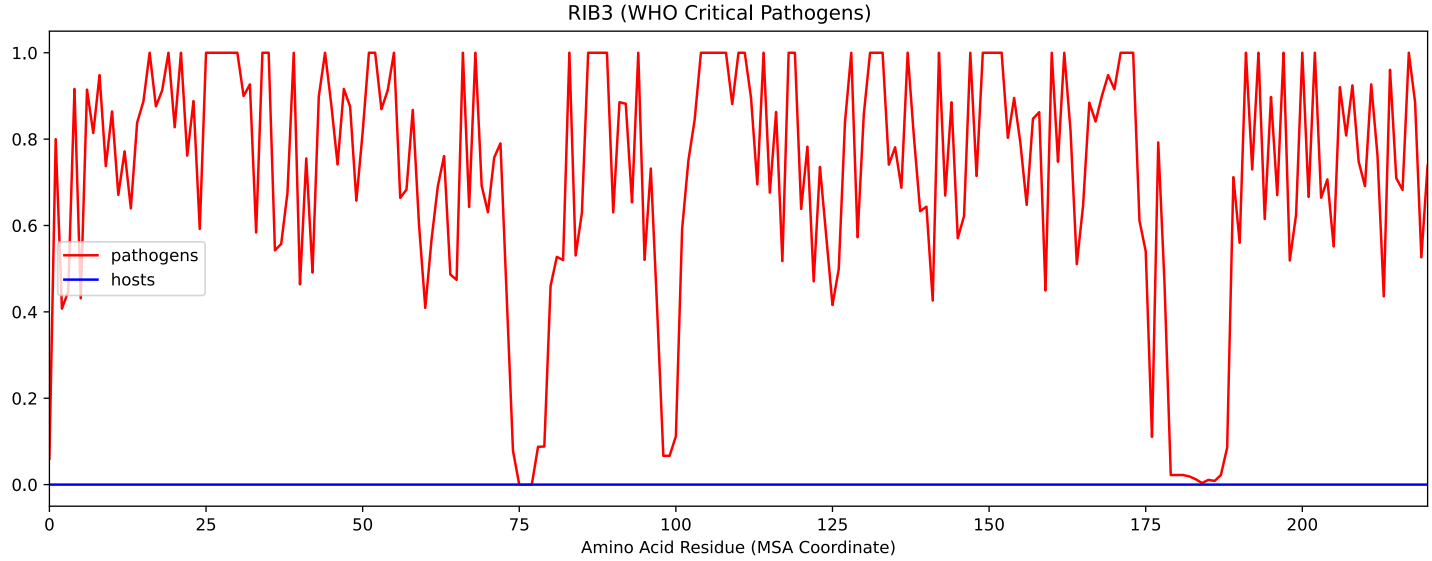

Figure S131: Sneath Similarity of Rib3 for WHO Critical Pathogens, cf. Figure S129

S2.12.2 Top 10 Agricultural Fungal Pathogens

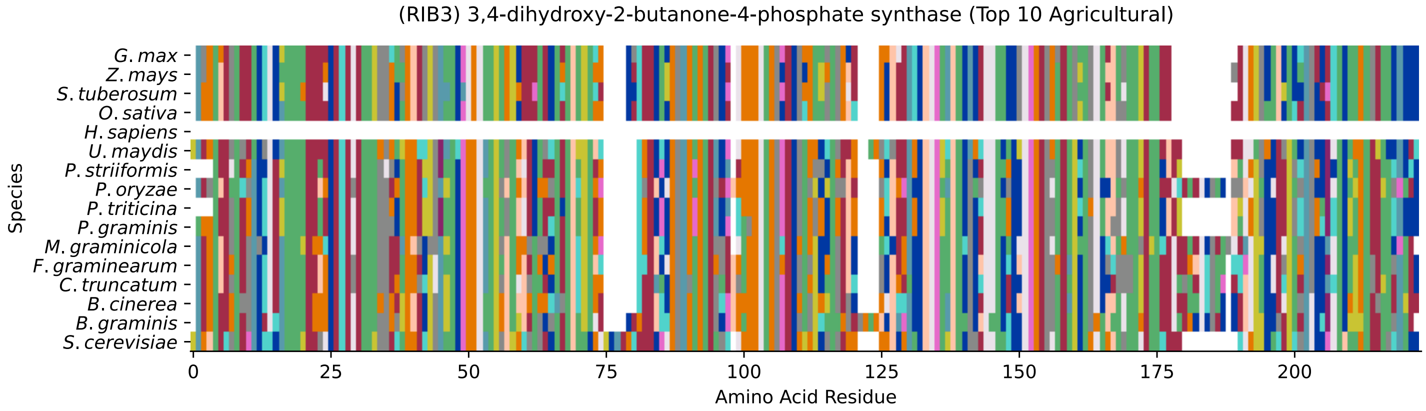

Figure S132: Multiple sequence alignment of yeast Rib3 (Top 10 Agricultural Fungal Pathogens). Cf. Figure S133 for alignment quality, and Figure S134 for Sneath similarity. Cf. Table S29 for protein names, and pairwise alignment metrics with yeast Rib3.

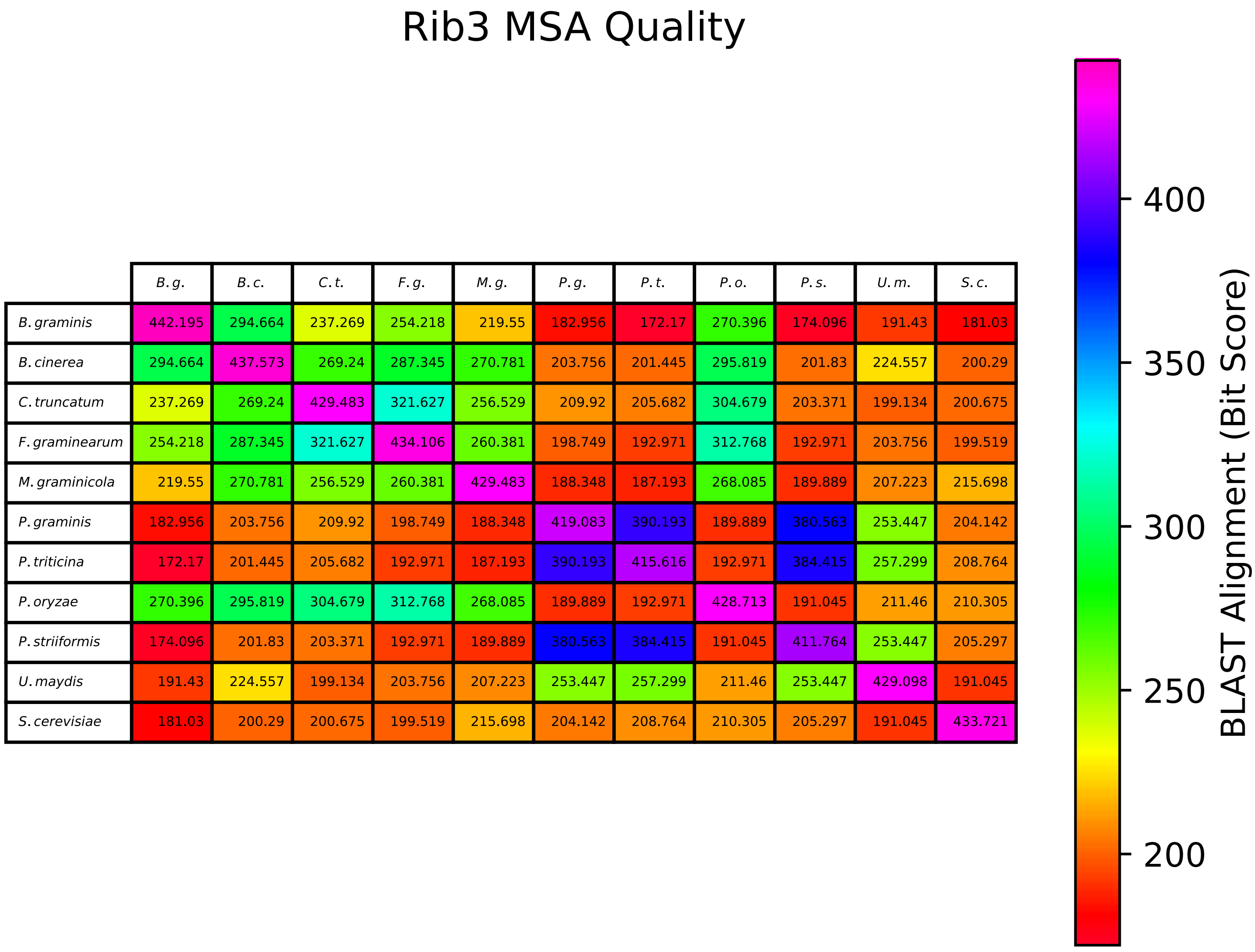

	B.g.	B.c.	C.t.	F.g.	M.g.	P.g.	P.t.	P.o.	P.s.	U.m.	S.c.
B. graminis	442.195	294.664	237.269	254.218	219.55	182.956	172.17	270.396	174.096	191.43	181.03
B. cinerea	294.664	437.573	269.24	287.345	270.781	203.756	201.445	295.819	201.83	224.557	200.29
C. truncatum	237.269	269.24	429.483	321.627	256.529	209.92	205.682	304.679	203.371	199.134	200.675
F. graminearum	254.218	287.345	321.627	434.106	260.381	198.749	192.971	312.768	192.971	203.756	199.519
M. graminicola	219.55	270.781	256.529	260.381	429.483	188.348	187.193	268.085	189.889	207.223	215.698
P. graminis	182.956	203.756	209.92	198.749	188.348	419.083	390.193	189.889	380.563	253.447	204.142
P. triticina	172.17	201.445	205.682	192.971	187.193	390.193	415.616	192.971	384.415	257.299	208.764
P. oryzae	270.396	295.819	304.679	312.768	268.085	189.889	192.971	428.713	191.045	211.46	210.305
P. striiformis	174.096	201.83	203.371	192.971	189.889	380.563	384.415	191.045	411.764	253.447	205.297
U. maydis	191.43	224.557	199.134	203.756	207.223	253.447	257.299	211.46	253.447	429.098	191.045
S. cerevisiae	181.03	200.29	200.675	199.519	215.698	204.142	208.764	210.305	205.297	191.045	433.721

Figure S133: Multiple sequence alignment quality of Rib3 (Top 10 Agricultural Fungal Pathogens). Cf. Figure S132

Species	Hit Protein	Hit Length (a.a.)	evalue	align_len	bit_score	identity	positive	score	gaps	% identity	% positive
G.max	XP_003545697.1 bifunctional r-iboflavin biosynthesis protein RIBA 1, chloroplastic Glycine max	207	3.4e-60	207	197.978	101	137	502	6	48.6	65.9
Z.mays	XP_008677643.1 uncharacterize-d protein LOC100383821 isoform X5 Zea mays	207	2.5e-59	207	193.356	101	135	490	5	48.6	64.9
S.tuberosum	XP_006351756.1 PREDICTED: bif-unctional riboflavin biosynthe-sis protein RIBA 1, chloroplas-tic-like Solanum tuberosum	207	4.5e-60	207	197.208	99	138	500	6	47.6	66.3
O.sativa	XP_015651119.1 probable bifun-ctional riboflavin biosynthesi-s protein RIBA 1, chloroplasti-c isoform X1 Oryza sativa Japo-nica Group	207	6.4e-60	207	196.823	100	141	499	5	48.1	67.8
H.sapiens	-	-	-	-	-	-	-	-	-	-	-
U.maydis	XP_011392588.1 putative 3,4-d-ihydroxy-2-butanone 4-phosphat-e synthase Ustilago maydis 521	211	9.6e-62	211	191.045	96	140	484	9	46.2	67.3
P.striiformis	XP_047796296.1 uncharacterize-d protein Pst134EA 032002 Pucc-inia striiformis f. sp. tritic-i	205	1.4e-67	205	206.068	101	140	523	7	48.6	67.3
P.oryzae	mRNA M BR32 EuGene 00029391-p1 — transcript=mRNA M BR32 EuGe-ne 00029391 — gene=M BR32 EuGe-ne 00029391 — organism=Pyricul-aria oryzae BR32 — gene produc-t=unspecified product — transc-ript product=unspecified produ-ct — location=BR32 scaffold000-02:3710575-3711355(+) — protei-n length=233 — sequence SO=sup-ercontig — SO=protein coding g-ene — is pseudo=false	217	2.3e-69	217	210.69	106	145	535	16	51.0	69.7
P.triticina	XP_053020497.1 uncharacterize-d protein PtA15 5A515 Puccinia triticina	205	3.7e-69	205	209.534	103	138	532	7	49.5	66.3
P.graminis	XP_003338670.1 3,4-dihydroxy–2-butanone 4-phosphate synthas-e Puccinia graminis f. sp. tri-tici CRL 75-36-700-3	208	3.6e-67	208	204.527	98	141	519	7	47.1	67.8
M.graminicola	ZTRI 3.823.mRNA-p1 — transcrip-t=ZTRI 3.823.mRNA — gene=ZTRI 3.823 — organism=Zymoseptoria tritici IPO323 — gene product=-similar to 3 — transcript prod-uct=similar to 3 — location=Zt-ri chr 3:2587765-2588507(-) — protein length=226 — sequence SO=chromosome — SO=protein cod-ing gene — is pseudo=false	216	1.4e-71	216	216.083	108	149	549	16	51.9	71.6
F.graminearum	XP_011320316.1 hypothetical p-rotein FGSG 08452 Fusarium gra-minearum PH-1	217	6.9e-65	217	199.134	100	138	505	16	48.1	66.3
C.truncatum	XP_036587073.1 3,4-dihydroxy–2-butanone 4-phosphate synthas-e Colletotrichum truncatum	217	2.2e-65	217	200.675	100	140	509	16	48.1	67.3
B.cinerea	XP_001552345.1 Bcrib3 Botryti-s cinerea B05.10	217	4.1e-65	217	200.29	100	142	508	16	48.1	68.3
B.graminis	VDB94000.1 — transcript=BGT962-24V316 LOCUS7590 t1 — gene=BGT-96224V316 LOCUS7590 — organism-=Blumeria graminis f. sp. trit-ici 96224 — gene product=unspe-cified product — transcript pr-oduct=unspecified product — lo-cation=LR026992:19353413-19354-355(-) — protein length=247 — sequence SO=chromosome — SO=pr-otein coding gene — is pseudo=-false	220	7e-58	220	181.03	100	138	458	18	48.1	66.3

Table S29: Pairwise alignment info from yeast Rib3 (DEG20010261), cf. Figure S132.

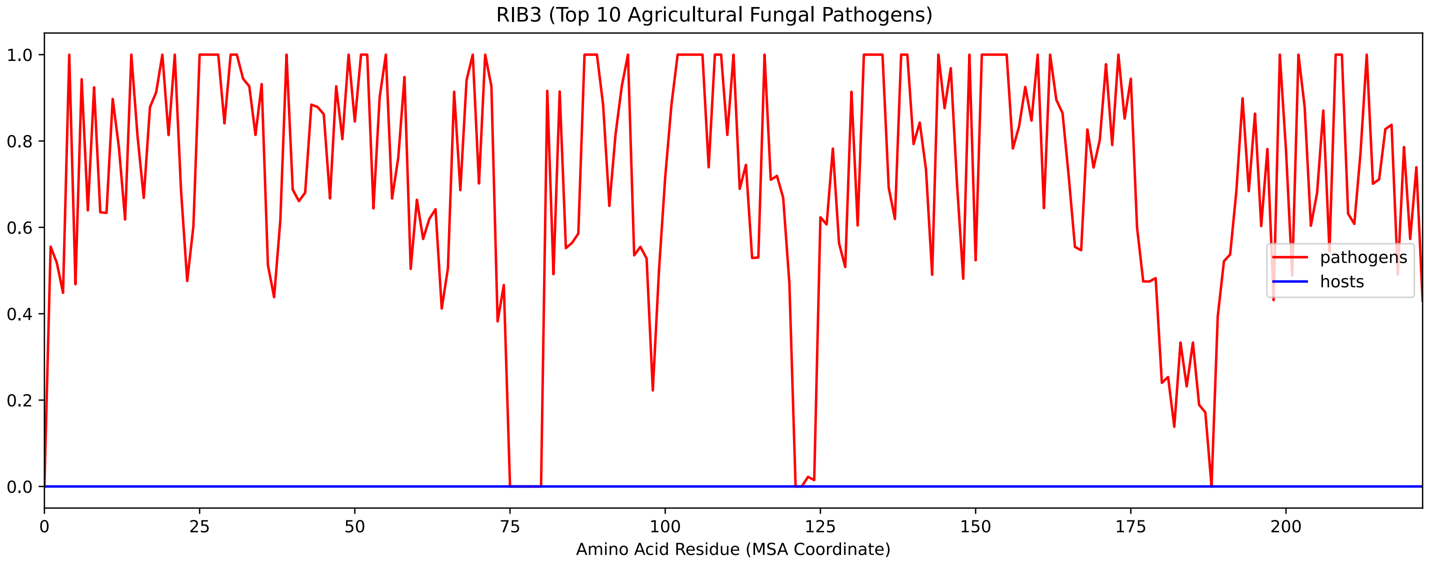

Figure S134: Sneath Similarity of Rib3 for Top 10 Agricultural Fungal Pathogens, cf. Figure S132

S2.12.3 NR

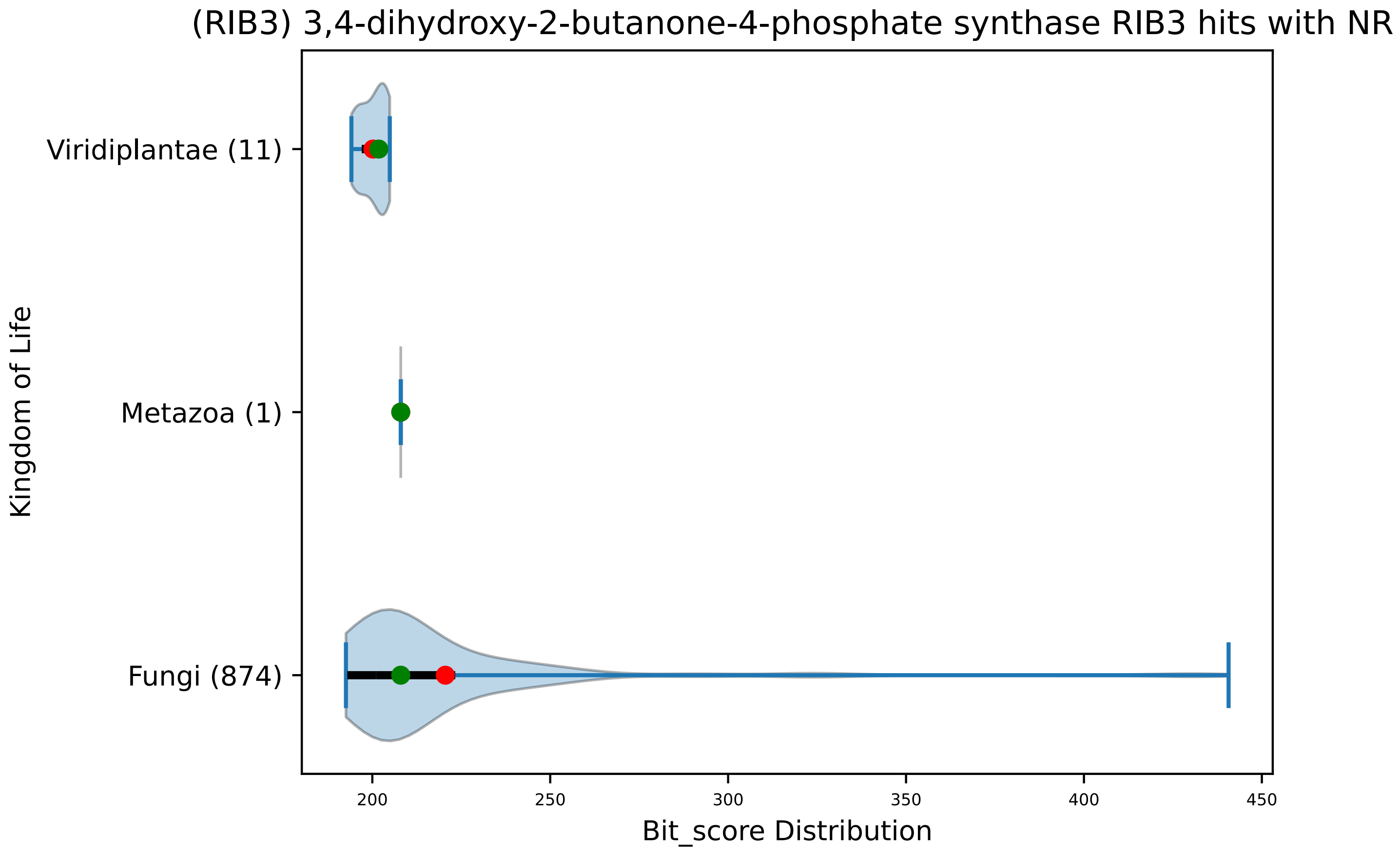

Figure S135: Non-redundant (NR) protein hits for DEG20010261/Rib3, with expectation value of no more than 0.1. Green points are medians, and red points are arithmetic means.

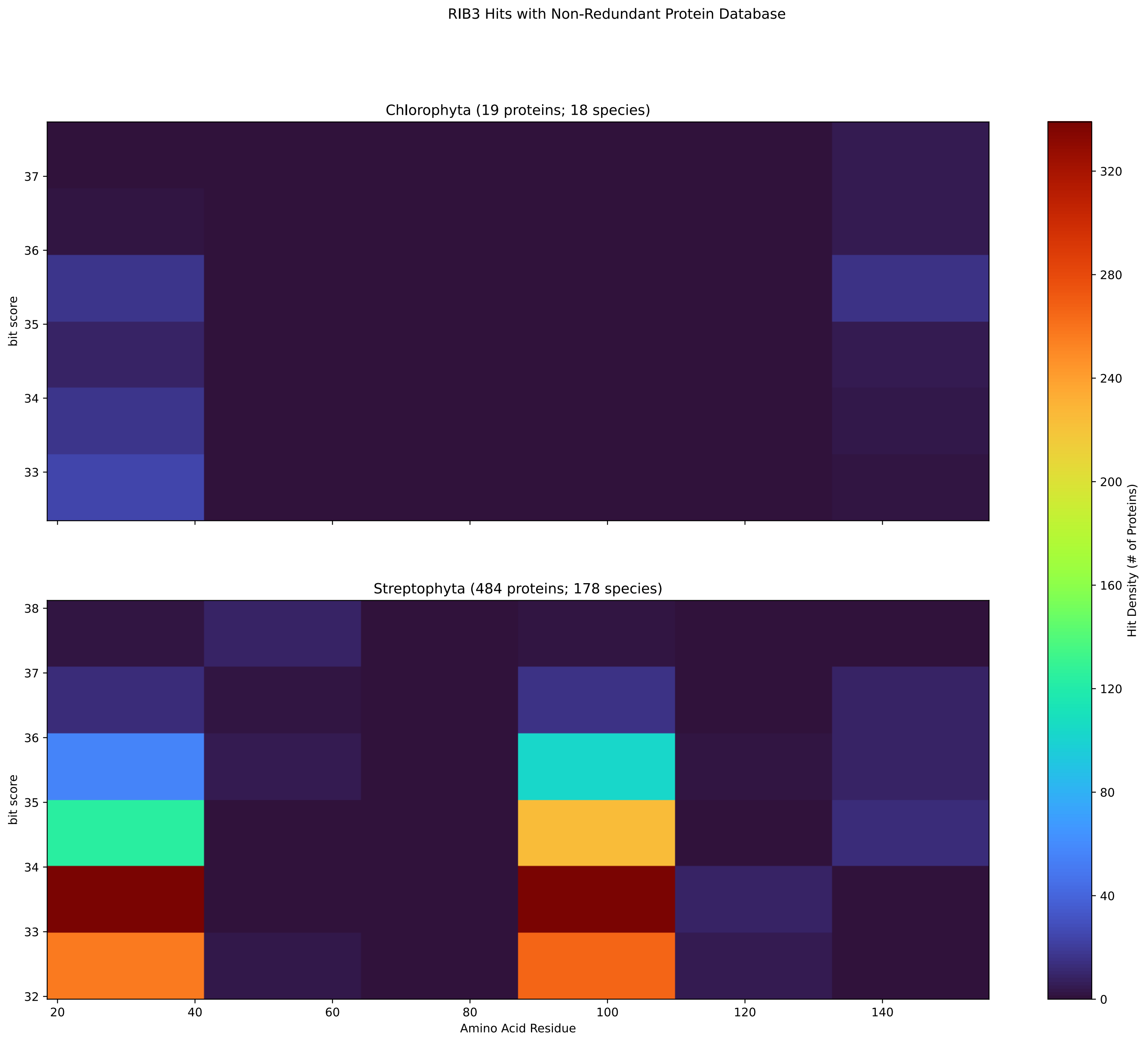

Figure S136: Non-redundant (NR) protein hits for Rib3 in the kingdom Viridiplantae.

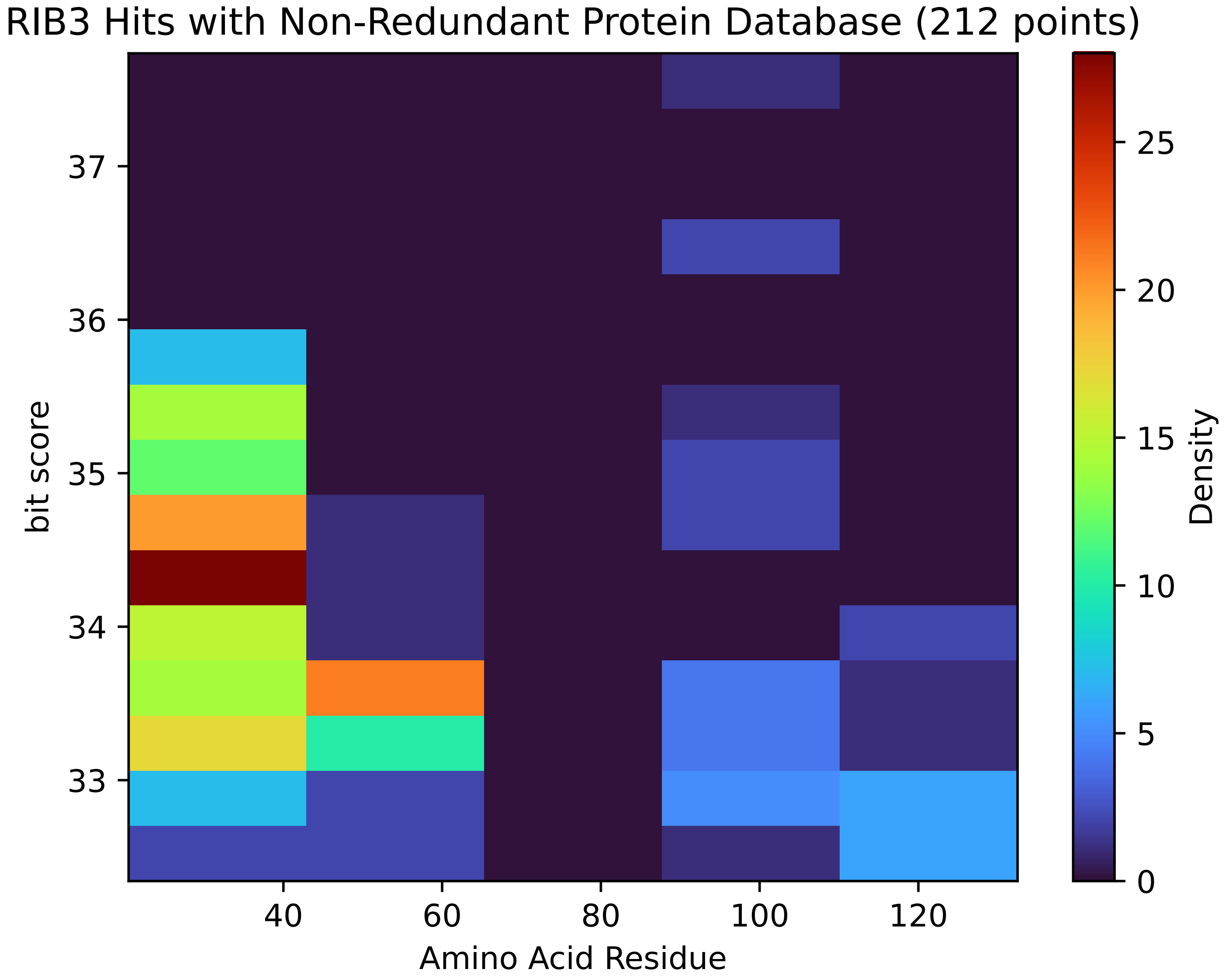

Figure S137: Non-redundant (NR) protein hits for Rib3 in the kingdom SAR.

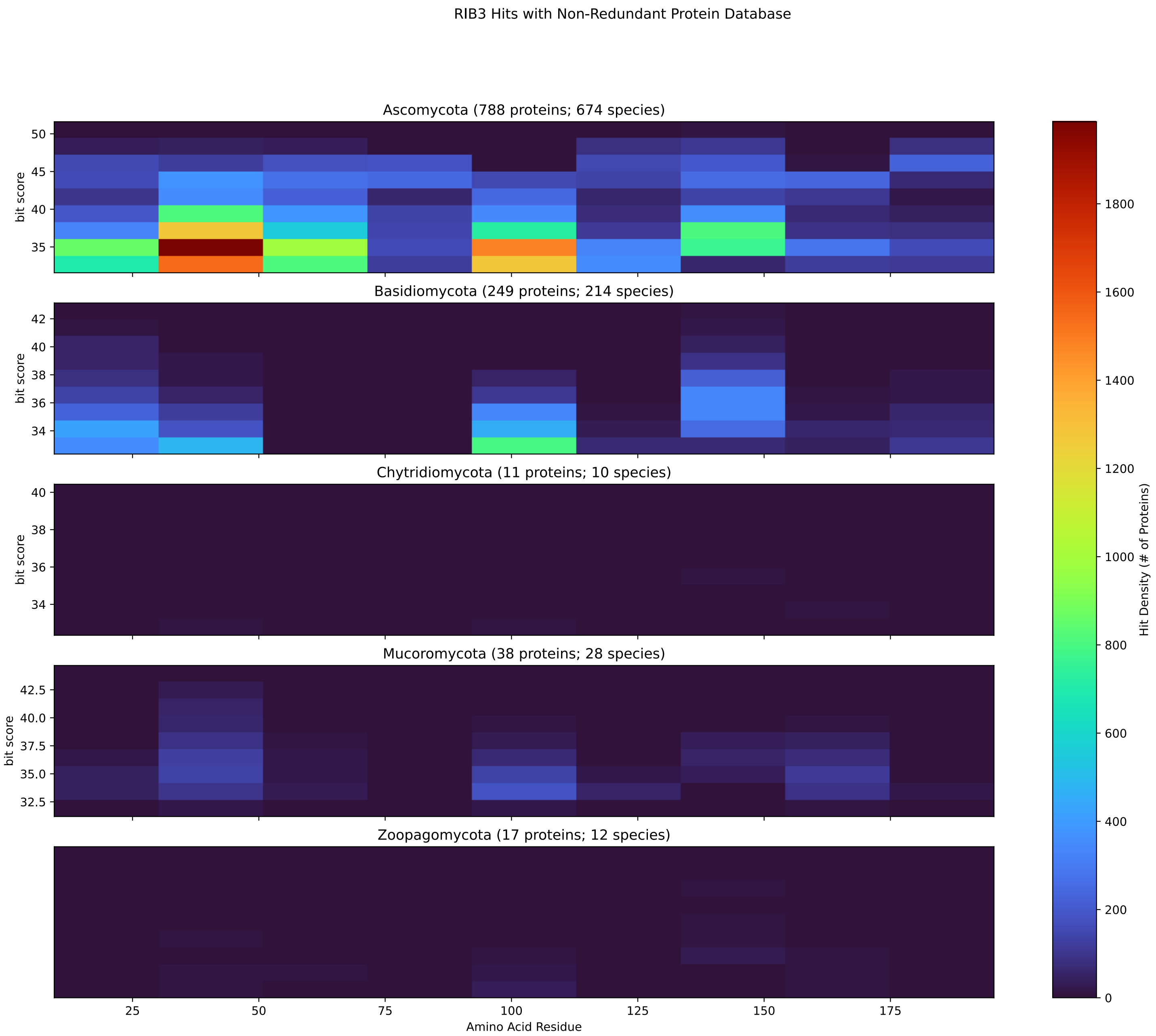

Figure S138: Non-redundant (NR) protein hits for Rib3 in the kingdom Fungi.

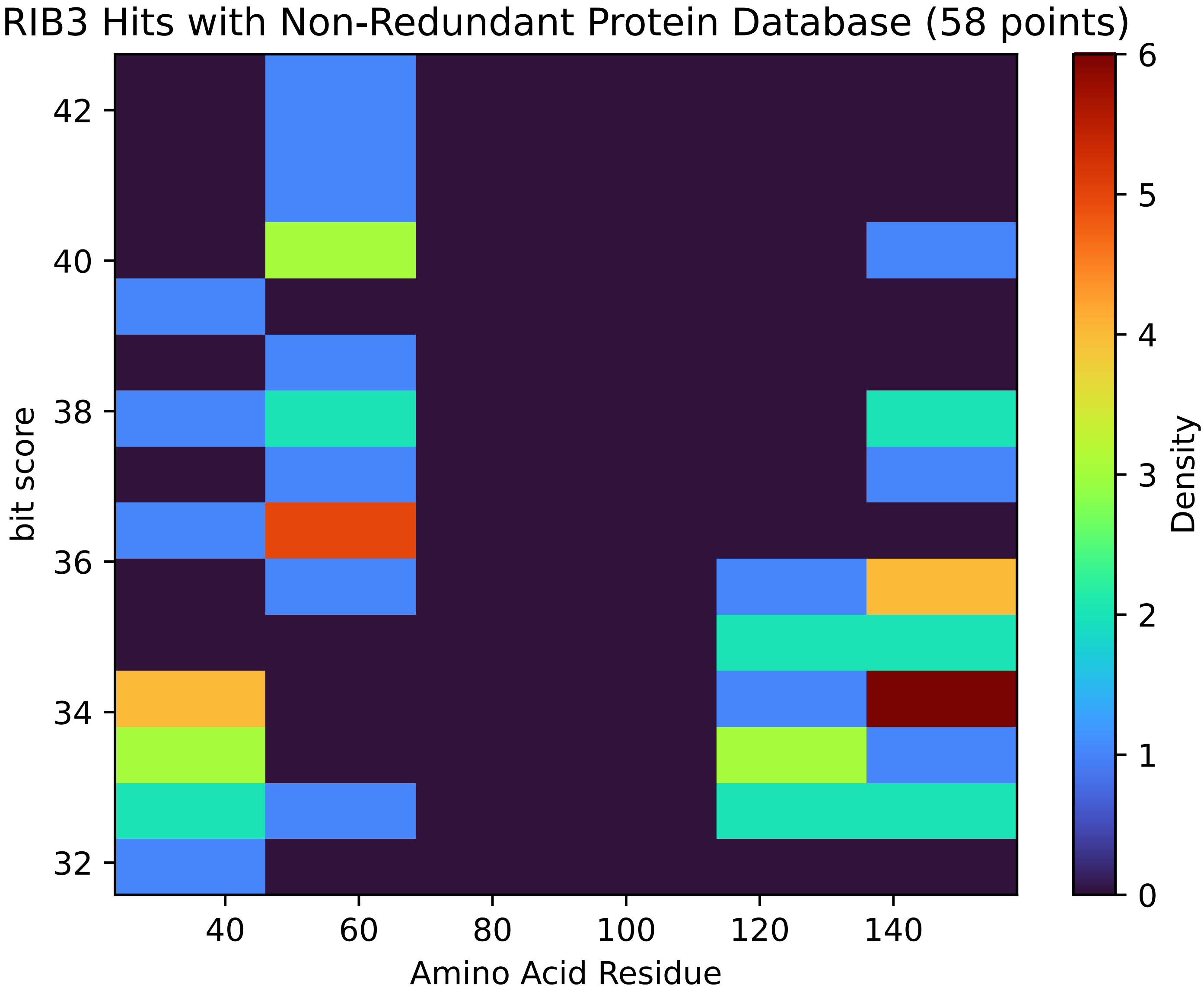

Figure S139: Non-redundant (NR) protein hits for Rib3 in the kingdom Metazoa.

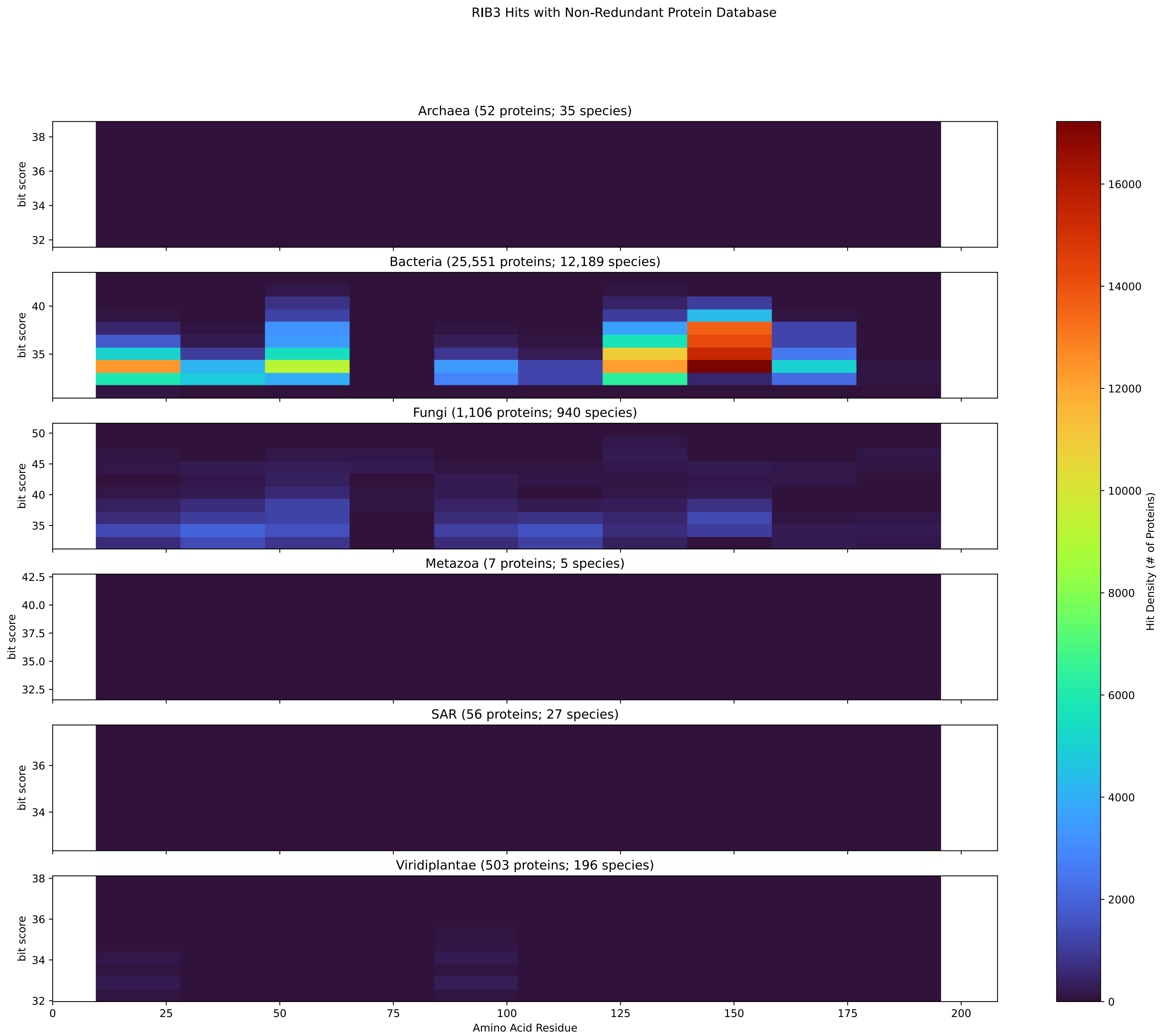

Figure S140: Non-redundant (NR) protein hits for DEG20010261/Rib3 at 20 amino acid length queries.

S2.13 Rib5

S2.13.1 WHO Critical Pathogens

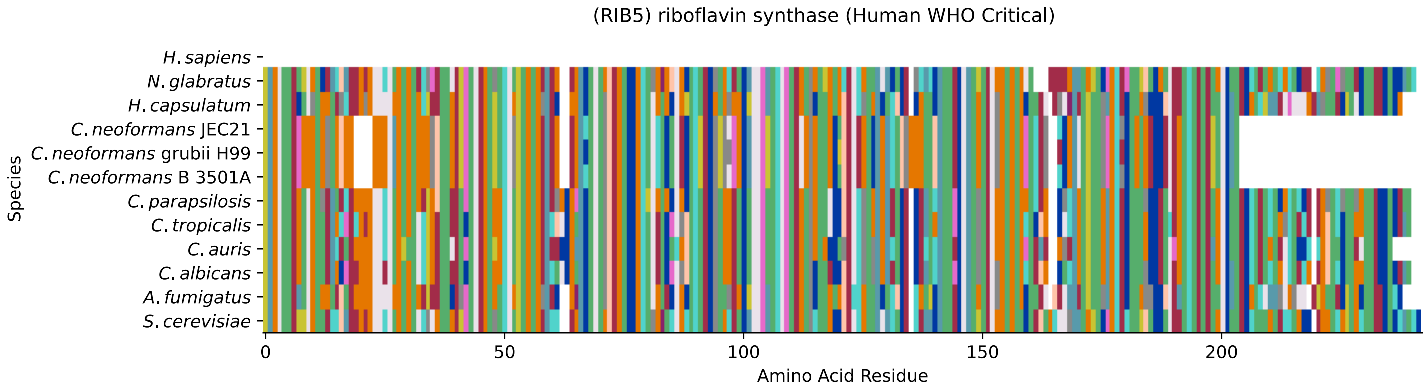

Figure S141: Multiple sequence alignment of yeast Rib5 (WHO Critical Pathogens). Cf. Figure S142 for alignment quality, and Figure S143 for Sneath similarity. Cf. Table S30 for protein names, and pairwise alignment metrics with yeast Rib5.

Rib5 MSA Quality

	A. f.	*C. a.*	*C. a.*	*C. p.*	*C. t.*	*C. n.*	*C. n.*	*C. n.*	*H. c.*	*N. g.*	*S. c.*
A. fumigatus	473.396	258.84	244.588	261.922	245.358	211.46	216.468	211.46	380.563	239.195	233.032
C. albicans	243.432	471.855	343.969	375.555	427.172	199.904	202.986	199.904	261.536	259.225	274.248
C. auris	228.409	343.969	464.922	338.576	340.502	202.986	204.912	202.986	249.98	261.536	255.758
C. parapsilosis	246.128	375.555	338.576	470.7	379.022	211.075	214.542	211.075	263.848	272.707	273.092
C. tropicalis	232.261	427.172	340.502	379.022	470.7	193.741	197.208	193.741	246.128	259.225	268.085
C. neoformans B 3501A	207.608	199.904	202.986	211.075	193.741	399.438	390.578	399.438	216.083	190.274	189.119
C. neoformans grubii H99	211.846	202.986	204.912	214.542	197.208	390.578	400.208	390.578	221.09	193.741	194.512
C. neoformans JEC21	207.608	199.904	202.986	211.075	193.741	399.438	390.578	399.438	216.083	190.274	189.119
H. capsulatum	363.614	261.536	249.98	263.848	246.128	216.083	221.09	216.083	474.937	236.498	238.039
N. glabratus	226.098	259.225	261.536	272.707	259.225	190.274	193.741	190.274	236.498	477.633	355.525
S. cerevisiae	219.164	274.248	255.758	273.092	268.085	189.119	194.512	189.119	238.039	355.525	484.182

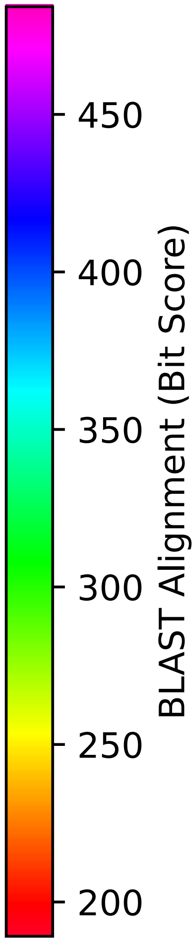

Figure S142: Multiple sequence alignment quality of Rib5 (WHO Critical Pathogens). Cf. Figure S141

Species	Hit Protein	Hit Length (a.a.)	evalue	align_len	bit_score	identity	positive	score	gaps	% identity	% positive
H.sapiens	-	-	-	-	-	-	-	-	-	-	-
N.glabratus	XP_445640.1 uncharacterized protein CAGL0D05302g Nakaseomyces glabratus	237	1.4e-126	237	356.295	172	205	913	3	72.3	86.1
H.capsulatum	XP_045285331.1 riboflavin synthase Histoplasma capsulatum G-186AR	235	8.5e-80	235	238.039	114	160	606	2	47.9	67.2
C.neoformans.JEC21	XP_569876.1 riboflavin synthase, putative Cryptococcus neoformans var. neoformans JEC21	202	9.8e-61	202	188.734	96	135	478	7	40.3	56.7
C.neoformans.grubii.H99	XP_012048272.1 riboflavin synthase, alpha subunit Cryptococcus neoformans var. grubii H99	202	8.4e-63	202	194.126	98	136	492	7	41.2	57.1
C.neoformans.B.3501A	XP_776613.1 hypothetical protein CNBC1060 Cryptococcus neoformans var. neoformans B-3501A	202	9.4e-61	202	188.734	96	135	478	7	40.3	56.7
C.parapsilosis	XP_036663168.1 uncharacterized protein CPAR2 100220 Candida parapsilosis	238	1.1e-93	238	273.092	132	181	697	4	55.5	76.1
C.tropicalis	XP_002546658.1 riboflavin synthase alpha chain Candida tropicalis MYA-3404	238	7.9e-92	238	268.47	130	178	685	4	54.6	74.8
C.auris	XP_028891770.1 riboflavin synthase, alpha subunit Candida auris	234	3.4e-87	234	256.144	130	168	653	4	54.6	70.6
C.albicans	XP_721932.2 riboflavin synthase Candida albicans SC5314	238	3.4e-94	238	274.248	134	176	700	4	56.3	73.9
A.fumigatus	XP_750373.1 riboflavin synthase, alpha subunit Aspergillus fumigatus Af293	234	2.4e-72	234	219.164	115	157	557	1	48.3	66.0

Table S30: Pairwise alignment info from yeast Rib5 (DEG20010082), cf. Figure S141.

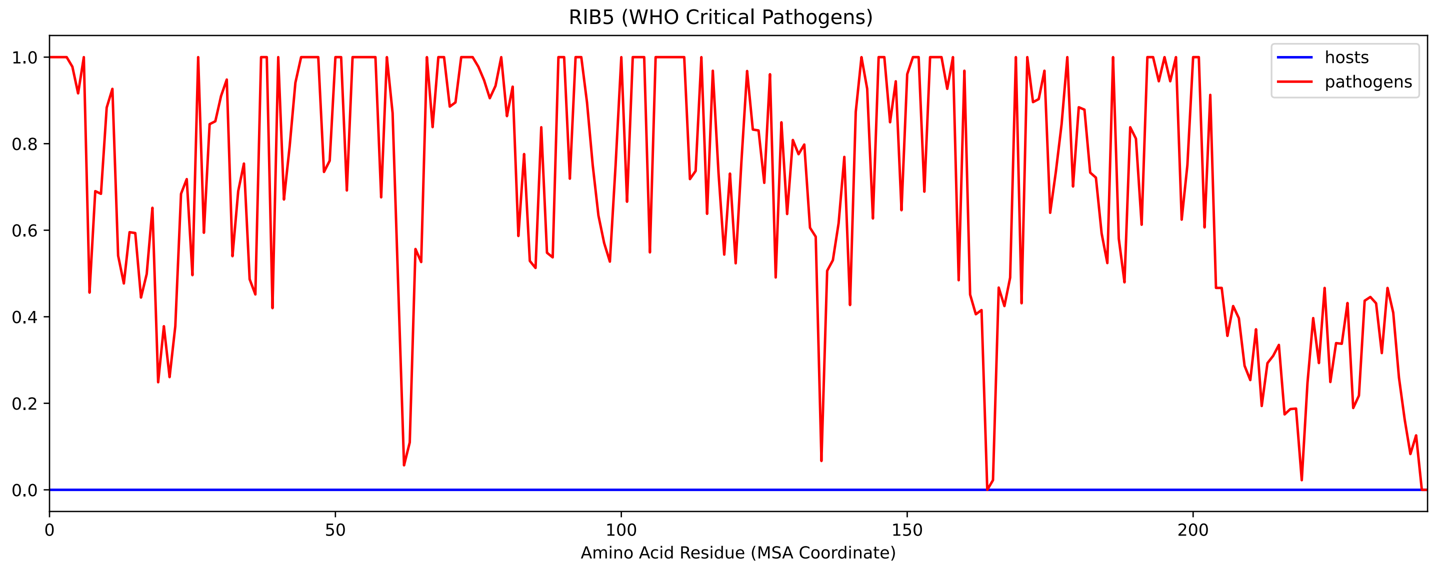

Figure S143: Sneath Similarity of Rib5 for WHO Critical Pathogens, cf. Figure S141

S2.13.2 Top 10 Agricultural Fungal Pathogens

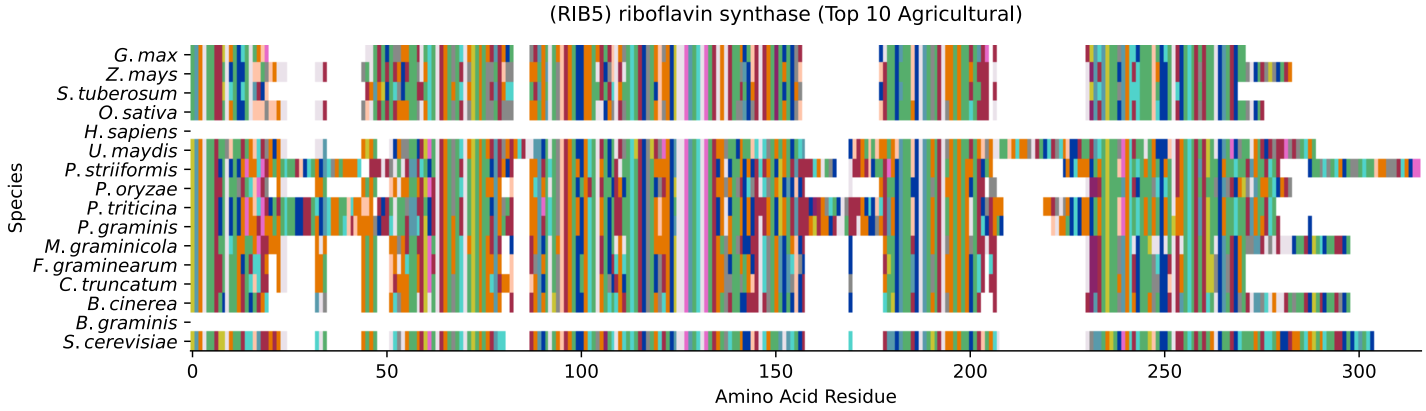

Figure S144: Multiple sequence alignment of yeast Rib5 (Top 10 Agricultural Fungal Pathogens). Cf. Figure S145 for alignment quality, and Figure S146 for Sneath similarity. Cf. Table S31 for protein names, and pairwise alignment metrics with yeast Rib5.

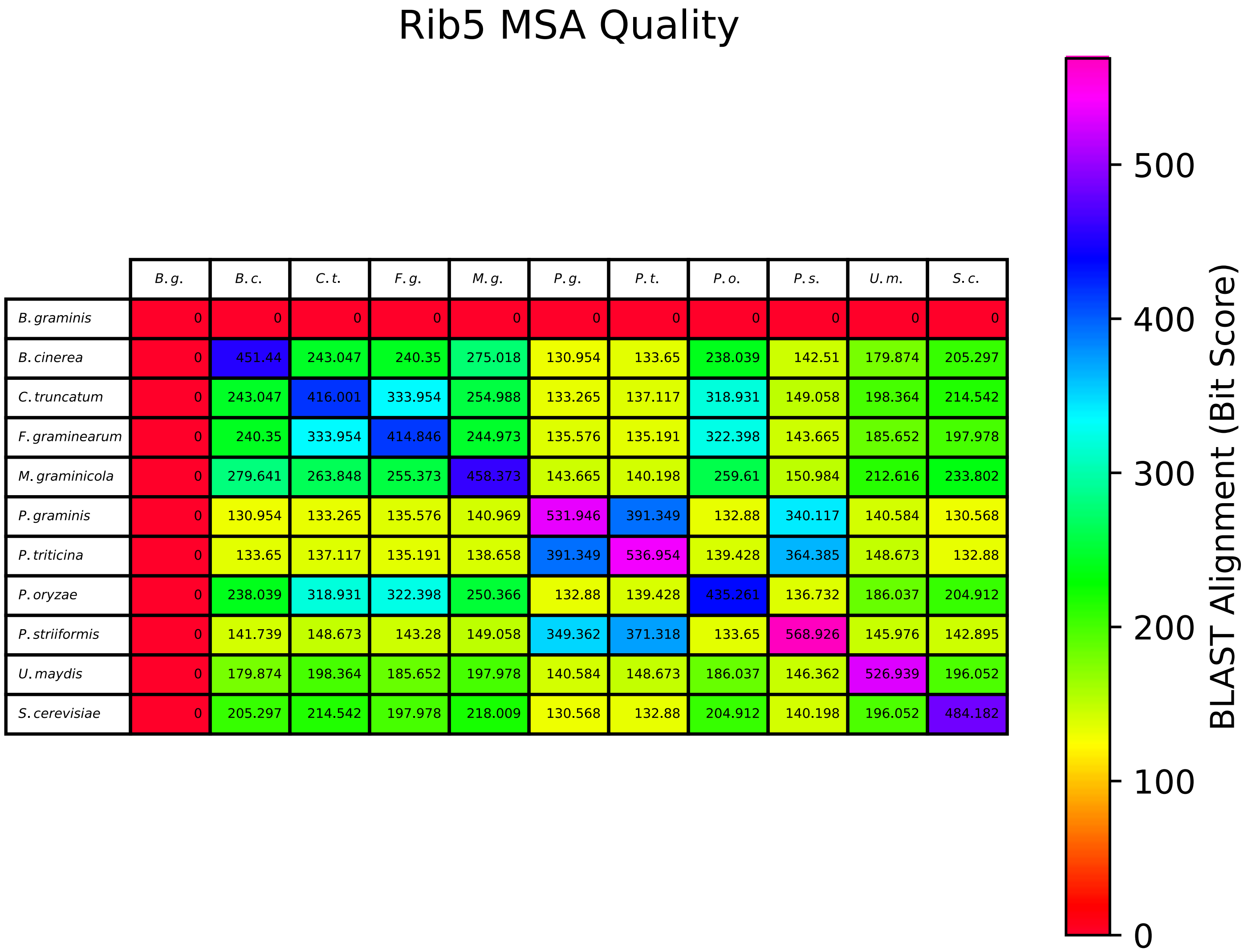

	B. g.	B. c.	C. t.	F. g.	M. g.	P. g.	P. t.	P. o.	P. s.	U. m.	S. c.
B. graminis	0	0	0	0	0	0	0	0	0	0	0
B. cinerea	0	451.44	243.047	240.35	275.018	130.954	133.65	238.039	142.51	179.874	205.297
C. truncatum	0	243.047	416.001	333.954	254.988	133.265	137.117	318.931	149.058	198.364	214.542
F. graminearum	0	240.35	333.954	414.846	244.973	135.576	135.191	322.398	143.665	185.652	197.978
M. graminicola	0	279.641	263.848	255.373	458.373	143.665	140.198	259.61	150.984	212.616	233.802
P. graminis	0	130.954	133.265	135.576	140.969	531.946	391.349	132.88	340.117	140.584	130.568
P. triticina	0	133.65	137.117	135.191	138.658	391.349	536.954	139.428	364.385	148.673	132.88
P. oryzae	0	238.039	318.931	322.398	250.366	132.88	139.428	435.261	136.732	186.037	204.912
P. striiformis	0	141.739	148.673	143.28	149.058	349.362	371.318	133.65	568.926	145.976	142.895
U. maydis	0	179.874	198.364	185.652	197.978	140.584	148.673	186.037	146.362	526.939	196.052
S. cerevisiae	0	205.297	214.542	197.978	218.009	130.568	132.88	204.912	140.198	196.052	484.182

Figure S145: Multiple sequence alignment quality of Rib5 (Top 10 Agricultural Fungal Pathogens). Cf. Figure S144

Species	Hit Protein	Hit Length (a.a.)	evalue	align_len	bit_score	identity	positive	score	gaps	% identity	% positive
G.max	XP_003539920.1 riboflavin synthase Glycine max	207	1.5e-53	207	174.866	96	135	442	11	40.3	56.7
Z.mays	NP_001149354.1 uncharacterized protein LOC100282978 Zea mays	226	3.4e-49	226	164.081	87	137	414	15	36.6	57.6
S.tuberosum	XP_006357493.1 PREDICTED: riboflavin synthase Solanum tuberosum	205	2.4e-52	205	171.014	92	134	432	11	38.7	56.3
O.sativa	XP_015618419.1 riboflavin synthase isoform X1 Oryza sativa Japonica Group	216	4.8e-47	216	158.303	84	129	399	10	35.3	54.2
H.sapiens	-	-	-	-	-	-	-	-	-	-	-
U.maydis	XP_011388024.1 riboflavin synthase Ustilago maydis 521	259	1e-62	259	196.438	104	152	498	36	43.7	63.9
P.striiformis	XP_047801472.1 hypothetical protein Pst134EA 022744 Puccinia striiformis f. sp. tritici	282	1.4e-40	282	139.813	97	145	351	47	40.8	60.9
P.oryzae	mRNA M BR32 EuGene 00002111-p1 — transcript=mRNA M BR32 EuGene 00002111 — gene=M BR32 EuGene 00002111 — organism=Pyricularia oryzae BR32 — gene product=unspecified product — transcript product=unspecified product — location=BR32 scaffold000-01:606531-607319(-) — protein length=214 — sequence SO=supercontig — SO=protein coding gene — is pseudo=false	219	5.3e-67	219	204.912	101	149	520	6	42.4	62.6
P.triticina	XP_053025945.1 uncharacterized protein PtA15 12A379 Puccinia triticina	264	6.4e-38	264	132.88	91	133	333	51	38.2	55.9
P.graminis	XP_003330596.1 riboflavin synthase, alpha subunit Puccinia graminis f. sp. tritici CRL 75-36-700-3	272	6.6e-37	272	130.568	92	128	327	64	38.7	53.8
M.graminicola	ZTRI 2.263.mRNA-p1 — transcript=ZTRI 2.263.mRNA — gene=ZTRI 2.263 — organism=Zymoseptoria tritici IPO323 — gene product=-similar to riboflavin synthase — transcript product=similar to riboflavin synthase — location=Ztri chr 2:855688-856441(–) — protein length=234 — sequence SO=chromosome — SO=protein coding gene — is pseudo=false	232	5.2e-72	232	218.394	110	157	555	1	46.2	66.0
F.graminearum	XP_011316898.1 riboflavin synthase alpha chain Fusarium graminearum PH-1	207	2.2e-63	207	196.823	100	142	499	8	42.0	59.7
C.truncatum	XP_036583443.1 riboflavin synthase Colletotrichum truncatum	207	4e-70	207	214.157	103	147	544	6	43.3	61.8
B.cinerea	XP_024551623.1 Bcrib5 Botrytis cinerea B05.10	232	1.1e-66	232	204.912	108	149	520	9	45.4	62.6
B.graminis	-	-	-	-	-	-	-	-	-	-	-

Table S31: Pairwise alignment info from yeast Rib5 (DEG20010082), cf. Figure S144.

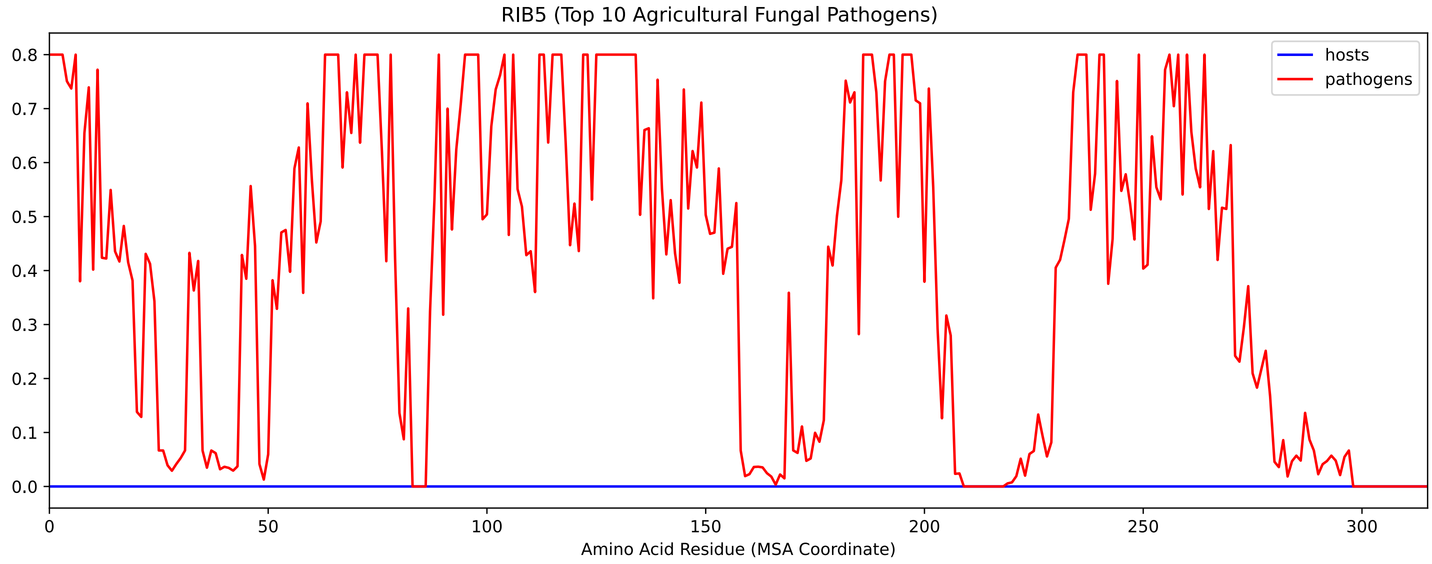

Figure S146: Sneath Similarity of Rib5 for Top 10 Agricultural Fungal Pathogens, cf. Figure S144

S2.13.3 NR

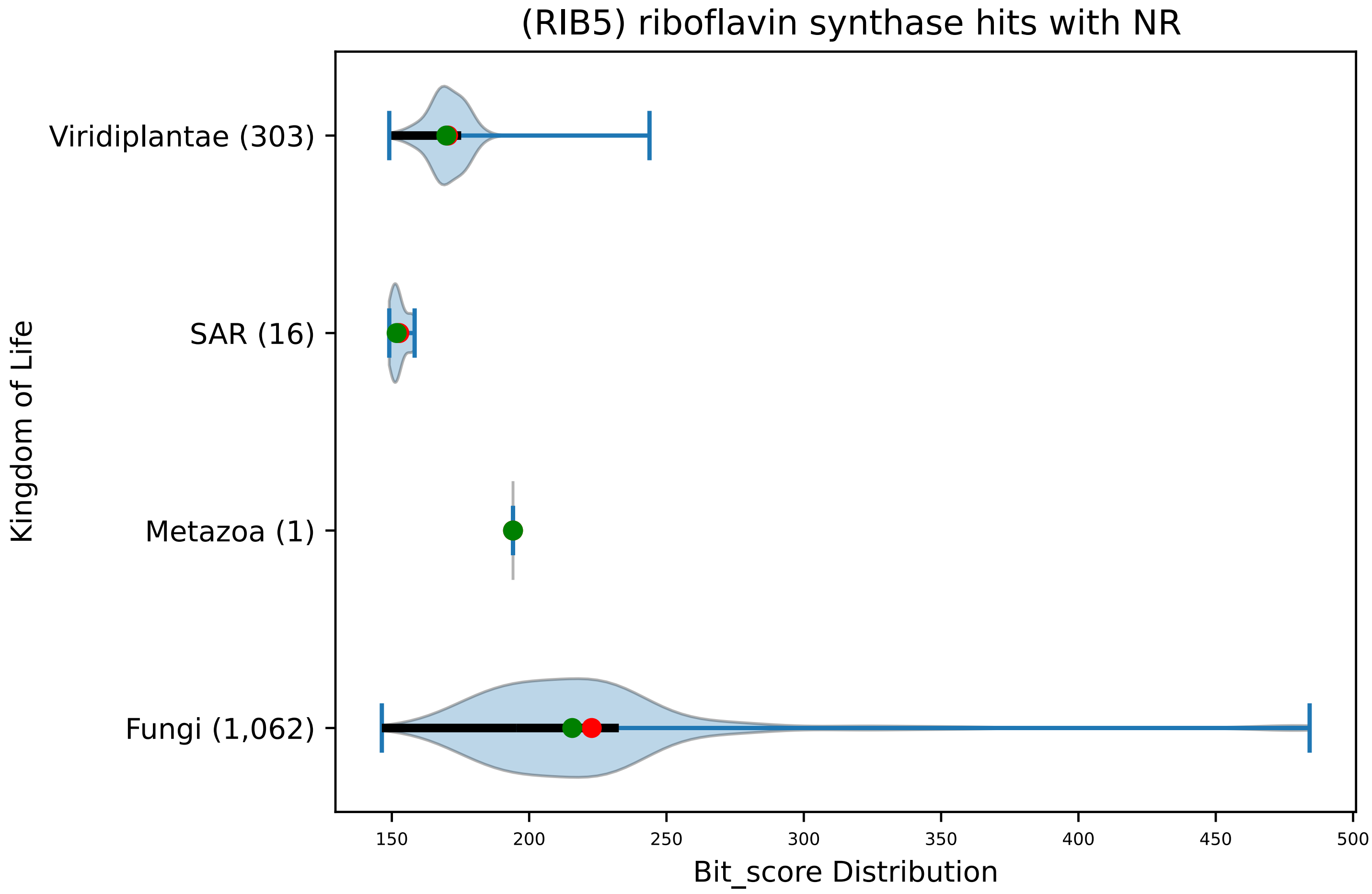

Figure S147: Non-redundant (NR) protein hits for DEG20010082/Rib5, with expectation value of no more than 0.1. Green points are medians, and red points are arithmetic means.

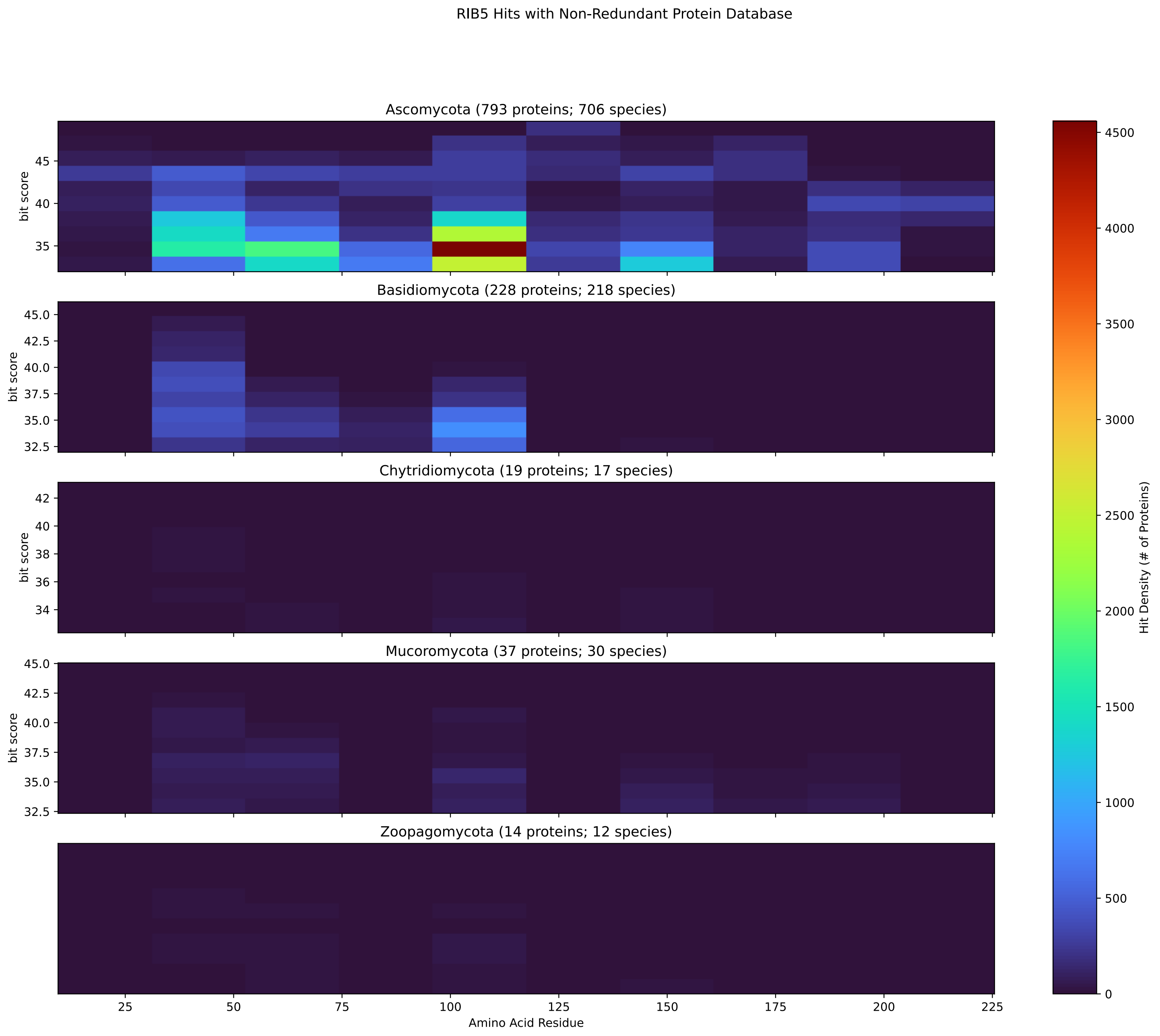

Figure S148: Non-redundant (NR) protein hits for Rib5 in the kingdom Fungi.

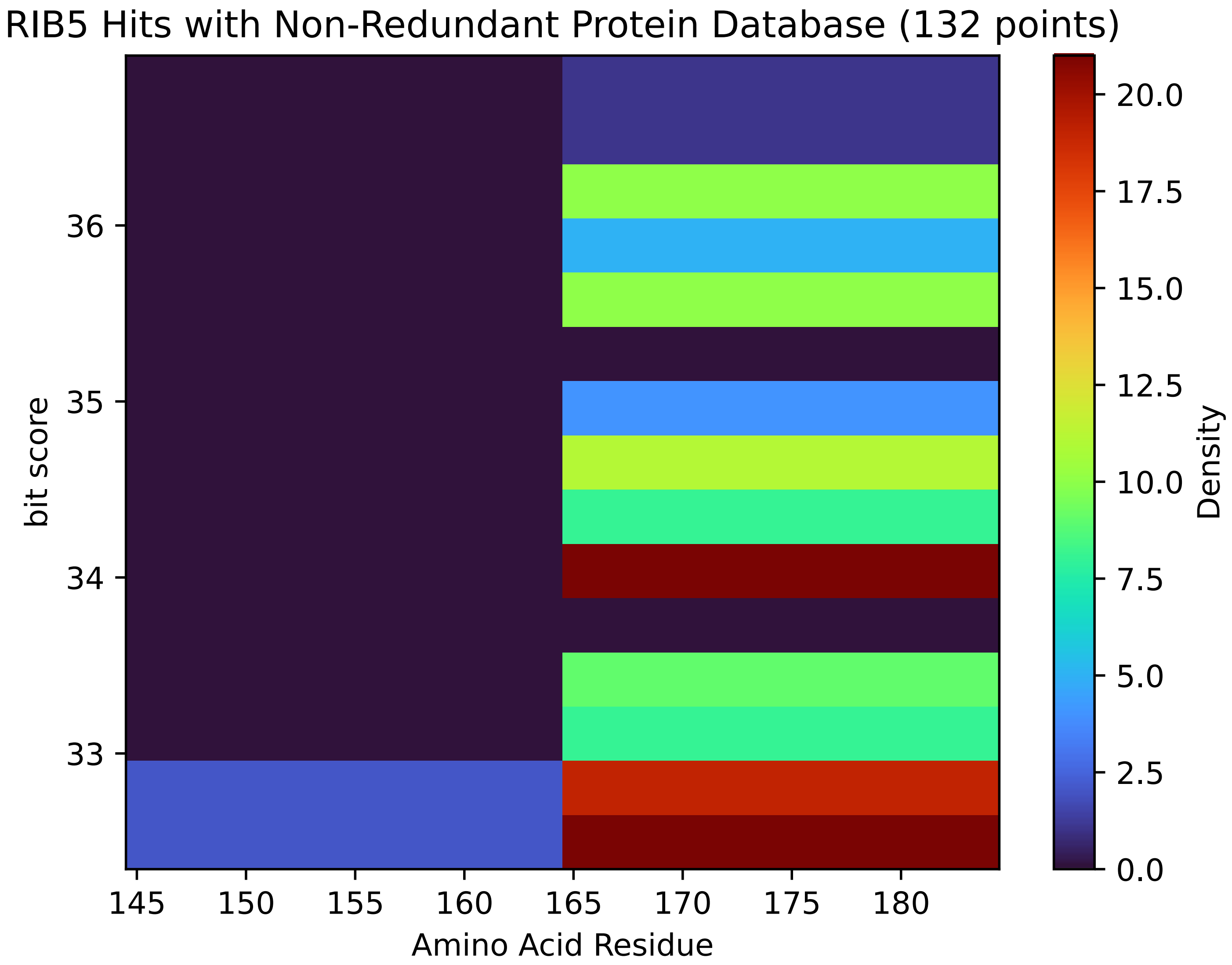

Figure S149: Non-redundant (NR) protein hits for Rib5 in the kingdom SAR.

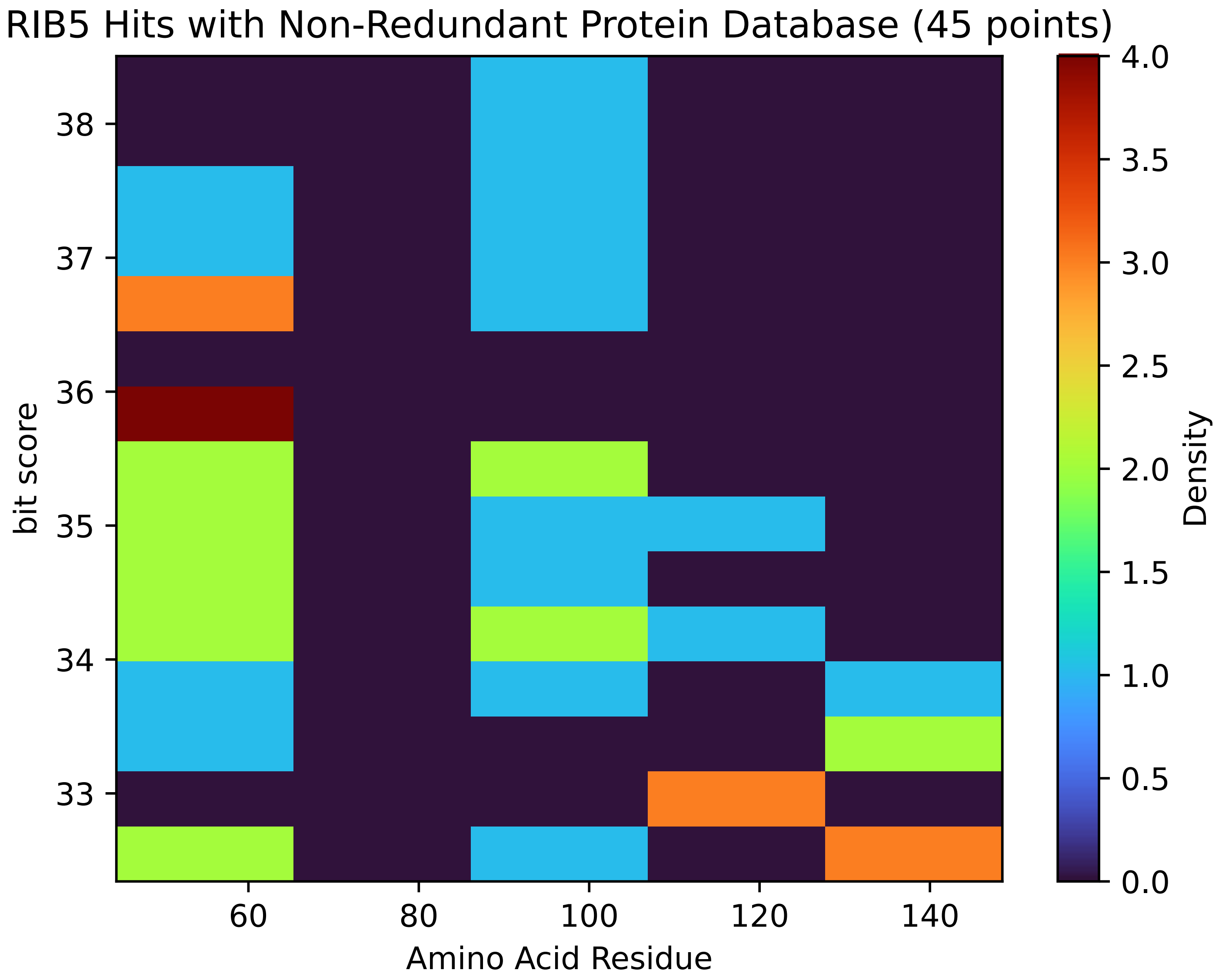

Figure S150: Non-redundant (NR) protein hits for Rib5 in the kingdom Metazoa.

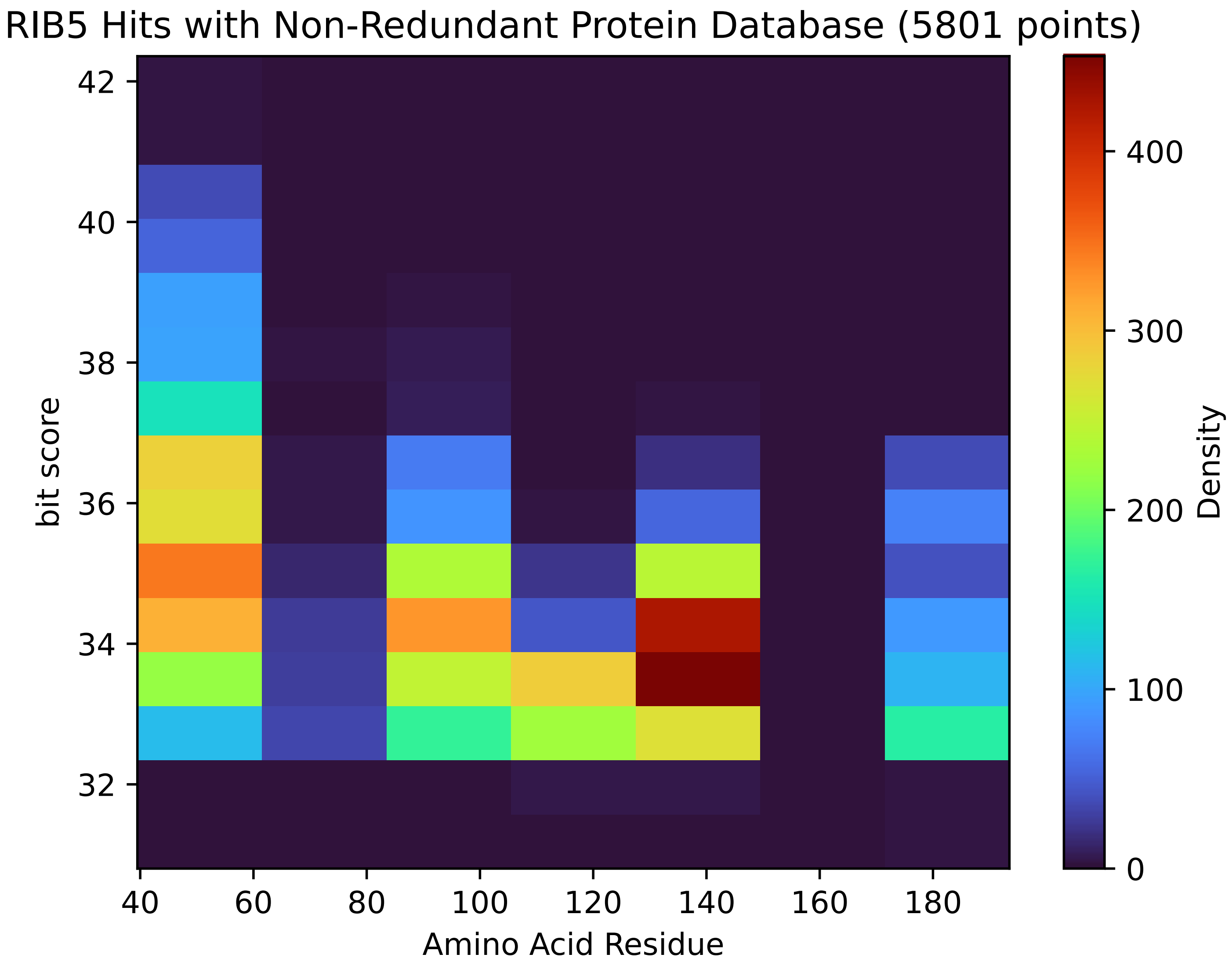

Figure S151: Non-redundant (NR) protein hits for Rib5 in the kingdom Viridiplantae.

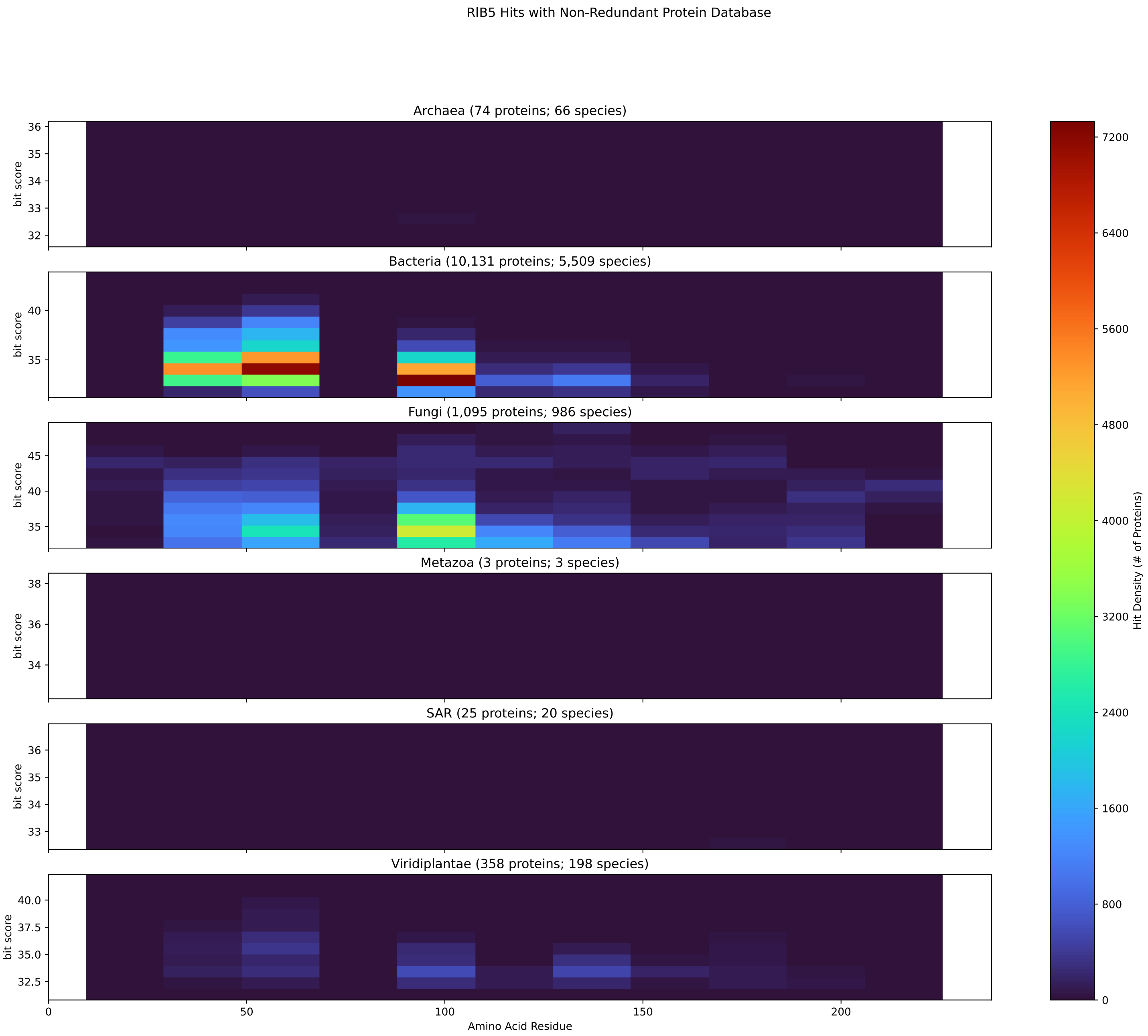

Figure S152: Non-redundant (NR) protein hits for DEG20010082/Rib5 at 20 amino acid length queries.

S2.14 Ssy1

S2.14.1 WHO Critical Pathogens

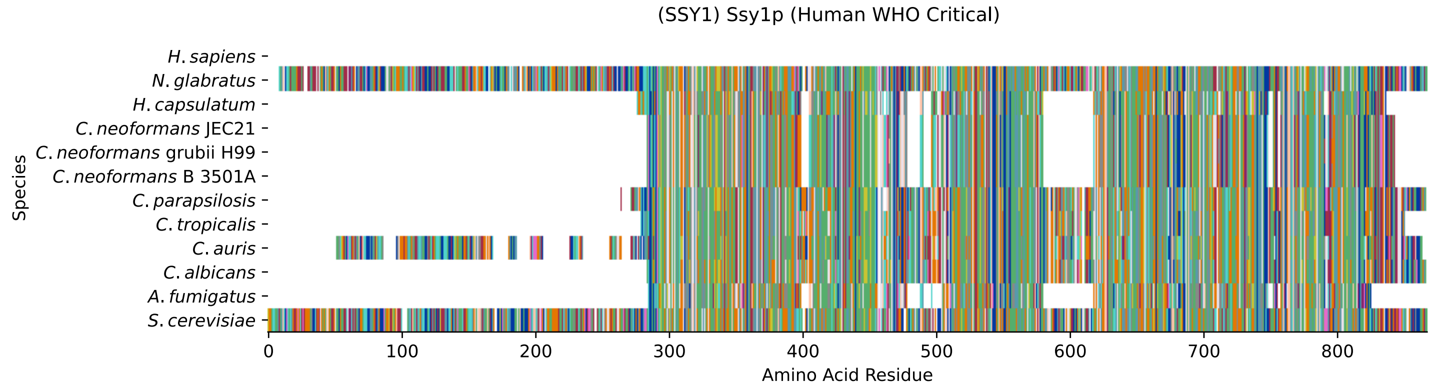

Figure S153: Multiple sequence alignment of yeast Ssy1 (WHO Critical Pathogens). Cf. Figure S154 for alignment quality, and Figure S155 for Sneath similarity. Cf. Table S32 for protein names, and pairwise alignment metrics with yeast Ssy1.

SSY1 MSA Quality

	A. f.	*C. a.*	*C. a.*	*C. p.*	*C. t.*	*C. n.*	*C. n.*	*C. n.*	*H. c.*	*N. g.*	*S. c.*
A. fumigatus	937.176	269.626	234.187	231.106	264.618	449.129	446.817	449.129	357.836	231.491	239.58
C. albicans	269.626	1174.84	806.209	907.516	1003.43	310.842	311.997	310.842	258.07	453.366	472.241
C. auris	244.588	822.002	1503.03	770.77	818.92	316.235	315.464	316.235	264.618	480.715	485.337
C. parapsilosis	240.35	926.006	771.926	1199.11	913.294	302.753	303.138	302.753	262.692	451.825	462.611
C. tropicalis	264.618	1003.43	803.897	899.042	1139.41	317.775	320.087	317.775	254.603	466.077	475.322
C. neoformans B 3501A	449.129	310.842	306.99	291.967	317.775	998.423	984.941	998.423	444.891	304.679	304.679
C. neoformans grubii H99	446.817	311.997	306.22	291.967	320.087	984.941	998.038	984.941	444.121	308.916	303.138
C. neoformans JEC21	449.129	310.842	306.99	291.967	317.775	998.423	984.941	998.423	444.891	304.679	304.679
H. capsulatum	357.836	258.07	259.225	253.447	254.603	444.891	444.121	444.891	984.941	247.284	257.299
N. glabratus	237.654	461.455	478.019	446.432	473.781	317.005	320.857	317.005	256.914	1739.16	922.924
S. cerevisiae	239.58	472.241	472.626	450.284	475.322	304.679	303.138	304.679	257.299	901.353	1758.42

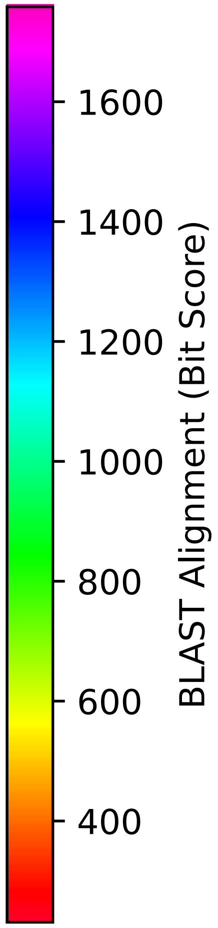

Figure S154: Multiple sequence alignment quality of Ssy1 (WHO Critical Pathogens). Cf. Figure S153

Species	Hit Protein	Hit Length (a.a.)	evalue	align_len	bit_score	identity	positive	score	gaps	% identity	% positive
H.sapiens	-	-	-	-	-	-	-	-	-	-	-
N.glabratus	XP_445735.1 uncharacterized p-rotein CAGL0E01089g Nakaseomyc-es glabratus	857	0	857	901.353	457	606	2328	23	53.6	71.1
H.capsulatum	XP_045289907.1 arginine perme-ase Histoplasma capsulatum G18-6AR	553	1.9e-77	553	260.381	174	280	664	62	20.4	32.9
C.neoformans.JEC21	XP_568394.1 amino acid transp-orter, putative Cryptococcus n-eoformans var. neoformans JEC2-1	555	6.9e-95	555	307.76	181	300	787	61	21.2	35.2
C.neoformans.grubii.H99	XP_012052990.1 AAT family ami-no acid transporter Cryptococc-us neoformans var. grubii H99	555	1.9e-94	555	306.605	183	298	784	61	21.5	35.0
C.neoformans.B.3501A	XP_772149.1 hypothetical prot-ein CNBM0690 Cryptococcus neof-ormans var. neoformans B-3501A	555	6.6e-95	555	307.76	181	300	787	61	21.2	35.2
C.parapsilosis	XP_036666456.1 uncharacterize-d protein CPAR2 209340 Candida parapsilosis	603	7e-150	603	457.988	249	365	1177	24	29.2	42.8
C.tropicalis	XP_002547148.1 hypothetical p-rotein CTRG 01454 Candida trop-icalis MYA-3404	575	3.8e-159	575	485.337	251	359	1248	19	29.5	42.1
C.auris	XP_028890914.2 hypothetical p-rotein Candida auris	817	8.1e-156	817	473.396	290	439	1217	104	34.0	51.5
C.albicans	XP_720938.2 Ssy1p Candida alb-icans SC5314	589	2.9e-157	589	480.33	254	374	1235	24	29.8	43.9
A.fumigatus	XP_748191.1 amino acid permea-se, putative Aspergillus fumig-atus Af293	535	7.6e-72	535	245.743	153	267	626	66	18.0	31.3

Table S32: Pairwise alignment info from yeast Ssy1 (DEG20010185), cf. Figure S153.

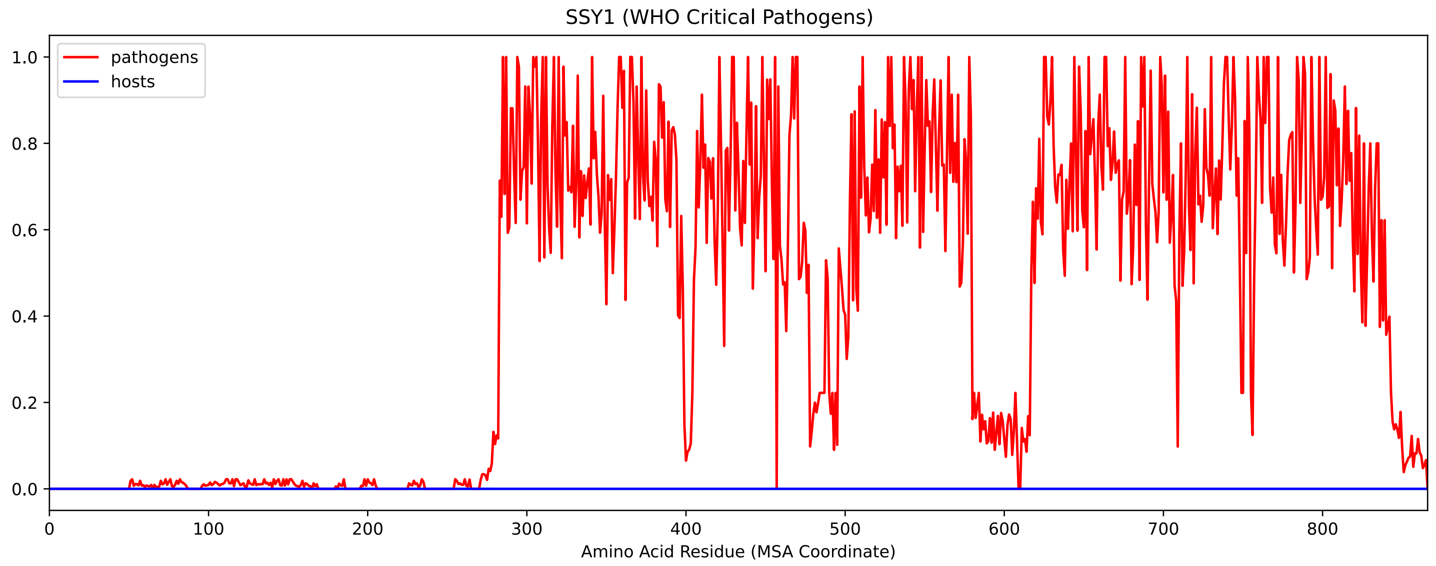

Figure S155: Sneath Similarity of Ssy1 for WHO Critical Pathogens, cf. Figure S153

S2.14.2 Top 10 Agricultural Fungal Pathogens

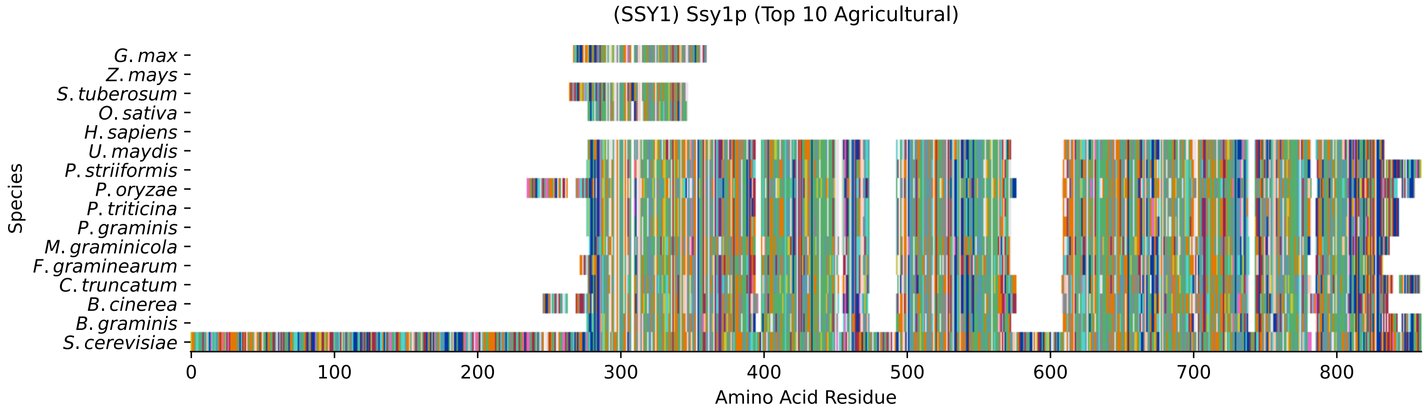

Figure S156: Multiple sequence alignment of yeast Ssy1 (Top 10 Agricultural Fungal Pathogens). Cf. Figure S157 for alignment quality, and Figure S158 for Sneath similarity. Cf. Table S33 for protein names, and pairwise alignment metrics with yeast Ssy1.

Ssy1 MSA Quality

	B. g.	*B. c.*	*C. t.*	*F. g.*	*M. g.*	*P. g.*	*P. t.*	*P. o.*	*P. s.*	*U. m.*	*S. c.*
B. graminis	1013.06	364.77	390.193	539.265	299.671	444.506	432.18	374.4	452.21	357.066	218.779
B. cinerea	369.007	1056.2	457.218	362.459	355.525	472.626	449.514	350.132	481.871	430.639	256.529
C. truncatum	402.905	458.373	1027.7	385.185	355.525	469.929	447.973	359.762	480.715	410.223	268.085
F. graminearum	539.265	351.673	373.629	980.319	308.145	434.106	424.476	354.369	436.802	373.629	250.366
M. graminicola	307.375	363.999	355.525	319.701	970.689	420.624	411.764	288.5	434.876	444.891	250.751
P. graminis	444.506	465.692	455.292	434.106	407.142	999.194	884.019	399.053	904.049	473.011	289.656
P. triticina	451.44	468.389	457.218	444.121	415.616	919.072	1001.5	400.979	930.243	481.1	303.908
P. oryzae	374.4	340.502	344.739	354.369	278.1	399.053	387.882	1103.2	391.349	353.599	236.498
P. striiformis	452.21	473.781	466.848	436.802	422.165	904.049	895.575	391.349	1031.55	479.174	295.434
U. maydis	375.941	444.506	410.223	392.504	447.588	491.115	473.011	364.77	496.123	968.378	273.092
S. cerevisiae	218.779	253.447	261.151	250.366	240.736	289.656	290.426	236.498	295.434	263.462	1758.42

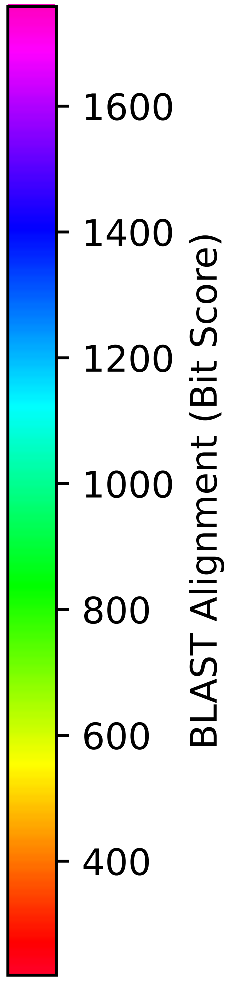

Figure S157: Multiple sequence alignment quality of Ssy1 (Top 10 Agricultural Fungal Pathogens). Cf. Figure S156

Species	Hit Protein	Hit Length (a.a.)	evalue	align_len	bit_score	identity	positive	score	gaps	% identity	% positive
G.max	XP_003555221.1 cationic amino acid transporter 7, chloroplastic Glycine max	92	0.019	92	39.6614	29	46	91	2	3.4	5.4
Z.mays	-	-	-	-	-	-	-	-	-	-	-
S.tuberosum	XP_006353075.1 PREDICTED: cationic amino acid transporter 1--like Solanum tuberosum	90	0.00083	90	43.1282	29	49	100	9	3.4	5.8
O.sativa	XP_015627412.1 cationic amino acid transporter 9, chloroplastic Oryza sativa Japonica Group	69	0.05	69	37.7354	22	35	86	1	2.6	4.1
H.sapiens	-	-	-	-	-	-	-	-	-	-	-
U.maydis	XP_011389724.1 putative general amino acid permease Ustilago maydis 521	552	2e-79	552	266.159	171	276	679	68	20.1	32.4
P.striiformis	XP_047811281.1 hypothetical protein Pst134EA 002462 Puccinia striiformis f. sp. tritici	578	1.1e-91	578	299.286	180	295	765	67	21.1	34.6
P.oryzae	mRNA M BR32 EuGene 00049831-p1 — transcript=mRNA M BR32 EuGene 00049831 — gene=M BR32 EuGene 00049831 — organism=Pyricularia oryzae BR32 — gene product=unspecified product — transcript product=unspecified product — location=BR32 scaffold000-04:1622099-1624325(-) — protein length=549 — sequence SO=supercontig — SO=protein coding gene — is pseudo=false	610	8.1e-69	610	236.884	168	287	603	71	19.7	33.7
P.triticina	XP_053023344.1 uncharacterized protein PtA15 8A695 Puccinia triticina	561	5.9e-90	561	294.278	180	288	752	66	21.1	33.8
P.graminis	XP_003329009.2 AAT family amino acid transporter Puccinia graminis f. sp. tritici CRL 75–36-700-3	561	4.1e-89	561	292.352	170	292	747	66	20.0	34.3
M.graminicola	ZTRI 4.872.mRNA-p1 — transcript=ZTRI 4.872.mRNA — gene=ZTRI 4.872 — organism=Zymoseptoria tritici IPO323 — gene product=-similar to amino acid permease — transcript product=similar to amino acid permease — location=Ztri chr 4:2785263-2787024-(-) — protein length=550 — sequence SO=chromosome — SO=protein coding gene — is pseudo=false	555	2.5e-71	555	243.817	171	281	621	69	20.1	33.0
F.graminearum	XP_011326118.1 hypothetical protein FGSG 06508 Fusarium graminearum PH-1	554	3.5e-75	554	253.447	173	272	646	67	20.3	31.9
C.truncatum	XP_036576509.1 amino acid permease Colletotrichum truncatum	575	9.6e-79	575	265.003	172	285	676	65	20.2	33.5
B.cinerea	XP_001552845.1 hypothetical protein BCIN 14g01410 Botrytis cinerea B05.10	589	9.5e-76	589	256.914	172	289	655	71	20.2	33.9
B.graminis	VDB92912.1 — transcript=BGT962-24V316 LOCUS6671 t1 — gene=BGT-96224V316 LOCUS6671 — organism=Blumeria graminis f. sp. tritici 96224 — gene product=unspecified product — transcript product=unspecified product — location=LR026992:5342891-534455-2(+) — protein length=536 — sequence SO=chromosome — SO=protein coding gene — is pseudo=false	584	3.1e-64	584	223.402	162	281	568	83	19.0	33.0

Table S33: Pairwise alignment info from yeast Ssy1 (DEG20010185), cf. Figure S156.

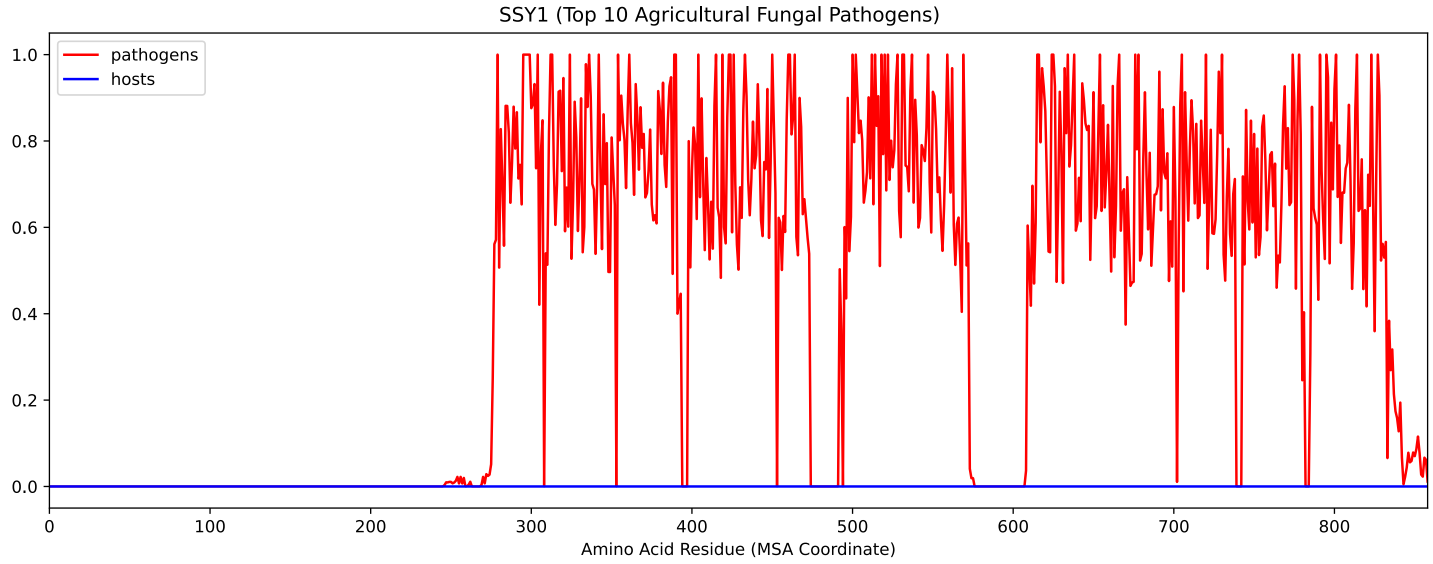

Figure S158: Sneath Similarity of Ssy1 for Top 10 Agricultural Fungal Pathogens, cf. Figure S156

S2.14.3 NR

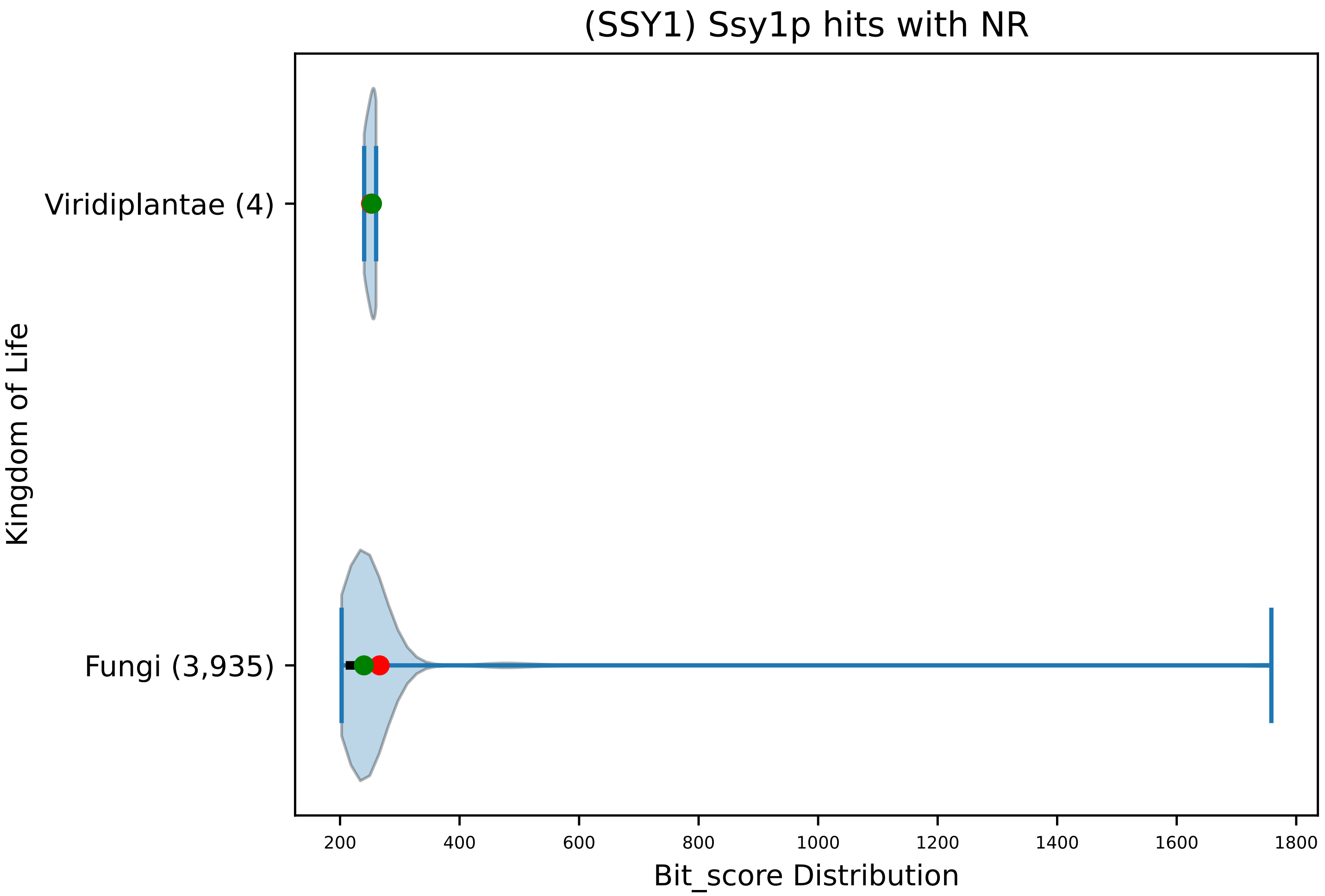

Figure S159: Non-redundant (NR) protein hits for DEG20010185/Ssy1, with expectation value of no more than 0.1. Green points are medians, and red points are arithmetic means.

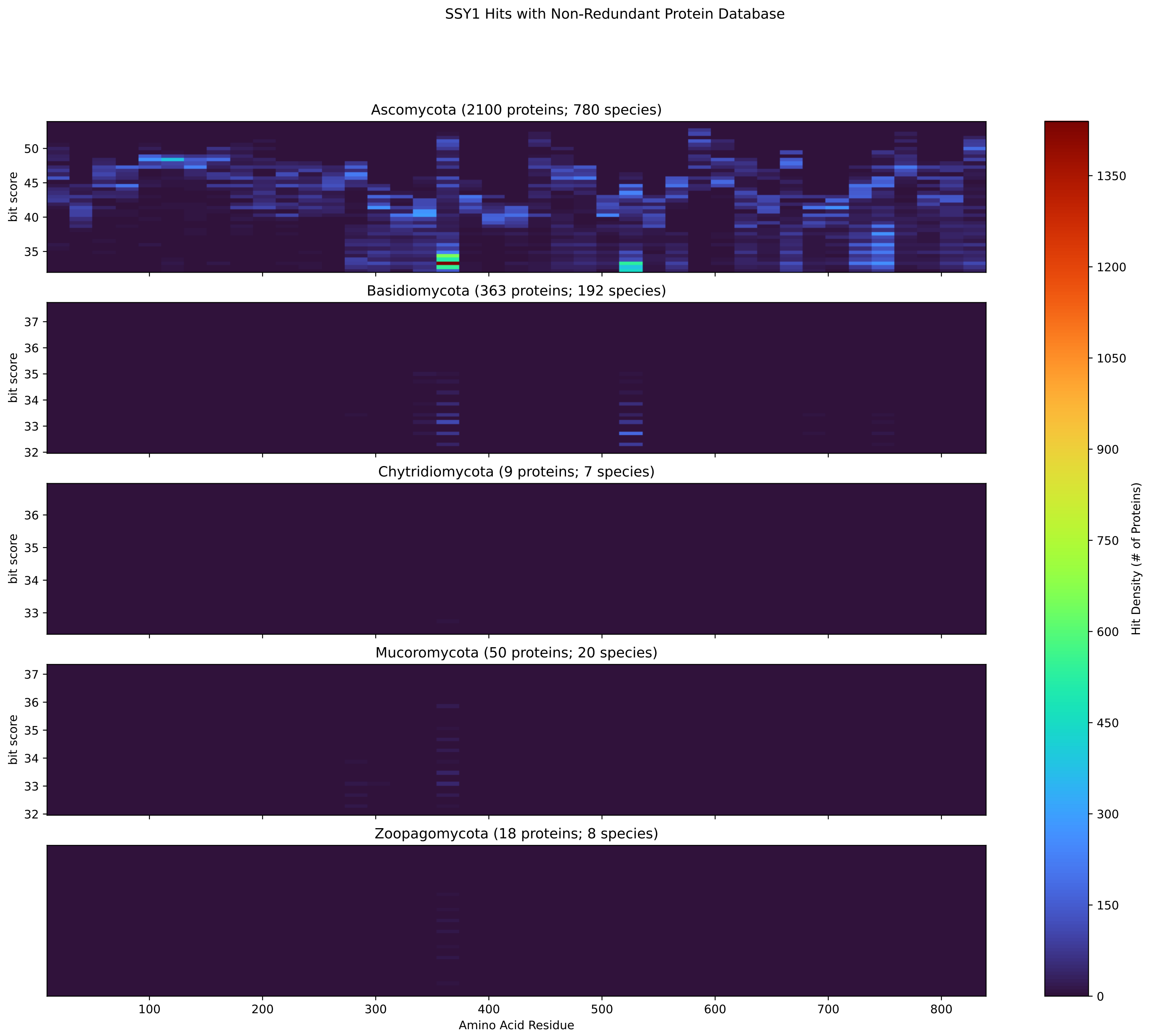

Figure S160: Non-redundant (NR) protein hits for Ssy1 in the kingdom Fungi.

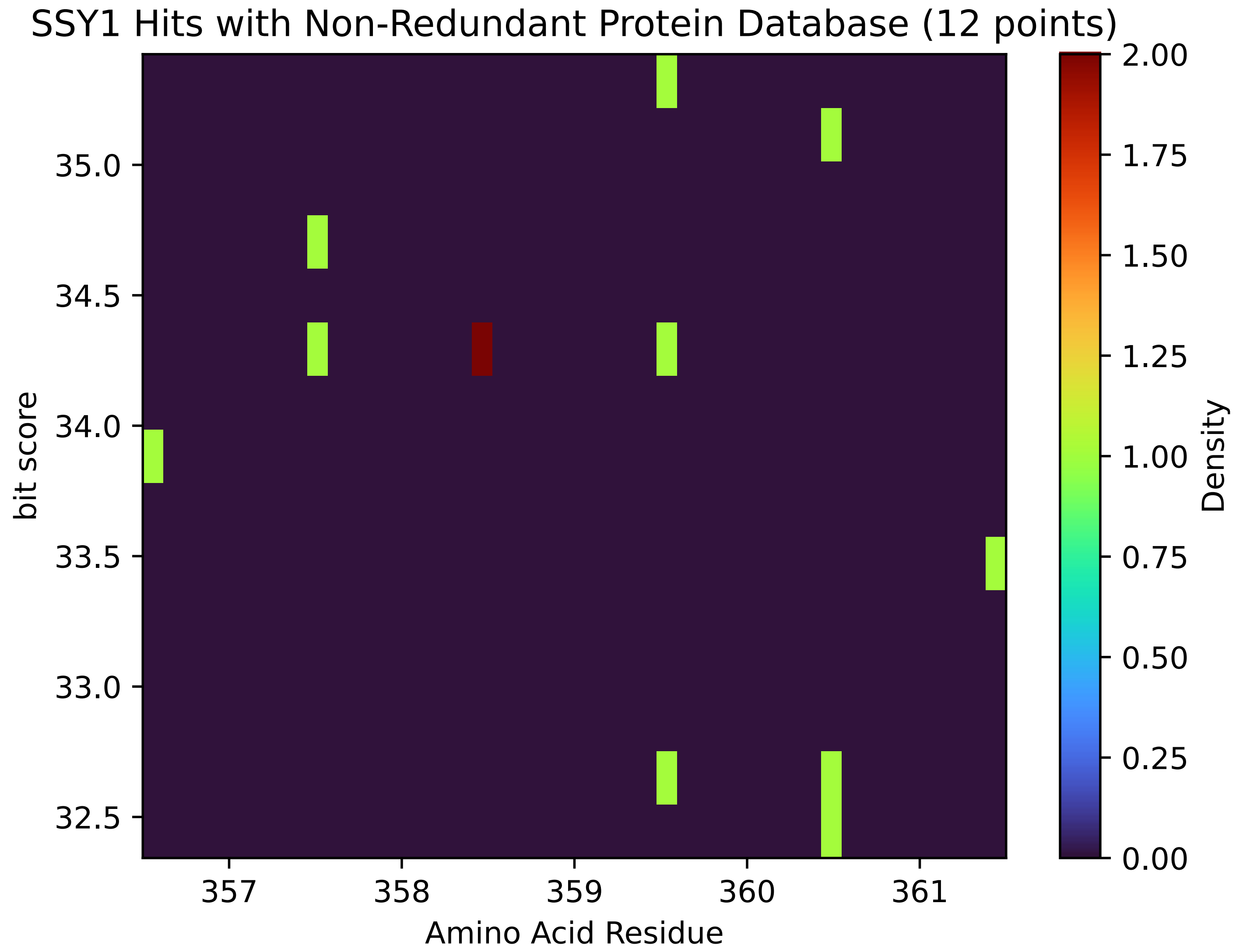

Figure S161: Non-redundant (NR) protein hits for Ssy1 in the kingdom Metazoa.

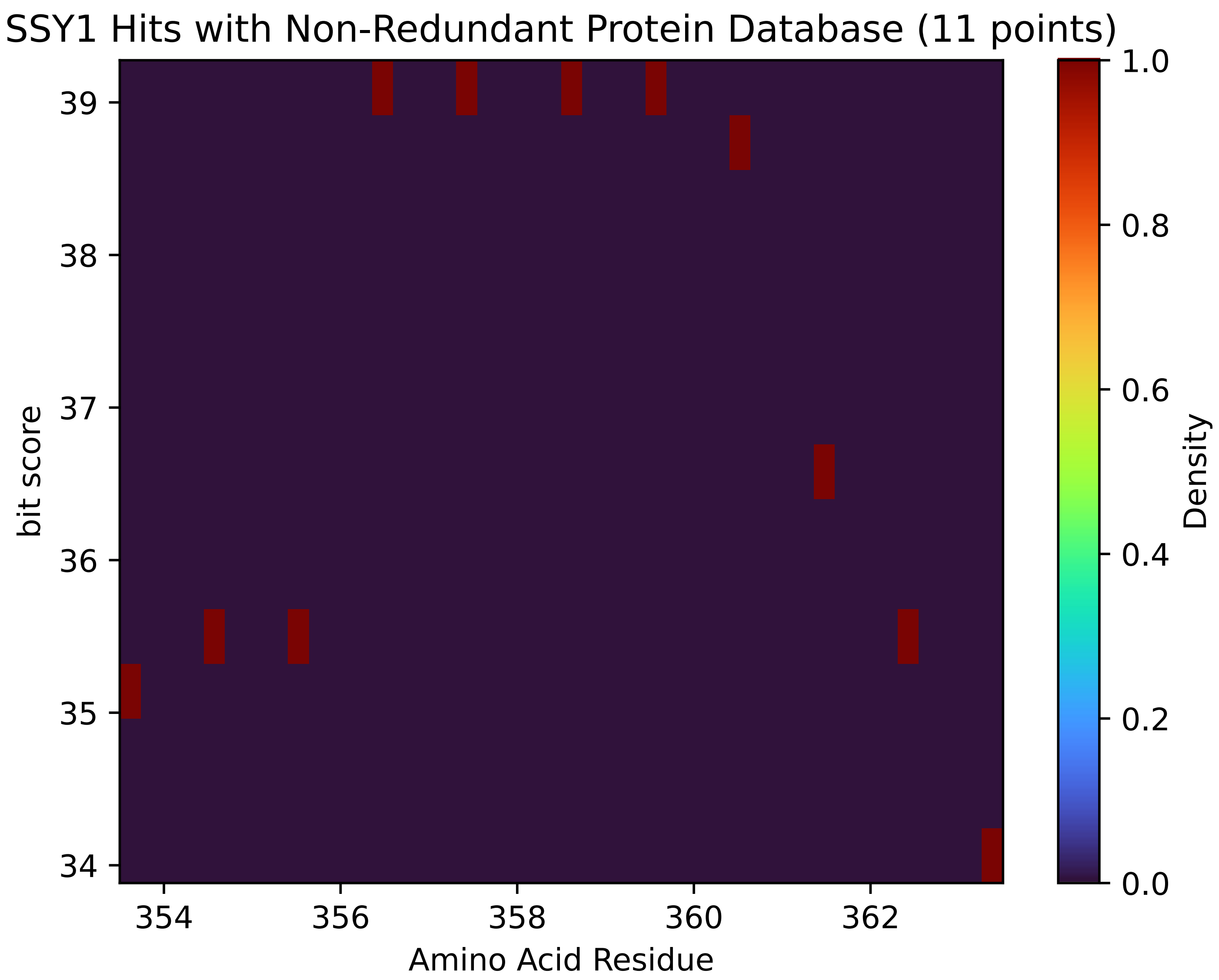

Figure S162: Non-redundant (NR) protein hits for Ssy1 in the kingdom SAR.

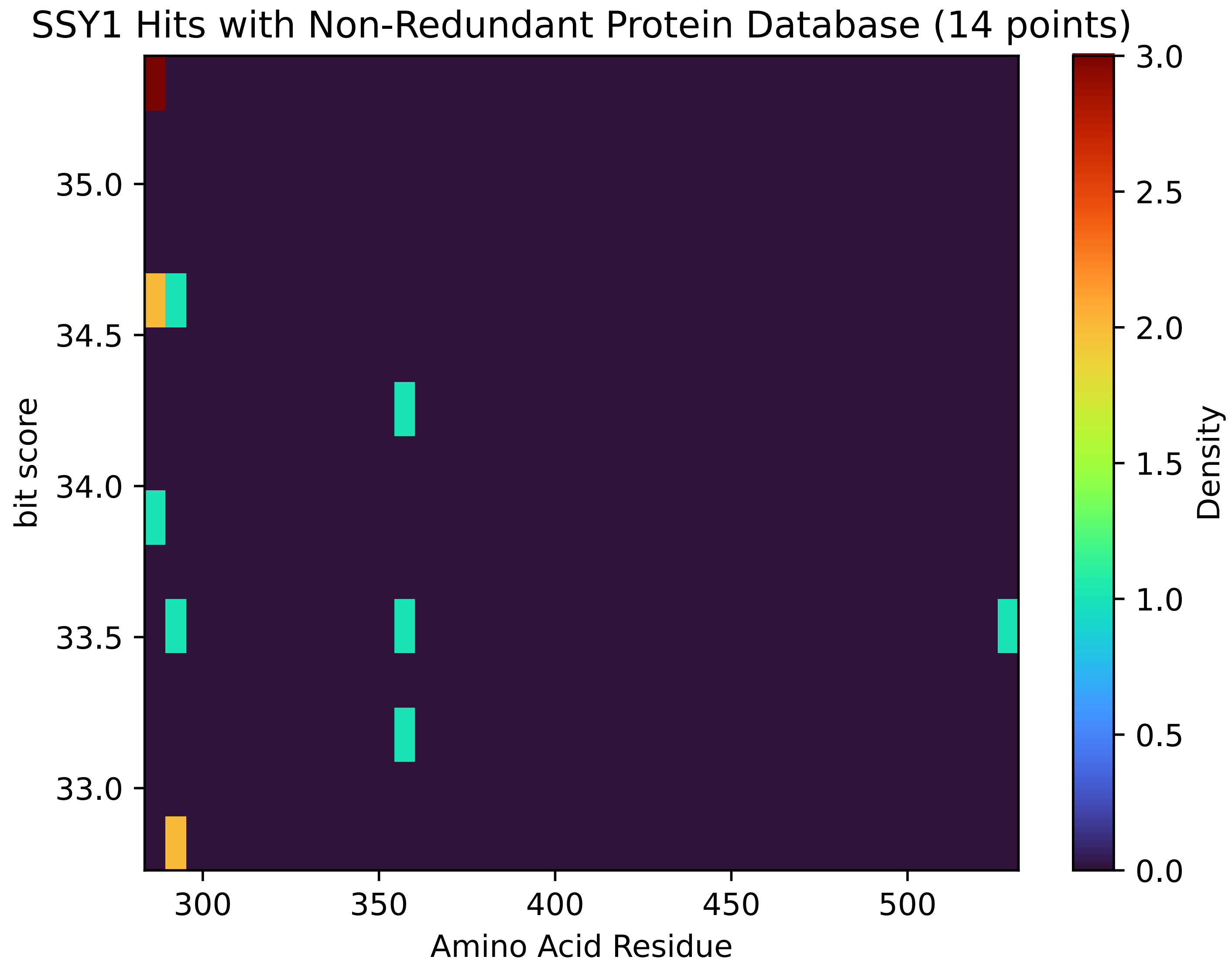

Figure S163: Non-redundant (NR) protein hits for Ssy1 in the kingdom Viridiplantae.

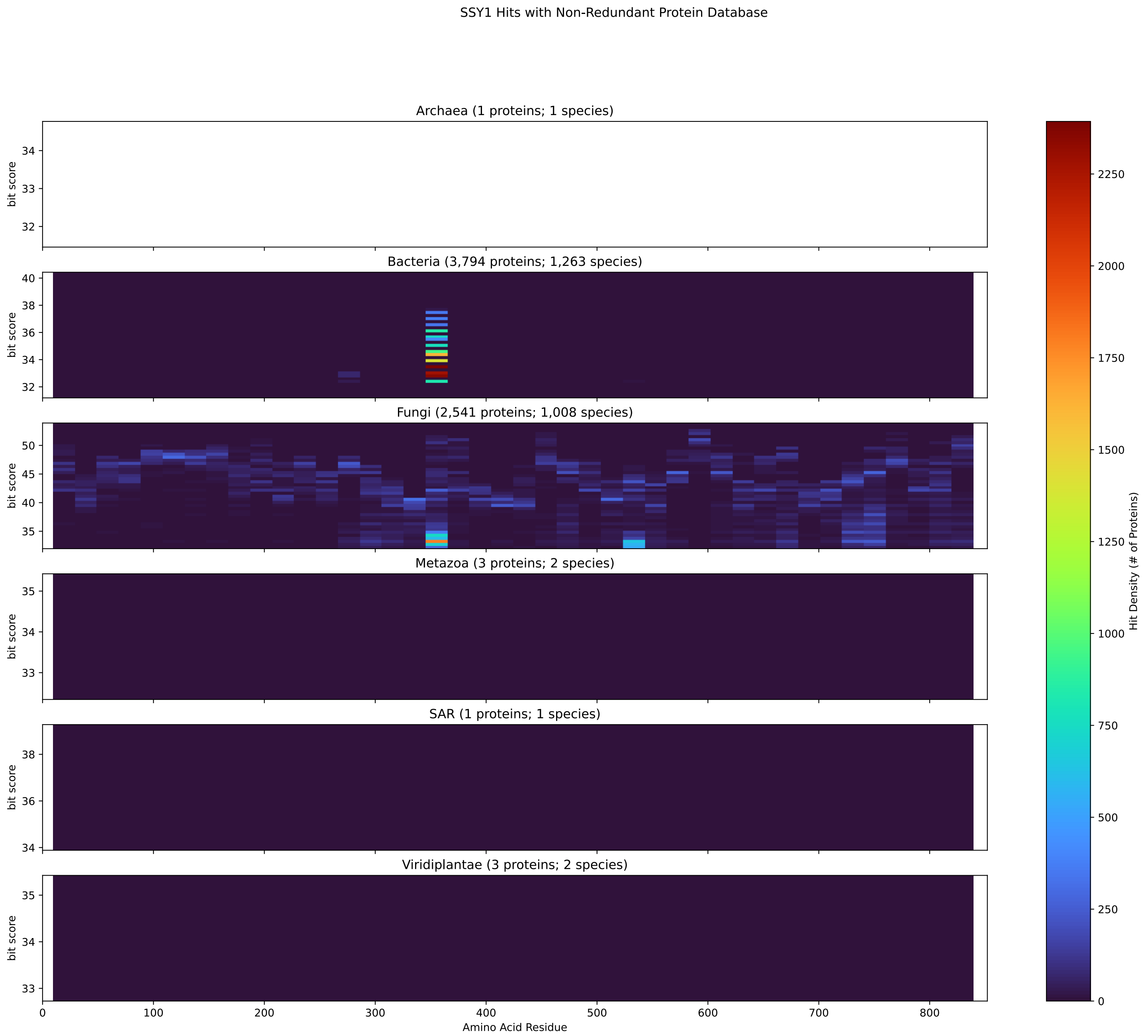

Figure S164: Non-redundant (NR) protein hits for DEG20010185/Ssy1 at 20 amino acid length queries.

S2.15 Ste12

S2.15.1 WHO Critical Pathogens

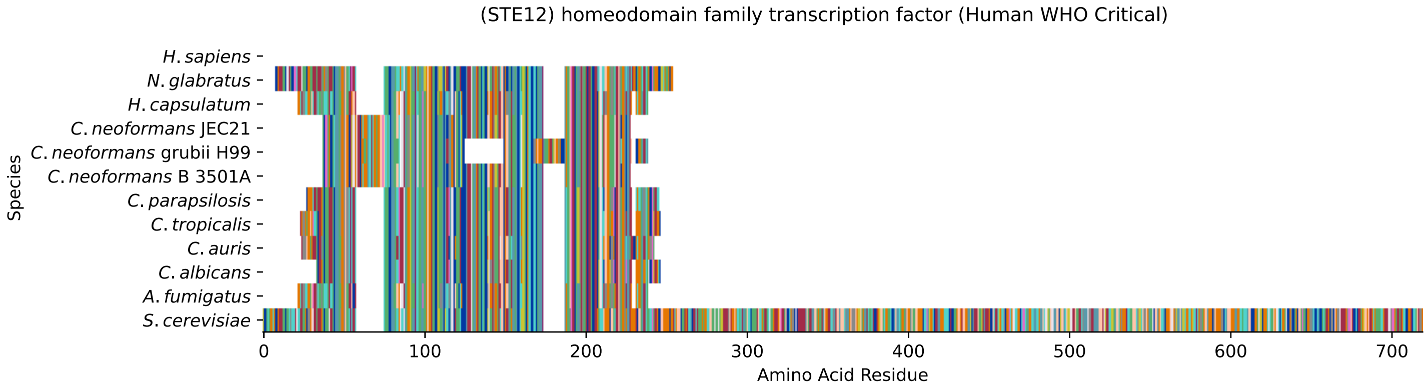

Figure S165: Multiple sequence alignment of yeast Ste12 (WHO Critical Pathogens). Cf. Figure S166 for alignment quality, and Figure S167 for Sneath similarity. Cf. Table S34 for protein names, and pairwise alignment metrics with yeast Ste12.

Ste12 MSA Quality

	A. f.	*C. a.*	*C. a.*	*C. p.*	*C. t.*	*C. n.*	*C. n.*	*C. n.*	*H. c.*	*N. g.*	*S. c.*
A. fumigatus	382.104	234.958	244.588	232.261	238.424	235.343	164.081	235.343	358.992	222.246	236.113
C. albicans	234.958	382.874	309.301	352.058	367.081	197.978	135.191	197.978	231.106	276.174	280.796
C. auris	244.588	309.301	392.889	305.449	314.694	198.364	132.109	198.364	239.58	272.322	277.715
C. parapsilosis	232.261	352.058	305.449	389.037	358.221	199.134	137.117	199.134	233.032	275.789	276.559
C. tropicalis	238.424	367.081	314.694	358.221	401.749	199.134	135.191	199.134	233.032	279.256	283.878
C. neoformans B 3501A	235.343	197.978	198.364	199.134	199.134	368.237	273.863	368.237	238.81	197.593	192.971
C. neoformans grubii H99	164.081	135.191	132.109	137.117	135.191	273.863	362.073	273.863	167.548	131.339	121.709
C. neoformans JEC21	235.343	197.978	198.364	199.134	199.134	368.237	273.863	368.237	238.81	197.593	192.971
H. capsulatum	358.992	231.106	239.58	233.032	233.032	238.81	167.548	238.81	380.563	218.009	233.802
N. glabratus	222.246	276.174	272.322	275.789	279.256	197.593	131.339	197.593	218.009	448.358	310.457
S. cerevisiae	236.113	280.796	277.715	276.559	283.878	192.971	121.709	192.971	233.802	310.457	1425.22

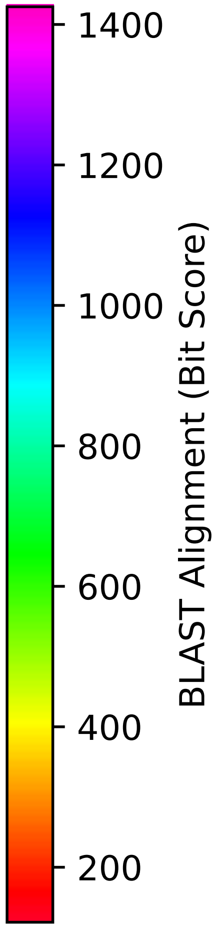

Figure S166: Multiple sequence alignment quality of Ste12 (WHO Critical Pathogens). Cf. Figure S165

Species	Hit Protein	Hit Length (a.a.)	evalue	align_len	bit_score	identity	positive	score	gaps	% identity	% positive
H.sapiens	-	-	-	-	-	-	-	-	-	-	-
N.glabratus	XP_449399.1 uncharacterized p-rotein CAGL0M01254g Nakaseomyc-es glabratus	216	4.4e-101	216	320.087	144	180	819	1	20.9	26.2
H.capsulatum	XP_045286576.1 transcription factor steA Histoplasma capsul-atum G186AR	187	2.3e-71	187	244.588	109	147	623	3	15.8	21.4
C.neoformans.JEC21	XP_024512781.1 conserved hypo-thetical protein Cryptococcus neoformans var. neoformans JEC-21	178	8.1e-56	178	202.986	97	122	515	18	14.1	17.7
C.neoformans.grubii.H99	XP_012049551.1 transcription factor STE12 Cryptococcus neof-ormans var. grubii H99	202	9.3e-32	202	130.568	78	104	327	57	11.3	15.1
C.neoformans.B.3501A	XP_776009.1 hypothetical prot-ein CNBD0580 Cryptococcus neof-ormans var. neoformans B-3501A	178	9.2e-56	178	202.601	97	122	514	18	14.1	17.7
C.parapsilosis	XP_036666384.1 uncharacterize-d protein CPAR2 208600 Candida parapsilosis	189	2.6e-88	189	287.345	129	157	734	2	18.8	22.8
C.tropicalis	XP_002549862.1 transcription factor CPH1 Candida tropicalis MYA-3404	194	2.9e-89	194	290.041	132	156	741	2	19.2	22.7
C.auris	XP_028890469.2 protein STE12 Candida auris	189	1.6e-87	189	281.952	126	152	720	0	18.3	22.1
C.albicans	XP_713877.1 homeodomain famil-y transcription factor Candida albicans SC5314	184	2.5e-89	184	290.812	129	154	743	2	18.8	22.4
A.fumigatus	XP_754013.1 sexual developmen-t transcription factor SteA As-pergillus fumigatus Af293	187	3.9e-72	187	246.128	110	147	627	3	16.0	21.4

Table S34: Pairwise alignment info from yeast Ste12 (DEG20010472), cf. Figure S165.

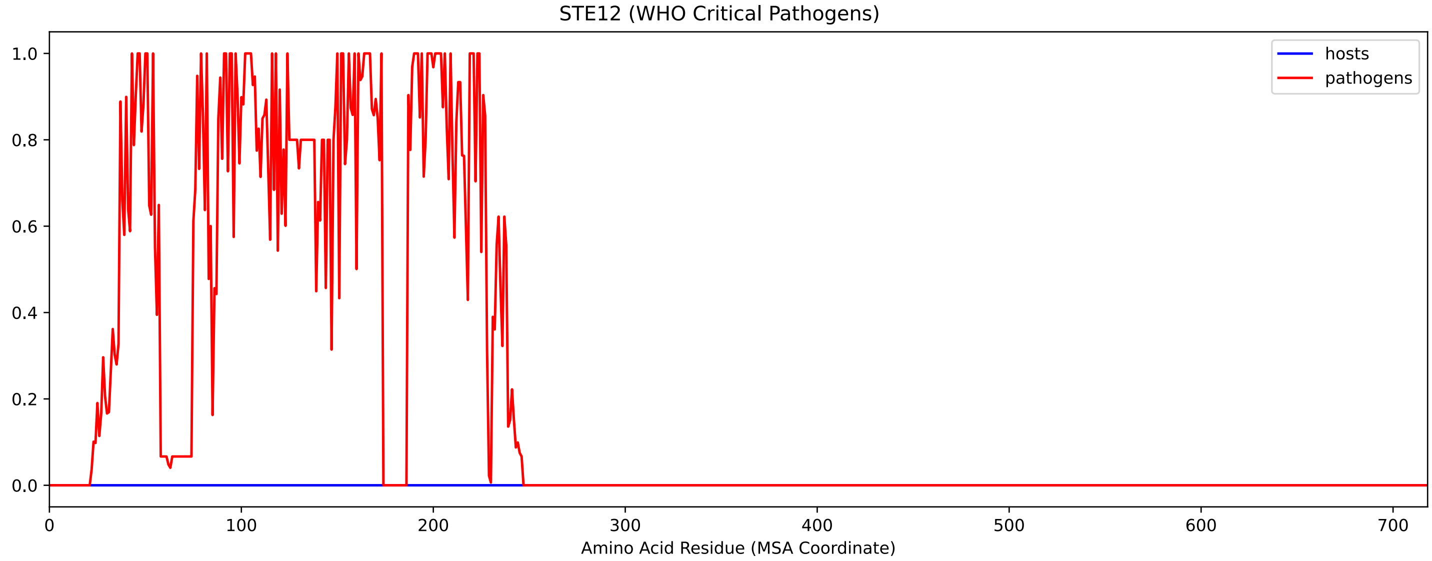

Figure S167: Sneath Similarity of Ste12 for WHO Critical Pathogens, cf. Figure S165

S2.15.2 Top 10 Agricultural Fungal Pathogens

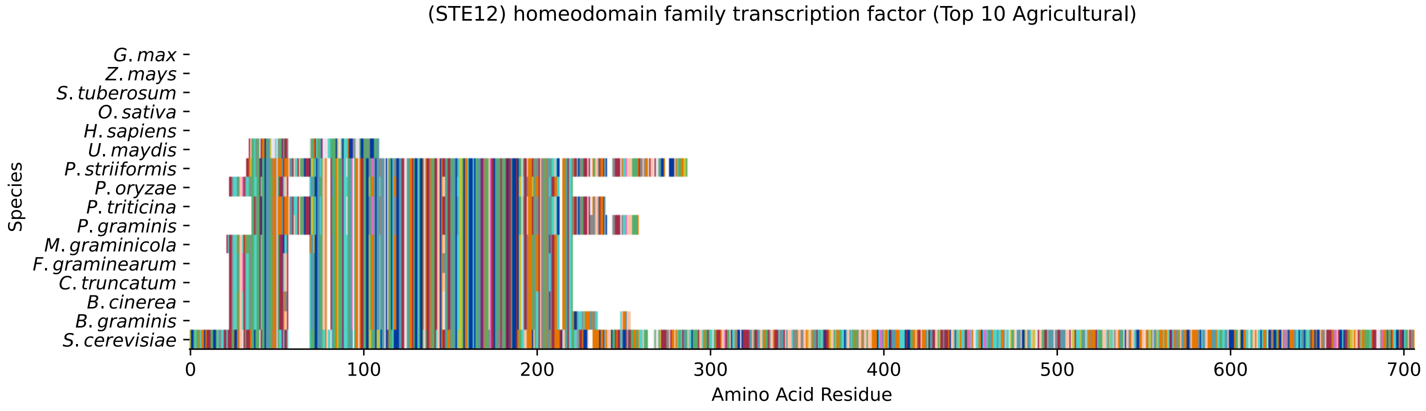

Figure S168: Multiple sequence alignment of yeast Ste12 (Top 10 Agricultural Fungal Pathogens). Cf. Figure S169 for alignment quality, and Figure S170 for Sneath similarity. Cf. Table S35 for protein names, and pairwise alignment metrics with yeast Ste12.

Ste12 MSA Quality

	B. g.	B. c.	C. t.	F. g.	M. g.	P. g.	P. t.	P. o.	P. s.	U. m.	S. c.
B. graminis	418.313	375.17	364.385	365.925	357.451	254.218	254.603	363.614	259.225	0	234.958
B. cinerea	375.17	376.711	361.688	362.459	357.066	253.062	253.832	360.533	258.455	0	233.417
C. truncatum	364.385	361.688	378.252	375.941	355.525	252.677	253.832	377.096	257.299	0	237.269
F. graminearum	365.925	362.459	375.941	379.022	352.443	253.062	253.832	374.785	257.684	0	237.654
M. graminicola	357.451	357.066	355.525	352.443	379.793	251.906	253.062	354.755	256.914	0	224.557
P. graminis	254.218	253.062	252.677	253.062	251.906	444.121	403.675	253.062	433.721	0	199.519
P. triticina	254.603	253.832	253.832	253.832	253.062	403.675	413.305	254.218	402.905	0	196.438
P. oryzae	363.614	360.533	377.096	374.785	354.755	253.062	254.218	379.407	257.684	0	237.269
P. striiformis	259.225	258.455	257.299	257.684	256.914	433.721	402.905	257.684	507.294	0	204.142
U. maydis	0	15.779	16.5494	16.1642	17.705	0	0	16.5494	0	127.102	27.7202
S. cerevisiae	234.958	233.417	237.269	237.654	224.557	199.519	196.438	237.269	204.142	27.7202	1425.22

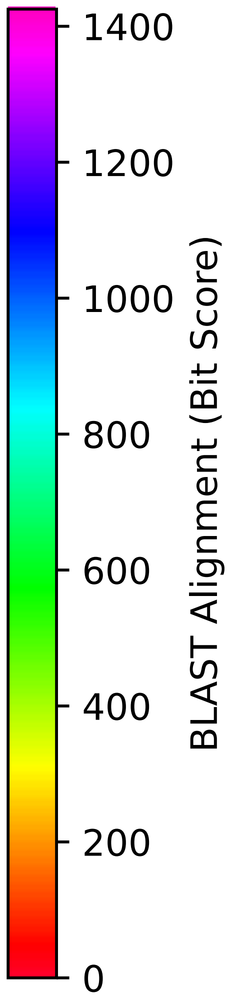

Figure S169: Multiple sequence alignment quality of Ste12 (Top 10 Agricultural Fungal Pathogens). Cf. Figure S168

Species	Hit Protein	Hit Length (a.a.)	evalue	align_len	bit_score	identity	positive	score	gaps	% identity	% positive
G.max	-	-	-	-	-	-	-	-	-	-	-
Z.mays	-	-	-	-	-	-	-	-	-	-	-
S.tuberosum	-	-	-	-	-	-	-	-	-	-	-
O.sativa	-	-	-	-	-	-	-	-	-	-	-
H.sapiens	-	-	-	-	-	-	-	-	-	-	-
U.maydis	XP_011389356.1 uncharacterize-d protein UMAG 02954 Ustilago maydis 521	65	0.034	65	35.8094	16	26	81	2	2.3	3.8
P.striiformis	XP_047804633.1 hypothetical p-rotein Pst134EA 017974 Puccini-a striiformis f. sp. tritici	253	6.8e-59	253	212.616	117	155	540	25	17.0	22.5
P.oryzae	mRNA M BR32 EuGene 00048151-p1 — transcript=mRNA M BR32 EuGe-ne 00048151 — gene=M BR32 EuGe-ne 00048151 — organism=Pyricul-aria oryzae BR32 — gene produc-t=unspecified product — transc-ript product=unspecified produ-ct — location=BR32 scaffold000-04:1144110-1146702(-) — protei-n length=764 — sequence SO=sup-ercontig — SO=protein coding g-ene — is pseudo=false	185	7.8e-72	185	246.899	112	146	629	3	16.3	21.2
P.triticina	XP_053024960.1 uncharacterize-d protein PtA15 11A92 Puccinia triticina	203	1.4e-56	203	205.682	102	137	522	17	14.8	19.9
P.graminis	XP_003325390.2 transcription factor STE12 Puccinia graminis f. sp. tritici CRL 75-36-700–3	222	8.8e-58	222	209.149	109	144	531	20	15.8	20.9
M.graminicola	ZTRI 8.206.mRNA-p1 — transcrip-t=ZTRI 8.206.mRNA — gene=ZTRI 8.206 — organism=Zymoseptoria tritici IPO323 — gene product=-similar to transcription facto-r steA — transcript product=si-milar to transcription factor steA — location=Ztri chr 8:759-315-761612(+) — protein length-=726 — sequence SO=chromosome — SO=protein coding gene — is pseudo=false	187	6.8e-68	187	235.343	105	145	599	3	15.3	21.1
F.graminearum	XP_011327056.1 transcription factor steA Fusarium graminear-um PH-1	185	8.5e-73	185	248.44	111	146	633	3	16.1	21.2
C.truncatum	XP_036586582.1 ste12-like tra-nscription factor Colletotrich-um truncatum	185	3.3e-72	185	247.284	112	146	630	3	16.3	21.2
B.cinerea	XP_024551490.1 Bcste12 Botryt-is cinerea B05.10	185	4.5e-71	185	243.047	109	145	619	3	15.8	21.1
B.graminis	VCU39448.1 — transcript=BGT962-24V316 LOCUS707 t1 — gene=BGT9-6224V316 LOCUS707 — organism=B-lumeria graminis f. sp. tritic-i 96224 — gene product=unspeci-fied product — transcript prod-uct=unspecified product — loca-tion=LR026985:958957-961327(+) — protein length=692 — sequen-ce SO=chromosome — SO=protein coding gene — is pseudo=false	205	4e-71	205	243.047	112	152	619	3	16.3	22.1

Table S35: Pairwise alignment info from yeast Ste12 (DEG20010472), cf. Figure S168.

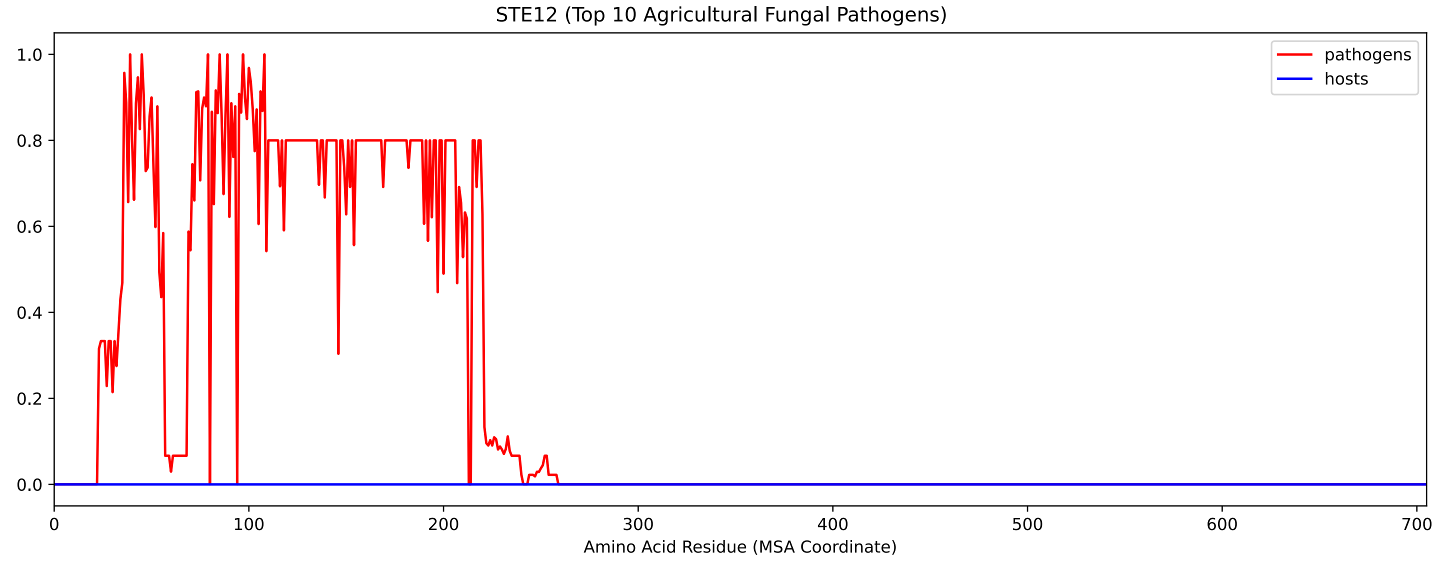

Figure S170: Sneath Similarity of Ste12 for Top 10 Agricultural Fungal Pathogens, cf. Figure S168

S2.15.3 NR

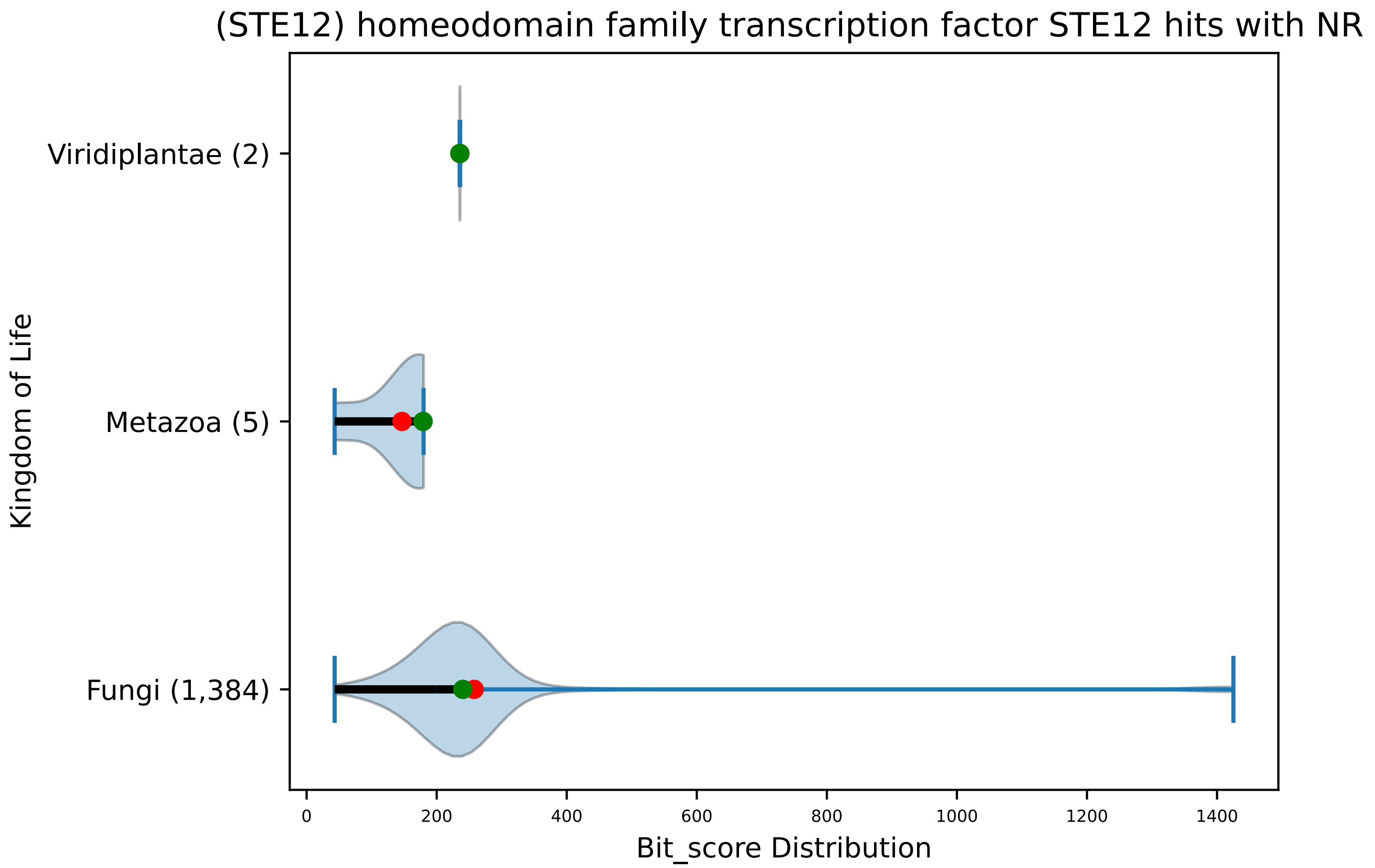

Figure S171: Non-redundant (NR) protein hits for DEG20010472/Ste12, with expectation value of no more than 0.1. Green points are medians, and red points are arithmetic means.

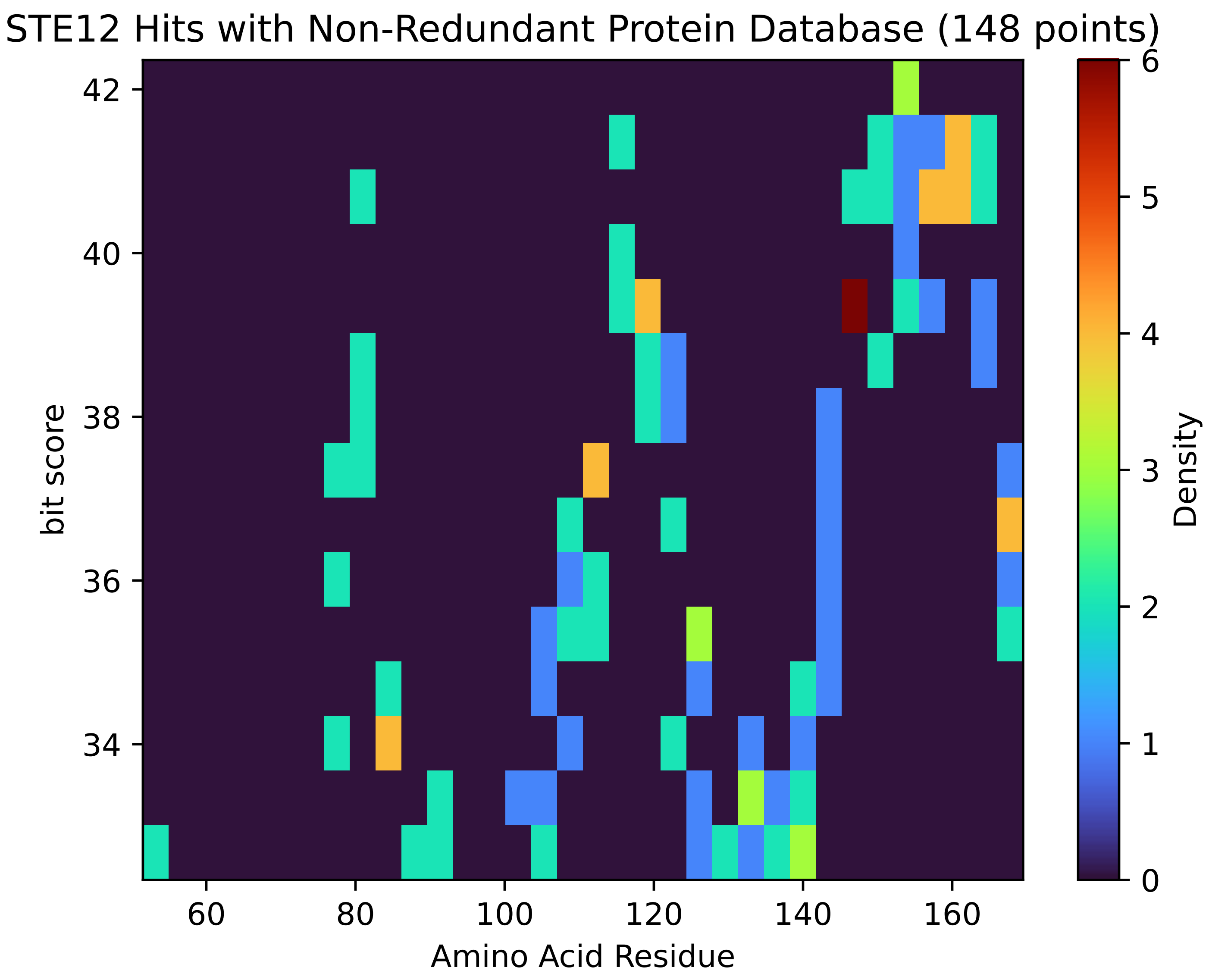

Figure S172: Non-redundant (NR) protein hits for Ste12 in the kingdom Viridiplantae.

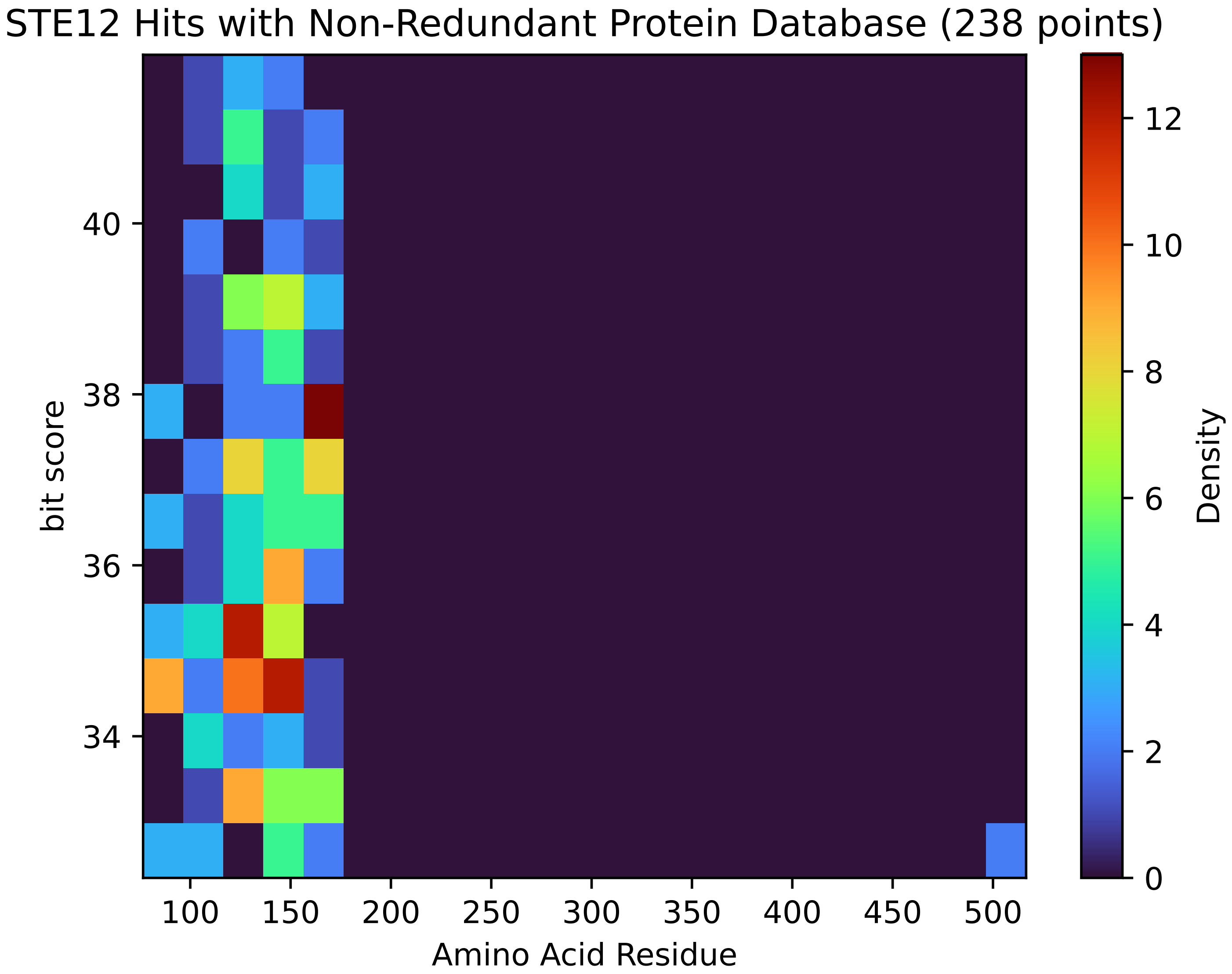

Figure S173: Non-redundant (NR) protein hits for Ste12 in the kingdom Metazoa.

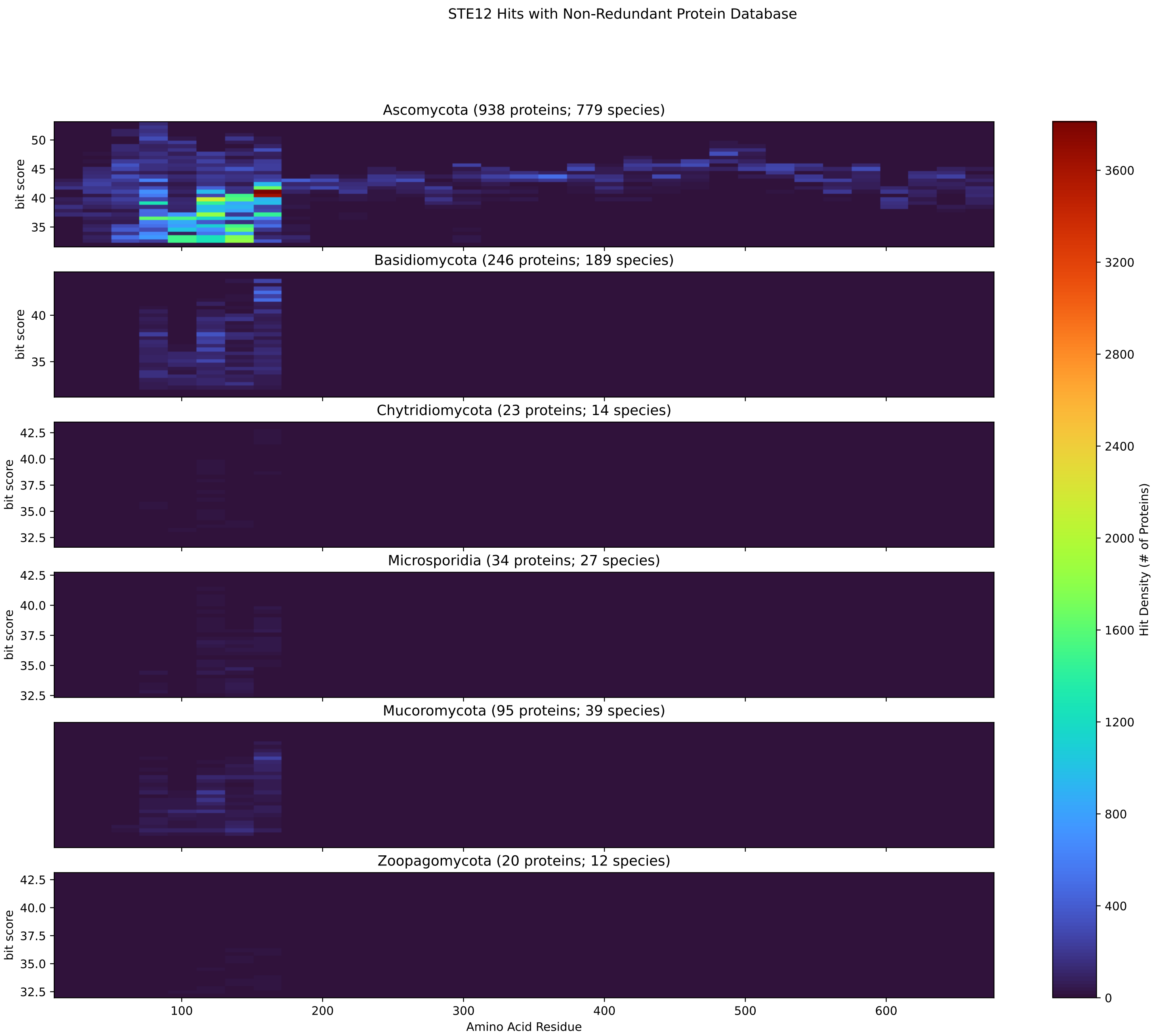

Figure S174: Non-redundant (NR) protein hits for Ste12 in the kingdom Fungi.

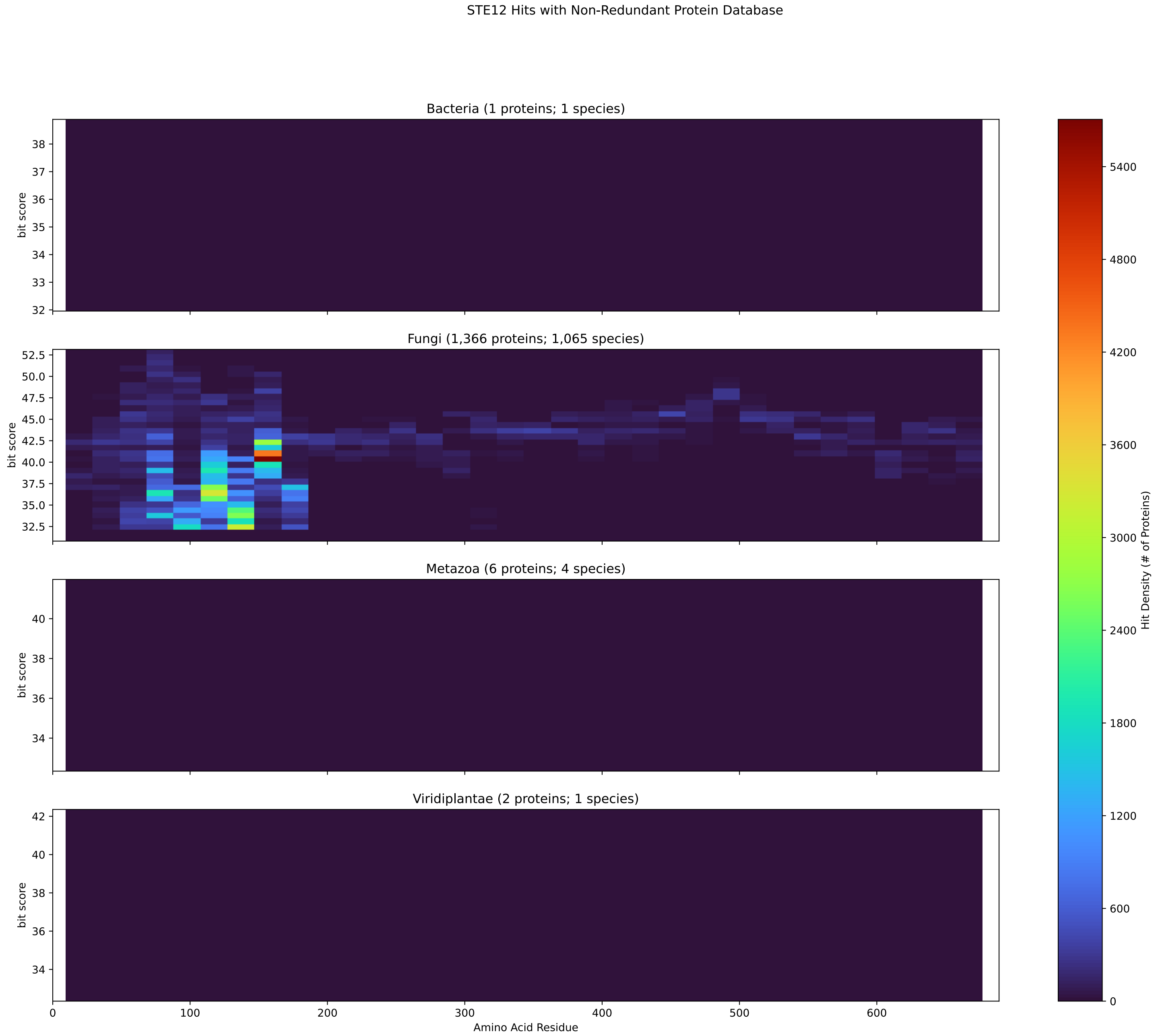

Figure S175: Non-redundant (NR) protein hits for DEG20010472/Ste12 at 20 amino acid length queries.

S2.16 Trl1

S2.16.1 WHO Critical Pathogens

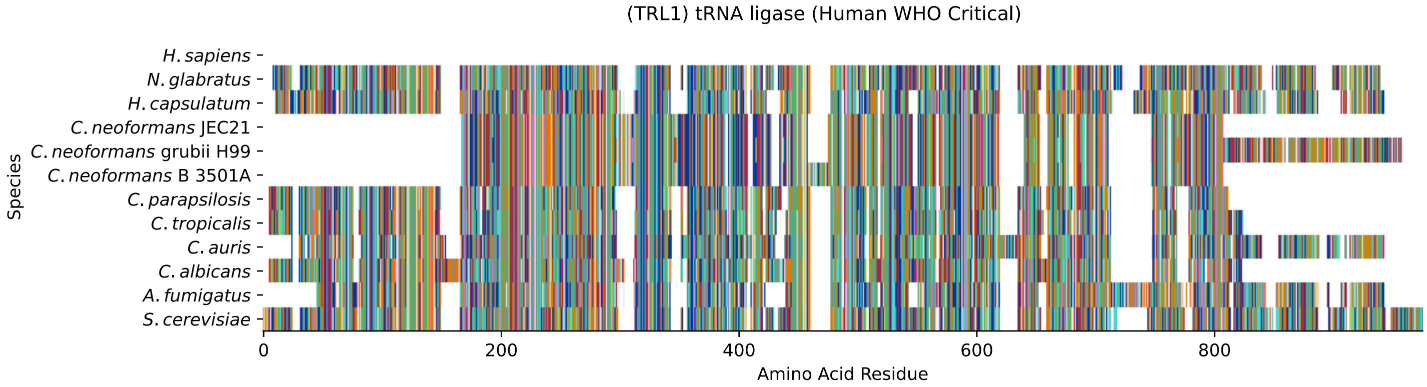

Figure S176: Multiple sequence alignment of yeast Trl1 (WHO Critical Pathogens). Cf. Figure S177 for alignment quality, and Figure S178 for Sneath similarity. Cf. Table S36 for protein names, and pairwise alignment metrics with yeast Trl1.

Trl1 MSA Quality

	A. f.	*C. a.*	*C. a.*	*C. p.*	*C. t.*	*C. n.*	*C. n.*	*C. n.*	*H. c.*	*N. g.*	*S. c.*
A. fumigatus	1573.14	430.254	384.03	458.373	428.713	225.328	238.81	236.884	1017.3	499.59	509.22
C. albicans	431.024	1447.57	436.417	848.195	911.368	203.371	216.083	214.542	434.106	509.605	520.776
C. auris	384.03	435.261	1577.76	449.514	458.373	176.792	189.889	182.956	390.578	401.749	418.313
C. parapsilosis	458.373	845.499	449.514	1348.18	871.692	177.178	183.341	186.037	467.233	523.472	524.628
C. tropicalis	428.713	908.286	458.373	871.692	1367.83	189.504	199.134	199.134	446.047	506.138	532.332
C. neoformans B 3501A	225.328	203.756	176.792	177.178	189.504	1146.72	1048.11	1092.03	231.106	201.83	196.823
C. neoformans grubii H99	240.736	215.698	193.741	182.956	198.749	1048.5	1409.82	1058.13	251.906	207.994	211.46
C. neoformans JEC21	236.884	214.927	182.956	186.037	199.134	1092.03	1057.36	1110.52	239.965	211.075	206.453
H. capsulatum	1017.3	433.335	390.578	467.233	446.047	231.106	250.366	239.965	1619.36	490.73	498.049
N. glabratus	499.59	509.605	401.749	523.472	506.138	201.83	207.608	211.075	490.73	1639.78	906.36
S. cerevisiae	509.22	519.62	418.313	524.628	532.332	196.823	207.223	206.453	498.049	906.36	1710.66

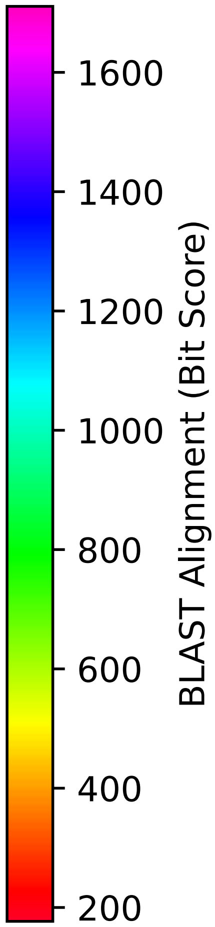

Figure S177: Multiple sequence alignment quality of Trl1 (WHO Critical Pathogens). Cf. Figure S176

Species	Hit Protein	Hit Length (a.a.)	evalue	align_len	bit_score	identity	positive	score	gaps	% identity	% positive
H.sapiens	-	-	-	-	-	-	-	-	-	-	-
N.glabratus	XP_449481.1 uncharacterized protein CAGL0M03091g Nakaseomyces glabratus	804	0	804	906.746	450	572	2342	30	54.4	69.2
H.capsulatum	XP_045288514.1 tRNA ligase Histoplasma capsulatum G186AR	819	1.2e-164	819	496.893	308	457	1278	76	37.2	55.3
C.neoformans.JEC21	XP_024512786.1 RNA ligase (ATP), putative Cryptococcus neoformans var. neoformans JEC21	567	5.4e-56	567	206.838	157	280	525	67	19.0	33.9
C.neoformans.grubii.H99	XP_012049418.1 tRNA ligase Cryptococcus neoformans var. grubii H99	713	5.8e-56	713	207.223	183	326	526	81	22.1	39.4
C.neoformans.B.3501A	XP_775856.1 hypothetical protein CNBD2650 Cryptococcus neoformans var. neoformans B-3501A	582	6.9e-53	582	197.593	157	283	501	80	19.0	34.2
C.parapsilosis	XP_036664316.1 uncharacterized protein CPAR2 301270 Candida parapsilosis	680	7.9e-176	680	524.628	299	415	1350	31	36.2	50.2
C.tropicalis	XP_002550758.1 tRNA ligase Candida tropicalis MYA-3404	693	5.2e-179	693	533.102	295	427	1372	33	35.7	51.6
C.auris	XP_028888635.2 tRNA ligase Candida auris	806	2.7e-134	806	417.927	282	431	1073	79	34.1	52.1
C.albicans	XP_721349.1 tRNA ligase Candida albicans SC5314	722	2.3e-173	722	519.62	302	426	1337	57	36.5	51.5
A.fumigatus	XP_752336.1 tRNA ligase Aspergillus fumigatus Af293	802	3.4e-169	802	509.605	312	441	1311	94	37.7	53.3

Table S36: Pairwise alignment info from yeast Trl1 (DEG20010555), cf. Figure S176.

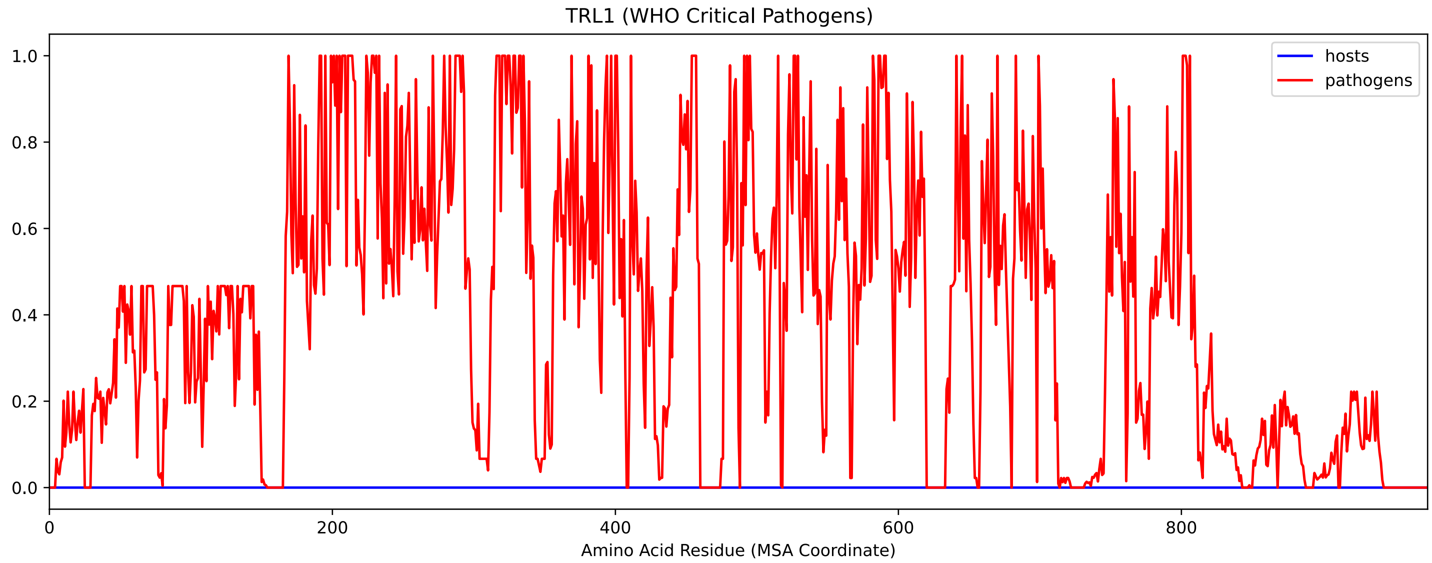

Figure S178: Sneath Similarity of Trl1 for WHO Critical Pathogens, cf. Figure S176

S2.16.2 Top 10 Agricultural Fungal Pathogens

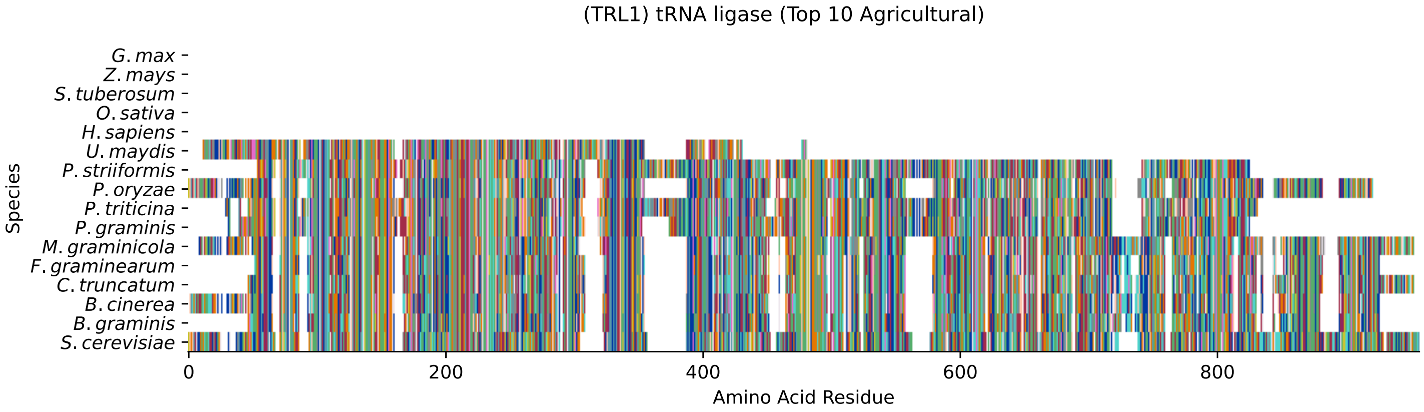

Figure S179: Multiple sequence alignment of yeast Trl1 (Top 10 Agricultural Fungal Pathogens). Cf. Figure S180 for alignment quality, and Figure S181 for Sneath similarity. Cf. Table S37 for protein names, and pairwise alignment metrics with yeast Trl1.

Trl1 MSA Quality

	B. g.	B. c.	C. t.	F. g.	M. g.	P. g.	P. t.	P. o.	P. s.	U. m.	S. c.
B. graminis	1508.43	880.167	793.112	783.867	737.643	303.138	283.878	687.567	291.197	231.491	455.292
B. cinerea	864.759	1602.42	901.353	900.197	845.884	329.717	306.99	802.357	320.472	235.728	494.197
C. truncatum	793.882	918.302	1580.46	1061.98	850.892	333.569	335.109	894.419	305.449	246.514	521.161
F. graminearum	774.237	910.598	1062.75	1498.8	802.742	327.791	317.775	865.914	294.278	251.906	493.041
M. graminicola	718.768	845.884	841.262	792.727	1679.84	336.65	330.102	751.125	309.301	251.521	480.715
P. graminis	294.664	329.717	330.102	325.479	336.65	1498.03	983.786	346.665	1139.79	223.016	290.041
P. triticina	283.878	315.849	344.739	323.553	340.502	1003.05	1494.56	338.191	991.104	236.498	282.722
P. oryzae	669.848	802.357	889.026	862.448	751.125	346.665	327.405	1595.48	319.701	252.677	489.574
P. striiformis	283.108	320.472	302.368	291.967	309.301	1139.79	962.6	319.701	1501.11	231.876	277.33
U. maydis	230.72	235.728	246.128	251.521	251.521	223.016	236.113	252.677	231.876	789.26	223.402
S. cerevisiae	437.958	494.197	514.612	476.863	480.715	290.041	276.174	489.574	277.33	223.402	1710.66

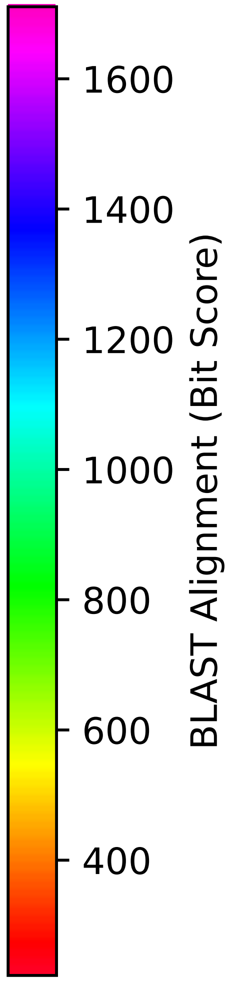

Figure S180: Multiple sequence alignment quality of Trl1 (Top 10 Agricultural Fungal Pathogens). Cf. Figure S179

Species	Hit Protein	Hit Length (a.a.)	evalue	align_len	bit_score	identity	positive	score	gaps	% identity	% positive
G.max	-	-	-	-	-	-	-	-	-	-	-
Z.mays	-	-	-	-	-	-	-	-	-	-	-
S.tuberosum	-	-	-	-	-	-	-	-	-	-	-
O.sativa	-	-	-	-	-	-	-	-	-	-	-
H.sapiens	-	-	-	-	-	-	-	-	-	-	-
U.maydis	XP_011386892.1 tRNA ligase Us- tilago maydis 521	380	6e-62	380	224.942	140	192	572	45	16.9	23.2
P.striiformis	XP_047800696.1 hypothetical p- rotein Pst134EA 024359 Puccini- a striiformis f. sp. tritici	736	2.6e-80	736	277.33	225	340	708	98	27.2	41.1
P.oryzae	mRNA M BR32 EuGene 00050301-p1 — transcript=mRNA M BR32 EuGe- ne 00050301 — gene=M BR32 EuGe- ne 00050301 — organism=Pyricul- aria oryzae BR32 — gene produc- t=unspecified product — transc- ript product=unspecified produ- ct — location=BR32 scaffold000- 04:1761131-1763962(-) — protei- n length=943 — sequence SO=sup- ercontig — SO=protein coding g- ene — is pseudo=false	815	2.2e-160	815	490.345	304	450	1261	71	36.8	54.4
P.triticina	XP_053026401.1 uncharacterize- d protein PtA15 13A245 Puccini- a triticina	760	1.6e-80	760	276.559	221	336	706	108	26.7	40.6
P.graminis	XP_003326541.2 hypothetical p- rotein PGTG 07519 Puccinia gra- minis f. sp. tritici CRL 75-36- -700-3	736	4.8e-85	736	290.041	228	362	741	85	27.6	43.8
M.graminicola	ZTRI 1.1976.mRNA-p1 — transcri- pt=ZTRI 1.1976.mRNA — gene=ZTR- I 1.1976 — organism=Zymoseptor- ia tritici IPO323 — gene produ- ct=similar to trna ligase — tr- anscript product=similar to tr- na ligase — location=Ztri chr 1:5571847-5574309(-) — protein length=820 — sequence SO=chro- mosome — SO=protein coding gen- e — is pseudo=false	848	4.6e-158	848	480.33	311	457	1235	81	37.6	55.3
F.graminearum	XP_011318754.1 hypothetical p- rotein FGSG 11594 Fusarium gra- minearum PH-1	773	3.9e-157	773	477.248	293	429	1227	75	35.4	51.9
C.truncatum	XP_036575045.1 tRNA ligase Co- lletotrichum truncatum	807	4.3e-171	807	515.383	317	448	1326	70	38.3	54.2
B.cinerea	XP_001558448.2 Bctrl1 Botryti- s cinerea B05.10	825	3.9e-163	825	494.967	311	461	1273	88	37.6	55.7
B.graminis	VDB94992.1 — transcript=BGT962- 24V316 LOCUS8047 t1 — gene=BGT- 96224V316 LOCUS8047 — organism- =Blumeria graminis f. sp. trit- ici 96224 — gene product=unspe- cified product — transcript pr- oduct=unspecified product — lo- cation=LR026993:6965370-696788- 2(+) — protein length=803 — se- quence SO=chromosome — SO=prot- ein coding gene — is pseudo=fa- lse	780	3.9e-142	780	437.958	294	427	1125	75	35.6	51.6

Table S37: Pairwise alignment info from yeast Trl1 (DEG20010555), cf. Figure S179.

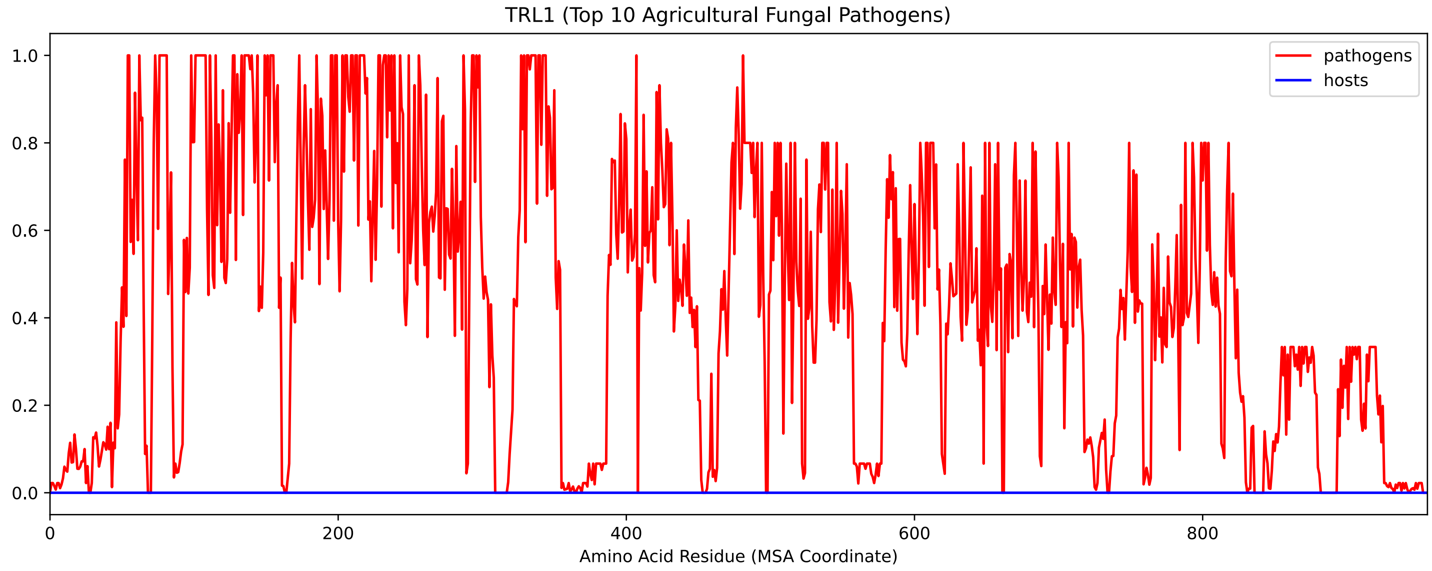

Figure S181: Sneath Similarity of Trl1 for Top 10 Agricultural Fungal Pathogens, cf. Figure S179

S2.16.3 NR

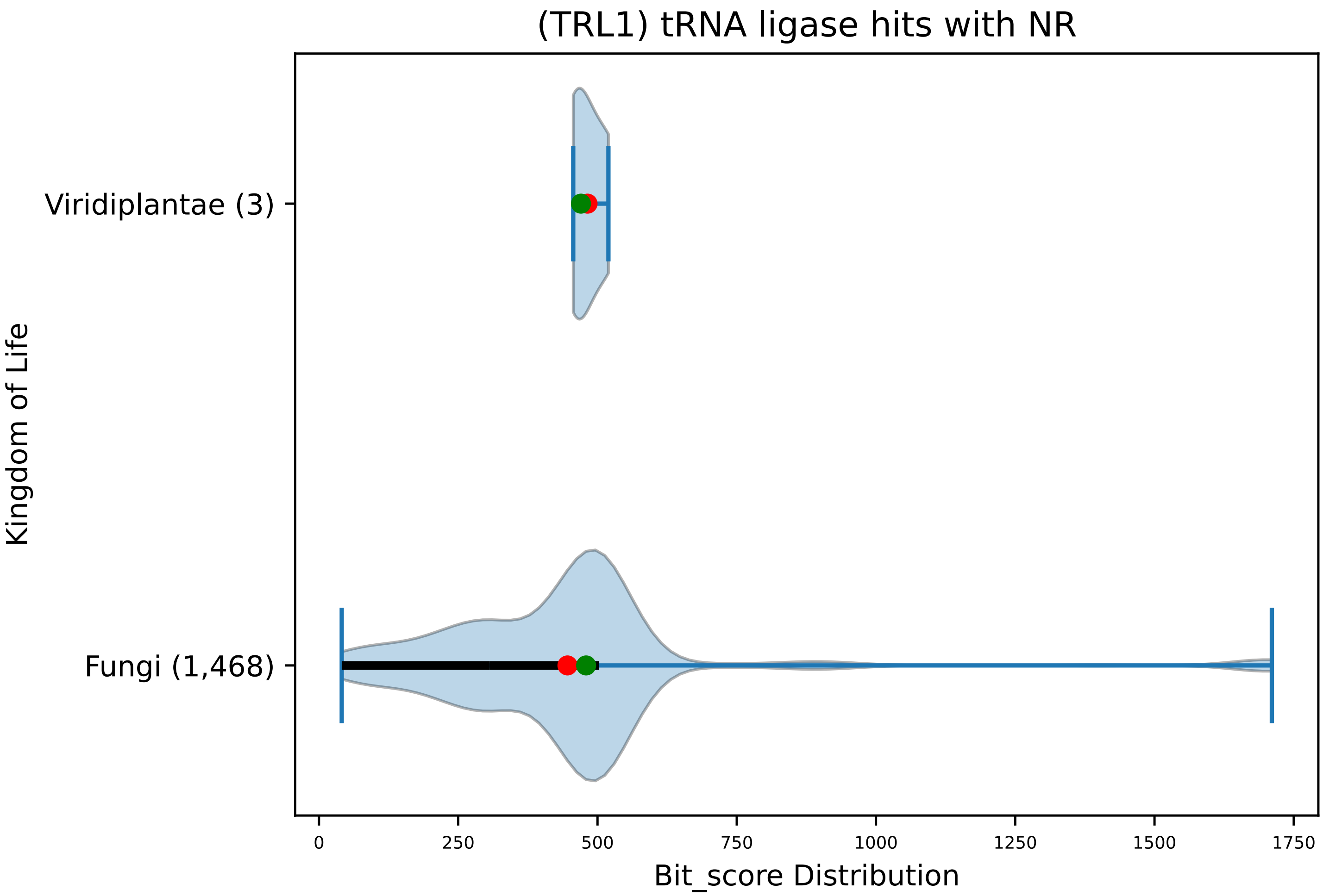

Figure S182: Non-redundant (NR) protein hits for DEG20010555/Trl1, with expectation value of no more than 0.1. Green points are medians, and red points are arithmetic means.

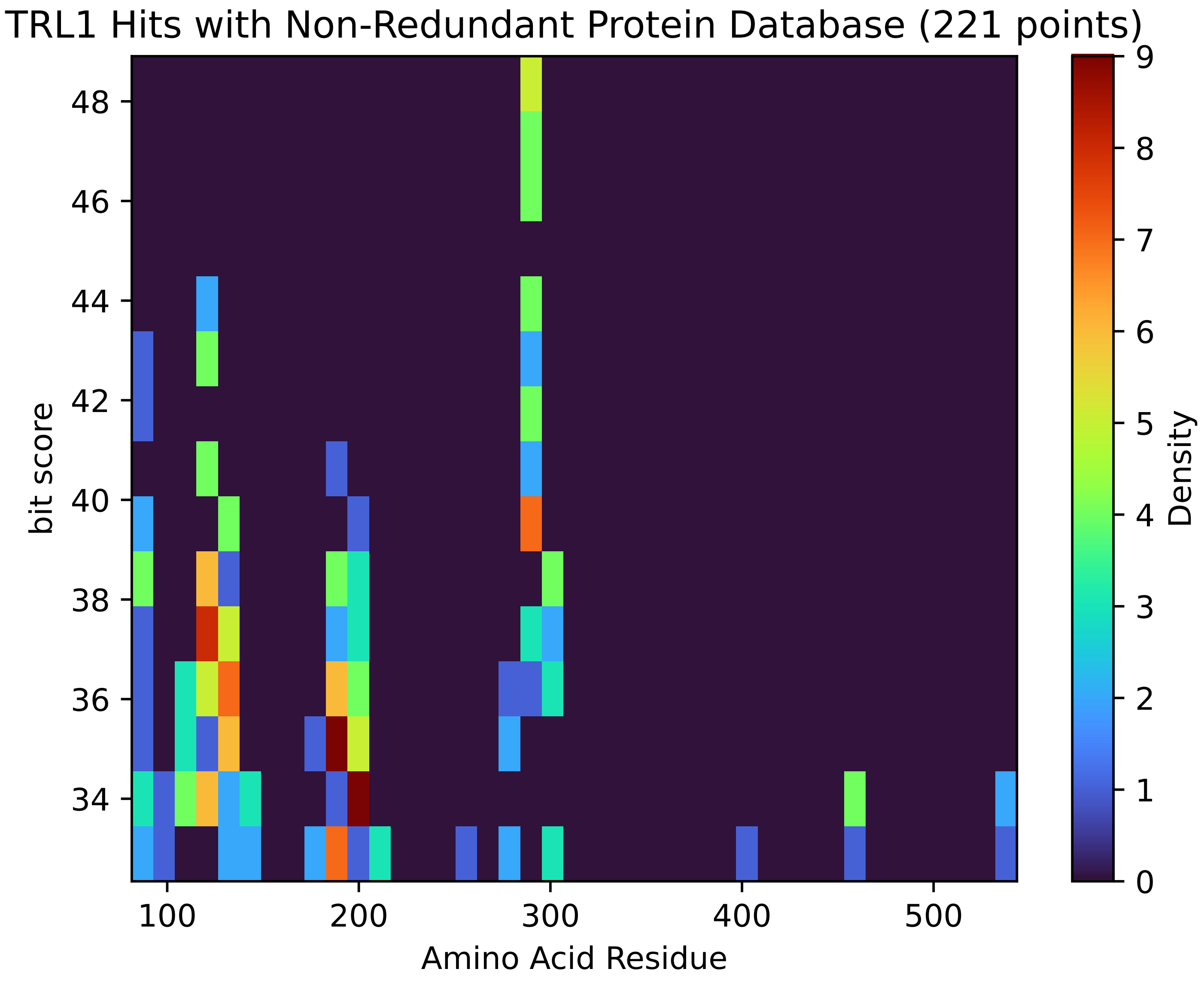

Figure S183: Non-redundant (NR) protein hits for Trl1 in the kingdom Viridiplantae.

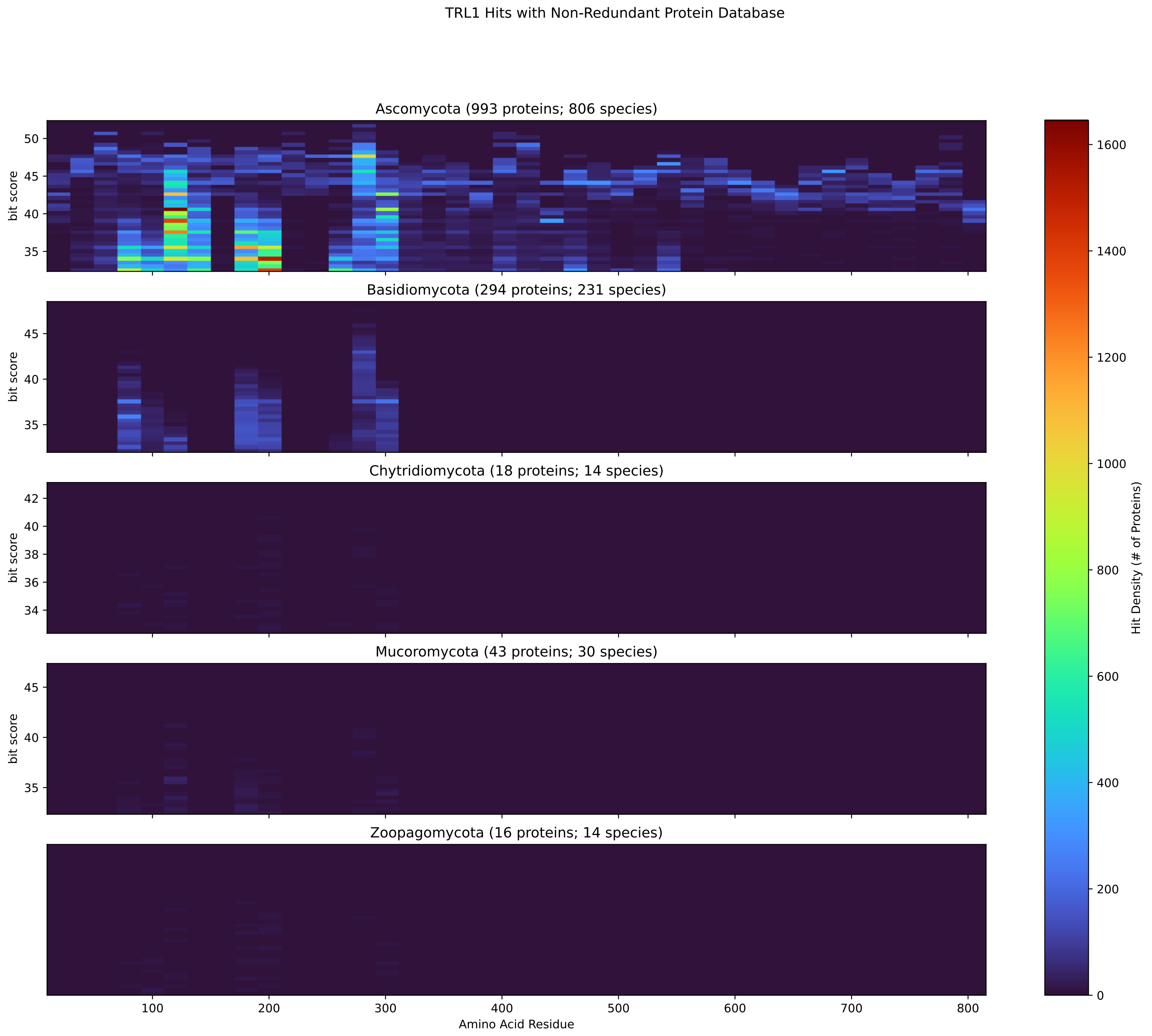

Figure S184: Non-redundant (NR) protein hits for Trl1 in the kingdom Fungi.

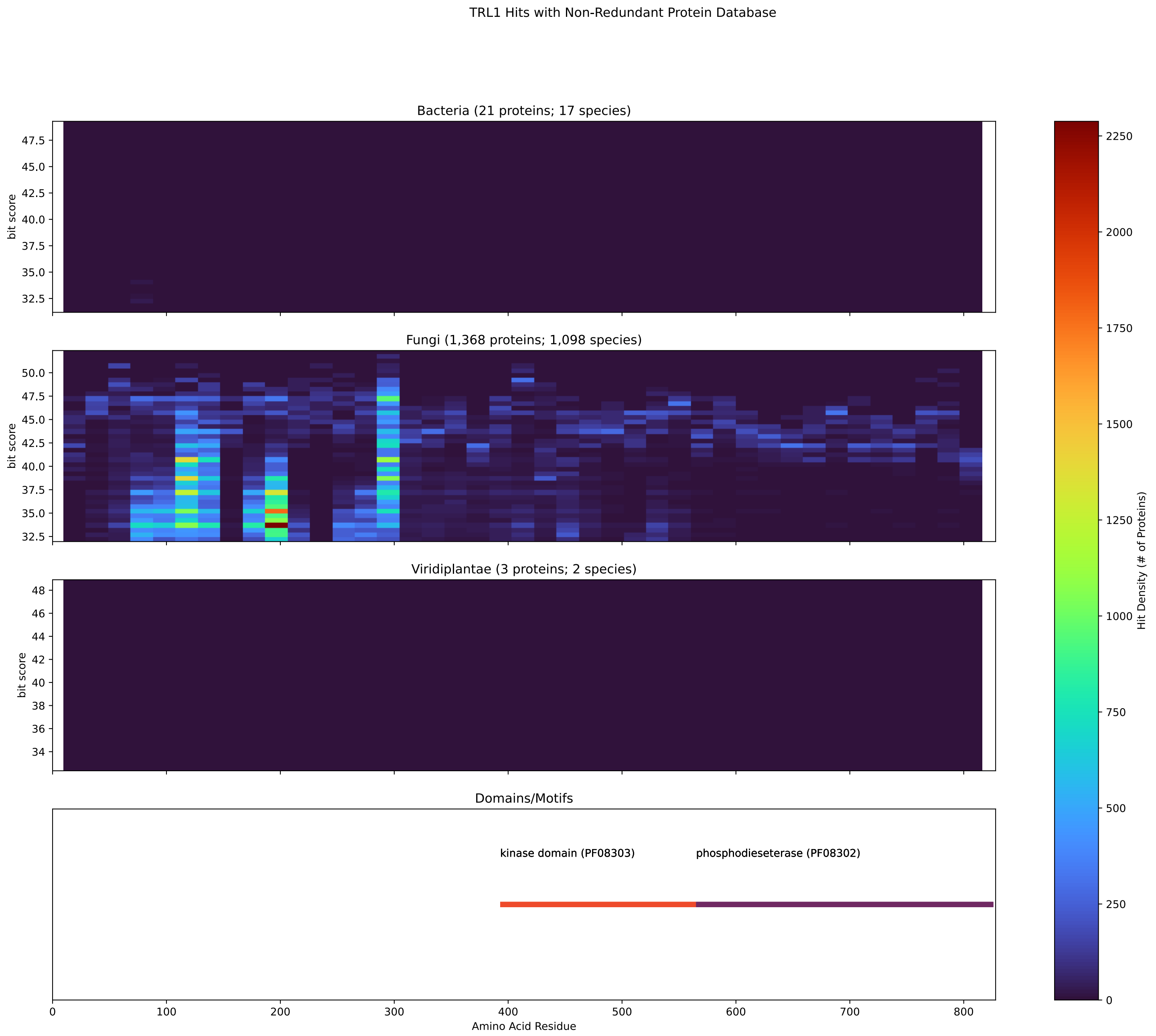

Figure S185: Non-redundant (NR) protein hits for DEG20010555/Trl1 at 20 amino acid length queries.

S2.17 Yef3

S2.17.1 WHO Critical Pathogens

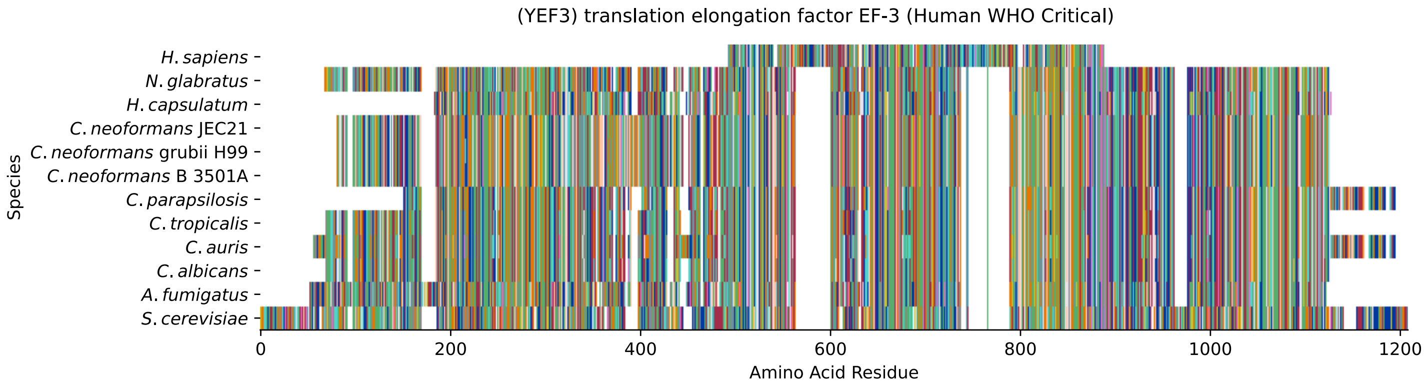

Figure S186: Multiple sequence alignment of yeast Yef3 (WHO Critical Pathogens). Cf. Figure S187 for alignment quality, and Figure S188 for Sneath similarity. Cf. Table S38 for protein names, and pairwise alignment metrics with yeast Yef3.

Yef3 MSA Quality

	A. f.	*C. a.*	*C. a.*	*C. p.*	*C. t.*	*C. n.*	*C. n.*	*C. n.*	*H. c.*	*N. g.*	*S. c.*
A. fumigatus	1957.96	922.924	951.429	900.582	918.687	801.586	801.201	801.586	1395.18	955.666	631.328
C. albicans	909.057	1857.03	1520.75	1466.44	1715.66	794.653	795.423	794.653	949.503	1277.69	597.43
C. auris	939.488	1520.75	2024.59	1506.12	1501.88	805.823	801.971	805.823	962.6	1270.37	616.305
C. parapsilosis	897.501	1484.93	1521.14	1818.13	1468.75	806.209	801.971	806.209	962.214	1221.07	604.749
C. tropicalis	908.672	1726.07	1511.89	1472.6	1847.4	803.512	803.512	803.512	950.658	1290.4	600.897
C. neoformans B 3501A	787.334	794.653	805.823	788.104	793.882	1877.83	1866.66	1877.83	803.897	797.349	658.292
C. neoformans grubii H99	787.719	795.423	801.971	784.252	793.497	1866.66	1875.91	1866.66	804.668	793.497	660.603
C. neoformans JEC21	787.334	794.653	805.823	788.104	793.882	1877.83	1866.66	1877.83	803.897	797.349	658.292
H. capsulatum	1383.24	949.503	962.6	952.199	946.806	803.897	804.668	803.897	1702.18	951.044	629.787
N. glabratus	956.822	1295.8	1287.32	1219.53	1305.81	807.364	804.283	807.364	964.911	1869.74	620.928
S. cerevisiae	633.254	609.757	629.402	609.372	606.29	676.011	678.707	676.011	647.121	621.698	2163.27

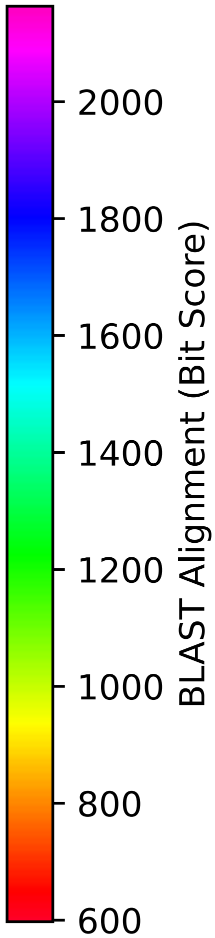

Figure S187: Multiple sequence alignment quality of Yef3 (WHO Critical Pathogens). Cf. Figure S186

Species	Hit Protein	Hit Length (a.a.)	evalue	align_len	bit_score	identity	positive	score	gaps	% identity	% positive
H.sapiens	NP_001338227.1 ATP-binding cassette sub-family F member 3 isoform 2 Homo sapiens	396	3.2e-39	396	159.073	117	172	401	98	11.2	16.5
N.glabratus	XP_445575.1 uncharacterized protein CAGL0D03674g Nakaseomyces glabratus	939	0	939	620.928	364	545	1600	60	34.9	52.2
H.capsulatum	XP_045289814.1 elongation factor 3 Histoplasma capsulatum G-186AR	851	0	851	647.892	368	511	1670	58	35.2	48.9
C.neoformans.JEC21	XP_566522.1 mRNA export factor elf1, putative Cryptococcus neoformans var. neoformans JEC-21	922	0	922	676.011	377	527	1743	38	36.1	50.5
C.neoformans.grubii.H99	XP_012046531.1 elongation factor 3 Cryptococcus neoformans var. grubii H99	922	0	922	678.322	379	527	1749	38	36.3	50.5
C.neoformans.B.3501A	XP_778089.1 hypothetical protein CNBA0920 Cryptococcus neoformans var. neoformans B-3501A	922	0	922	676.011	377	527	1743	38	36.1	50.5
C.parapsilosis	XP_036668290.1 uncharacterized protein CPAR2 701340 Candida parapsilosis	916	0	916	610.527	349	538	1573	62	33.4	51.5
C.tropicalis	XP_002546443.1 mRNA export factor elf1 Candida tropicalis MYA-3404	932	0	932	606.675	343	537	1563	55	32.9	51.4
C.auris	XP_028888995.2 hypothetical protein Candida auris	1010	0	1010	632.098	369	573	1629	61	35.3	54.9
C.albicans	XP_716466.1 Elf1p Candida albicans SC5314	944	0	944	609.372	349	541	1570	76	33.4	51.8
A.fumigatus	XP_747719.1 mRNA-nucleus export ATPase (Elf1), putative Aspergillus fumigatus Af293	966	0	966	634.024	385	553	1634	64	36.9	53.0

Table S38: Pairwise alignment info from yeast Yef3 (DEG20010729), cf. Figure S186.

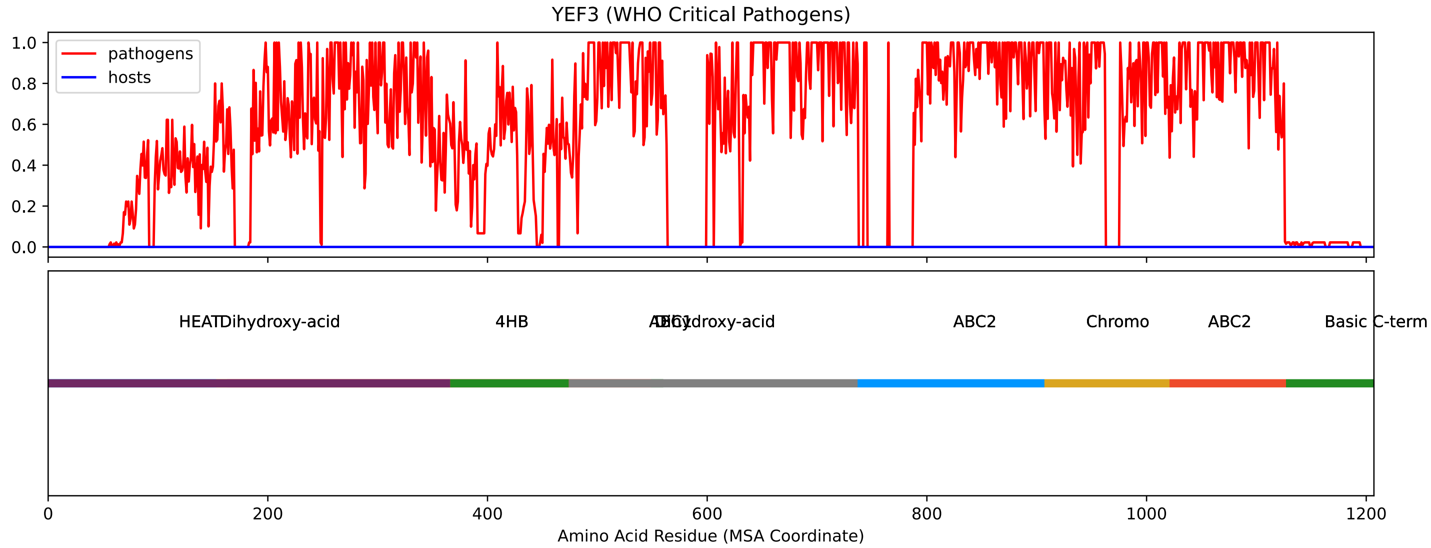

Figure S188: Sneath Similarity of Yef3 for WHO Critical Pathogens, cf. Figure S186

S2.17.2 Top 10 Agricultural Fungal Pathogens

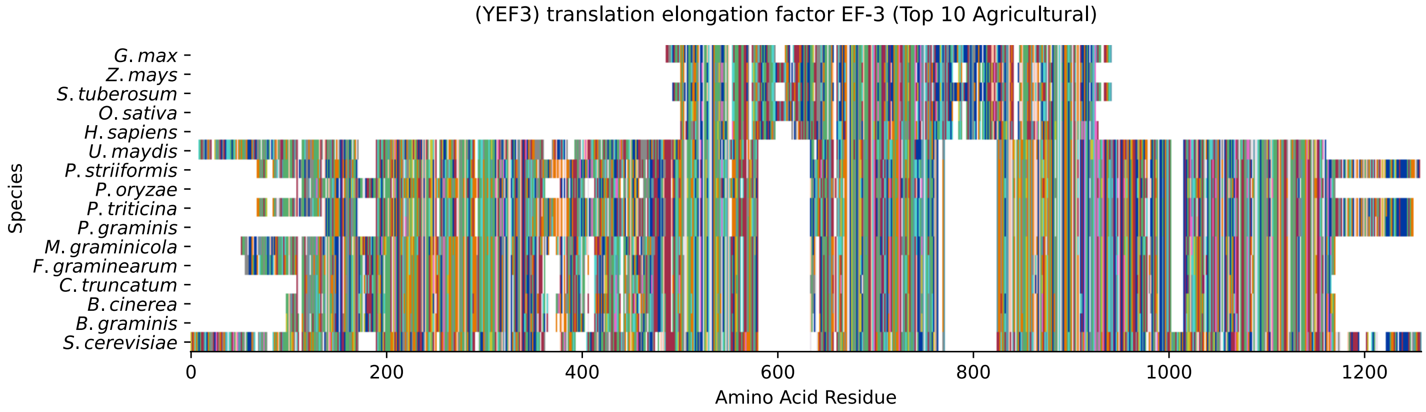

Figure S189: Multiple sequence alignment of yeast Yef3 (Top 10 Agricultural Fungal Pathogens). Cf. Figure S190 for alignment quality, and Figure S191 for Sneath similarity. Cf. Table S39 for protein names, and pairwise alignment metrics with yeast Yef3.

Yef3 MSA Quality

	B. g.	*B. c.*	*C. t.*	*F. g.*	*M. g.*	*P. g.*	*P. t.*	*P. o.*	*P. s.*	*U. m.*	*S. c.*
B. graminis	1890.16	1580.84	1466.06	1485.7	1377.07	778.859	780.015	1443.33	778.859	821.617	629.017
B. cinerea	1580.84	1894.78	1455.66	1471.83	1380.16	784.637	785.408	1457.58	780.015	845.114	652.514
C. truncatum	1466.06	1455.66	1849.33	1588.93	1352.42	770.77	776.548	1536.55	764.992	823.157	651.358
F. graminearum	1485.7	1471.83	1588.93	1956.8	1406.35	793.112	788.875	1566.59	781.941	838.565	643.269
M. graminicola	1377.07	1380.16	1352.42	1406.35	1993.01	780.4	782.711	1339.71	776.548	844.343	608.601
P. graminis	778.859	784.637	770.77	793.112	780.4	1975.67	1924.06	781.941	1919.05	951.429	606.29
P. triticina	780.015	785.408	776.548	788.875	782.711	1924.06	2100.86	784.637	2032.3	976.082	607.831
P. oryzae	1443.33	1457.58	1536.55	1566.59	1339.71	781.941	784.637	1854.72	776.548	806.979	652.899
P. striiformis	779.63	785.793	771.926	787.719	782.326	1919.05	2032.3	782.711	2116.27	990.334	610.142
U. maydis	838.565	861.677	844.729	864.759	871.307	975.696	1002.28	829.706	1004.59	2025.75	681.789
S. cerevisiae	647.121	668.307	671.774	664.455	626.32	645.966	642.884	669.463	644.04	689.108	2163.27

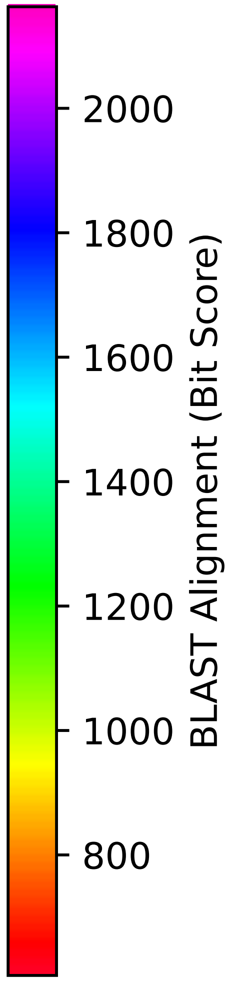

Figure S190: Multiple sequence alignment quality of Yef3 (Top 10 Agricultural Fungal Pathogens). Cf. Figure S189

Species	Hit Protein	Hit Length (a.a.)	evalue	align_len	bit_score	identity	positive	score	gaps	% identity	% positive
G.max	XP_003542630.1 ABC transporter F family member 4 Glycine max	439	3.6e-47	439	182.185	124	195	461	117	11.9	18.7
Z.mays	NP_001349290.1 ABC transporter F family member 3-like Zea mays	408	6.8e-42	408	165.622	120	174	418	114	11.5	16.7
S.tuberosum	XP_006362455.1 PREDICTED: ABC transporter F family member 4--like Solanum tuberosum	431	1.8e-48	431	185.267	127	192	469	114	12.2	18.4
O.sativa	XP_015626086.1 ABC transporter F family member 3 Oryza sativa Japonica Group	408	2e-42	408	167.162	120	174	422	114	11.5	16.7
H.sapiens	NP_001338227.1 ATP-binding cassette sub-family F member 3 isoform 2 Homo sapiens	396	3.2e-39	396	159.073	117	172	401	98	11.2	16.5
U.maydis	XP_011390724.1 putative mrna export factor elf1 Ustilago maydis 521	1001	0	1001	689.878	406	580	1779	59	38.9	55.6
P.striiformis	XP_047803383.1 hypothetical protein Pst134EA 019464 Puccinia striiformis f. sp. tritici	1043	0	1043	644.425	374	581	1661	83	35.8	55.7
P.oryzae	mRNA M BR32 EuGene 00061071-p1 — transcript=mRNA M BR32 EuGene 00061071 — gene=M BR32 EuGene 00061071 — organism=Pyricularia oryzae BR32 — gene product=unspecified product — transcript product=unspecified product — location=BR32 scaffold00005:2291252-2294682(-) — protein length=1118 — sequence SO=supercontig — SO=protein coding gene — is pseudo=false	922	0	922	669.848	381	548	1727	64	36.5	52.5
P.triticina	XP_053024635.1 uncharacterized protein PtA15 10A503 Puccinia triticina	1040	0	1040	643.269	380	578	1658	89	36.4	55.4
P.graminis	XP_003319416.1 hypothetical protein PGTG 01590 Puccinia graminis f. sp. tritici CRL 75-36--700-3	977	0	977	645.966	369	559	1665	83	35.3	53.5
M.graminicola	ZTRI 5.109.mRNA-p1 — transcript=ZTRI 5.109.mRNA — gene=ZTRI 5.109 — organism=Zymoseptoria tritici IPO323 — gene product=similar to mRNA-nucleus export ATPase (Elf1)/ABC Transporter — transcript product=similar to mRNA-nucleus export ATPase (Elf1)/ABC Transporter — location=Ztri chr 5:449343-452784(+-) — protein length=1128 — sequence SO=chromosome — SO=protein coding gene — is pseudo=false	984	0	984	625.935	376	547	1613	76	36.0	52.4
F.graminearum	XP_011320226.1 prion formation protein 1 Fusarium graminearum PH-1	1001	0	1001	664.07	392	573	1712	90	37.5	54.9
C.truncatum	XP_036575201.1 ABC transporter Colletotrichum truncatum	916	0	916	672.544	383	545	1734	60	36.7	52.2
B.cinerea	XP_001545837.2 Bcnew1 Botrytis cinerea B05.10	935	0	935	667.537	388	550	1721	63	37.2	52.7
B.graminis	VDB89801.1 — transcript=BGT96224V316 LOCUS5359 t1 — gene=BGT96224V316 LOCUS5359 — organism=Blumeria graminis f. sp. tritici 96224 — gene product=unspecified product — transcript product=unspecified product — location=LR026990:14052689-14056061(+) — protein length=1104 — sequence SO=chromosome — SO=protein coding gene — is pseudo=false	935	0	935	647.121	385	545	1668	66	36.9	52.2

Table S39: Pairwise alignment info from yeast Yef3 (DEG20010729), cf. Figure S189.

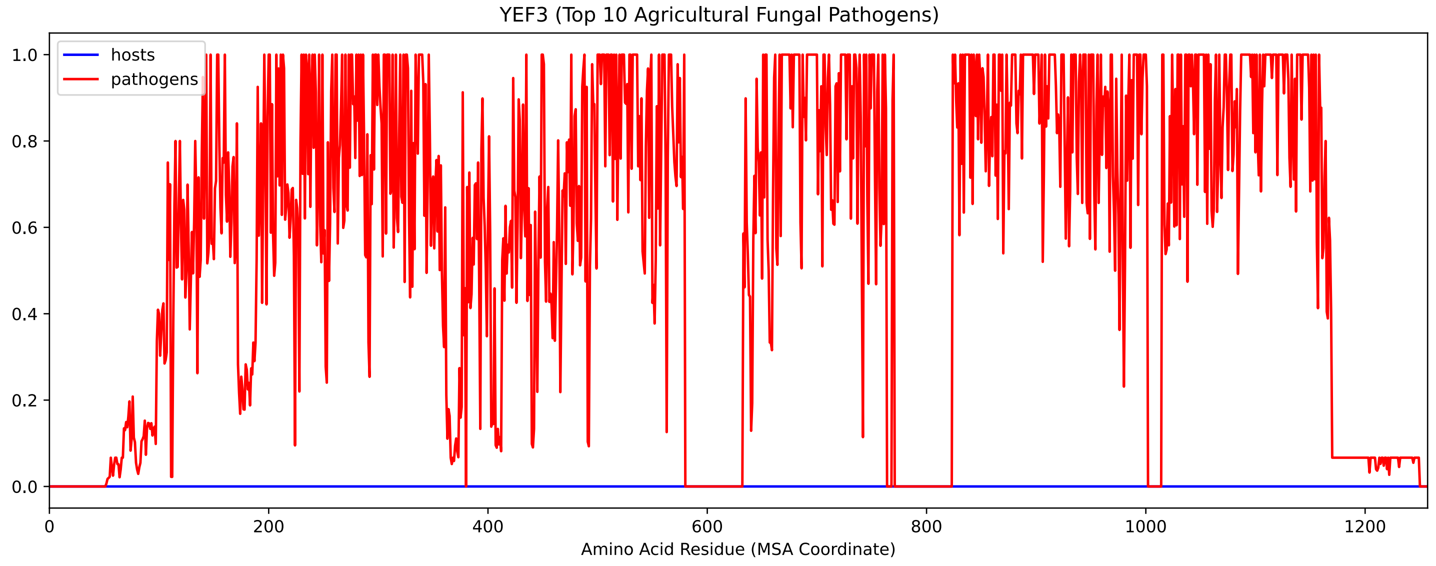

Figure S191: Sneath Similarity of Yef3 for Top 10 Agricultural Fungal Pathogens, cf. Figure S189

S2.17.3 NR

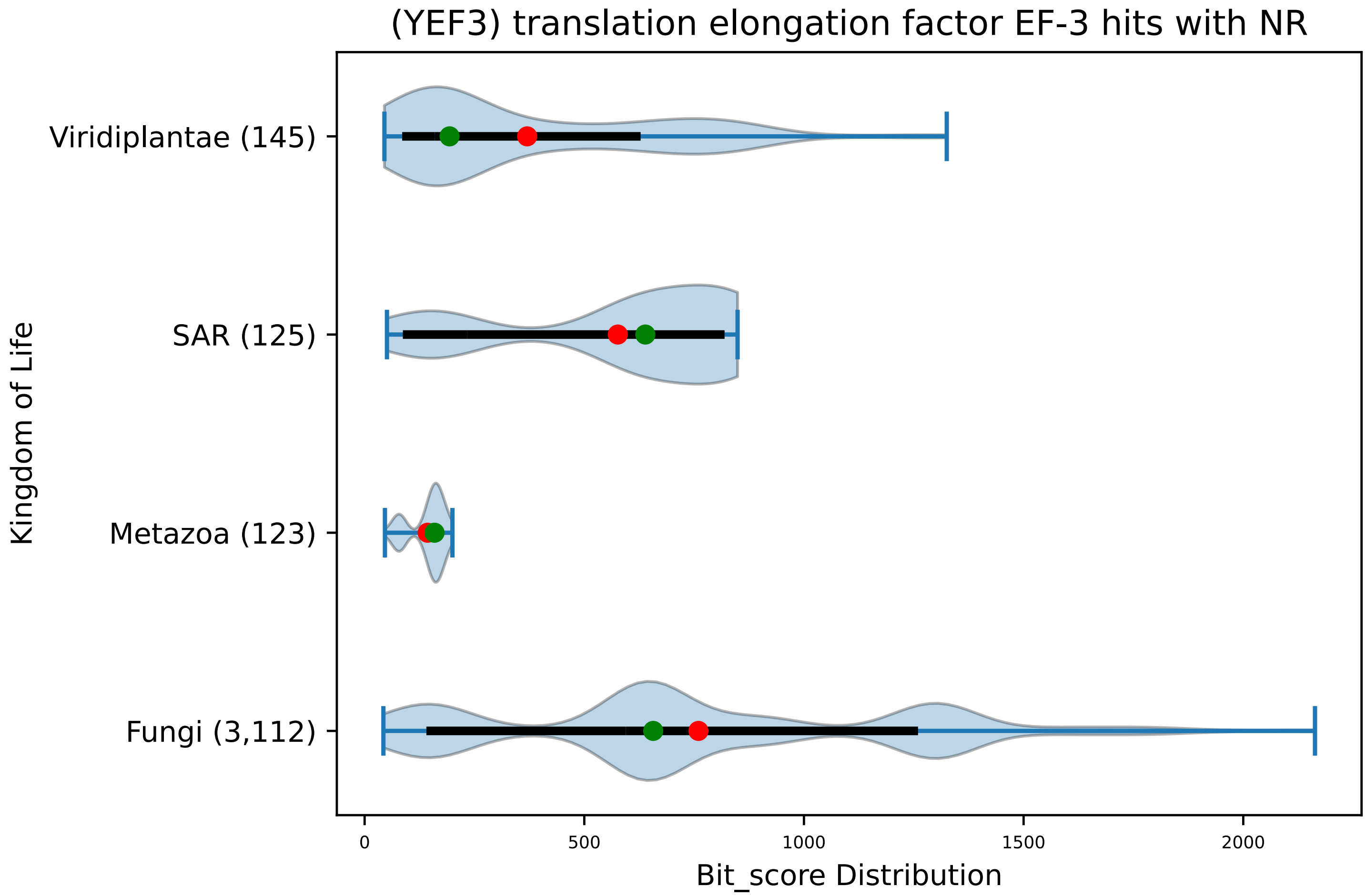

Figure S192: Non-redundant (NR) protein hits for DEG20010729/Yef3, with expectation value of no more than 0.1. Green points are medians, and red points are arithmetic means.

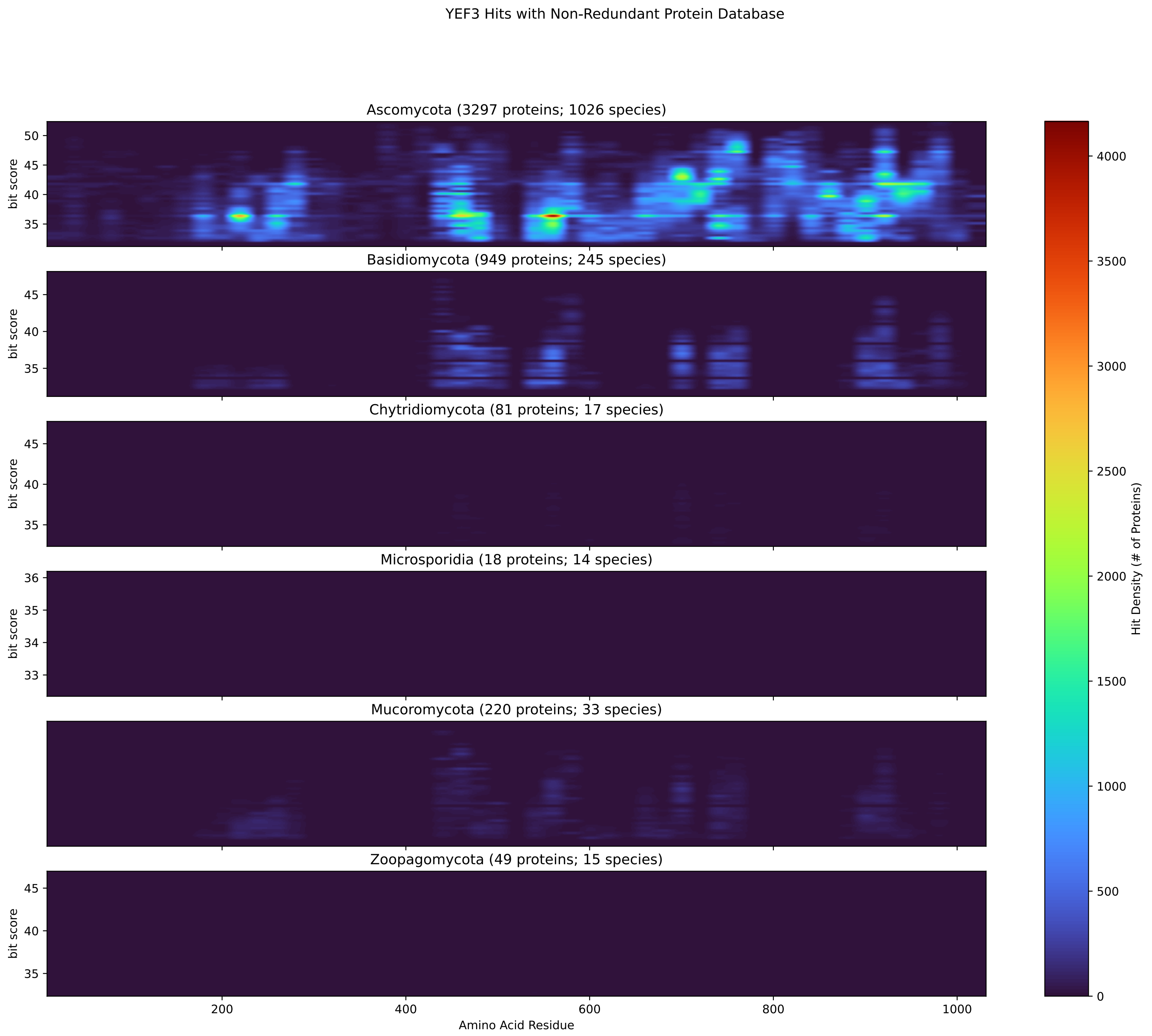

Figure S193: Non-redundant (NR) protein hits for Yef3 in the kingdom Fungi.

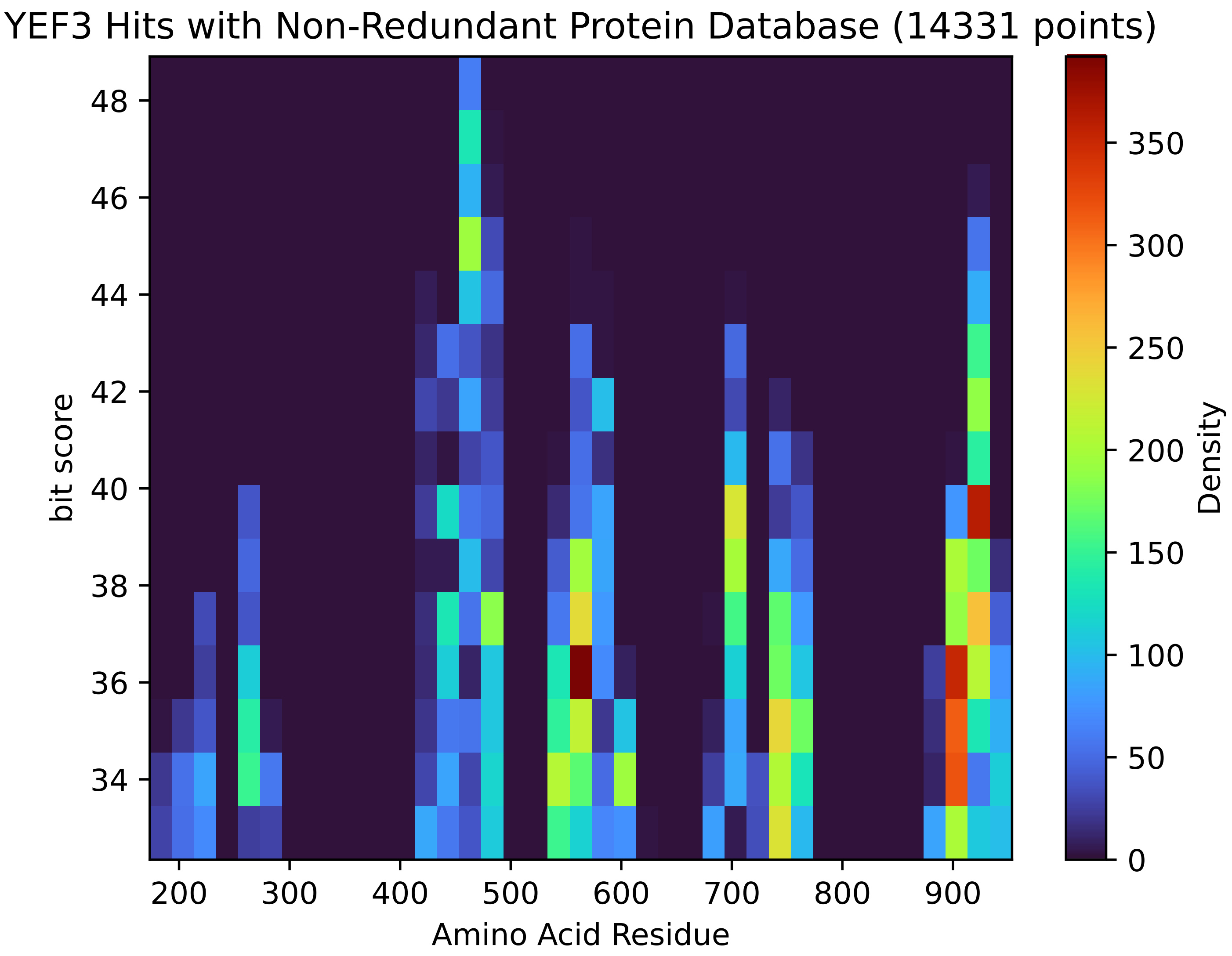

Figure S194: Non-redundant (NR) protein hits for Yef3 in the kingdom SAR.

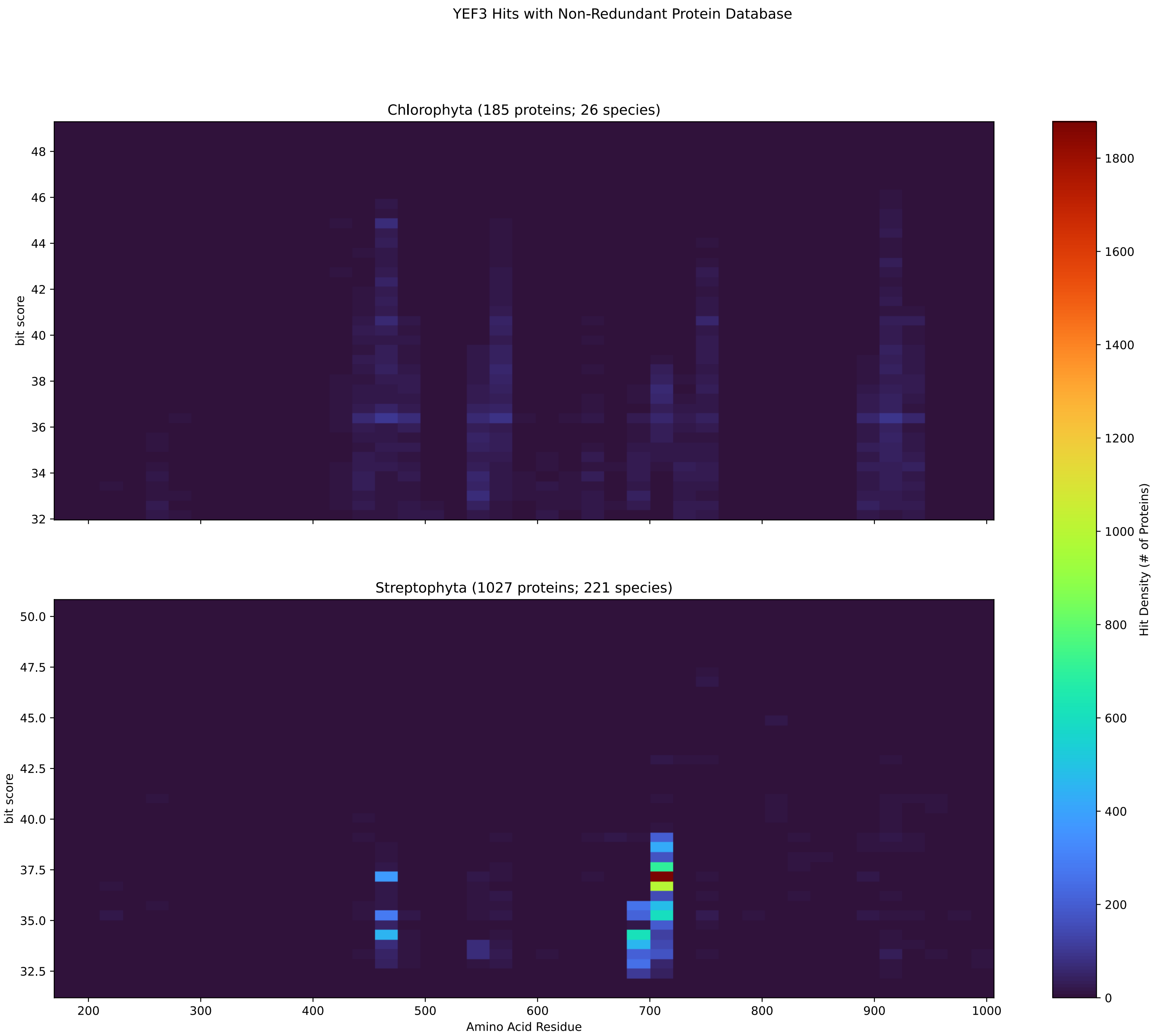

Figure S195: Non-redundant (NR) protein hits for Yef3 in the kingdom Viridiplantae.

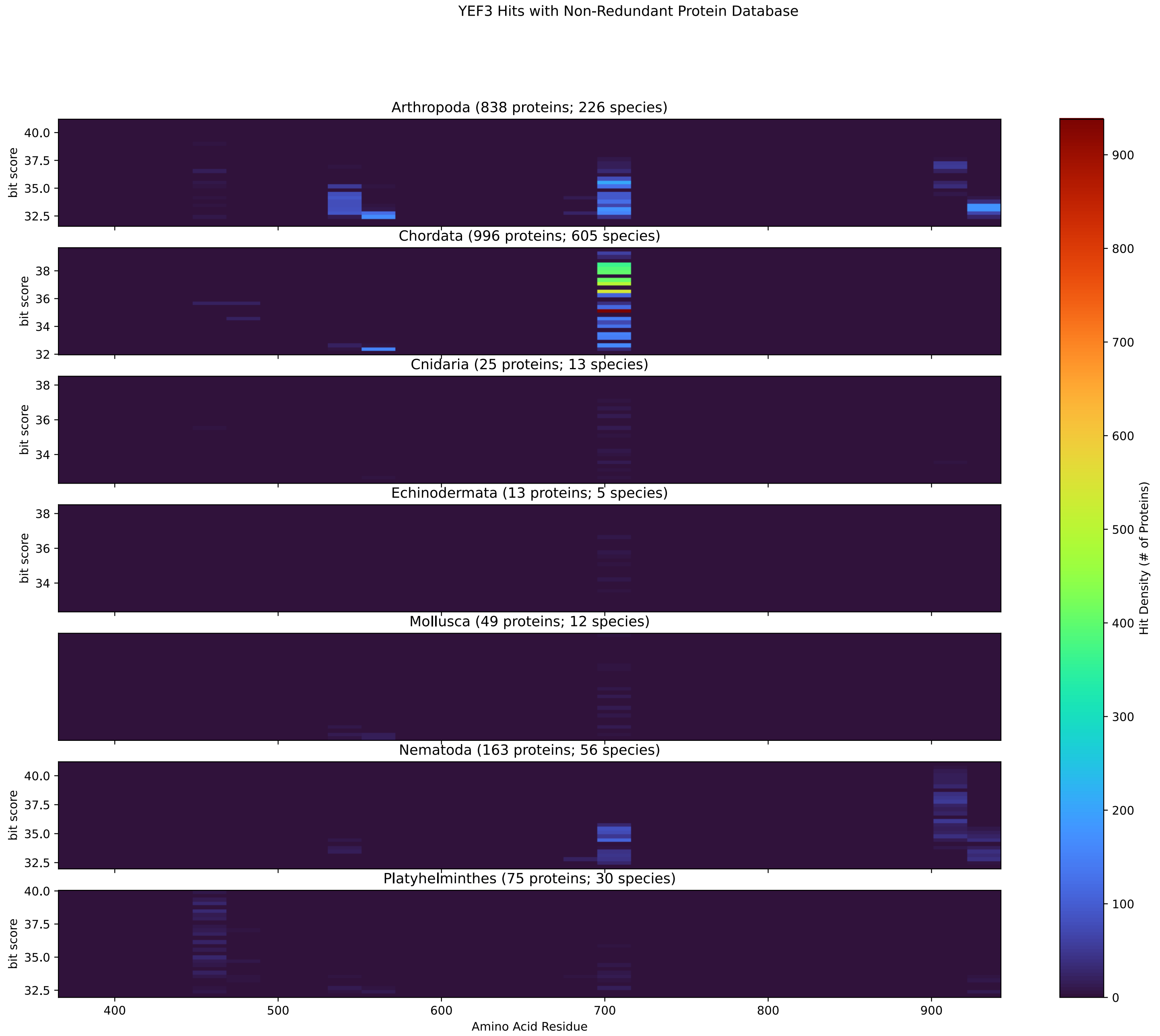

Figure S196: Non-redundant (NR) protein hits for Yef3 in the kingdom Metazoa.

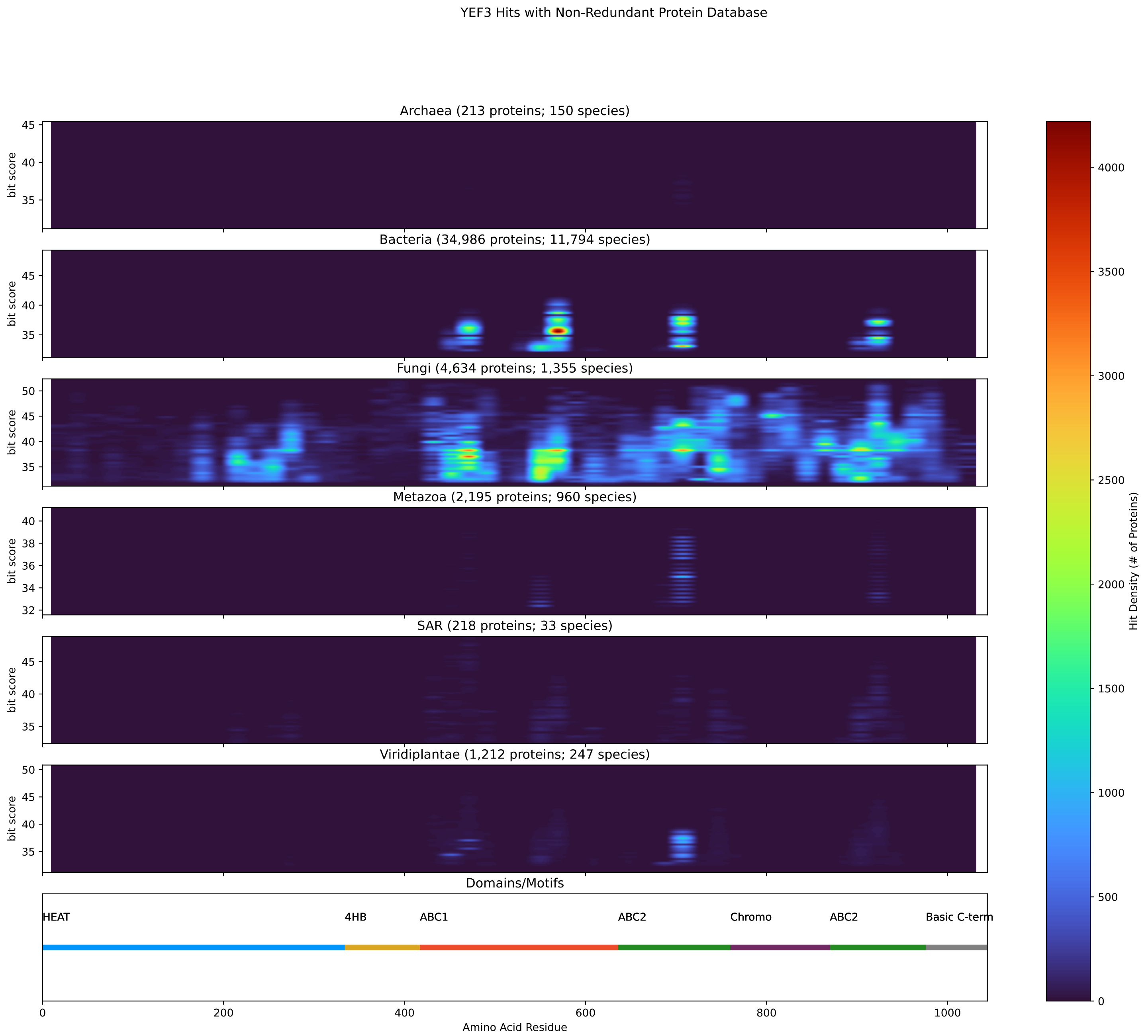

Figure S197: Non-redundant (NR) protein hits for DEG20010729/Yef3 at 20 amino acid length queries.

S3 Previously identified protein targets

S3.1 Erg11

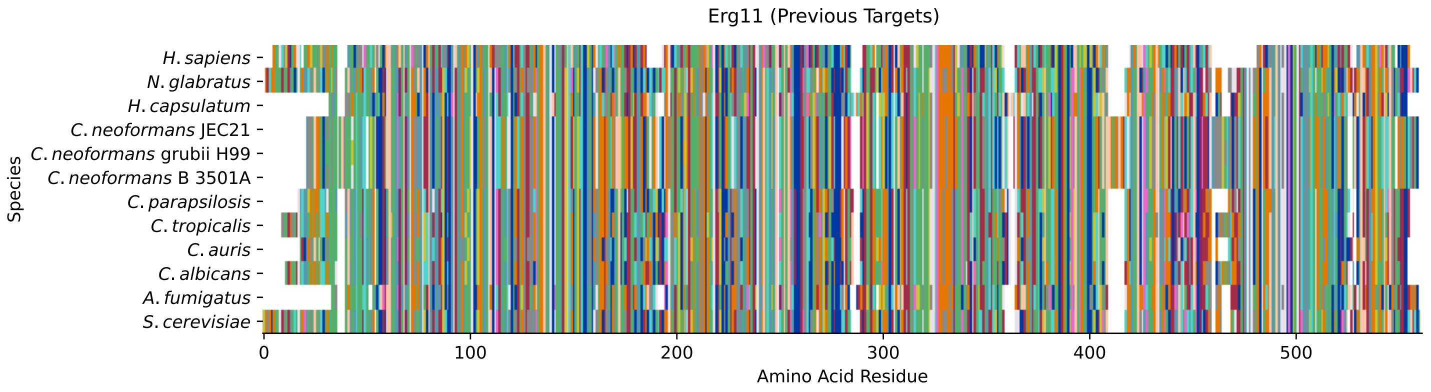

Figure S198: MSA for Erg11; cf. Table S40 for gene names and exact alignment values.

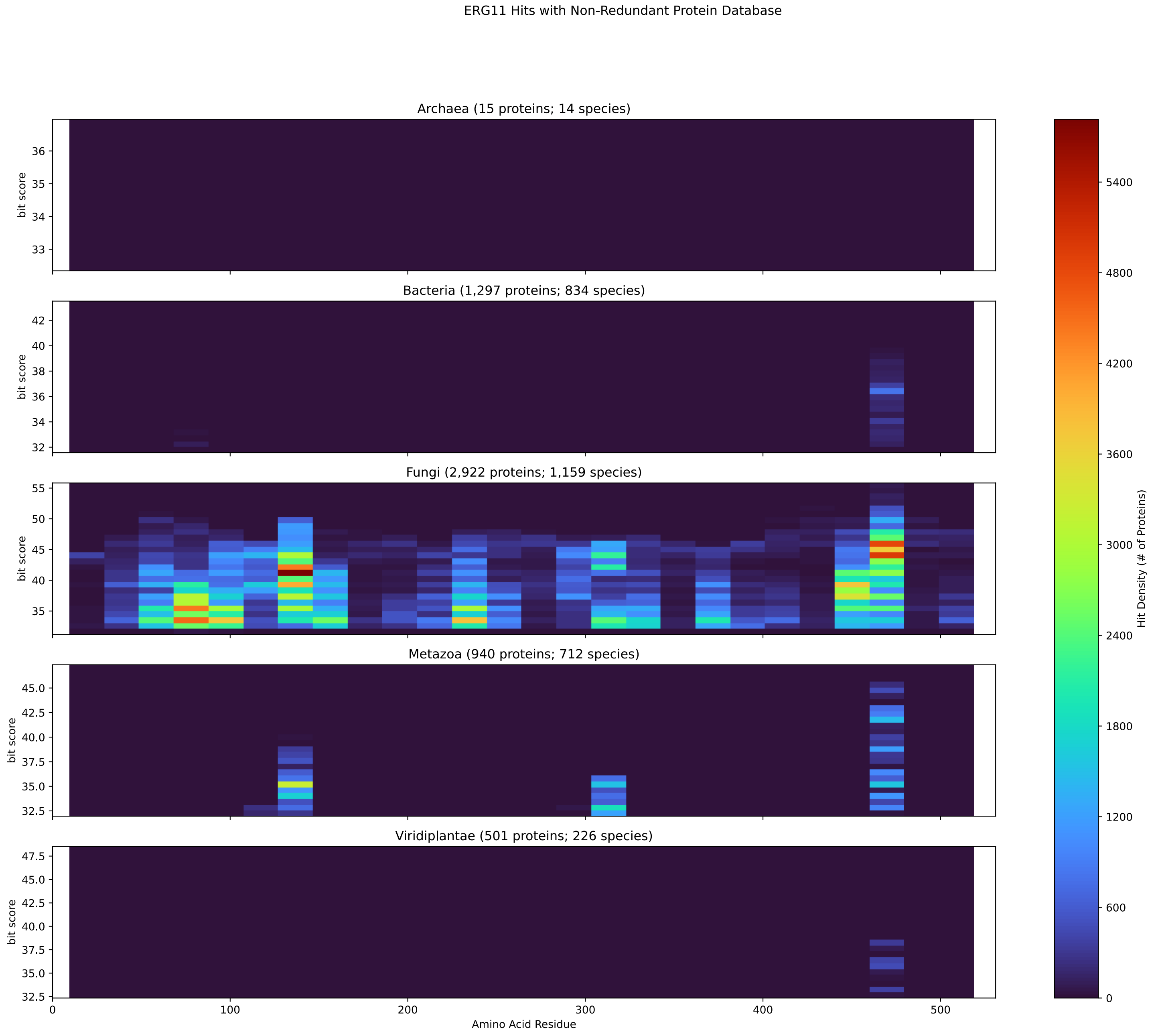

Figure S199: Histogram of Erg11 hits against NR in 20-aa windows

Species	Hit Protein	Hit Length (a.a.)	evalue	align_len	bit_score	identity	positive	score	gaps	% identity	% positive
H.sapiens	lanosterol 14-alpha demethylase isoform 1 precursor Homo sapiens	522	9e-104	522	323.168	191	290	827	33	36.0	54.6
N.glabratus	uncharacterized protein CAGL0E-04334g Nakaseomyces glabratus	530	0	530	938.717	440	486	2425	2	82.9	91.5
H.capsulatum	cytochrome P450 sterol 14 alpha-demethylase Histoplasma capsulatum G186AR	503	6.1e-175	503	501.901	253	339	1291	15	47.6	63.8
C.neoformans.JEC21	sterol 14-demethylase, putative Cryptococcus neoformans var. neoformans JEC21	531	2.8e-169	531	488.804	238	351	1257	29	44.8	66.1
C.neoformans.grubii.H99	cytochrome P450, family 51 (sterol 14-demethylase) Cryptococcus neoformans var. grubii H99	531	5.1e-170	531	490.73	240	351	1262	29	45.2	66.1
C.neoformans.B.3501A	hypothetical protein CNBA0300 Cryptococcus neoformans var. neoformans B-3501A	531	2.7e-169	531	488.804	238	351	1257	29	44.8	66.1
C.parapsilosis	uncharacterized protein CPAR2 303740 Candida parapsilosis	512	0	512	717.227	341	410	1850	7	64.2	77.2
C.tropicalis	cytochrome P450 51 Candida tropicalis MYA-3404	528	0	528	714.146	346	409	1842	13	65.2	77.0
C.auris	lanosterol 14-alpha demethylase Candida auris	510	0	510	742.651	343	420	1916	5	64.6	79.1
C.albicans	sterol 14-demethylase Candida albicans SC5314	521	0	521	711.064	339	411	1834	9	63.8	77.4
A.fumigatus	14-alpha sterol demethylase Cyp51B Aspergillus fumigatus Af293	498	1.6e-180	498	516.924	249	352	1330	7	46.9	66.3

Table S40: Pairwise alignment info from yeast Erg11

S3.2 Erg24

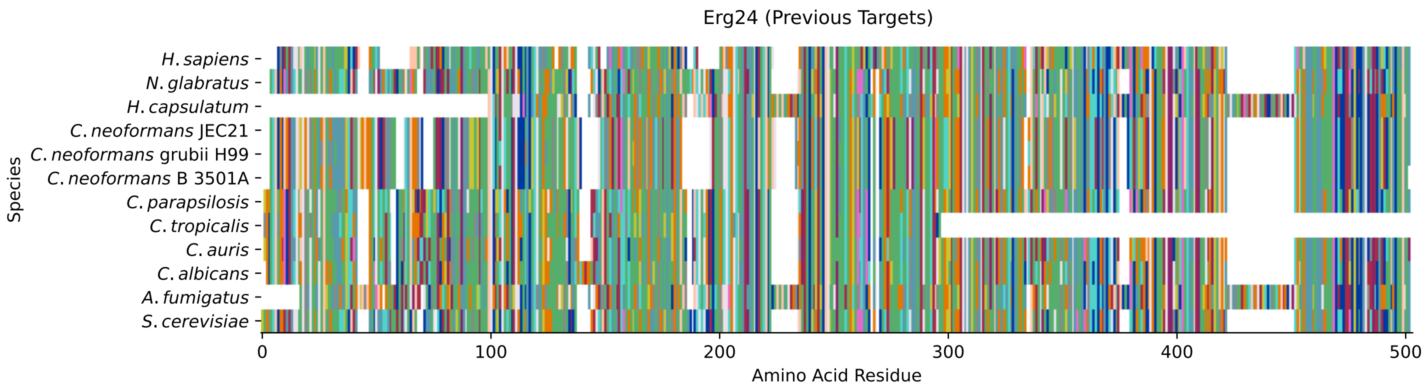

Figure S200: MSA for Erg24; cf. Table S41 for gene names and exact alignment values.

Species	Hit Protein	Hit Length (a.a.)	evalue	align_len	bit_score	identity	positive	score	gaps	% identity	% positive
H.sapiens	delta(14)-sterol reductase LBR Homo sapiens	442	1.3e-90	442	289.271	176	242	739	40	40.1	55.1
N.glabratus	uncharacterized protein CAGL0I-02970g Nakaseomyces glabratus	436	0	436	705.671	331	388	1820	2	75.4	88.4
H.capsulatum	c-14 sterol reductase Histopla-sma capsulatum G186AR	394	8.7e-88	394	275.018	146	228	702	49	33.3	51.9
C.neoformans.JEC21	C-14 sterol reductase, putativ-e Cryptococcus neoformans var. neoformans JEC21	448	6.3e-122	448	361.303	198	274	926	34	45.1	62.4
C.neoformans.grubii.H99	delta14-sterol reductase Crypt-ococcus neoformans var. grubii H99	448	6.1e-126	448	371.318	201	278	952	34	45.8	63.3
C.neoformans.B.3501A	hypothetical protein CNBA1040 Cryptococcus neoformans var. n-eoformans B-3501A	447	5e-121	447	358.992	197	273	920	34	44.9	62.2
C.parapsilosis	uncharacterized protein CPAR2 405900 Candida parapsilosis	444	5.3e-175	444	495.352	250	324	1274	10	56.9	73.8
C.tropicalis	hypothetical protein CTRG 0186-9 Candida tropicalis MYA-3404	270	6.6e-88	270	267.314	139	177	682	4	31.7	40.3
C.auris	delta(14)-sterol reductase Can-dida auris	445	1.3e-173	445	491.886	256	326	1265	12	58.3	74.3
C.albicans	delta(14)-sterol reductase Can-dida albicans SC5314	453	0	453	514.998	264	331	1325	25	60.1	75.4
A.fumigatus	c-14 sterol reductase Aspergil-lus fumigatus Af293	479	1.3e-89	479	280.411	174	256	716	67	39.6	58.3

Table S41: Pairwise alignment info from yeast Erg24

S3.3 Erg2

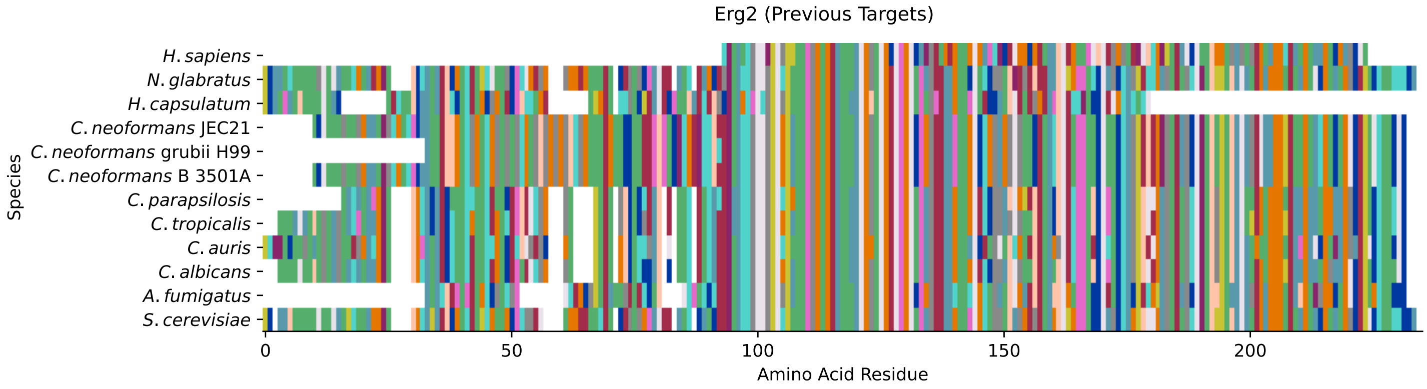

Figure S201: MSA for Erg2; cf. Table S42 for gene names and exact alignment values.

Species	Hit Protein	Hit Length (a.a.)	evalue	align_len	bit_score	identity	positive	score	gaps	% identity	% positive
H.sapiens	sigma non-opioid intracellular receptor 1 isoform 8 Homo sapiens	131	3.6e-23	131	94.7449	55	77	234	0	24.7	34.5
N.glabratus	uncharacterized protein CAGL0L-10714g Nakaseomyces glabratus	224	4.8e-127	224	356.295	170	193	913	2	76.2	86.5
H.capsulatum	C-8 sterol isomerase Histoplasma capsulatum G186AR	170	1.5e-39	170	134.42	73	104	337	10	32.7	46.6
C.neoformans.JEC21	C-8 sterol isomerase, putative Cryptococcus neoformans var. neoformans JEC21	225	9.2e-59	225	184.496	104	137	467	19	46.6	61.4
C.neoformans.grubii.H99	C-8 sterol isomerase Cryptococcus neoformans var. grubii H99	202	1.3e-58	202	184.111	99	126	466	13	44.4	56.5
C.neoformans.B.3501A	hypothetical protein CNBA8120 Cryptococcus neoformans var. neoformans B-3501A	225	8.9e-59	225	184.496	104	137	467	19	46.6	61.4
C.parapsilosis	uncharacterized protein CPAR2 109890 Candida parapsilosis	205	4e-85	205	249.98	129	144	637	4	57.8	64.6
C.tropicalis	C-8 sterol isomerase Candida tropicalis MYA-3404	218	5.7e-81	218	239.58	127	147	610	8	57.0	65.9
C.auris	C-8 sterol isomerase ERG2 Candida auris	221	6.7e-79	221	234.187	116	157	596	5	52.0	70.4
C.albicans	C-8 sterol isomerase Candida albicans SC5314	218	1.4e-83	218	246.128	128	152	627	8	57.4	68.2
A.fumigatus	C-8 sterol isomerase (Erg-1), putative Aspergillus fumigatus Af293	192	1.7e-64	192	198.749	99	128	504	6	44.4	57.4

Table S42: Pairwise alignment info from yeast Erg2

S3.4 Fks1

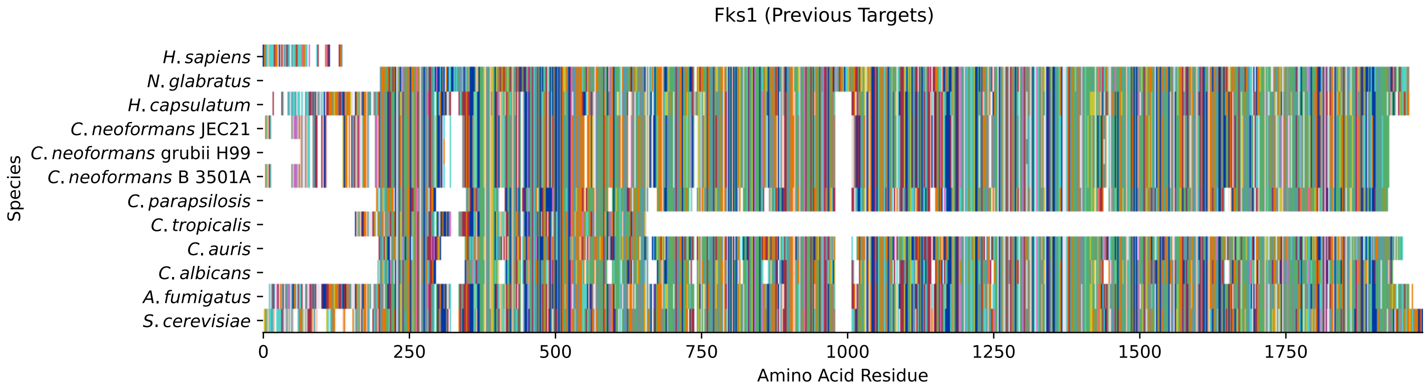

Figure S202: MSA for Fks1; cf. Table S43 for gene names and exact alignment values.

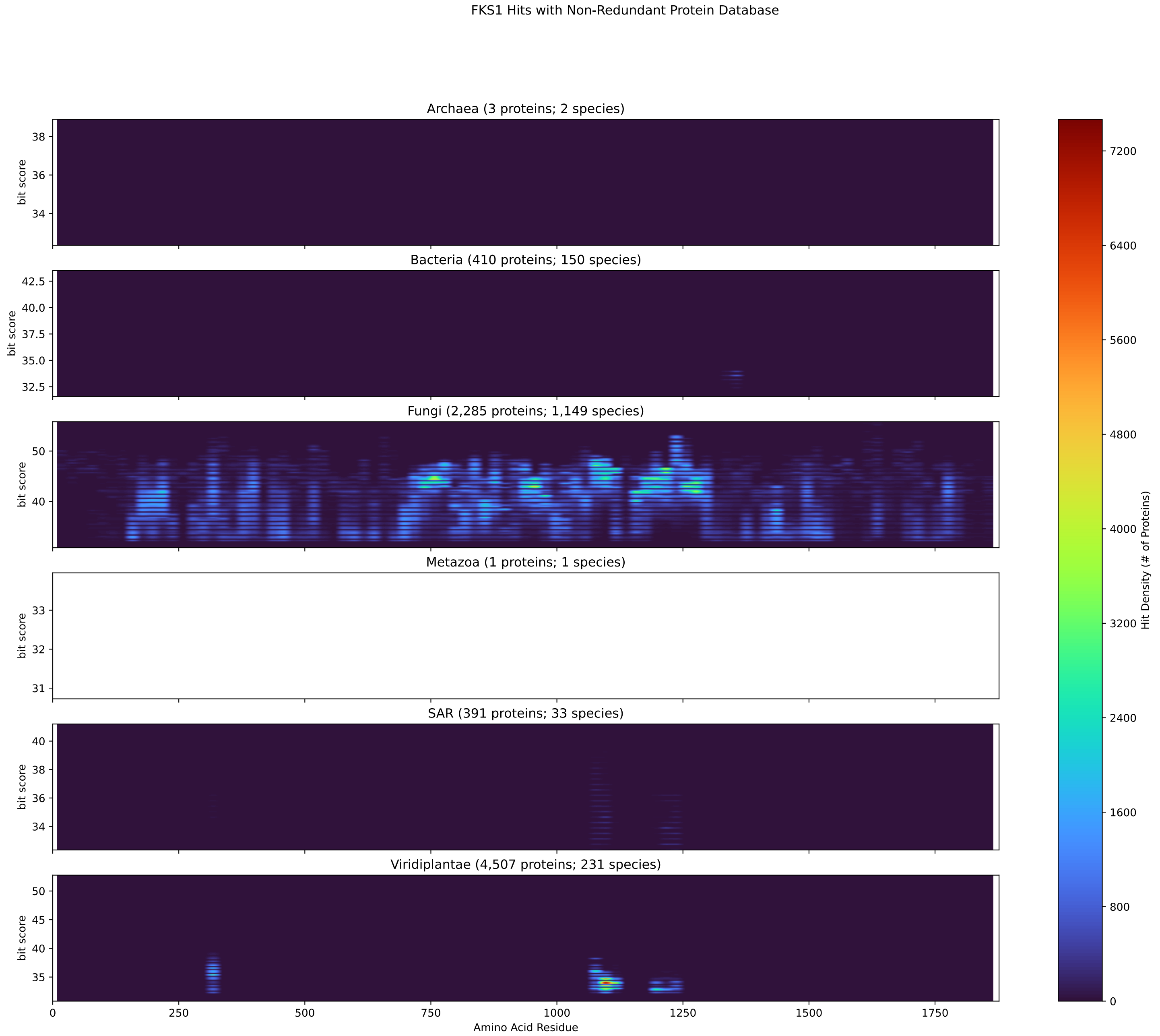

Figure S203: Histogram of Fks1 hits against NR in 20-aa windows

Species	Hit Protein	Hit Length (a.a.)	evalue	align_len	bit_score	identity	positive	score	gaps	% identity	% positive
H.sapiens	TATA-binding protein-associated factor 2N isoform 2 Homo sapiens	104	0.025	104	41.9726	32	47	97	20	1.7	2.5
N.glabratus	uncharacterized protein CAGL0M13827g Nakaseomyces glabratus	1765	0	1765	1888.62	953	1257	4891	73	50.8	67.0
H.capsulatum	glucan synthase Histoplasma capsulatum G186AR	1879	0	1879	2468.34	1208	1449	6396	56	64.4	77.2
C.neoformans.JEC21	1,3-beta-glucan synthase, putative Cryptococcus neoformans var. neoformans JEC21	1808	0	1808	2004.56	997	1271	5192	59	53.1	67.7
C.neoformans.grubii.H99	1,3-beta-glucan synthase component FKS1 Cryptococcus neoformans var. grubii H99	1782	0	1782	1998.79	991	1260	5177	55	52.8	67.1
C.neoformans.B.3501A	hypothetical protein CNBN2360 Cryptococcus neoformans var. neoformans B-3501A	1808	0	1808	2005.33	997	1271	5194	59	53.1	67.7
C.parapsilosis	uncharacterized protein CPAR2 109680 Candida parapsilosis	1690	0	1690	1472.99	763	1085	3812	136	40.6	57.8
C.tropicalis	1,3-beta-glucan synthase component GLS1, partial Candida tropicalis MYA-3404	485	0	485	684.1	328	398	1764	8	17.5	21.2
C.auris	hypothetical protein Candida auris	1702	0	1702	1627.45	810	1137	4213	50	43.2	60.6
C.albicans	Gsl1p Candida albicans SC5314	1689	0	1689	1486.09	783	1068	3846	139	41.7	56.9
A.fumigatus	1,3-beta-glucan synthase catalytic subunit FksP Aspergillus fumigatus Af293	1898	0	1898	2426.36	1212	1463	6287	64	64.6	77.9

Table S43: Pairwise alignment info from yeast Fks1

S3.5 Fks3

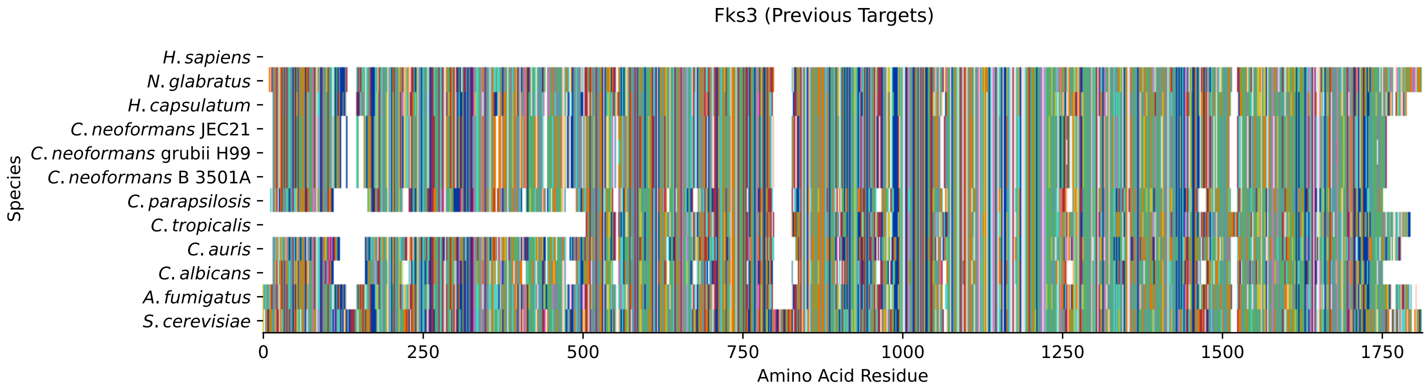

Figure S204: MSA for Fks3; cf. Table S44 for gene names and exact alignment values.

Species	Hit Protein	Hit Length (a.a.)	evalue	align_len	bit_score	identity	positive	score	gaps	% identity	% positive
H.sapiens	-	-	-	-	-	-	-	-	-	-	-
N.glabratus	uncharacterized protein CAGL0K04037g Nakaseomyces glabratus	1787	0	1787	2006.88	980	1278	5198	63	54.9	71.6
H.capsulatum	glucan synthase Histoplasma capsulatum G186AR	1758	0	1758	2002.64	975	1254	5187	73	54.6	70.2
C.neoformans.JEC21	1,3-beta-glucan synthase, putative Cryptococcus neoformans var. neoformans JEC21	1723	0	1723	1706.42	863	1149	4418	77	48.3	64.3
C.neoformans.grubii.H99	1,3-beta-glucan synthase component FKS1 Cryptococcus neoformans var. grubii H99	1723	0	1723	1702.18	861	1143	4407	77	48.2	64.0
C.neoformans.B.3501A	hypothetical protein CNBN2360 Cryptococcus neoformans var. neoformans B-3501A	1723	0	1723	1706.03	864	1149	4417	77	48.4	64.3
C.parapsilosis	uncharacterized protein CPAR2 109680 Candida parapsilosis	1725	0	1725	1459.12	771	1083	3776	167	43.2	60.6
C.tropicalis	1,3-beta-glucan synthase component GLS1 Candida tropicalis MYA-3404	1287	0	1287	1488.01	751	953	3851	51	42.0	53.4
C.auris	hypothetical protein Candida auris	1749	0	1749	1512.28	787	1111	3914	103	44.1	62.2
C.albicans	Gsl1p Candida albicans SC5314	1720	0	1720	1500.34	792	1090	3883	175	44.3	61.0
A.fumigatus	1,3-beta-glucan synthase catalytic subunit FksP Aspergillus fumigatus Af293	1782	0	1782	1965.27	994	1271	5090	74	55.7	71.2

Table S44: Pairwise alignment info from yeast Fks3

S3.6 Gsc2

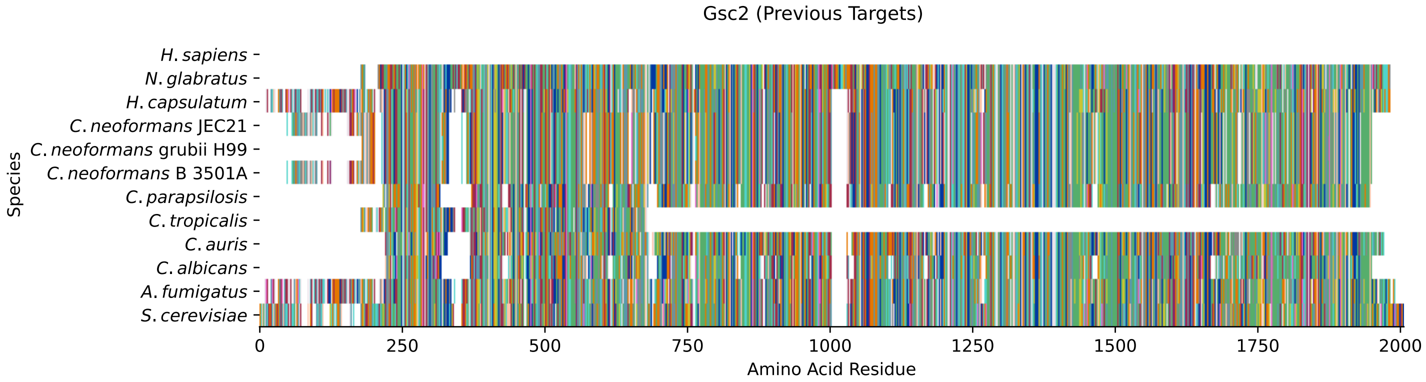

Figure S205: MSA for Gsc2; cf. Table S45 for gene names and exact alignment values.

Species	Hit Protein	Hit Length (a.a.)	evalue	align_len	bit_score	identity	positive	score	gaps	% identity	% positive
H.sapiens	-	-	-	-	-	-	-	-	-	-	-
N.glabratus	uncharacterized protein CAGL0M-13827g Nakaseomyces glabratus	1787	0	1787	1897.09	959	1266	4913	66	50.6	66.8
H.capsulatum	glucan synthase Histoplasma ca-psulatum G186AR	1905	0	1905	2468.73	1207	1450	6397	82	63.7	76.5
C.neoformans.JEC21	1,3-beta-glucan synthase, puta-tive Cryptococcus neoformans v-ar. neoformans JEC21	1813	0	1813	2006.11	997	1266	5196	74	52.6	66.8
C.neoformans.grubii.H99	1,3-beta-glucan synthase compo-nent FKS1 Cryptococcus neoform-ans var. grubii H99	1733	0	1733	2009.96	986	1243	5206	59	52.0	65.6
C.neoformans.B.3501A	hypothetical protein CNBN2360 Cryptococcus neoformans var. n-eoformans B-3501A	1813	0	1813	2006.11	996	1264	5196	74	52.5	66.7
C.parapsilosis	uncharacterized protein CPAR2 109680 Candida parapsilosis	1687	0	1687	1488.01	775	1094	3851	130	40.9	57.7
C.tropicalis	1,3-beta-glucan synthase compo-nent GLS1, partial Candida tro-picalis MYA-3404	489	0	489	678.322	331	391	1749	7	17.5	20.6
C.auris	hypothetical protein Candida a-uris	1704	0	1704	1632.08	812	1139	4225	54	42.8	60.1
C.albicans	Gsl1p Candida albicans SC5314	1684	0	1684	1478.77	786	1069	3827	139	41.5	56.4
A.fumigatus	1,3-beta-glucan synthase catal-ytic subunit FksP Aspergillus fumigatus Af293	1914	0	1914	2419.81	1217	1461	6270	80	64.2	77.1

Table S45: Pairwise alignment info from yeast Gsc2

S4 Results: Genus-specific Good Targets

S4.1 Ccc1

S4.1.1 WHO Critical Pathogens

Species	Hit Protein	Hit Length (a.a.)	evalue	align_len	bit_score	identity	positive	score	gaps	% identity	% positive
H.sapiens	-	-	-	-	-	-	-	-	-	-	-
N.glabratus	XP_445212.1 uncharacterized p-rotein CAGL0C00693g Nakaseomyc-es glabratus	322	2.5e-133	322	379.793	224	256	974	23	69.6	79.5
H.capsulatum	XP_045287029.1 CCC1 Histoplas-ma capsulatum G186AR	246	1.4e-50	246	168.318	94	139	425	30	29.2	43.2
C.neoformans.JEC21	XP_568012.1 membrane fraction protein, putative Cryptococcu-s neoformans var. neoformans J-EC21	178	5.4e-22	178	93.5893	72	100	231	27	22.4	31.1
C.neoformans.grubii.H99	XP_012048745.1 membrane fract-ion protein Cryptococcus neofo-rmans var. grubii H99	177	4.8e-23	177	96.6709	73	100	239	25	22.7	31.1
C.neoformans.B.3501A	XP_773602.1 hypothetical prot-ein CNBI2160 Cryptococcus neof-ormans var. neoformans B-3501A	178	5.2e-22	178	93.5893	72	100	231	27	22.4	31.1
C.parapsilosis	XP_036667882.1 uncharacterize-d protein CPAR2 405240 Candida parapsilosis	325	3.1e-96	325	285.804	165	218	730	14	51.2	67.7
C.tropicalis	XP_002547990.1 conserved hypo-thetical protein Candida tropi-calis MYA-3404	325	1.1e-99	325	294.278	169	216	752	18	52.5	67.1
C.auris	XP_028891400.2 hypothetical p-rotein Candida auris	264	1.2e-104	264	306.605	152	196	784	3	47.2	60.9
C.albicans	XP_712749.1 Ccc1p Candida alb-icans SC5314	325	1.2e-95	325	283.878	165	212	725	18	51.2	65.8
A.fumigatus	XP_754622.1 vacuolar iron tra-nsporter Ccc1, putative Asperg-illus fumigatus Af293	265	1.6e-60	265	194.512	116	156	493	13	36.0	48.4

Table S46: Pairwise alignment info from *S. cerevisiae* Ccc1 with all pathogens.

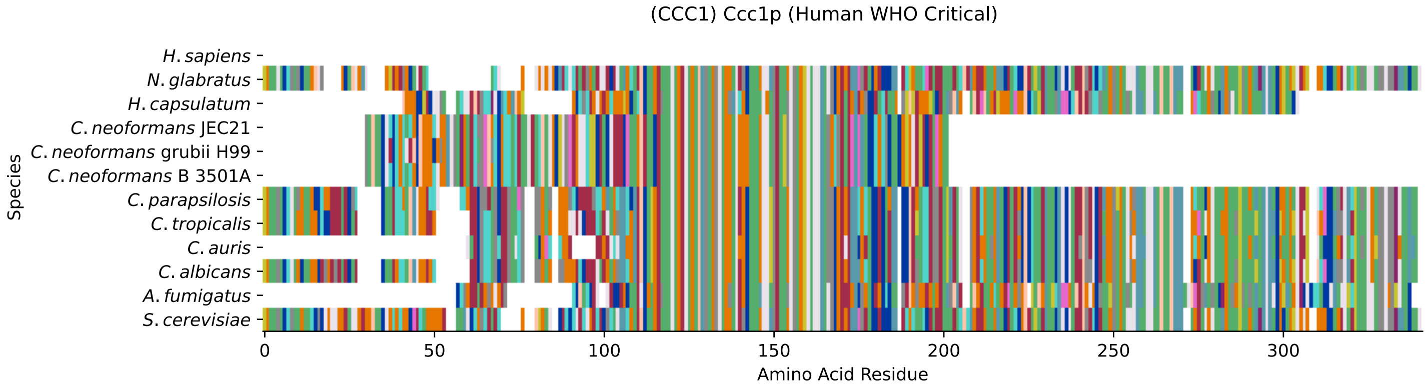

Figure S206: MSA of Ccc1, with all pathogens in the WHO Critical Pathogens group. Cf. Table S46 and Fig. S207.

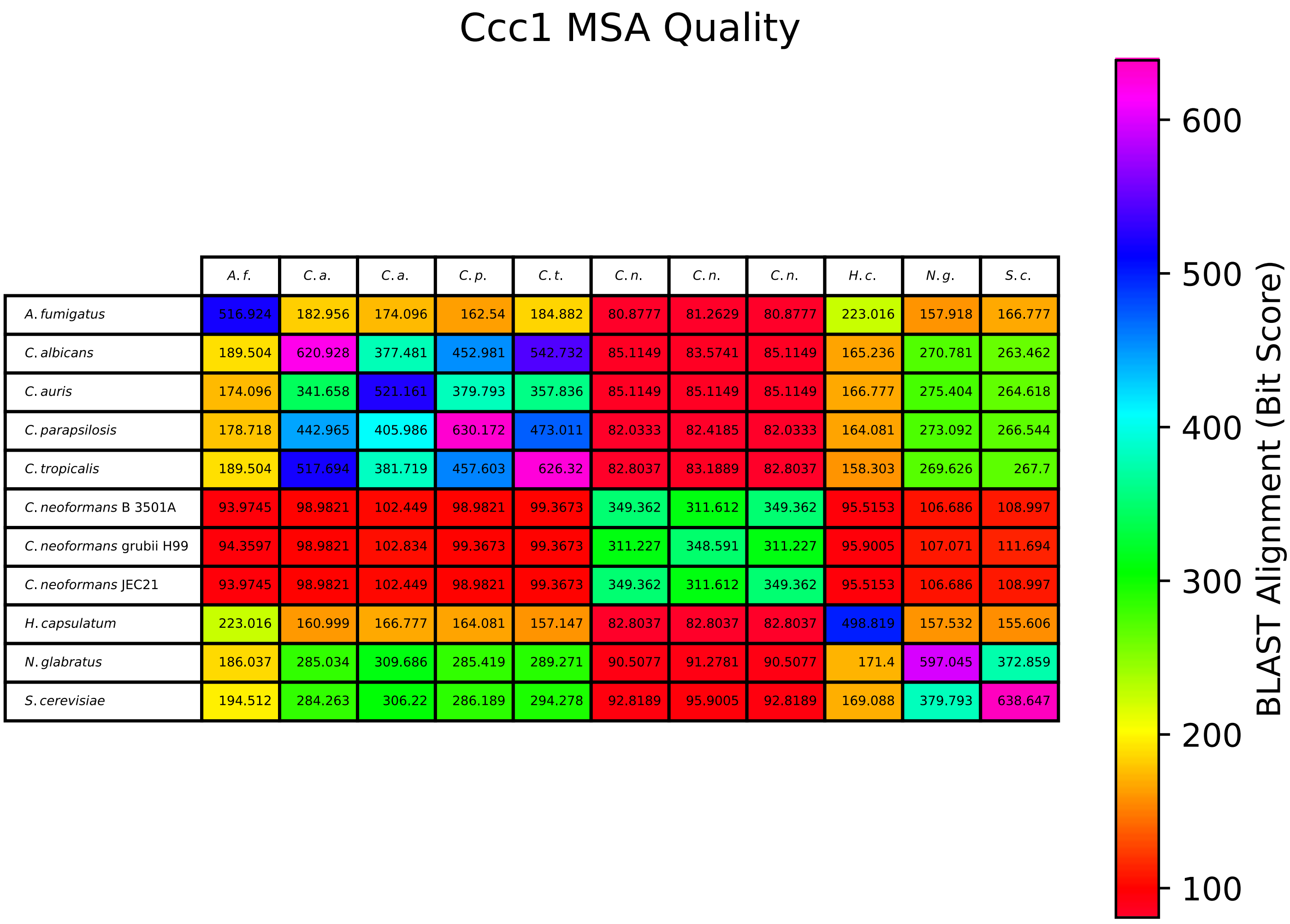

	A. f.	*C. a.*	*C. a.*	*C. p.*	*C. t.*	*C. n.*	*C. n.*	*C. n.*	*H. c.*	*N. g.*	*S. c.*
A. fumigatus	516.924	182.956	174.096	162.54	184.882	80.8777	81.2629	80.8777	223.016	157.918	166.777
C. albicans	189.504	620.928	377.481	452.981	542.732	85.1149	83.5741	85.1149	165.236	270.781	263.462
C. auris	174.096	341.658	521.161	379.793	357.836	85.1149	85.1149	85.1149	166.777	275.404	264.618
C. parapsilosis	178.718	442.965	405.986	630.172	473.011	82.0333	82.4185	82.0333	164.081	273.092	266.544
C. tropicalis	189.504	517.694	381.719	457.603	626.32	82.8037	83.1889	82.8037	158.303	269.626	267.7
C. neoformans B 3501A	93.9745	98.9821	102.449	98.9821	99.3673	349.362	311.612	349.362	95.5153	106.686	108.997
C. neoformans grubii H99	94.3597	98.9821	102.834	99.3673	99.3673	311.227	348.591	311.227	95.9005	107.071	111.694
C. neoformans JEC21	93.9745	98.9821	102.449	98.9821	99.3673	349.362	311.612	349.362	95.5153	106.686	108.997
H. capsulatum	223.016	160.999	166.777	164.081	157.147	82.8037	82.8037	82.8037	498.819	157.532	155.606
N. glabratus	186.037	285.034	309.686	285.419	289.271	90.5077	91.2781	90.5077	171.4	597.045	372.859
S. cerevisiae	194.512	284.263	306.22	286.189	294.278	92.8189	95.9005	92.8189	169.088	379.793	638.647

Figure S207: 2-D MSA of Ccc1, with all pathogens in the group. Cf. Table S46.

S4.1.2 Aspergillus

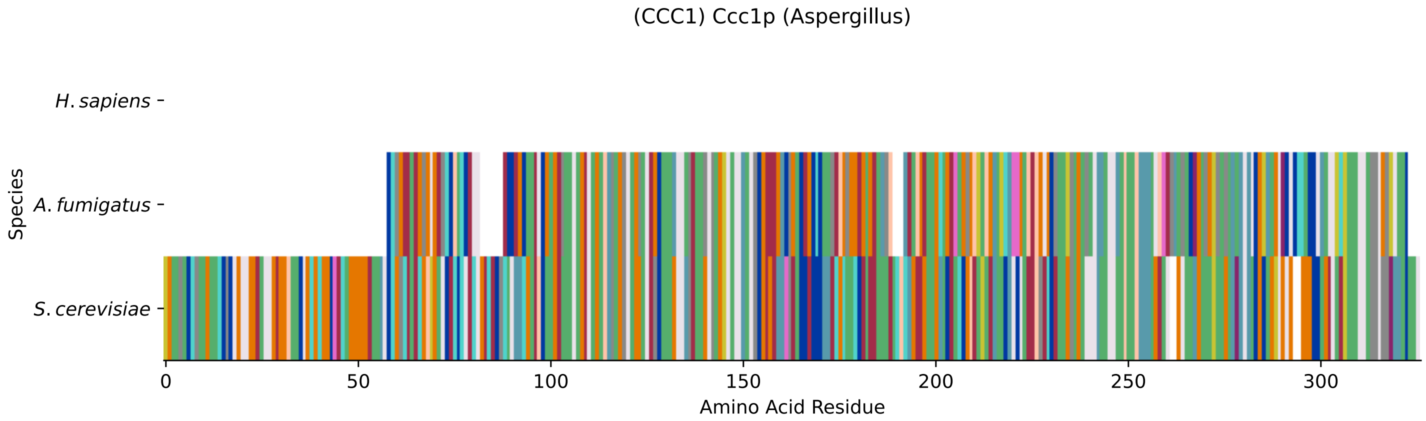

Figure S208: MSA of Ccc1 with only *Aspergillus*, with all genes in the group. Cf. Table S46 and Fig. S206.

S4.1.3 Candida

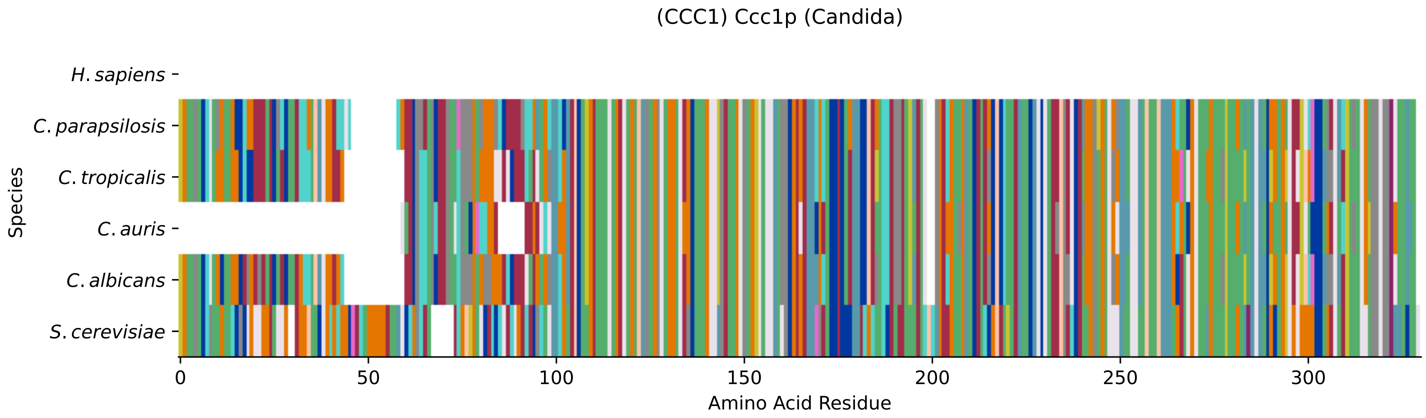

Figure S209: MSA of Ccc1 with only *Candida*, with all genes in the group. Cf. Table S46 and Fig. S206.

S4.1.4 Cryptococcus

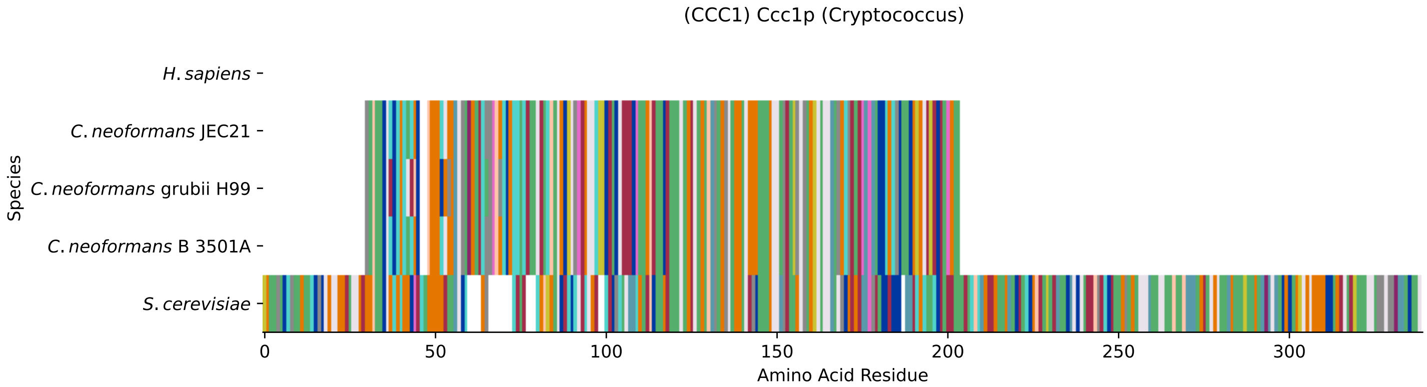

Figure S210: MSA of Ccc1 with only *Cryptococcus*, with all genes in the group. Cf. Table S46 and Fig. S206.

S4.1.5 Top 10 Agricultural Fungal Pathogens

Species	Hit Protein	Hit Length (a.a.)	evalue	align_len	bit_score	identity	positive	score	gaps	% identity	% positive
G.max	XP_003530927.1 vacuolar iron transporter 1 Glycine max	222	2.2e-48	222	163.31	85	136	412	9	26.4	42.2
Z.mays	XP_008670169.1 vacuolar iron transporter 2 Zea mays	211	6.6e-46	211	156.762	87	129	395	12	27.0	40.1
S.tuberosum	XP_006355092.2 PREDICTED: vacuolar iron transporter 1, partial Solanum tuberosum	223	8.1e-45	223	154.836	86	132	390	13	26.7	41.0
O.sativa	XP_015636127.1 vacuolar iron transporter 1.1 Oryza sativa Japonica Group	221	6.6e-48	221	161.77	86	136	408	9	26.7	42.2
H.sapiens	-	-	-	-	-	-	-	-	-	-	-
U.maydis	XP_011388301.1 uncharacterized protein UMAG 06298 Ustilago maydis 521	287	1e-44	287	155.606	102	146	392	63	31.7	45.3
P.striiformis	XP_047807231.1 hypothetical protein Pst134EA 011404 Puccinia striiformis f. sp. tritici	231	9.1e-42	231	148.673	89	127	374	44	27.6	39.4
P.oryzae	mRNA M BR32 EuGene 00028701-p1 — transcript=mRNA M BR32 EuGene 00028701 — gene=M BR32 EuGene 00028701 — organism=Pyricularia oryzae BR32 — gene product=unspecified product — transcript product=unspecified product — location=BR32 scaffold000-02:3526435-3527539(+) — protein length=338 — sequence SO=supercontig — SO=protein coding gene — is pseudo=false	196	4.7e-32	196	120.939	88	114	302	17	27.3	35.4
P.triticina	XP_053019341.1 uncharacterized protein PtA15 4A235 Puccinia triticina	273	4.7e-48	273	164.851	104	147	416	48	32.3	45.7
P.graminis	XP_003334675.1 hypothetical protein PGTG 16534 Puccinia graminis f. sp. tritici CRL 75-36--700-3	301	9.7e-43	301	149.443	98	153	376	68	30.4	47.5
M.graminicola	ZTRI 5.300.mRNA-p1 — transcript=ZTRI 5.300.mRNA — gene=ZTRI 5.300 — organism=Zymoseptoria tritici IPO323 — gene product=-similar to vacuolar iron transporter Ccc1 — transcript product=similar to vacuolar iron transporter Ccc1 — location=Ztri chr 5:1094678-1095529(-) — protein length=283 — sequence SO-=chromosome — SO=protein coding gene — is pseudo=false	229	2.9e-57	229	185.652	104	138	470	6	32.3	42.9
F.graminearum	XP_011327657.1 hypothetical protein FGSG 07832 Fusarium graminearum PH-1	220	3.1e-49	220	164.466	91	135	415	5	28.3	41.9
C.truncatum	XP_036587805.1 vacuolar iron transporter Colletotrichum truncatum	213	2.6e-58	213	189.889	108	136	481	9	33.5	42.2
B.cinerea	XP_001546942.1 hypothetical protein BCIN 11g00770 Botrytis cinerea B05.10	218	8.6e-47	218	159.458	112	139	402	8	34.8	43.2
B.graminis	-	-	-	-	-	-	-	-	-	-	-

Table S47: Pairwise alignment info from *S. cerevisiae* Ccc1 with all pathogens.

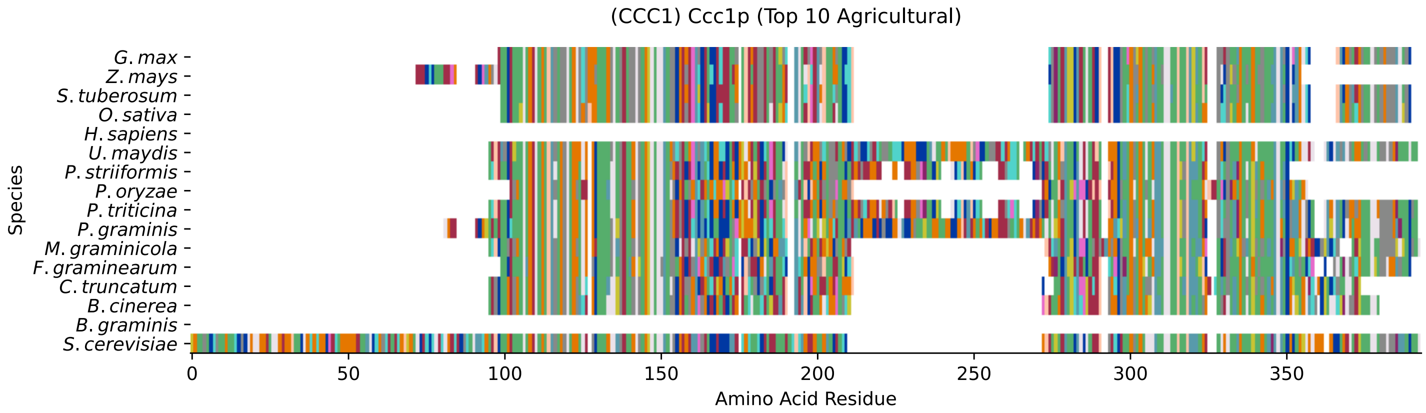

Figure S211: MSA of Ccc1, with all pathogens in the Top 10 Agricultural Fungal Pathogens group. Cf. Table S47 and Fig. S212.

Ccc1 MSA Quality

	B.g.	B.c.	C.t.	F.g.	M.g.	P.g.	P.t.	P.o.	P.s.	U.m.	S.c.
B. graminis	0	0	0	0	0	0	0	0	0	0	0
B. cinerea	0	433.335	228.024	145.976	180.644	116.316	133.65	167.933	128.642	128.257	171.4
C. truncatum	0	197.978	427.172	150.599	192.971	90.8929	132.88	207.608	127.487	87.8113	167.548
F. graminearum	0	117.087	150.599	437.187	149.828	138.658	141.354	97.4413	134.42	140.584	141.354
M. graminicola	0	145.976	192.971	149.828	453.751	108.997	124.02	154.066	117.857	125.176	158.303
P. graminis	0	85.1149	90.8929	96.2857	108.997	588.956	301.982	74.7146	239.195	232.646	124.79
P. triticina	0	103.605	132.88	141.354	124.02	301.982	546.584	82.0333	411.764	244.202	136.732
P. oryzae	0	171.4	235.728	128.257	185.267	91.2781	105.531	377.481	103.605	110.153	132.109
P. striiformis	0	102.064	134.42	137.887	120.168	248.054	439.499	82.0333	463.381	212.231	135.576
U. maydis	0	92.8189	87.8113	140.584	125.176	232.646	244.202	85.5001	204.912	565.074	124.79
S. cerevisiae	0	160.229	187.578	162.54	184.496	149.443	163.31	119.398	147.902	152.91	638.647

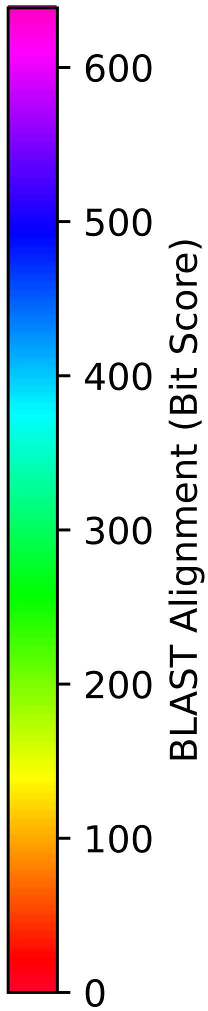

Figure S212: 2-D MSA of Ccc1, with all pathogens in the group. Cf. Table S47.
